# Four near-complete genome assemblies reveal the landscape and evolution of centromeres in Salicaceae

**DOI:** 10.1186/s13059-025-03578-7

**Published:** 2025-05-02

**Authors:** Yubo Wang, Lulu Zhao, Deyan Wang, Kai Chen, Tiannan Luo, Jianglin Luo, Chengzhi Jiang, Zhoujian He, Heng Huang, Jiaxiao Xie, Yuanzhong Jiang, Jianquan Liu, Tao Ma

**Affiliations:** 1 Key Laboratory for Bio-Resource and Eco-Environment of Ministry of Education & Sichuan Zoige Alpine Wetland Ecosystem National Observation and Research Station, College of Life Science, Sichuan University, Chengdu, China; 2 School of Life Science and Technology, University of Electronic Science and Technology of China, Chengdu, 610054 China; 3 State Key Laboratory of Herbage Innovation and Grassland Agro-Ecosystem, College of Ecology, Lanzhou University, Lanzhou, 730000 China

## Abstract

**Background:**

Centromeres play a crucial role in maintaining genomic stability during cell division. They are typically composed of large arrays of tandem satellite repeats, which hinder high-quality assembly and complicate our efforts to understand their evolution across species. Here, we use long-read sequencing to generate near-complete genome assemblies for two *Populus* and two *Salix* species belonging to the Salicaceae family and characterize the genetic and epigenetic landscapes of their centromeres.

**Results:**

The results show that only limited satellite repeats are present as centromeric components in these species, while most of them are located outside the centromere but exhibit a homogenized structure similar to that of the *Arabidopsis* centromeres. Instead, the Salicaceae centromeres are mainly composed of abundant transposable elements, including *CRM* and *ATHILA*, while LINE elements are exclusively discovered in the poplar centromeres. Comparative analysis reveals that these centromeric repeats are extensively expanded and interspersed with satellite arrays in a species-specific and chromosome-specific manner, driving rapid turnover of centromeres both in sequence compositions and genomic locations in the Salicaceae.

**Conclusions:**

Our results highlight the dynamic evolution of diverse centromeric landscapes among closely related species mediated by satellite homogenization and widespread invasions of transposable elements and shed further light on the role of centromere in genome evolution and species diversification.

**Supplementary Information:**

The online version contains supplementary material available at 10.1186/s13059-025-03578-7.

## Background

Centromeres are essential chromosomal regions that ensure the accurate segregation of replicated chromosomes during cell division [1, 2]. They are typically located in heterochromatin and exhibit unique epigenetic modifications when compared to other parts of the genome [3, 4]. One of the most conserved epigenetic mechanisms is the packaging of DNA sequences around the centromere-specific histone H3 variant, CENH3, in plants (CENP-A in animals), which establishes the epigenetic identity and function of the centromere [2, 5–8]. They play a significant role in driving karyotype evolution and speciation by shaping the organization of genomic architecture and chromatin composition [9–12]. However, due to the presence of large amounts of repetitive sequences in centromeres, sequencing and assembly of them have been challenging until recent advancements in long-read DNA sequencing technologies and assembly methods [12–16]. Our understanding of the genetic composition and epigenetic landscape of centromeres, and their contributions to genome evolution remains limited and requires further investigation.

Despite their conserved role during chromosome segregation, centromeres exhibit high structural and sequence diversity both within and between species, reflecting their rapid evolution. For example, centromeres of many species are commonly composed of large tandem repeat arrays (TRAs) organized by centromere-specific satellite DNA with monomer length ranging from 100 to 200 bp, such as the α satellite DNA in human (*αSat*, ~ 171 bp) and *AthCEN178* in *Arabidopsis thaliana* (formerly *CEN180*) [12, 17–19]. These satellite DNA can occur as different subtypes, repeated in a head-to-tail orientation and further arranged into higher-order repeats (HORs), which extend from kilobases (kb) to megabases (Mb) in length [12, 20]. The centromeric TRAs in humans tend to undergo rapid evolution through a “layered expansion” pattern during the homogenization process of satellite DNA, resulting in frequent changes in structure and size and high genetic diversity among individuals [12, 17, 21]. Moreover, in some plants and animals, specific transposable elements (TEs), especially long terminal repeat (LTR) retrotransposons, are interspersed with centromeric satellite repeats, providing potential sites for CENH3 loading. For example, homologous elements of the *Ty3-gypsy* group of centromeric retrotransposons (*CR*) are located within functional centromeric regions of maize (*CRM*), wheat (*CRW*), rice (*CRR*), and cabbage [22–25]. In addition, recent study has shown that the rapid cycles of retrotransposons invasions by *ATHILA* elements and purging through satellite homogenization contribute to centromere diversity in *Arabidopsis* [12, 26]. Notably, the discovery of satellite-free centromeres in potato and equids suggests that satellite repeats are not essential for centromere function [27, 28]. Overall, these studies have provided valuable insights into the structural organization and remarkable sequence diversity of eukaryotic centromeres. Further comparative investigation of genetic and epigenetic variations at centromeres between closely related species will facilitate a deeper understanding of centromere evolution.

*Populus* (poplars) and *Salix* (willows) are two sister genera in the Salicaceae family with approximately 100 and 500 species, respectively, many of which are woody trees of significant cultural, medical, ecological, and economic importance [29, 30]. As essential components of temperate, boreal, and arctic forest ecosystems throughout the Northern Hemisphere, they have long served as a model system for tree biology research, including molecular mechanisms, speciation, sex chromosomes, phenotypic diversification, environmental adaptation, and ecosystem services [31–39]. The genomes of many species in these two genera have been sequenced, and comparative analysis revealed that *Populus* and *Salix* shared the “Salicoid” whole genome duplication event near the K-Pg boundary and have maintained a relatively stable karyotype with 2n = 38 in diploid organism, along with long term and persistent interspecific gene flow during their evolutionary history [40–46]. However, the detailed composition of centromeric sequences and their evolution in Salicaceae have remained elusive. Recent study on hybrid aspen (*P. tremula* × *P. alba*) showed that both TRA-based and array-free centromeres exist in its genome, but the most abundant TRAs are located outside the centromeres [47]. In addition, a long interspersed nuclear element (LINE) retrotransposon was recently identified within the centromeres of *P. trichocarpa* [48]. These studies suggest that Salicaceae centromeres may have a distinct sequence structure and composition compared to those of other species. To address this issue, we generated near-complete genome assemblies of four Salicaceae species, including *P. alba* var. *pyramidalis*, *P. euphratica*, *S. chaenomeloides*, and *S. arbutifolia*, representing different sections or subgenera of the genera *Populus* and *Salix*. We characterized the genetic and epigenetic landscapes of their centromeres and compared the dynamic changes in their sequence composition, epigenetic features, and chromosome rearrangements mediated by centromere turnover and repositioning within and between species. Our findings provide new insights into the extensive diversity and rapid turnover of centromeres in closely related species, as well as their potential roles in genome evolution and species diversification.

## Results

### Telomere-to-telomere haplotype-resolved genome assembly of four Salicaceae species

**Fig. 1:**
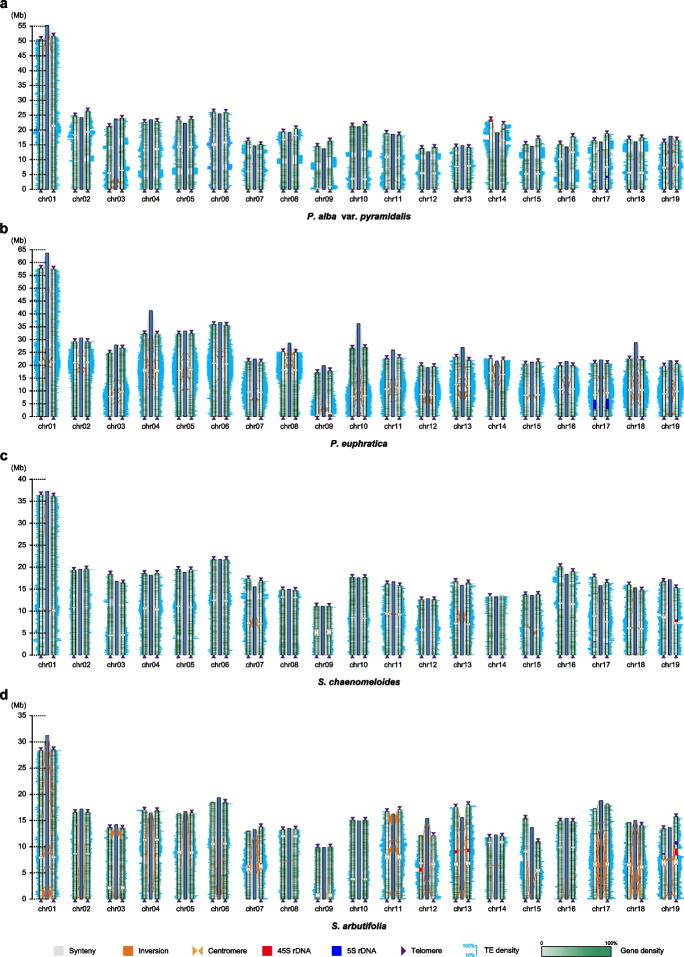
Syntenic alignments among assembled Salicaceae haplotype genomes and their reference genome of *P. alba* var. *pyramidalis* (**a**), *P. euphratica* (**b**), *S. chaenomeloides* (**c**), and *S. arbutifolia* (**d**). The syntenic regions among haplotype I (left), reference genome (middle) and haplotype II (right) are shown by gray lines. Centromeres are shown as Khaki triangles. Telomeres are shown as black triangles at chromosome ends. Inversions between each haplotype and the reference genome are shown in orange blocks. 5S and 45S rDNA regions are shown as blue and yellow rectangles, respectively. TE density of each chromosome is shown as cyan histograms

We sequenced and assembled the haplotype-resolved genomes for four Salicaceae species by combining PacBio HiFi reads (> 60 ×) and high-throughput chromosome conformation capture (Hi-C) data (> 600 ×). The estimated genome size of *P. alba* var. *pyramidalis*, *P. euphratica*, *S. chaenomeloides*, and *S. arbutifolia* was 423.05, 528.42, 358.78, and 328.39 Mb, respectively. And the estimated heterozygosity was 1.58, 1.17, 1.23, and 0.67%, respectively (Additional file 1: Fig. S1; Additional file 2: Table S1). The length of the assembled eight genomes ranged from 295.91 to 497.16 Mb, with each haplotype having 29 to 102 contigs anchored onto 19 pseudo-chromosomes and a contig N50 ranging from 9.75 to 21.42 Mb (Fig. 1; Additional file 1: Fig. S2; Additional file 2: Tables S2-S3). Collinearity analysis showed a high degree of similarity between the two haplotype genomes of each species. In addition to the chromosomal rearrangements of chromosome 1 (chr01) and chr16 between *Populus* and *Salix* species, all the other chromosomes had clear homologous relationships between species (Additional file 1: Fig. S3), which is consistent with previous reports [44]. These assemblies also showed extensive collinearity with their previously released genomes [36, 49, 50], with some chromosomal inversions around genomic regions that are rich in repetitive elements (Fig. 1).

A total of 29,381 to 37,077 protein-coding genes were predicted in these haplotype genomes, and 25,237 to 29,596 allele pairs were identified between the two haplotypes of each species, accounting for 79.28 to 87.46% of the total genes (Additional file 2: Table S2). We also identified telomeric repeat sequences (5′-TTTAGGG- 3′)_n_ at both distal ends of the chromosomes, with 27, 37, and 35 telomeric locations detected in the haplotype I of *S. arbutifolia*, and haplotype I and II of *S. chaenomeloides*, respectively, while 38 telomeric locations were identified on all 19 chromosomes in the remaining five haplotype assemblies (Fig. 1; Additional file 2: Table S4). Furthermore, we identified a 5S rDNA array on chr19 of the *S. arbutifolia* haplotypes and on chr17 of all other assemblies, containing 174 to 1359 5S rDNAs with a total length of 91.15 kb to 4.09 Mb (Fig. 1; Additional file 2: Table S5). In contrast, the distribution of 45S rDNA showed a high degree of diversity among species. Only one 45S rDNA array was identified on chr14, chr09, and chr19 of *P. alba* var. *pyramidalis*, *P. euphratica*, and *S. chaenomeloides*, respectively, while in *S. arbutifolia*, four arrays were identified on chr07, chr12, chr13, and chr19 (Fig. 1; Additional file 2: Table S5). All these results confirmed the high quality of our nearly complete genome assemblies.

### Identification and epigenetic landscape of Salicaceae centromeres

**Fig. 2:**
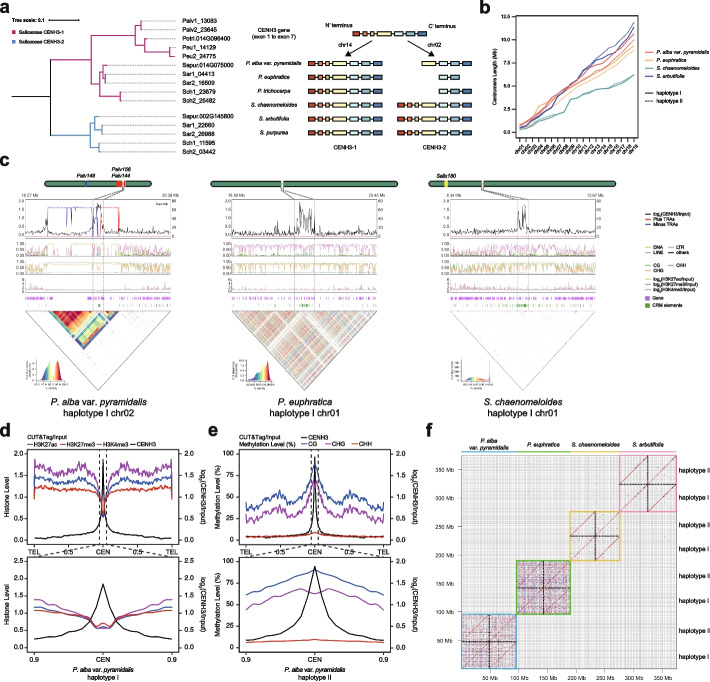
Profiles of Salicaceae centromeres. **a** Salicaceae CENH3 genes. The phylogenetic tree (left) was constructed by CENH3 genes in assembled genomes as well as the published genome of *P. trichocarpa* and *S. purpurea*, and branches are color-coded according to CENH3 type. The schematic illustration of Salicaceae CENH3 genes (right) are shown as boxes representing exons. The two copies of CENH3 gene are identified in willows, while the CENH3-2 loses exons at the N’ terminus and became dysfunctional in poplars. **b** Line graph of cumulative centromere length in different genomes. **c** Characteristics and epigenetics for representative centromere of *P. alba* var. *pyramidalis* haplotype I, *P. euphratica* haplotype I, and *S. chaenomeloides* haplotype I. Plots from top to bottom separately represent TRAs on forward (red) and reverse (blue) strands and CENH3 CUT&Tag distribution per 10-kb, transposable element distribution per 10-kb, DNA methylation level per 10-kb, histone modification level per 10-kb, gene distribution, *CRM* element distribution and sequence similarity on centromeres and adjacent regions. Regions marked between grey dashlines represent centromeres. **d** The chromosomal-scale histone modification feature of *P. alba* var. *pyramidalis* haplotype I, and the lower figure represents the closed-up plot of centromeres. **e** Same as **d** but showing the WGBS methylation feature. **f** Dot plot of syntenic pericentromeres from all genome assemblies using a 500-bp search window. Red and blue points indicate forward- and reverse-strand similarly, respectively

We identified two members of the CENH3 gene, CENH3-1, and CENH3-2, in the willow genome, while only CENH3-1 was found in the poplar genome, with the ortholog of CENH3-2 was pseudogenized (Fig. 2a; Additional file 1: Fig. S4). We therefore performed both immunofluorescence assay and the cleavage under targets and tagmentation (CUT&Tag) sequencing using anti-CENH3-1 and anti-CENH3-2 antibodies to identify the centromeric locations of these species, except for *S. arbutifolia* due to the lack of fresh material (Fig. 2a; Additional file 1: Fig. S5; Additional file 2: Table S1). The boundaries of the core centromere on each chromosome were precisely defined by CENH3 occupancy, where the two CENH3 members were almost coincident in the enriched locations in *S. chaenomeloides*, whereas CENH3-2 was not enriched in the two poplar species, consistent with the loss of this gene member (Additional file 1: Fig. S6; Additional file 2: Table S6). The sizes of the core centromeres ranged from 0.21 to 1.32 Mb in *P. euphratica*, from 0.15 to 0.99 Mb in *P. alba* var. *pyramidalis*, and from 0.02 to 1.37 Mb in *S. chaenomeloides*. A similar range of lengths was also obtained using the MACS2 software (Fig. 2b; Additional file 1: Figs. S7-S12; Additional file 2: Tables S6-S7). For each haplotype, we identified only 1 to 5 gaps in these regions, indicating that the majority of the centromeres were fully assembled (Additional file 1: Figs. S7-S12; Additional file 2: Tables S2 and S6). No significant differences in centromere size were observed between the two haplotypes of the same species, and no correlation was found between centromere size and chromosome size (Additional file 2: Table S8). Moreover, we observed higher GC level, lower gene density, and higher TE density near the centromeres (Fig. 2c; Additional file 1: Figs. S6-S12).

To characterize the epigenetic organization within centromeres, we also examined the enrichment of three additional histones (H3K4me3, H3K27me3 and H3K27ac) and DNA methylation levels by scaling all chromosome arms between telomeres and centromere midpoints (Additional file 2: Table S1). The results showed that the levels of these histone modifications are reduced within the centromere relative to other regions of the genome (Fig. 2d; Additional file 1: Fig. S15). However, we found a slight increase in H3K27me3 enrichment at the centromeric midpoint in all species and in H3K27ac enrichment in two poplar species. Consistently, DNA methylation levels were also increased at the centromeres in CG, CHG, and CHH contexts (Fig. 2e; Additional file 1: Fig. S16). However, CHG DNA methylation showed relatively depletion at the centromere midpoint compared with CG methylation in all species. These observations are consistent with CENH3 replacement for H3 leading to reduced maintenance of non-CG methylation within the *Arabidopsis* centromeres [51].

Interestingly, sequence alignments showed that these centromeres exhibit substantially differences in sequence compositions and structural features between species but maintain a high degree of similarity between haplotypes of the same species (Fig. 2f). However, significant enrichment of *CRM* LTR retrotransposons was observed in the centromeres of all three species, consistent with the recent findings in other plants (Fig. 2c; Additional file 1: Figs. S6-S12) [20, 22, 52–55]. Based on this feature, we inferred the potential centromere locations in *S. arbutifolia* chromosomes, which showed similar genetic organization and DNA methylation profile to those observed in the other three Salicaceae species (Additional file 1: Figs. S13-S14 and S16; Additional file 2: Table S6). Taken together, these results supported rapid evolution and turnover of centromeric sequences in these species, but with similar epigenetic landscapes.

### Centromeric TRAs are limited and highly diverse among Salicaceae species

**Fig. 3:**
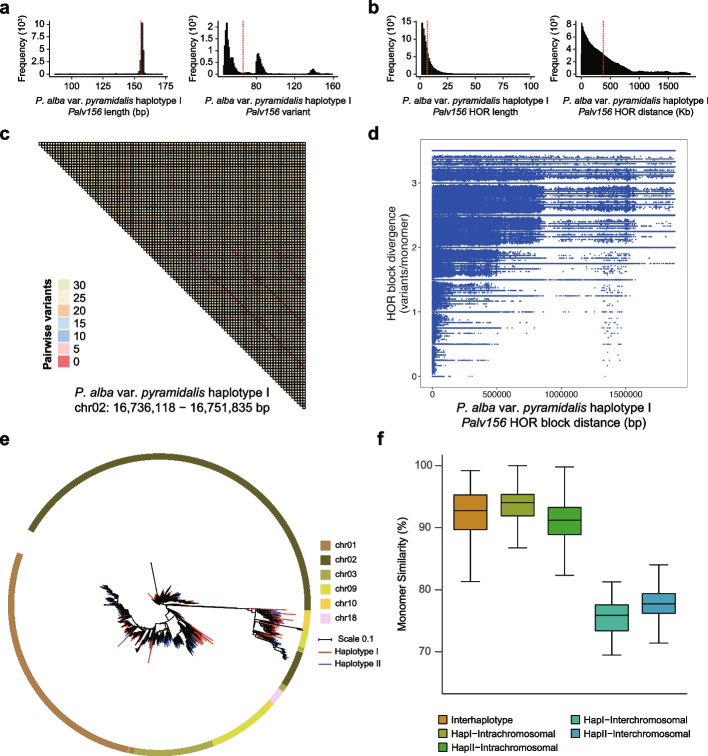
Characteristics of centromeric *Palv156* TRAs.** a** Histograms of *Palv156* monomer lengths (left) and variant distances relative to the genomewide consensus (right) in *P. alba* var. *pyramidalis* haplotype I. **b** Histograms of monomer number (length) of *Palv156* in HOR blocks (left) and the distances between HORs (right) in *P. alba* var. *pyramidalis* haplotype I. **c** Representative *Palv156* TRA region heatmap colored according to pairwise variants between *Palv156* monomers. **d** Dot plot distribution of *Palv156* HORs distance and HORs variation levels in *P. alba* var. *pyramidalis* haplotype I. **e** Phylogenetic tree of sampled *Palv148* monomers. The color of the outer circle and the tree branch represented the corresponding chromosome and genome haplotype, respectively. **f** Boxplot of *Palv156* TRAs monomer similarity comparisons in both within and between chromosomes, haplotypes, and species

We identified only 12 to 41 TRAs that showed extensive variation in total length between species, with the longest being 54.91 Mb in haplotype II of *P. alba* var. *pyramidalis* and the shortest being 1.04 Mb in haplotype I of *P. euphratica* (Additional file 1: Figs. S17-S18; Additional file 2: Table S9). More importantly, we found that only a small fraction of these TRAs located in centromeric or pericentromeric regions, and most of them are not occupied by CENH3 nucleosomes reflected by low CUT&Tag/Input enrichment (Additional file 1: Figs. S7-S14; Additional file 2: Table S9). One of the most abundant centromeric TRAs was composed of a 156-bp monomer (*Palv156*) in *P. alba* var. *pyramidalis* (Fig. 2c; Additional file 1: Figs. S7-S8 and S17a; Additional file 2: Table S9). A total of 19,203 and 39,191 *Palv156* repeats were identified in the same six centromeres (including chr01, chr02, chr03, chr09, chr10, and chr18) of the haplotypes I and II of *P. alba* var. *pyramidalis*, with their locations predominantly on the same strand within each centromere (Fig. 3a; Additional file 1: Figs. S7-S8, S17a and S19a). These monomers were organized into higher-order repeats (HORs) and exhibited a high degree of sequence similarity that is negatively related to their distance (Figs. 3b–d; Additional file 1: Figs. S19b-c). Phylogenetic analysis showed that the *Palv156* monomers were clustered according to their chromosome of origin, which was consistent with their higher intrachromosomal similarity compared to interchromosomal similarity (Figs. 3e-f).

In addition to *Palv156*, we also identified several other types of centromeric TRAs in *P. alba* var. *pyramidalis*, such as *Palv78* in chr08 and chr14, *Palv107* in chr11, chr12, chr13, and chr15, and *Palv144* in chr02 and chr16, which exhibited relatively low copy numbers but similar evolutionary characteristics to *Palv156* (Additional file 1: Fig. S17a; Additional file 2: Table S9). Overall, centromeric TRAs were identified on 14 chromosomes in both haplotypes of *P. alba* var. *pyramidalis*, although different centromere may have distinct monomer compositions, such as *Palv311* only in haplotype I, while *Palv61*, *Palv212*, and *Palv616* only in haplotype II (Additional file 1: Fig. S17a; Additional file 2: Table S9). In comparison, the other three species had centromeric TRAs on only 5 (in *P. euphratica*) or 6 (in both willow species) chromosomes, which varied in length from 5.00 kb to 3.44 Mb and were primarily composed of different satellite repeats (Additional file 1: Figs. S9-S14, S17b and S18; Additional file 2: Table S9). These findings indicated a considerable degree of sequence and structural diversity in the Salicaceae centromeres both among and within species, implying their rapid evolution and turnover. As expected, sequence similarity was found between some of these satellite repeats, such as *Peu172* and *Peu177* in *P. euphratica*, *Sch69*, and *Sch75* in *S. chaenomeloides*, and *Sar479* and *Sar491* in *S. arbutifolia* (Additional file 1: Figs. S20a-c; Additional file 2: Table S10). Interestingly, similar satellite repeats were also found between species, including *Palv107* and *Peu108*, as well as *Palv78* and *Peu80*, between *P. alba* var. *pyramidalis* and *P. euphratica* (Additional file 1: Figs. S20d-e; Additional file 2: Table S10). Further phylogenetic analysis indicated that these satellite repeats may have originated in their common ancestor and underwent independent expansion after speciation (Additional file 1: Figs. S19d-f).

### Abundant non-centromeric TRAs are relatively ancient in origin but evolved independently among Salicaceae species

**Fig. 4:**
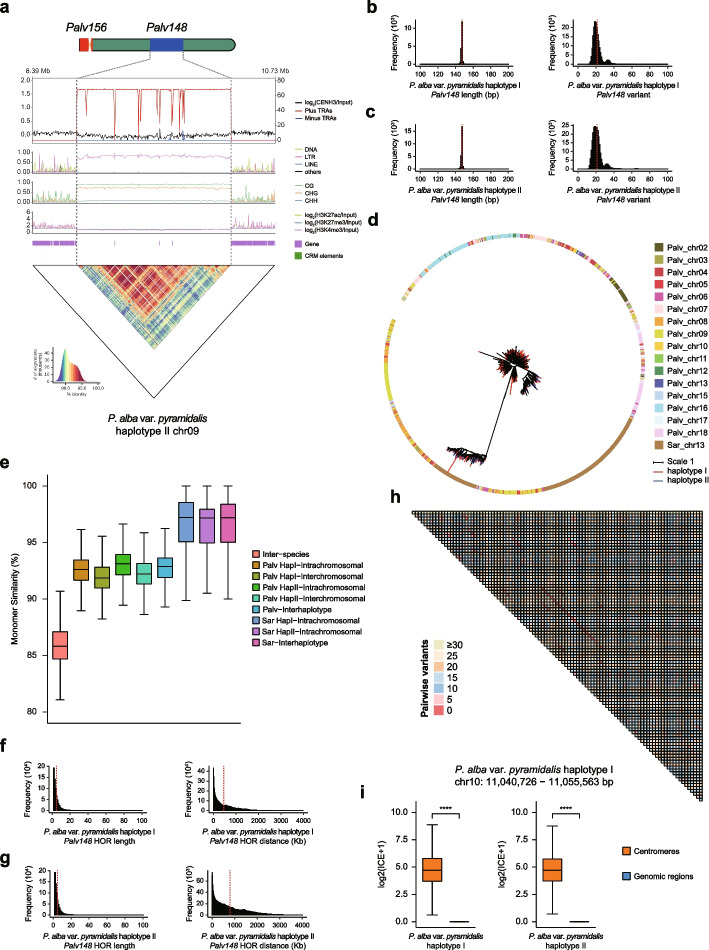
Characteristics of non-centromeric *Palv148* TRAs.** a** Characteristics and epigenetics for *Palv148* TRAs in chr09 of *P. alba* var. *pyramidalis* haplotype II. Plots from top to bottom separately represent TRAs on forward (red) and reverse (blue) strands and CENH3 CUT&Tag distribution per 10-kb, transposable element distribution per 10-kb, DNA methylation level per 10-kb, histone modification level per 10-kb, gene distribution, *CRM* element distribution and sequence similarity on centromeres and adjacent regions. **b** and **c** show histograms of *Palv148* monomer lengths (left) and variant distances relative to the genomewide consensus (right) in *P. alba* var. *pyramidalis* haplotype I and haplotype II, respectively. **d** Phylogenetic tree of sampled *Palv148* and *Sar145* monomers. The color of the outer circle and the tree branch represented the corresponding chromosome and genome haplotype, respectively. **e** Boxplot of *Palv148* and *Sar145* TRAs monomer similarity comparisons in both within and between chromosomes, haplotypes, and species. **f** and **g** show histograms of monomer number (length) of *Palv148* in HOR blocks (left) and the distances between HORs (right) in *P. alba* var. *pyramidalis* haplotype I and haplotype II, respectively. **h** Representative *Palv148* TRA region heatmap colored according to pairwise variants between *Palv148* monomers. **i** Hi-C interaction strengths comparisons of *Palv148* TRAs with centromeres and non-centromeric regions, respectively (two-tailed Wilcoxon rank-sum test, ****P ≤ 0.0001, ***P ≤ 0.001, **P ≤ 0.01, *P ≤ 0.05, ns: not significant)

Specially, we discovered that most of the remaining TRAs are located in genomic regions distant from centromeres. Among them, *Palv148* in *P. alba* var. *pyramidalis* and *Salix180* in the two willow species are the most prevalent satellite repeats, while they are relatively rare in *P. euphratica* (Fig. 4a; Additional file 1: Figs. S7-S14 and S17-S18; Additional file 2: Table S9). In total, we identified 182,323 and 248,934 *Palv148* copies across 16 and 15 chromosomes of haplotype I and II of *P. alba* var. *pyramidalis*, respectively, with a total length of 29.34 and 38.99 Mb and accounting for 73.68 and 71.00% of the TRAs (Fig. 4b,c; Additional file 2: Table S9). Interestingly, comparative analysis across species found a satellite repeat like *Palv148*, namely *Sar145*, which is the component unit of the centromeric TRA on chr13 of *S. arbutifolia* (Additional file 1: Fig. S20f; Additional file 2: Table S10). However, similarity and phylogenetic analysis revealed clear differentiation between the two repeats, suggesting that they evolved independently in their respective species (Fig. 4d,e). In addition, a total of 41,696 (8.13 Mb in length) and 29,786 (5.59 Mb) *Salix180* copies were identified across 9 chromosomes of haplotypes I and II of *S. chaenomeloides*, respectively, accounting for 91.01 and 93.09% of the total length of all TRAs (Additional file 1: Figs. S11-S12, S18a and S21a-b; Additional file 2: Table S9). Similarly, we identified 9,243 (2.00 Mb) and 21,383 (4.36 Mb) *Salix180* copies across 12 chromosomes of haplotype I and haplotype II of *S. arbutifolia*, respectively, representing 25.85 and 42.46% of the total TRA length (Additional file 1: Figs. S13-S14, S18b and S21c-d; Additional file 2: Table S9). Further similarity and phylogenetic analysis showed that different *Salix180* monomers expanded independently in the two willow species but maintained extremely high sequence identity between them (Additional file 1: Figs. S21e-f).

Although these non-centromeric TRAs did not detect CENH3 enrichment peaks, they exhibited similar signatures to centromeres. For example, satellite repeats in these TRAs organized into HORs which showed a homogenization structure as the *Arabidopsis* centromeres (Fig. 4f-h; Additional file 1: Figs. S21g-k). These non-centromeric TRAs also displayed significantly fewer genes, lower histone modification levels, and higher DNA methylation levels compared to adjacent regions (Fig. 4a; Additional file 1: Figs. S7-S8 and S11-S14). Moreover, both centromeric and non-centromeric TRAs exhibit significant diversity in GC content (Additional file 1: Fig. S22). Interestingly, most of these *Salix180* non-centromeric TRAs are located on the long arms of chromosomes (Additional file 1: Fig. S18). The exception is that there are two TRAs composed of *Salix180* on four (chr07, chr13, chr17, and chr19) and eight (chr04, chr07, chr11, chr12, chr13, chr17, chr18, and chr19) chromosomes of *S. chaenomeloides* and *S. arbutifolia*, respectively, and most of them are located at both distal ends of the chromosomes in *S. arbutifolia* (Additional file 1: Figs. S11-S14 and S18; Additional file 2: Table S9). In addition, these TRAs showed higher Hi-C interaction signals with centromeres compared with other genomic regions, suggesting a potential interaction between them (Fig. 4i; Additional file 1: Figs. S21l and S23). However, monomers of these non-centromeric TRAs have higher sequence identity than centromeric TRAs (Figs. 3f and 4e; Additional file 1: Fig. S21e), and phylogenetic analysis revealed a mixed relationship of these repeat sequences from different chromosomes and haplotypes (Fig. 4d; Additional file 1: Fig. S21f). Since *Salix180* TRAs are shared between two willow species that diverged 22 million years ago (Mya) [36], and *Palv148* TRAs have recently been also found in other aspens and white poplars [47], these results consistently indicate that the origin of these non-centromeric TRAs is relatively ancient, and distinct monomers have experienced a recent rapid expansion among species.

### Salicaceae centromeres are extensively invaded by various transposable elements

**Fig. 5:**
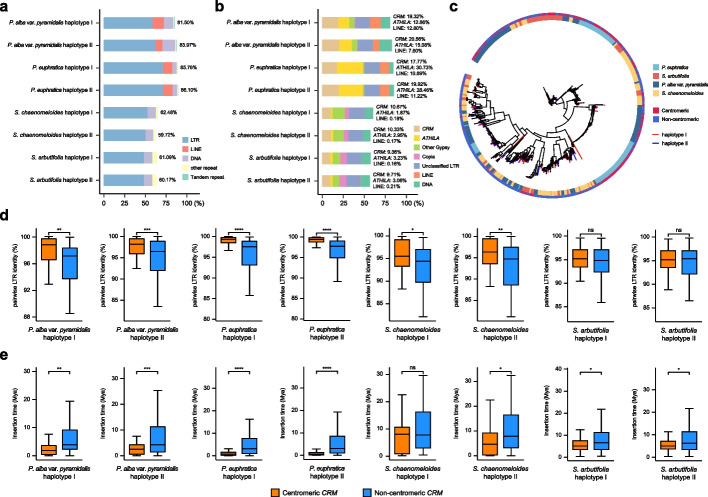
Analysis of *CRM* elements in Salicaceae genomes. **a** Boxplot of repetitive sequence statistics within centromeres, and the number of each boxplot represents the percentage of repetitive sequences. **b** Boxplot of *CRM*, *ATHILA*, LINE, and other repetitive sequence statistics within centromeres. **c** Phylogenetic tree of intact *CRM* elements. Colors of the outer and inner circle and the tree branch represent the corresponding *CRM* position, species, and genome haplotype, respectively. The distance scale is 0.1. **d** Boxplots of LTR identity comparisons between centromeric and non-centromeric *CRM* elements. Asterisks represent significant differences (two-tailed Wilcoxon rank-sum test, ****P ≤ 0.0001, ***P ≤ 0.001, **P ≤ 0.01, *P ≤ 0.05, ns: not significant). **e** Same as **d** but showing comparisons of LTR insertion time

Due to the limited TRAs in the centromeres of Salicaceae species, we next analyzed their sequence composition. Genome annotation showed that about 59.72 to 86.10% of the sequences in the functional centromeres are composed of various TEs, which could be supported by HiFi reads (Fig. 5a; Additional file 2: Tables S11-S15). Compared with other genomic regions, the centromeres were significantly enriched in Gypsy-like LTR retrotransposons, such as *CRM* and *ATHILA* elements in all species. Notably, *CRM* elements account for 9.36 to 23.78% of these centromeres (Fig. 5b). A phylogenetic tree was constructed using the intact *CRM* elements identified across the whole genomes, and the results showed that they clustered into two major clades, one of which was primarily composed of centromeric *CRM* elements and clustered according to species, while the other clade was non-centromeric elements and intermingled across species (Fig. 5c). This observation was also supported by higher sequence similarity between centromeric *CRM* elements than the other *CRM* elements (Fig. 5d). Further analysis showed that the median insertion times of these centromeric *CRM* elements in *P. euphratica* and *P. alba* var. *pyramidalis* were 0.75 and 1.94 Mya, later than the 4.76 and 5.07 Mya in *S. chaenomeloides* and *S. arbutifolia* respectively (Fig. 5e). These results indicated that Salicaceae species shared a common origin of centromeric *CRM* elements but undergo persistent species-specific amplification after their diversification. Consistently, we found that *ATHILA* elements, with the average length of 11.23 kb, were extensively expanded and significantly enriched in the centromeric and pericentromeric regions of the *P. euphratica* genome, resulting in a genome size that was nearly 90 Mb larger than that of *P. alba* var. *pyramidalis*, and a distinct organization from other species that exhibits an “accordion structure” with extremely high sequence similarity around the centromeres (Additional file 1: Figs. S9-S10 and S24; Additional file 2: Tables S16-S17). In contrast to *CRM* elements, centromeric *ATHILA* elements showed lower CENH3 enrichment, even lower than centromeric TRAs, and the insertion time of centromeric and non-centromeric *ATHILA* elements was similar across all genomes (Additional file 1: Figs. S25-S26 and S27a). Notably, *P. euphratica* exhibited a much later insertion time compared to the other species, indicating a more recent expansion of *ATHILA* elements (Additional file 1: Fig. S27b).

More specifically, we found that LINE elements were also significantly enriched in the centromeres of the two poplar species, with a proportion ranging from 7.85 to 12.62% (Fig. 5b). Interestingly, almost all the intact LINE elements (208 out of 247 in *P. alba* var. *pyramidalis* and 648 out of 676 in *P. euphratica*) were concentrated in the centromeric regions of the poplar genomes, while there were no intact LINE elements present in the entire willow genomes (Additional file 2: Tables S16-S19). Further analysis indicated that the previously reported LINE element “L1-1_PTr” [42] was shared but undergone independent expansions in the two poplar species within the last 5 million years, whereas a novel LINE element “TE_00000009” was specifically amplified in *P. euphratica* (Additional file 1: Figs. S28a-b, S29 and S30). Importantly, we found that these centromeric LINE elements, as well as centromeric *CRM* elements, showed significantly higher CENH3 enrichment than TEs outside centromeres (Additional file 1: Figs. S28c-d). Taken together, our findings indicated that LTR and LINE elements, rather than satellite tandem repeats, are the primary components of Salicaceae centromeres, serving as targeting sites for CENH3 (Additional file 2: Table S20).

### Frequent centromere turnover and repositioning in Salicaceae

Gene synteny analysis across these genomes revealed that most of the centromere positions were relatively conserved among species but were accompanied by the massive insertion of various repeat sequences and chromosomal inversions. As mentioned above, the species-specific insertion of TRAs and TEs around the centromere leads to considerable variations in their centromeric composition and size, with the *ATHILA* elements in *P. euphratica* being the most typical (Additional file 1: Figs. S24, S31 and S32). Moreover, multiple inversions were observed within and around the homologous centromeres, such as chr02, chr04, chr06, chr10, chr13, and chr19 between *P. euphratica* and *P. alba* var. *pyramidalis*, and chr03, chr07, chr08, chr12, chr15, chr18, and chr19 between *P. alba* var. *pyramidalis* and *S. chaenomeloides* (Additional file 1: Figs. S31-S32). Most importantly, our results showed that these events also frequently occur between haplotypes of the same species, which were further supported by both HiFi and Hi-C data (Additional file 1: Figs. S33-S42). These results suggest that repeat invasions and chromosomal inversions significantly and persistently contribute to the dynamic evolution of centromeres in Salicaceae.

**Fig. 6:**
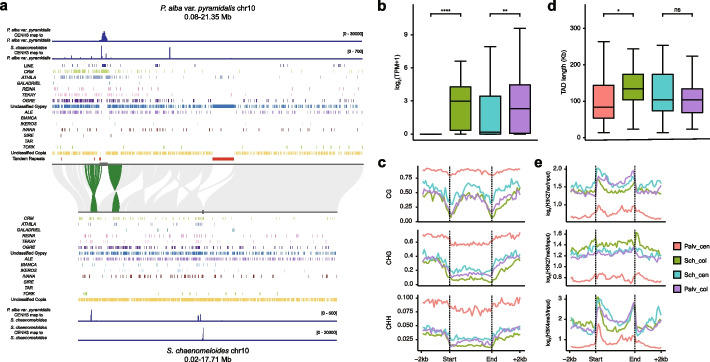
Analysis of centromere reposition between *P. alba* var. *pyramidalis* and *S. chaenomeloides*.** a** Synteny analysis of homologous chromosomes between *P. alba* var. *pyramidalis* and *S. chaenomeloides*. The two straight lines in the middle represent homologous chromosomes scaled by chromosome length and dark grey boxes on lines represent corresponding centromeres. Grey lines connecting chromosomes represent homologous genes pairs between *P. alba* var. *pyramidalis* and *S. chaenomeloides*, and green lines represent homologous genes located within centromeres and peri-centromeric regions of *P. alba* var. *pyramidalis*. Histograms represent the distribution of each type of LTRs and LINE elements from *P. alba* var. *pyramidalis* and *S. chaenomeloides*. CUT&Tag data coverage from both *S. chaenomeloides* and *P. alba* var. *pyramidalis* to the *P. alba* var. p*yramidalis* genome are shown at the top. CUT&Tag data coverage from both *S. chaenomeloides* and *P. alba* var. *pyramidalis* to the *S. chaenomeloides* genome are shown at the bottom. **b** Boxplot of expression comparison between genes within repositioned centromeres in *P. alba* var. p*yramidalis* and genes within homologous regions in *S. chaenomeloides*, and vice versa. Asterisks represent significant differences (two-tailed Wilcoxon rank-sum test, ****P ≤ 0.0001, ***P ≤ 0.001, **P ≤ 0.01, *P ≤ 0.05, ns: not significant). **c** Metaprofiles of methylation levels for genes within repositioned centromeres and homologous regions. **d** Same as **b** but showing TAD length comparisons. **e** Same as **c** but showing metaprofiles of histone modification levels. Palv_cen: genes within repositioned centromeres in *P. alba* var. p*yramidalis*. Sch_col: genes within homolougs regions of Palv_cen in *S. chaenomeloides*. Sch_cen: genes within repositioned centromeres in *S. chaenomeloides*. Palv_col: genes within homologous regions of Sch_cen in *P. alba* var. p*yramidalis*

Besides, we also discovered some repositioning events between *S. chaenomeloides* and the two poplar species that resulted in the relocation of the centromere to another position on the homologous chromosomes, including chr02, chr04, chr09, chr10, chr11, chr13, chr14, and chr16 (Fig. 6a; Additional file 1: Fig. S32). Although some inversions were observed in their adjacent regions, it is clear that these centromeres are located in different syntenic regions, suggesting that these repositioning events are not solely caused by chromosomal inversions. For example, the centromere of chr10 in *S. chaenomeloides* is positioned at the central part of the chromosome based on CENH3 enrichment, with arm ratios (length of the long arm/length of the short arm) of 1.08 and 1.11 in its two haplotypes. In contrast, it is more biased toward one side of the chromosome in poplars, with arm ratios of 2.39 and 2.33 in *P. euphratica* and 4.98 and 5.44 in *P. alba* var. *pyramidalis* (Fig. 6a; Additional file 1: Fig. S24 and S32; Additional file 2: Table S6). We then remapped the CENH3 CUT&Tag reads enriched in the centromere of *S. chaenomeloides* to its homologous chromosome in *P. alba* var. *pyramidalis* and found that they were mainly aligned to the centromere and corresponding collinear regions and vice versa (Fig. 6a; Additional file 1: Fig. S32). In comparison, the CUT&Tag-seq data from the native species detected only one CENH3-enriched peak, corresponding to the centromeric region of that species (Additional file 1: Fig. S32). This suggests that the repositioning of the centromere is achieved by the insertion of some homologous sequences that constitute the ancient centromere into the new chromosomal positions.

Consistent with other plants, we found that centromeres contained fewer expressed genes, and their expression levels were significantly lower than those of non-centromeric genes (Additional file 1: Fig. S43; Additional file 2: Table S21). To further investigate the impact of centromere repositioning on gene expression, we examined expression switches between collinear genes within the repositioned centromeres in *P. alba* var. *pyramidalis* and *S. chaenomeloides*. The results showed that the expression of centromere genes in one species was significantly reduced compared with the corresponding non-centromere collinear genes in another species, and the DNA methylation level was significantly increased especially in CG context (Fig. 6b,c). Consistently, we found that *P. alba* var. *pyramidalis* exhibited higher DNA methylation, shorter TAD lengths, and lower histone modification levels than *S. chaenomeloides* in the repositioned centromeres (Fig. 6d,e). More importantly, we found that the genes in these repositioned centromeres in *S. chaenomeloides*, but not in *P. alba* var. *pyramidalis*, showed significantly higher expression levels, lower methylation levels, and histone modification levels compared with other genes in the conserved centromeres (Additional file 1: Fig. S44). These results suggest that the corresponding centromeres of *S. chaenomeloides* may have been recently repositioned, without sufficient time to establish the epigenetic landscape that represents the stable centromere. Nevertheless, these findings suggest that changes in gene expression mediated by epigenetic modifications are critical for the stability of centromeres.

## Discussion

Centromeres play essential roles in guiding the separation of chromosomes during cell division. In this study, we assembled near-complete and haplotype-resolved genomes for four Salicaceae species to investigate the sequence variation and evolutionary history of their centromeres. As is typical for most eukaryotes, centromeres in Salicaceae are often found in heterochromatin regions with relatively high levels of DNA methylation. However, our results demonstrate that the sequence compositions and structural features of their centromeres vary extensively among chromosomes from both the same and different species. All four species showed the presence of TRA-based and array-free centromeres, with *P. alba* var. *pyramidalis* having the majority of centromeres that contained TRA, whereas the other three species predominantly lacked TRA in their centromeres. Overall, only a small fraction of TRAs were located in centromeric regions, exhibiting significant length differences and sequence variations among chromosomes and haplotypes. Even among TRAs with the same satellite repeats, such as *Palv156* in *P. alba* var. *pyramidalis*, most monomer sequences are exclusive to each centromere and display higher intrachromosomal similarity than interchromosomal similarity. This means that the homogenization of satellite repeats in Salicaceae centromeres is predominantly a chromosome-independent process, as demonstrated by its occurrence not only between various chromosomes within and between species, but also on homologous chromosomes between haplotypes, resulting in the emergence of haplotype-specific centromeric TRAs in the same species, such as *Palv311* in haplotype I of *P. alba* var. *pyramidalis*. These results revealed a highly rapid turnover and complex landscape of centromeric sequences in Salicaceae.

Interestingly, we discovered that, with the exception of *P. euphratica*, the most prevalent TRAs in the genomes of these species were scattered at chromosomal locations distant from the centromere. The structural characteristics of these non-centromeric TRAs exhibit a significant level of homogenization. In contrast to centromeric TRAs, the satellite monomers of these non-centromeric TRAs do not diverge independently between chromosomes but rather exhibit interchromosomal mixtures in their phylogenetic relationships. This observation could be attributed to a recent origin, without sufficient time for differentiation. However, our comparative analysis found similar satellite monomers among species, pointing to an evolutionary trajectory in which they arose from a common ancestor and homogenized individually after speciation. These results indicate that non-centromeric TRAs may have a distinct homogenization mechanism compared to centromeric TRAs, enabling the recombination or exchange of satellite monomers across chromosomes. More importantly, these results support their involvement in certain biological function, making them favored by natural selection and retained during long-term evolution. For example, studies have revealed that the TRA-containing heterochromatic knobs in maize can be transformed into centromere-like bodies that are preferentially segregated through female meiosis [56, 57]. If so, the kinesins that bind these non-centromeric TRAs in Salicaceae and their coevolution with diverged TRAs among species warrant further investigation. Moreover, a recent study on hybrid poplar have hypothesized that they may play a role in driving reproductive isolation between poplar species [47], similar to the species-specific heterochromatic TRAs in *Drosophila*, which prevents mitotic chromosome segregation, leading to hybrid lethality [58]. Regardless, these hypotheses need to be further validated in the future.

Finally, our study showed that the most abundant sequences constituting Salicaceae centromeres are derived from various TEs, including *CRM* and *ATHILA* elements in all four species and LINE elements in two poplars, whose extensive expansion and invasion drive the diversity and dynamic evolution of their centromeres. Among these, *CRM* and *ATHILA* elements have been reported in the centromeres of many plants, such as maize, wheat, cotton, and *Arabidopsis* [12, 26, 52–54, 59], which invaded the satellite array and made significant contributions to centromere diversity. In comparison, there are fewer reports on the enrichment of LINE elements in plant centromeres [60–62]. Our findings revealed that nearly all LINE elements in the poplar genomes are concentrated in their centromeric regions. Their notable biased insertion mechanism requires further investigation to elucidate the underlying biological significance. A similar centromeric architecture has been previously identified in the yellowhorn (*Xanthoceras sorbifolium*) genome, where centromeric regions are predominantly composed of newly inserted Gypsy and LINE1 elements rather than TRAs [60]. These collective findings provide compelling evidence for the highly dynamic nature of centromeric sequences across plant species. The observed compositional variations in TEs and TRAs among different species may reflect ongoing evolutionary processes involving competitive interactions and purging events between these sequence elements. However, this hypothesis requires further empirical validation through comprehensive comparative genomic studies. In addition, all of these centromeric TEs showed species-specific expansion patterns similar to those of centromeric TRAs. The pervasive insertion of TEs has disrupted collinearity between haplotypes in centromeric regions, mediated chromosomal inversions, and facilitated centromere repositioning across species. These differential TE insertions have further contributed to the divergence of gene expression and epigenetic landscapes among orthologous genes, underscoring their role in shaping the structural and functional evolution of Salicaceae centromeres. A striking example of TE-driven genomic evolution has been observed in *P. euphratica*, where the proliferation of *ATHILA* elements around centromeric regions has led to significant genome expansion and the establishment of a distinct genomic architecture compared to other species. This unique organization raises compelling questions regarding the functional consequences of *ATHILA* proliferation, particularly its potential role in conferring the species-specific adaptive traits of *P. euphratica*, such as its exceptional salt tolerance and reproductive isolation from other poplars. Future studies are needed to elucidate whether these centromeric *ATHILA* insertions contribute to the adaptive evolution and ecological divergence of *P. euphratica*, providing insights into the mechanisms underlying TE-mediated genomic diversification.

## Conclusions

In conclusion, our results highlight a considerable degree of sequence and structural diversity in the centromeres of Salicaceae species, implying species-specific and even chromosome-specific evolutionary trajectories, and ongoing centromere turnover which was mediated by satellite homogenization, widespread invasions of multiple TEs and repositioning events. These findings provide new insights into the highly complex and dynamic evolution of centromeres among closely related species.

## Methods

### Plant materials, HiFi and Hi-C library construction

All materials of *P. alba* var. *pyramidalis*, *P. euphratica*, *S. chaenomeloides*, and *S. arbutifolia* used in this study were collected in Lanzhou (Gansu), Lanzhou (Gansu), Hanzhong (Shanxi), and Baishan (Jilin) of China, respectively. For each species, high molecular weight DNA from fresh leaves was isolated using a standard CTAB protocol in which nuclei were isolated to remove the plastid and mitochondrial genomes. HiFi libraries were subsequently prepared according to the PacBio official manual. Size-selected libraries were sequenced on the PacBio Sequel II system, and the sequenced subreads were used to generate HiFi reads using ccs v6.0.0 (https://github.com/PacificBiosciences/ccs) with parameters “--min-passes=3 --min-rq=0.99.” Hi-C libraries for each species were constructed using the procedures described previously [36, 49, 50]. The resulting library was sequenced on an Illumina HiSeq X Ten platform to generate 150-bp paired end reads.

### De novo assembly and Hi-C scaffolding

The genome size and heterozygosity of four Salicaceae species were estimated using GenomeScope2 [63]. For each species, PacBio HiFi reads were assembled using hifiasm v0.16.1 [64] with the “Hi-C integration” assembly strategy to generate haplotype-resolved contigs. We subsequently integrated Hi-C data to generate pseudochromosomes for each haplotypes using Juicer v1.6 [65] and the 3D-DNA pipeline v180922 [66]. In brief, Hi-C Illumina paired end reads were processed by Juicer with default parameters. The output file “merged_nodups.txt” and contigs were subsequently used to produce the Hi-C contact matrix using 3D-DNA with the parameter “-r 0,” which was further visualized using the Juicebox software v1.11.08 [67] to anchor contigs into pseudochromosomes. The final assemblies were oriented and renamed based on the chromosome synteny of published reference genomes [37, 49, 50].

### Genome evaluation

To evaluate the genome quality, we firstly mapped PacBio HiFi reads to the final genome assembly using minimap2 v2.24 [68] with parameters “--secondary=no -t 20 -ax map-hifi” where both the mapping rate and the coverage depth were calculated. Assembly completeness evaluation was also performed using BUSCO v5.0.0 [69] with the embryophyta_odb10 dataset. Moreover, the 5S and 45S ribosomal DNA distribution of each genome was identified by performing the BLASTN v2.7.1+ [70] search using respective published *P. trichocarpa* ribosomal RNA gene sequences with the GenBank website (https://www.ncbi.nlm.nih.gov/genbank) IDs as “XR_002979605.1” and “MT796556.1,” and only alignments with coverage over 90% were retained.

### Repetitive sequence annotation

We performed both de novo prediction and homology search for TE annotation. We firstly combined sequences of all genomes and applied the Extensive de novo TE Annotator (EDTA) pipeline v2.1.0 [71] with parameters “--overwrite 1 --sensitive 1 --anno 1 --evaluate 1” to generate the non-redundant predicted TE library, and *P. trichocarpa* TEs in the Repbase database v29.01 [72] was used as the curated library. The whole-genome TE sequences were subsequently masked using RepeatMasker v4.1.2 (http://www.repeatmasker.org). For telomere annotation, we performed the Tandem Repeat Finder (TRF) software v4.09 [73] with parameters “2 7 7 80 10 50 2000,” and telomere regions were identified by searching the seven-base plant telomere repeat sequence arrays “CCCTAAA/TTTAGGG” at the two distal ends of each chromosome.

### Full-length long terminal repeats (LTRs) annotation and analysis

To detect the insertion time of transposable elements within centromeres and adjacent regions, full-length LTRs were firstly identified using LTRharvest and LTRdigest from the genometools software v1.5.10 [74], and intact LTRs annotated by the EDTA pipeline were also combined. All full-length LTRs were classified based on domain homology search with the REXdb database (http://repeatexplorer.org) using the dante.py script v0.1.3 (https://github.com/kavonrtep/dante). For analyzing LTRs, we extracted the 5′ and 3′ repeats of each LTR and performed alignments using MUSCLE v3.8.1551 [75] with default parameters. The LTR identity was defined as the percentage of identical nucleic acid pairs in the alignment result. For the insertion time of LTRs, we firstly calculated the divergence values of the 5′ and 3′ repeats using the dist.dna function in the ape R package v5.7-1 (https://cran.r-project.org/web/packages/ape). The insertion time of LTRs were finally estimated by the formula “T = K/(2r)” where the K and r represented the divergence value and the neutral mutation rate of 2.5 × 10^−9^ per year [42], respectively. The significance of the differences in identity and insertion time between centromeric and non-centromeric LTRs was estimated using the Wilcoxon rank-sum test for nonnormal distributions in R.

### Gene prediction

The protein-coding genes of all the Salicaceae genomes were predicted by three methods, including transcriptome-based annotation, homology-based annotation, and ab initio prediction. For transcriptome-based annotation, the RNA-seq Illumina paired end reads were firstly mapped to the repeat-masked genome using Hisat2 v2.2.1 [76] with the parameter “-dtx,” and genes were annotated based on the mapping result using TransDecoder v5.5.0 (https://github.com/TransDecoder/TransDecoder) with parameters “LongOrfs: -m 100 -G universal; Predict: --retain_long_orfs_mode dynamic.” For homology-based annotation, protein sequences from *A. thaliana* and 15 published Salicaceae genomes, including *Casearia decandra*, *Idesia polycarpa*, *Itoa orientalis*, *P. trichocarpa*, *P. alba* var. *pyramidalis*, *P. alba L.*, *P. euphratica*, *P. qiongdaoensis*, *P. tremula L.*, *P. tremuloides*, *S. brachista*, *S. chaenomeloides*, *S. purpurea*, *S. rehderiana* and *S. suchowensis* [34, 36, 39, 42, 44, 49, 50, 77, 78], were integrated and used them as the homologous evidence for genes prediction using GeneWise v2.4.1 [79] with parameters “--coverage_ratio 0.4 --evalue 1e-9.” Both the transcriptome and homology-annotation results were combined to obtain the training model for ab initio prediction using Augustus v3.2.3 [80] with parameters “--gff3=on --allow_hinted_splicesites=gcag,atac --alternatives-from-evidence=true --min_intron_len=30 --softmasking=1.” Finally, all the three results were merged using the GETA pipeline (https://github.com/daizao/geta) to generate high-quality gene models which were further validated by the Pfam v3.0 [81] database. The completeness of the final protein dataset was evaluated using BUSCO v5.0.0 under the protein mode with the embryophyta_odb10 dataset. Biological functions of genes were annotated using BLASTP v2.7.1+ against the Non-Redundant Protein Sequence Database (NR) v20190123 and the UniProt v20190725 sequences in the RefSeq database [82] with the parameter “-evalue 1e-5.” Gene Ontology (GO) was annotated by InterproScan v5.44-79.0 [83]. Kyoto Encyclopedia of Genes and Genomes (KEGG) pathways and Clusters of orthologous groups for eukaryotic complete genomes (COG) were annotated by eggNOG-mapper v5.0 [84]. For the annotation of non-coding RNAs, MicroRNA, and snRNA genes were annotated by INFERNAL v1.1.4 [85] against the Rfam v14.10 database with parameters “--cut_ga --nohmmonly --rfam --noali,” and tRNA genes were detected by tRNAscan-SE v2.0 [86] with the parameter “-G.”

### Allele gene identification and Circos

For each species, a reciprocal BLASTP was firstly performed to align proteins of the two haplotype gene sets with parameters “-evalue 1e-5 -perc_identity 60 -qcov_hsp_perc 60.” The alignment result and gene positions were subsequently subjected to the MCScanX [87] software to identify syntenic blocks with parameters “-a -e 1e-5 -u 1 -s 5.” Finally, paired genes within synteny blocks were manually checked and screened as alleles. Circos plots were generated using the TBtools v2.012 [88] software where the density of genes and repetitive elements as well as the CUT&Tag CENH3/Input ratio shown in the circos plot were calculated in non-overlapping 10-kb windows.

### Haplotype sequence collinearity analysis

For sequence collinearity between haplotype chromosome pairs, we applied the similar method for the published enikorn genomes [89]. Briefly, one chromosome was fragmented in non-overlapped 1-kb sequence segments, which were sequentially aligned to the homologous chromosome using BLASTN with parameters “-evalue 1e-5 -perc_identity 60 -qcov_hsp_perc 60.” The positions of the top five BLASTN hits for each segment were retained and used for the dot plot alignments by a homemade R script.

### Homologous gene pairs identification

We performed the Whole-Genome Duplication Integrated analysis (WGDI) v0.6.5 [90] tool to search homologous genes between species. Briefly, synteny blocks between the two genomes were firstly identified by MCScanX, and the synonymous substitution rate (Ks) of each gene pair in synteny blocks were subsequently calculated using PAML-yn00 with the YN method. For genes in one genome aligned to more than one position in the other genome, we manually selected one gene pair based on the two standards: (1) this gene pair had a lower Ks value; (2) the aligned gene was clustered with adjacent gene pairs on the chromosome. The final gene pairs were defined as homologous genes and visualized by the graphics.synteny script in JCVI (https://github.com/tanghaibao/jcvi).

### Centromere-specific histone H3 variant (CENH3) gene identification

The CENH3 gene of each genome was identified using BLASTP with the parameter “-evalue 1e-5 -qcov_hsp_perc 50” and the *A. thaliana* CENH3 protein HTR12 (AT1G01370) [91] was used as the reference. For searching CENH3 fragments in *Populus* Chr2, the coding sequence of each CENH3 protein was aligned to the corresponding genome using BLASTN with parameters “-evalue 1e-5 -word_size 4.” The neighborhood-joining phylogenetic tree of CENH3 genes was constructed and visualized using MEGA v11.0.13 [92] and FigTree v1.4.4 (https://github.com/rambaut/figtree), respectively.

### CUT&Tag library preparation and data analysis

The anti-CENH3-1 and anti-CENH3-2 for constructing CUT&Tag libraries were generated using rabbit polyclonal anti-CENH3 against peptide sequences “MPKRSDASPSTPRTPTSSRTRPQANDQQGSSTQR” from the Palv1_13083 gene and “MSRRQSGASPSTPQSPPLTPTSLRTPGQSKRSLATA” from the Sch1_11595 gene, respectively. The anti-H3K27ac was purchased from Abcam (https://www.abcam.cn) and both anti-H3K4me3 and anti-H3K27me3 were purchased from Sigma-Aldrich (https://www.sigmaaldrich.cn). About 10 g of fresh leaves were collected and used for nuclei extraction. Extracted nuclei were digested with micrococcal nuclease (Sigma, N3755), and subsequently used for CUT&Tag antibodies. About 5 µl antibodies, with a concentration of 0.83 mg/ml, were used for per 25 µg chromatin. Two biological replicates were set. The CUT&Tag libraries were sequenced to generate 150-bp paired end reads on the Illumina HiSeq platform (Illumina). Resulting CUT&Tag data of each histone modification was used for downstream analysis.

For centromere identification, we applied the similar method for the published maize genome [22]. Raw paired end reads of CUT&Tag and Input were firstly processed with fastp v0.21.0 [93] to remove low-quality bases and adapter sequences. Filtered reads were mapped to reference genome assembly using Bowtie2 v2.3.2 [94] with parameters “--end-to-end --very-sensitive --no-mixed --no-discordant -I 10 -X 700.” The generated bam files were sorted based on chromosomal coordinates and potential polymerase chain reaction duplicates were further removed using SAMtools v1.9 [95]. The enrichment level of each histone modification was defined by the BPM ratio of CUT&Tag to Input calculated using bamCompare in the Deeptools package v3.5.1 [96] with the parameters of “--binSize 1 --operation ratio --outFileFormat bedgraph --normalizsUsing BPM --scaleFactorsMethod None.” For centromere identification, the average enrichment of CENH3/Input ratio of each chromosome was calculated using BEDtools v2.30.0 [97] with the window bin size of 500 bp. Bins with the distance interval shorter than 100 kb were merged. The final positions of centromeres were validated by visual inspection of the distribution of CENH3 CUT&Tag/Input ratio using IGV v2.16.0 [98]. Centromere positions were also predicted using MACS2 v2.2.9.1 (https://pypi.org/project/MACS2) with the parameters of “--call-summits -q 0.01 -f BAMPE -B” for further validations.

To further explore the targeting sites of CENH3 proteins, we performed the de novo prediction pipeline using the Web-based Galaxy RepeatExplorer software (https://repeatexplorer-elixir.cerit-sc.cz/galaxy) [99]. Firstly, we randomly selected and uploaded 10 million reads from the input control to the Galaxy RepeatExplorer website and performed graph-based clustering using RepeatExplorer. For each repeat cluster, we secondly uploaded 10 million reads from CENH3 CUT&Tag samples to website and calculated the CUT&Tag/Input ratio of the normalized read counts. Clusters with the CUT&Tag/input ratio higher than 2 were identified as CENH3 binding sites.

### Tandem repeat annotation and analysis

We applied the TRASH pipeline v1.2 [100] to search tandem repeat arrays (TRAs) across all the genomes, and TRAs with the monomer length longer than 50 bp and the repeat number more than 50 were retained for downstream analysis. The sequence similarity of centromeres and adjacent regions including TRAs and TEs were visualized by the StainedGlass package v0.5 (https://github.com/mrvollger/StainedGlass). Higher-order repeats (HORs) of each TRA were identified using HOR program in the TRASH pipeline with parameters “--horonly --minhor 2 --maxdiv 5.”

To analyze the diversity of TRAs, we calculated the “variant distance” of each satellite monomer referenced from the published Col-CEN *A. thaliana* genome [12]. Taking *Palv156* monomers in chr01 of *P. alba* var. *pyramidalis* haplotype I as example, we firstly aligned all the 4682 *Palv156* monomers using MAFFT v7.490 [101] with default parameters. For each position of the generated alignment file (212 positions), we calculated the proportion of different elements, including A, G, C, T, and gaps. We subsequently extracted each monomer from the alignment file and the variant distance of each position was calculated as “1 - corresponding element proportion.” Variant distances of all positions were finally cumulated to assess the variation across all monomers. For tracing evolutionary origins of TRAs, we selected the monomer with the highest copy number in each chromosome as representative monomers, i.e., twelve *Palv156* and thirty-one *Palv148* representative monomers in *P. alba* var. *pyramidalis*, twenty-six *Salix180* representative monomers in *S. chaenomeloides* and thirty-nine *Salix180* representative monomers in *S. arbutifolia*. We subsequently aligned them to the combined TE library using BLASTN with parameters “-evalue 1e-5 -word_size 4.”

To construct the phylogenetic relationship of TRAs, we randomly selected each type of TRA sequences in each chromosome, and the number of TRAs selected in each chromosome was proportional to the total number of TRAs in this chromosome. For example, we randomly selected *Palv156* TRA monomers from the six corresponded chromosomes of *P. alba* var. *pyramidalis* haplotype I, including 696 monomers from chr01, 1828 monomers from chr02, 10 monomers from chr03, 155 monomers from chr09, 89 monomers from chr10, and 66 monomers from chr18. The selected TRA monomers were mutually aligned using MAFFT with default parameters, and the generated alignment file was used to construct the maximum-likelihood phylogenetic tree by FastTreeMP v2.1.11 [102] with the parameter “-nt.” The final phylogenetic structure of TRAs was visualized using the Interactive Tree of Life (iTOL; https://itol.embl.de) [103] website.

### Whole genome bisulfite sequencing (WGBS) and data analysis

Genomic DNA was extracted from fresh leaves of *P. alba* var. *pyramidalis*, *P. euphratica* and *S. chaenomeloides* using the CTAB method. To estimate the bisulfite conversion rate, appropriate lambda DNA were added to the purified DNA before applying the bisulfite treatment. The mixed DNA sample was subsequently fragmented using Covaris S220 to a mean size of 200 ~ 300 bp, followed by the blunt ending, dA addition to 3′-end, and adaptor addition following the Illumina manufacturer’s protocol. Adaptor-added DNA was subjected to bisulfite conversion using the Zymo Research EZ DNA Methylation Gold Kit. Finally, the bisulfite-treated DNAs were PCR amplified for 16 cycles. The resultant DNAs were sequenced on the BGI DNBseq instrument and 100 bp paired end reads were generated for downstream analysis. The bisulfite sequencing data of *S. arbutifolia* was obtained from the published data from the National Genomics Data Center (NGDC; https://bigd.big.ac.cn/bioproject) with the accession number of SAMC394240.

Raw paired end reads of bisulfite sequencing were firstly processed with fastp to remove low-quality bases and adapter sequences. Filtered reads were mapped to reference genome assembly using BSMAP v1.1.3 [104] with parameters “-v 0.04 -r 0.” The generated bam files were sorted based on chromosomal coordinates and potential polymerase chain reaction duplicates were further removed using SAMtool. DNA methylation sites covered by < 4 reads were removed. Moreover, more than three consecutive CHH methylation sites were excluded to remove false positive results. Positional DNA methylation levels, including CG, CHG, and CHH methylation, were calculated using the methratio.py script in the BSMAP package.

### RNA-seq sequencing and data analysis

For transcriptome sequencing (RNA-seq), fresh leaves of *P. alba* var. *pyramidalis*, *P. euphratica*, and *S. chaenomeloides* were collected and three biological replicates were constructed for each species. Total RNA was extracted from the tissue samples using a rNeasy Plant Mini Kit (Qiagen), and cDNA libraries were constructed following the manufacturer’s instructions. The resulting libraries were finally sequenced on an Illumina HiSeq X Ten platform to generate 150 bp paired end reads for downstream analysis. The RNA-seq sequencing data of *S. arbutifolia* was obtained from the published data from the NGDC website with the accession number of SAMC394238.

Raw paired end RNA-seq reads were firstly processed with fastp to remove low-quality bases and adapter sequences. Filtered reads were mapped to reference genome assembly by Hisat2 with parameters “-dtx.” The output bam files were used to measure gene expression levels using Stringtie v2.1.4 [105] to generate the transcript per million (TPM) value. For comparing the gene expression level across species, we extracted the TPM value of homologous genes for each replicate within each species, which was subsequently combined to calculate trimmed mean of M values (TMM) as normalization factors for each individual replicates using the “edgeR” package v3.18.1 [106] in R. TPM values of individual replicates from each species were finally normalized using these normalization factors.

### Hi-C data analysis

For the analysis of Hi-C data, raw paired end Hi-C reads were firstly processed with fastp to remove low-quality bases and adapter sequences. Filtered reads were subsequently mapped to reference genome assembly by Juicer. PCR-duplicated reads, multiple-mapped reads (> 3), single-end mapped reads, dangling-end reads, and reads with low mapping quality (MAPQ < 30) were filtered out for the downstream analysis. The remained valid pairs were used to create the contact matrix which was subsequently self-corrected using HiC-Pro software [107] with the Iterative correction and eigenvector decomposition (ICE) method. Topologically associating domains (TADs) were identified using cworld-dekker (https://github.com/dekkerlab/cworld-dekker) with the Hi-C resolution of 10 kb.

### Immunofluorescence assay and FISH

Immunofluorescence assay was performed according to previously reported protocols using the anti-CENH3-1 antibody in *P. euphratica* and anti-CENH3-2 antibody in *S. chaenomeloides* [108]. For the immunofluorescence combined with FISH assay of TE_00000009 in *P. euphratica*, the cytological preparations were performed and followed with a sequential FISH procedure as previously reported [45], and DNA probes of TE_00000009 were amplified using specific primers 5′-GCCTAGGGTTCTGAACG-3′ and 5′-CATGTATTAGCACAAGATGT-3′.

## Supplementary Information

Additional file 1.

Additional file 2.

## Data Availability

All sequence data, genome assembly, and annotation information of *P. alba* var. *p**yramidalis*, *P. euphratica*, *S. chaenomeloides* and *S. arbutifolia* used in this manuscript have been deposited in the National Genomics Data Center (NGDC; https://bigd.big.ac.cn/bioproject) under BioProject accession number PRJCA029103 [109]. Sequencing data and genome assemblies generated in this study can be found under the "GSA" and "GWH" heading within the same BioProject number, respectively. Publicly available RNA-seq and bisulfite sequencing data for *S. arbutifolia* can be found under NGDC BioProject number PRJCA005435 [110, 111]. Figures, Supplementary figures in Additional file1 and Supplementary tables in Additional file2 are also available at figshare (https://figshare.com/) with DOI numbers of 10.6084/m9.figshare.28767854, 10.6084/m9.figshare.28767809 and 10.6084/m9.figshare.28767881, respectively.
