# Supplementary material for: Four near-complete genome assemblies reveal the landscape and evolution of centromeres in Salicaceae
Source: Genome Biol. 2025 May 2;26:111. doi: 10.1186/s13059-025-03578-7 (PMC12046899; doi:10.1186/s13059-025-03578-7)
Supplement: Supplementary file 1 — Additional file 1. [file 13059_2025_3578_MOESM1_ESM.pdf]

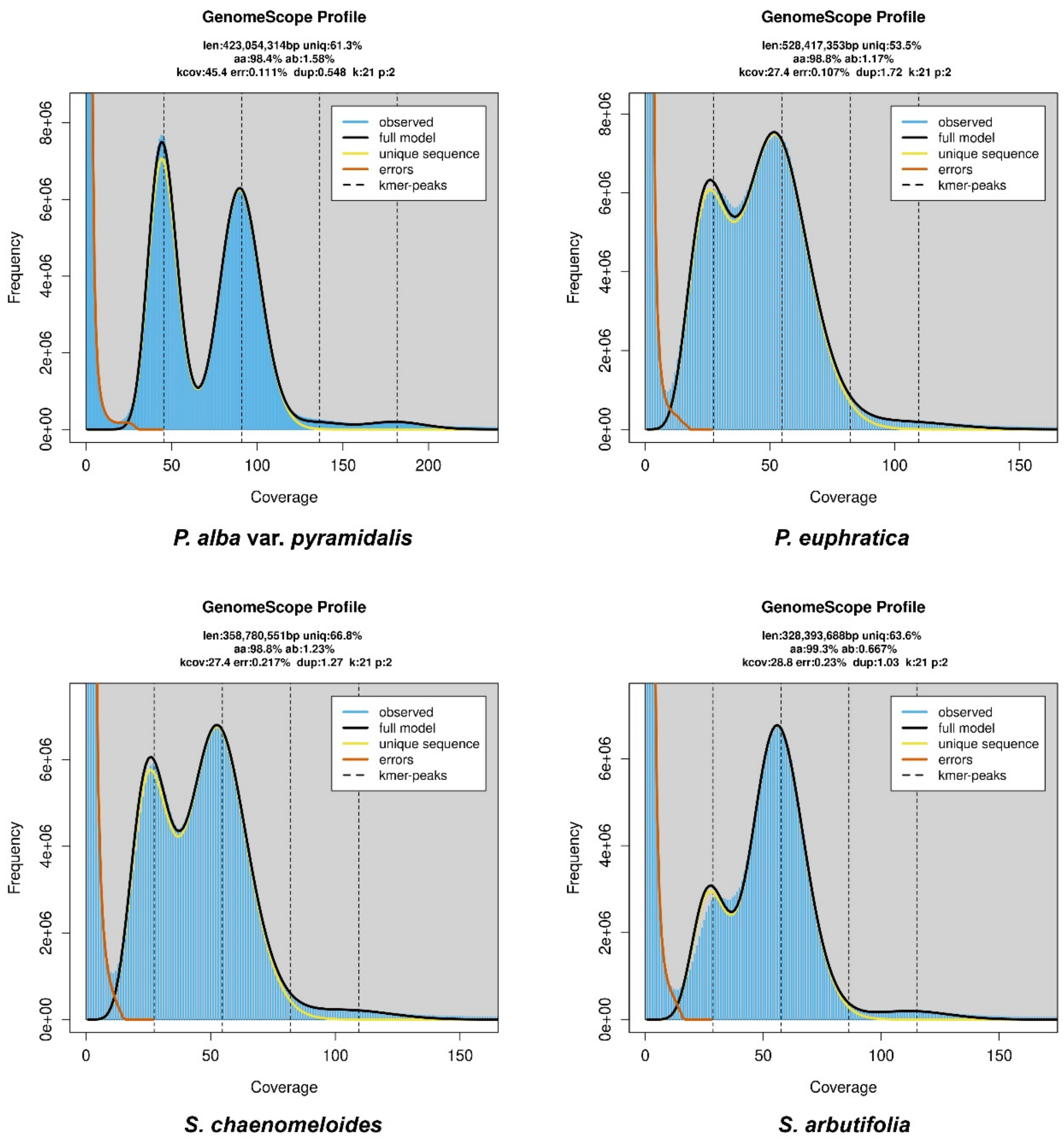

Fig. S1. Genome size and heterozygosity estimation of four Salicaceae species.

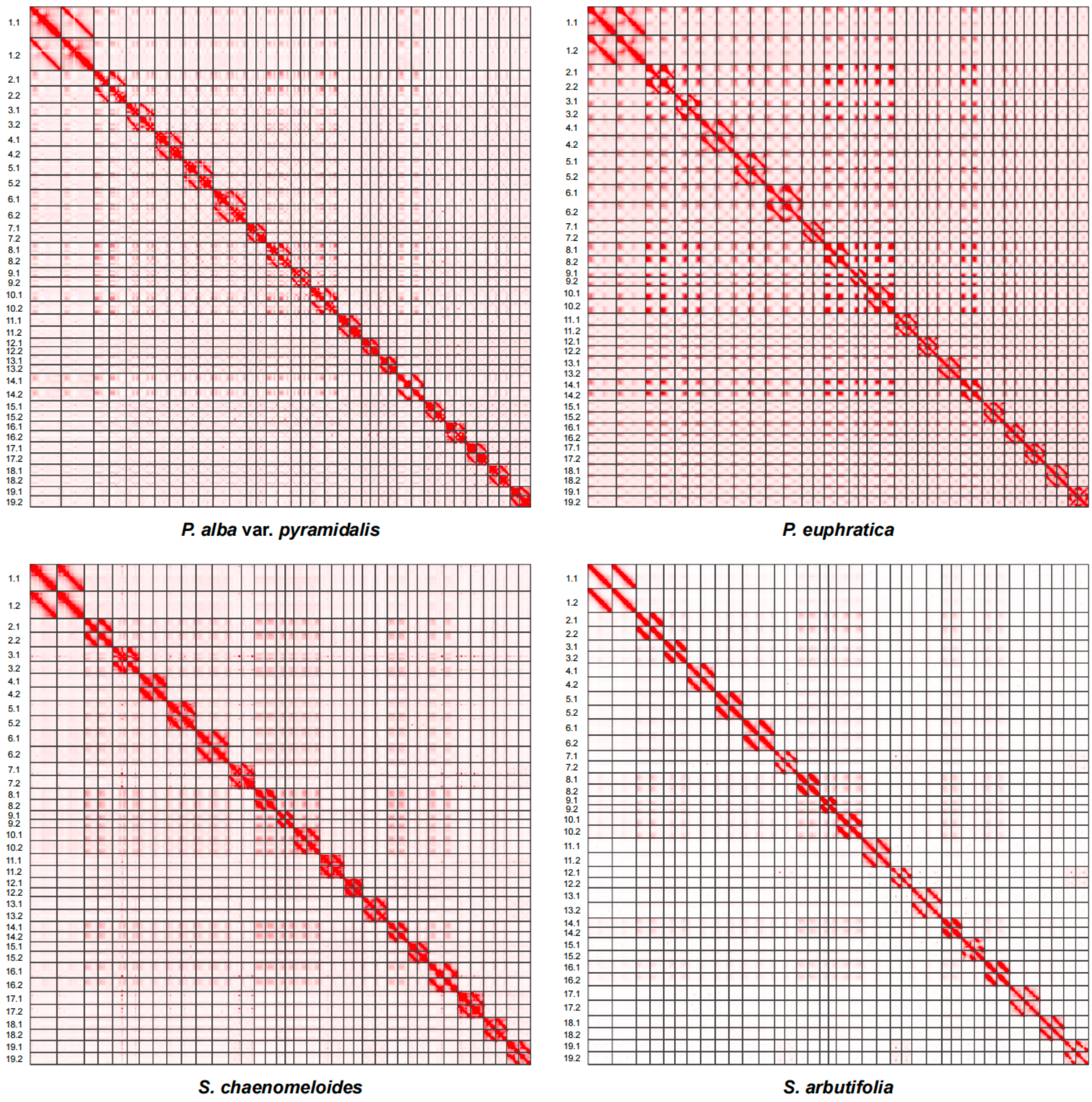

Fig. S2. Hi-C contact maps for assembled haplotype-phased genomes.

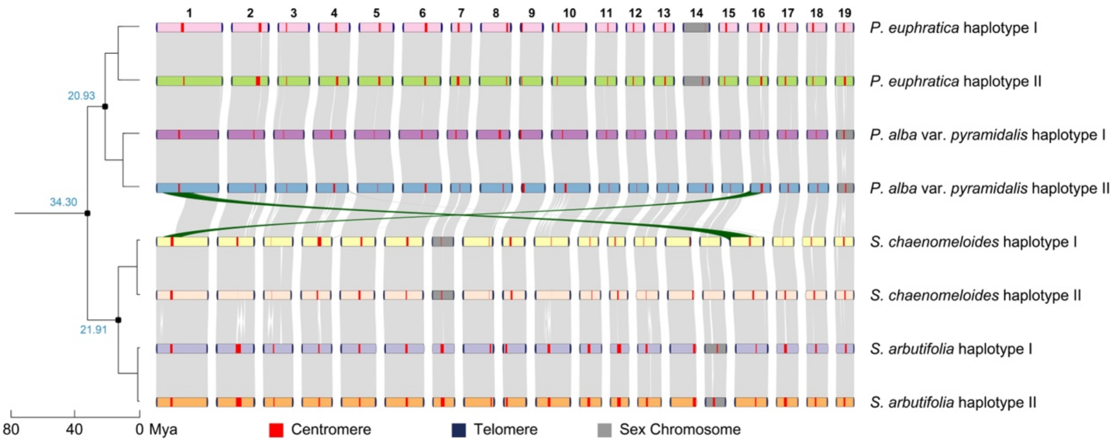

Fig. S3. Synteny plot illustrating collinearity of the assembled Salicaceae genomes. Grey alignment tracks represent homologous genes across genomes. Green and dark blue alignment tracks represent recombination of chr01 and chr16 between poplars and willows. The phylogenetic relationship of assembled genomes is shown on the left.

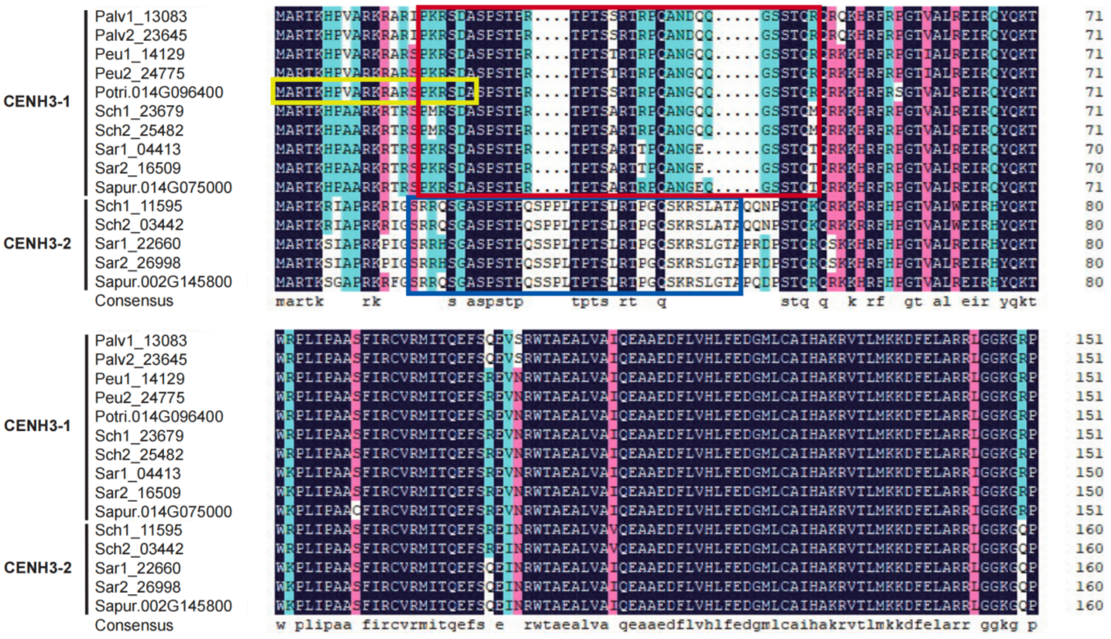

Fig. S4. Amino acid alignment of CENH3-1 and CENH3-2 proteins. Protein sequences of Palv1_13083 from *P. alba* var. *pyramidalis* haplotype I, Palv2_23645 from *P. alba* var. *pyramidalis* haplotype II, Peu1_14129 from *P. euphratica* haplotype I, Peu2_24775 from *P. euphratica* haplotype II, Sch1_11595 and Sch1_23679 from *S. chaenomeloides* haplotype I, Sch2_25482 and Sch2_03442 from *S. chaenomeloides* haplotype II, Sar1_04413 and Sar1_22660 from *S. arbutifolia* haplotype I, Sar2_16509 and Sar2_26998 from *S. arbutifolia* haplotype II, Potri.014G096400 from *P. trichocarpa*, Sapur.014G075000 and Sapur.002G145800 from *S. purpurea* were used for alignments. Peptide sequences used to generate anti-CENH3-1 and anti-CENH3-2 are highlighted by open red and blue rectangles, respectively. The published anti-CENH3 peptide sequence (Xin et al. 2024, Plant Physiology, doi.org/10.1093/plphys/kiae214) is highlighted by the open yellow rectangle.

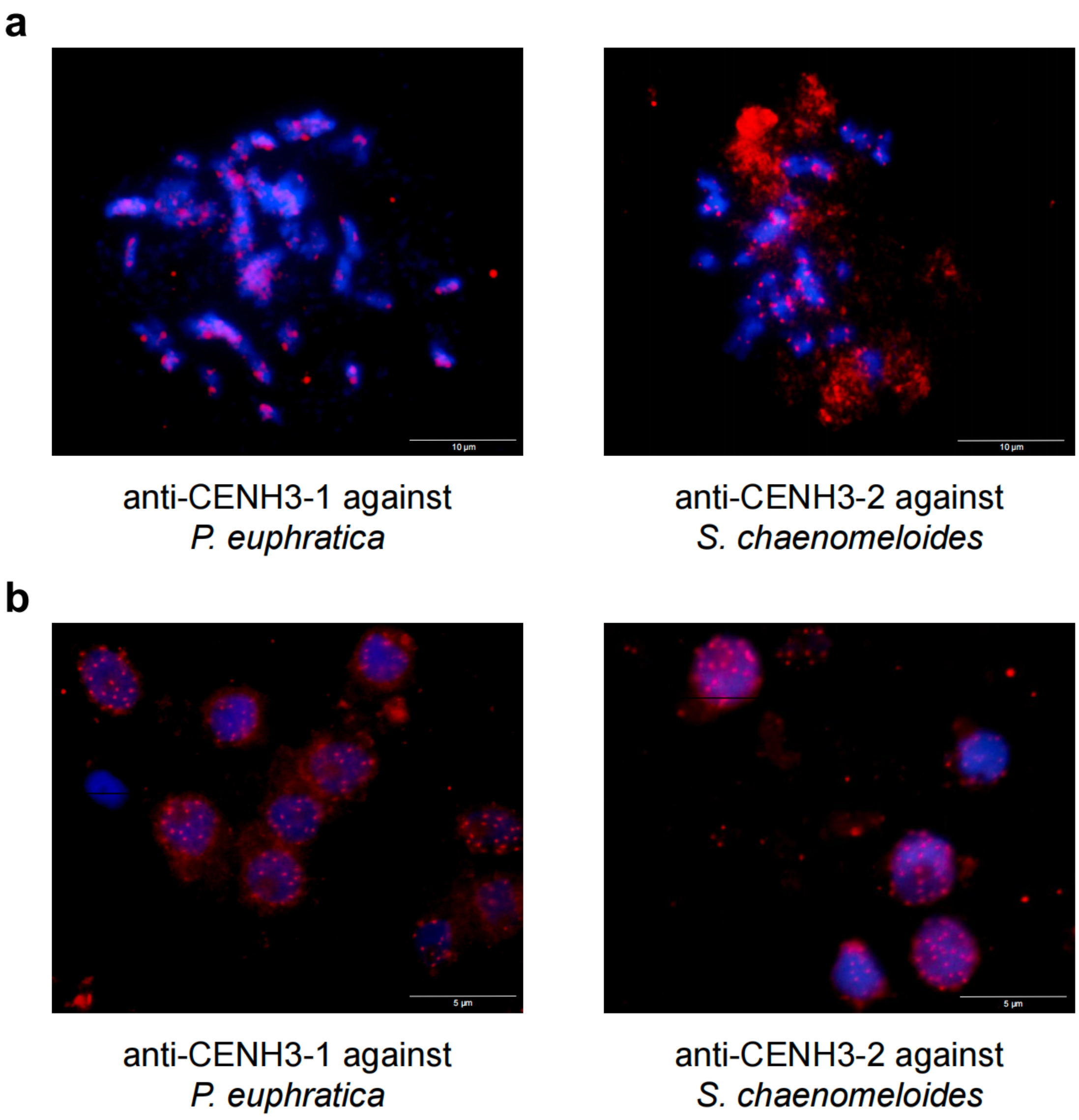

Fig. S5. Immunofluorescence assay of the anti-CENH3 antibodies on metaphase chromosomes (a) and interphase chromatin (b) in *P. euphratica* and *S. chaenomeloides*. Signals outside chromosome and chromatin represent background staining.

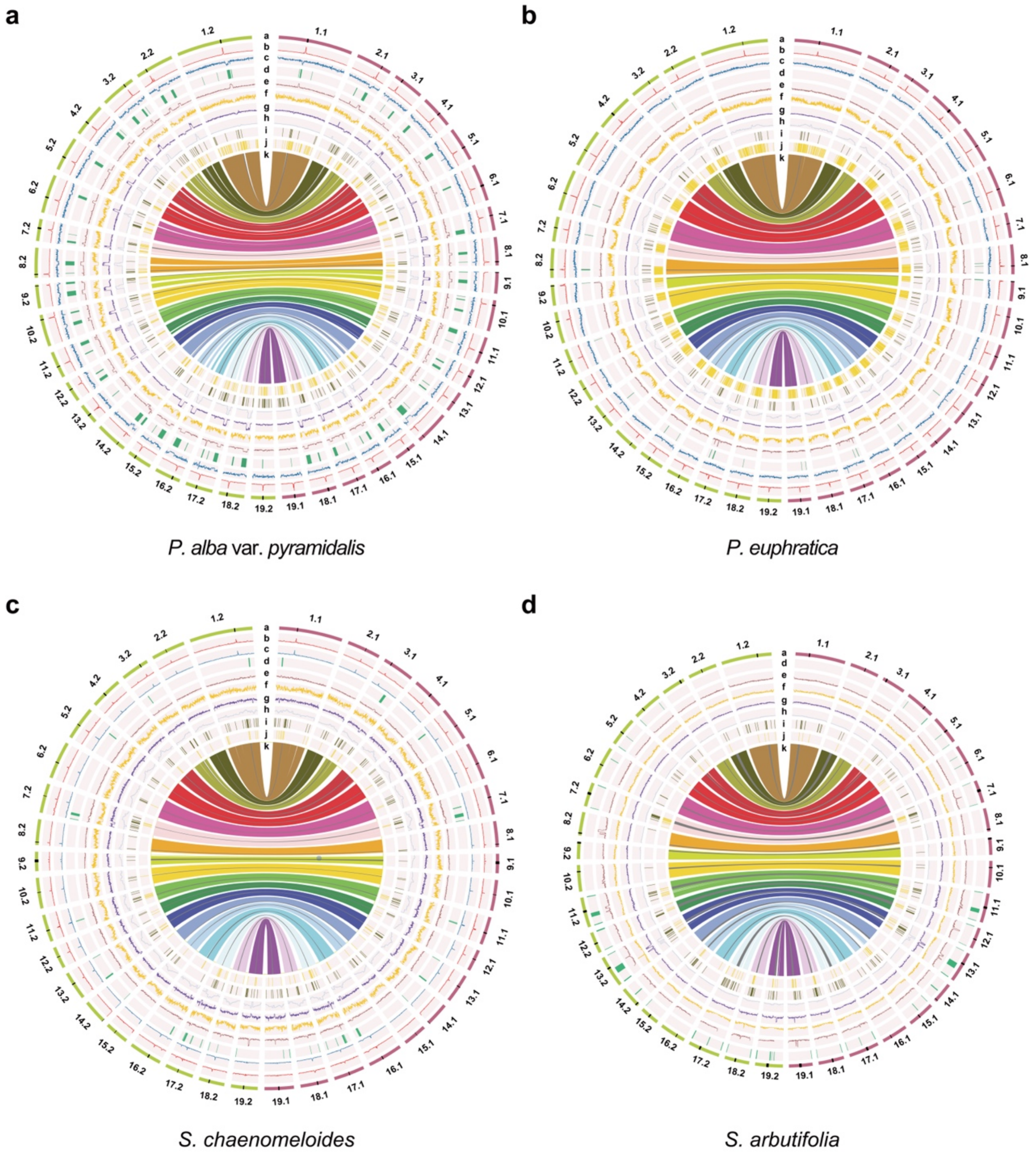

Fig. S6. Circos plots of genomic features for *P. alba* var. *pyramidalis* (a), *P. euphratica* (b), *S. chaenomeloides* (c) and *S. arbutifolia* (d). Circos from outer to inner separately represent chromosome and centromere position (a), CUT&Tag_CENH3-1 read coverage (b), CUT&Tag_CENH3-2 read coverage (c), tandem repeat distribution (d), GC content (e), gene density (f), transposable element density (g), LTR density (h), intact *CRM* distribution (i), intact *ATHILA* distribution (j) and allele gene colinear links (k). Centromeres in homologous chromosomes are connected by grey links.

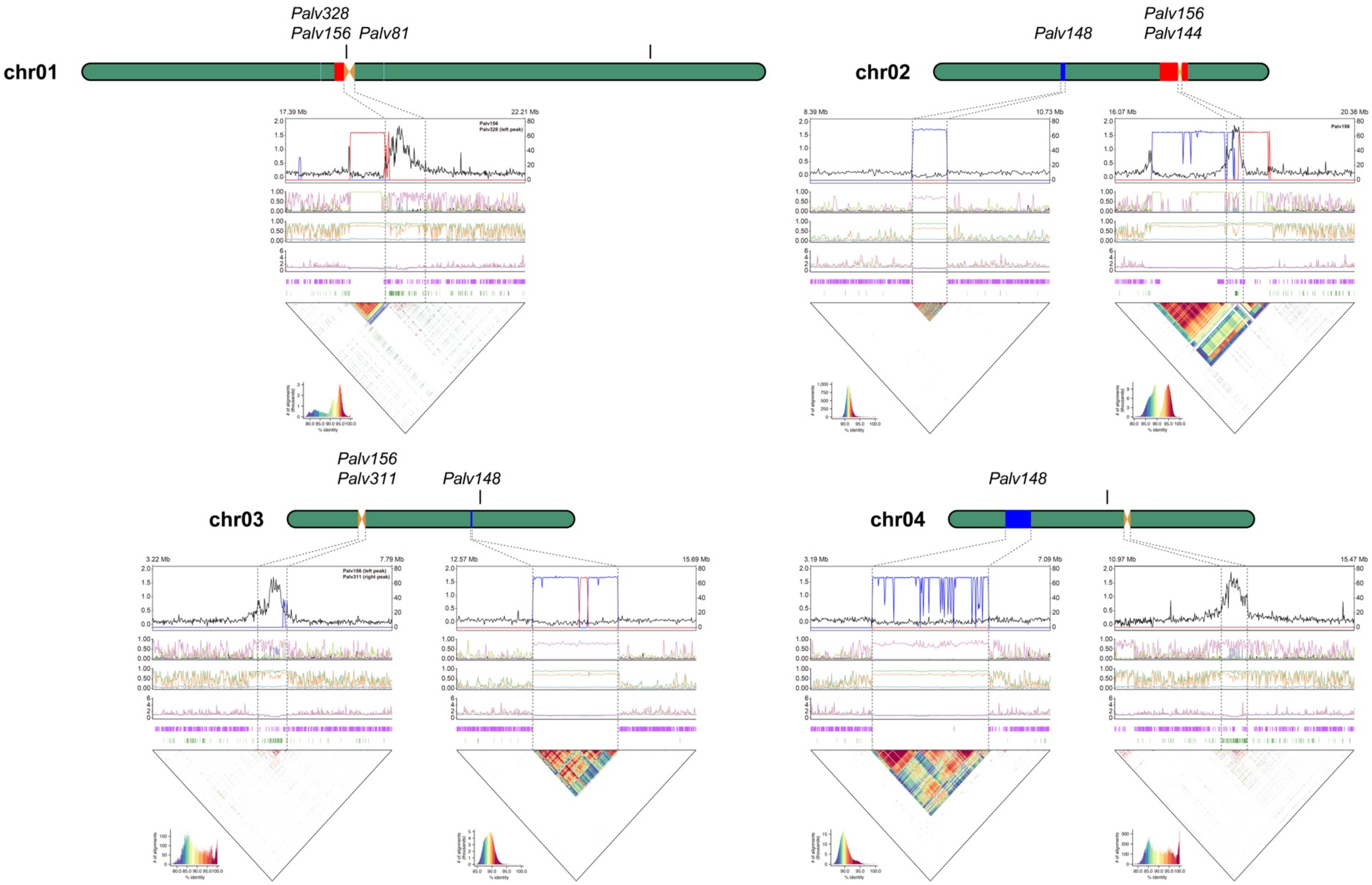
chr01
Palv328
Palv156
Palv81
17.39 Mb
22.21 Mb
chr02
Palv148
Palv156
Palv144
8.39 Mb
10.73 Mb
16.07 Mb
20.38 Mb
chr03
Palv156
Palv311
Palv148
3.22 Mb
7.79 Mb
12.57 Mb
15.69 Mb
chr04
Palv148
3.19 Mb
7.09 Mb
10.97 Mb
15.47 Mb

(continued)

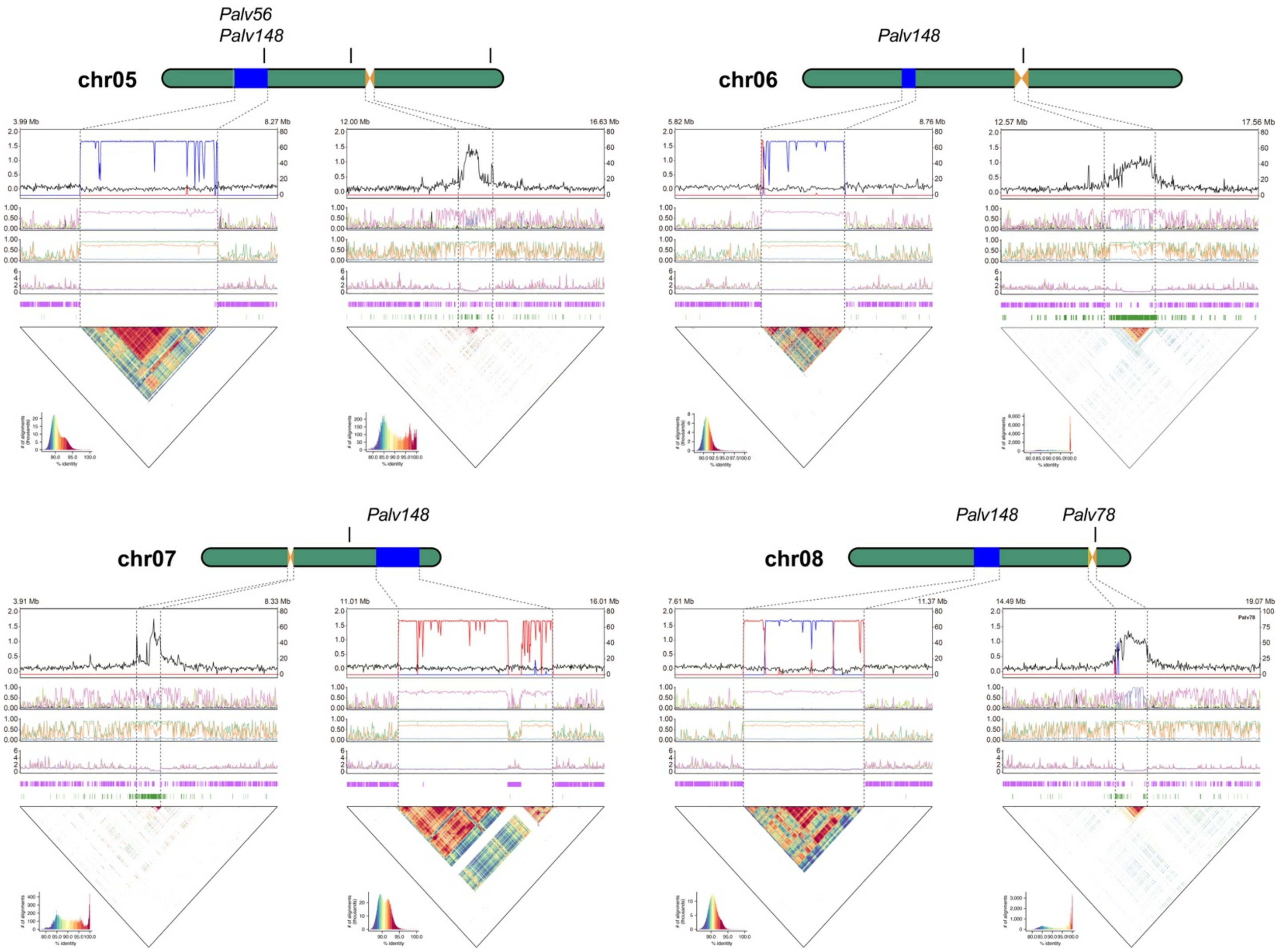
Palv56
Palv148
chr05
Palv148
chr06
Palv148
chr07
Palv148
Palv78
chr08
3.99 Mb
8.27 Mb
12.00 Mb
16.63 Mb
5.82 Mb
8.76 Mb
12.57 Mb
17.56 Mb
3.91 Mb
8.33 Mb
11.01 Mb
16.01 Mb
7.61 Mb
11.37 Mb
14.49 Mb
19.07 Mb
% identity

(continued)

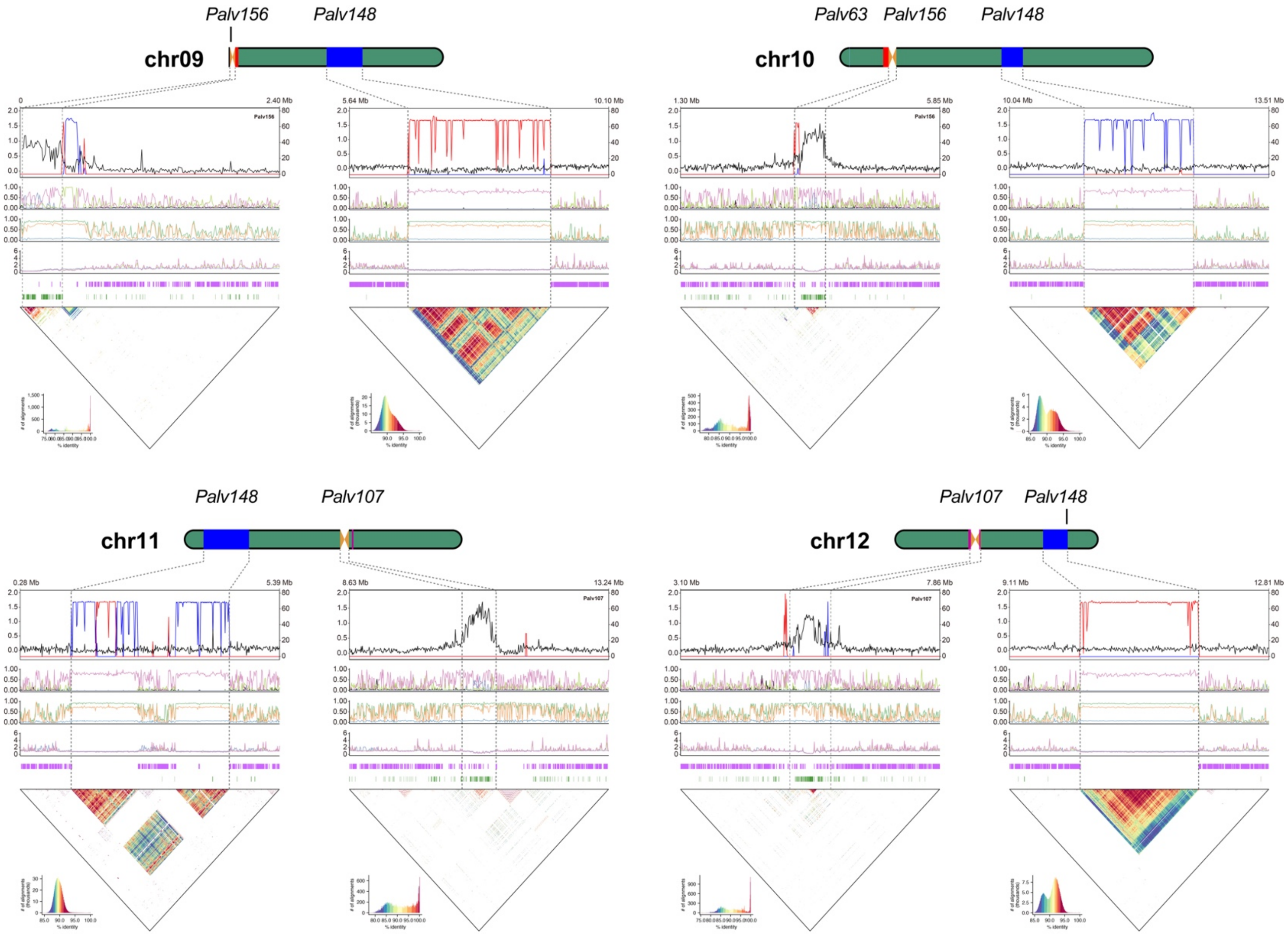

Palv156
Palv148
chr09
0
2.40 Mb
5.64 Mb
10.10 Mb
Palv63
Palv156
Palv148
chr10
1.30 Mb
5.85 Mb
10.04 Mb
13.51 Mb
Palv148
Palv107
chr11
0.28 Mb
5.39 Mb
8.63 Mb
13.24 Mb
Palv107
Palv148
chr12
3.10 Mb
7.86 Mb
9.11 Mb
12.81 Mb

(continued)

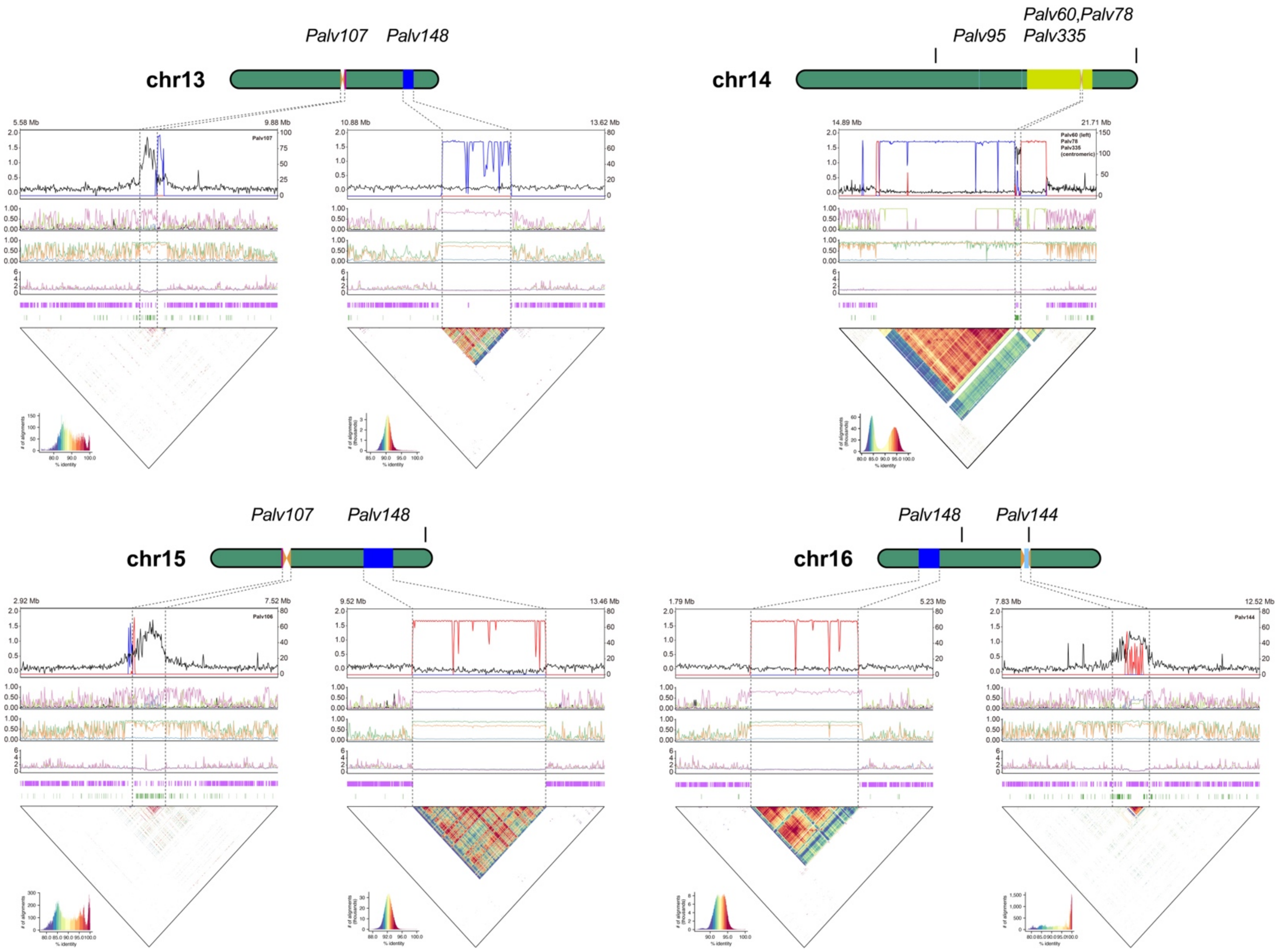

chr13
Palv107
Palv148
5.58 Mb
9.88 Mb
10.88 Mb
13.62 Mb
chr14
Palv95
Palv60,Palv78
Palv335
14.89 Mb
21.71 Mb
chr15
Palv107
Palv148
2.92 Mb
7.52 Mb
9.52 Mb
13.46 Mb
chr16
Palv148
Palv144
1.79 Mb
5.23 Mb
7.83 Mb
12.52 Mb

(continued)

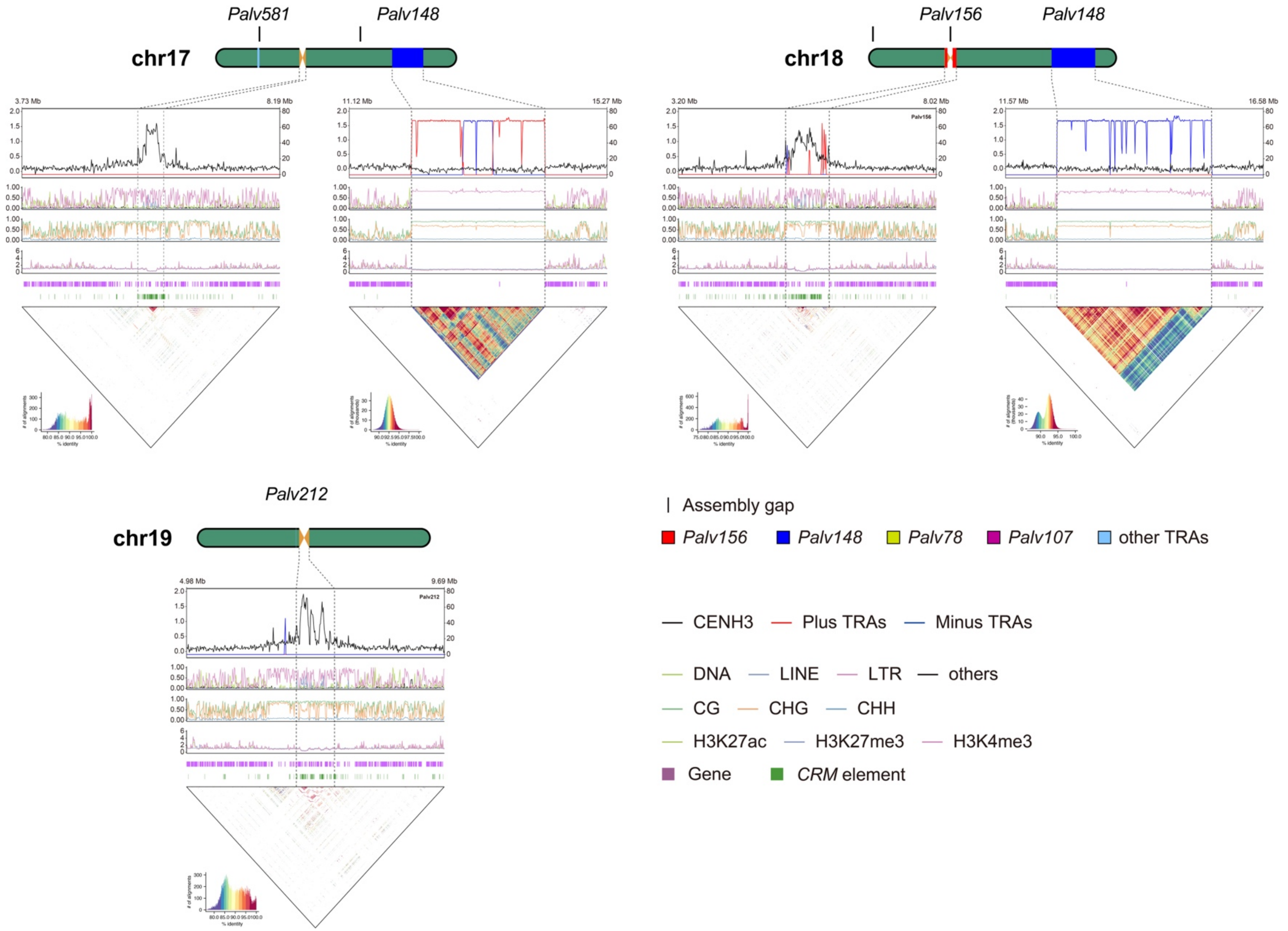

Palv581
Palv148
chr17
Palv156
Palv148
chr18
Palv212
chr19
Assembly gap
Palv156
Palv148
Palv78
Palv107
other TRAs
CENH3
Plus TRAs
Minus TRAs
DNA
LINE
LTR
others
CG
CHG
CHH
H3K27ac
H3K27me3
H3K4me3
Gene
CRM element

Fig. S7. Characteristics and epigenetics for centromeres and *Palv148* TRAs of *P. alba* var. *pyramidalis* haplotype I. Plots from top to bottom separately represent assembly gaps, tandem repeats on forward (red) and reverse (blue) strands and CENH3 CUT&Tag distribution per 10-kb, transposable element distribution, DNA methylation level, histone modification level, gene distribution, *CRM* element distribution and sequence similarity on centromeres and adjacent regions. Regions marked in orange triangles represent centromeres.

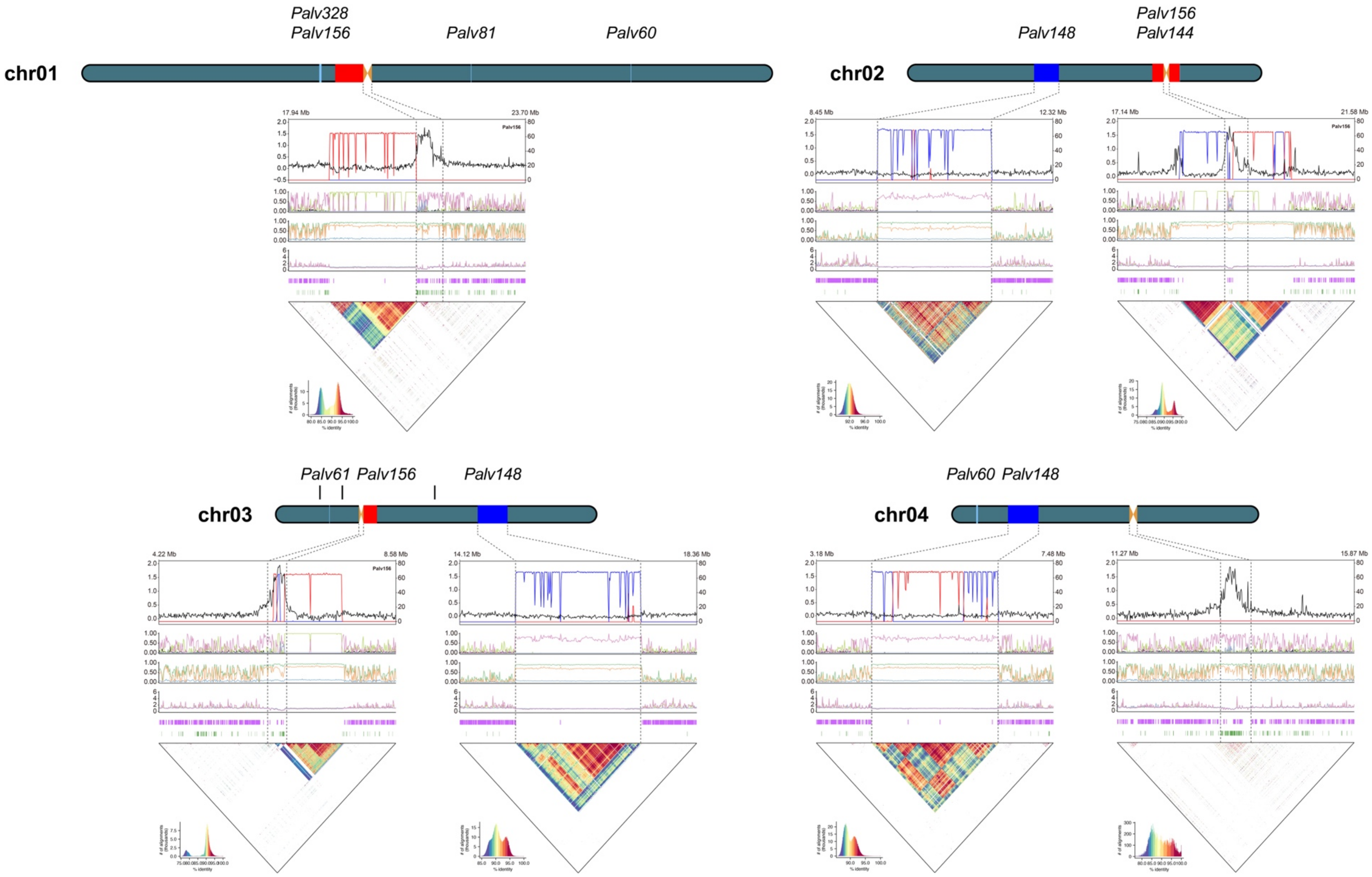
Palv328
Palv156
Palv81
Palv60
chr01
17.94 Mb
23.70 Mb
Palv148
Palv156
Palv144
chr02
8.45 Mb
12.32 Mb
17.14 Mb
21.58 Mb
Palv61
Palv156
Palv148
chr03
4.22 Mb
8.58 Mb
14.12 Mb
18.36 Mb
Palv60
Palv148
chr04
3.18 Mb
7.48 Mb
11.27 Mb
15.87 Mb

(continued)

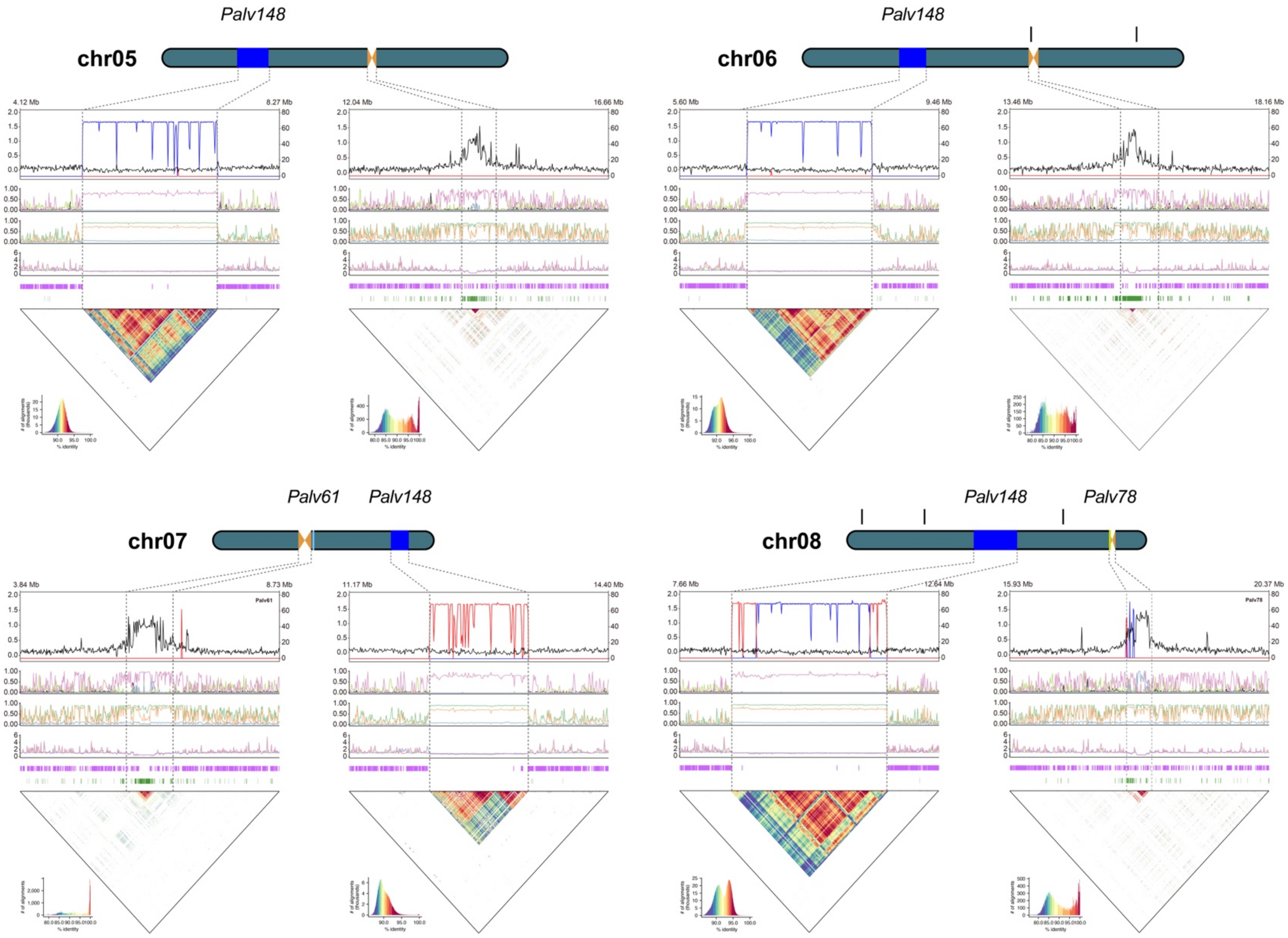

Palv148
chr05
Palv148
chr06
Palv61
Palv148
chr07
Palv148
Palv78
chr08

(continued)

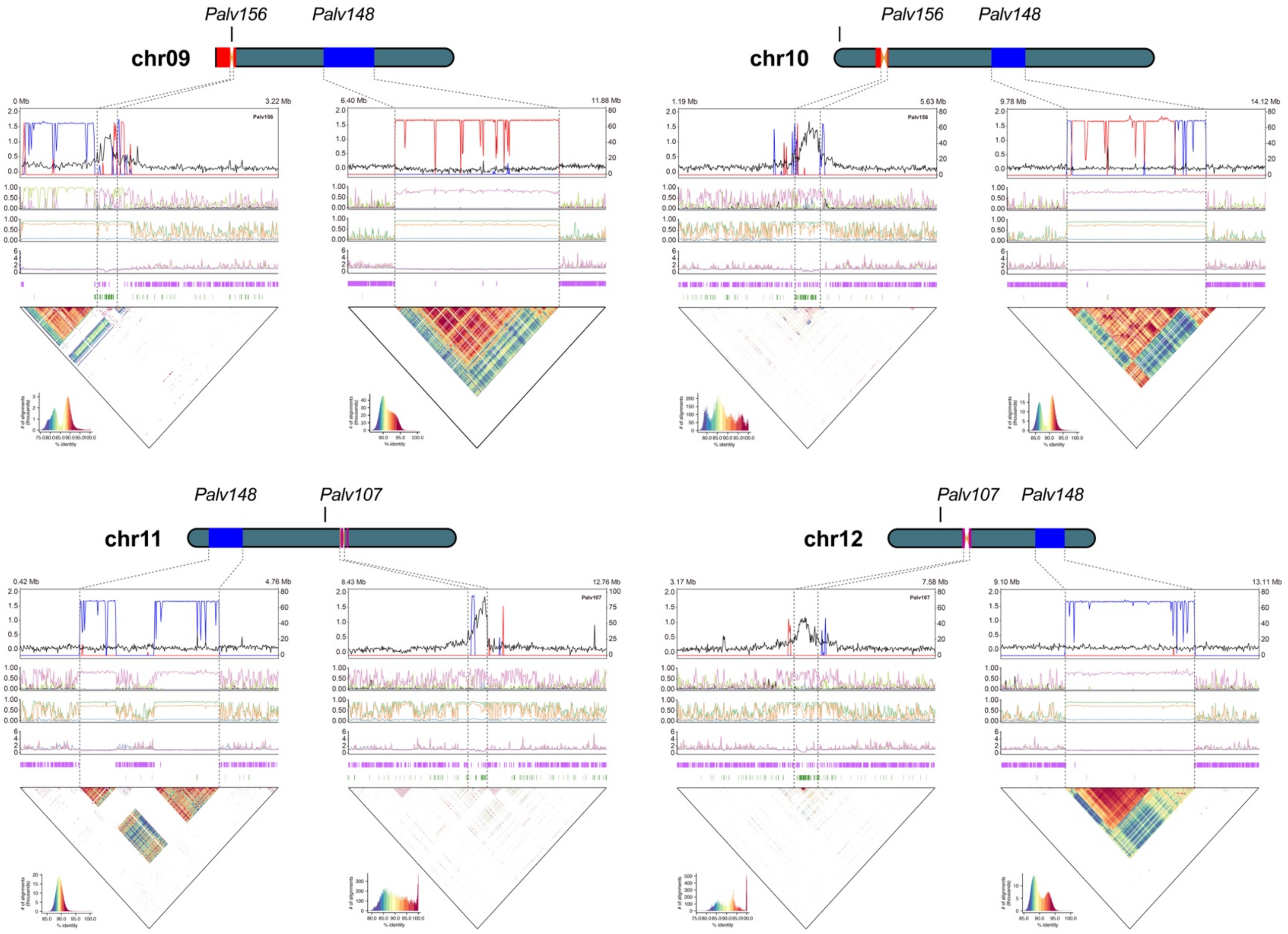

Palv156
Palv148
chr09
0 Mb
3.22 Mb
6.40 Mb
11.88 Mb
Palv156
Palv148
chr10
1.19 Mb
5.63 Mb
9.78 Mb
14.12 Mb
Palv148
Palv107
chr11
0.42 Mb
4.76 Mb
8.43 Mb
12.76 Mb
Palv107
Palv148
chr12
3.17 Mb
7.58 Mb
9.10 Mb
13.11 Mb

(continued)

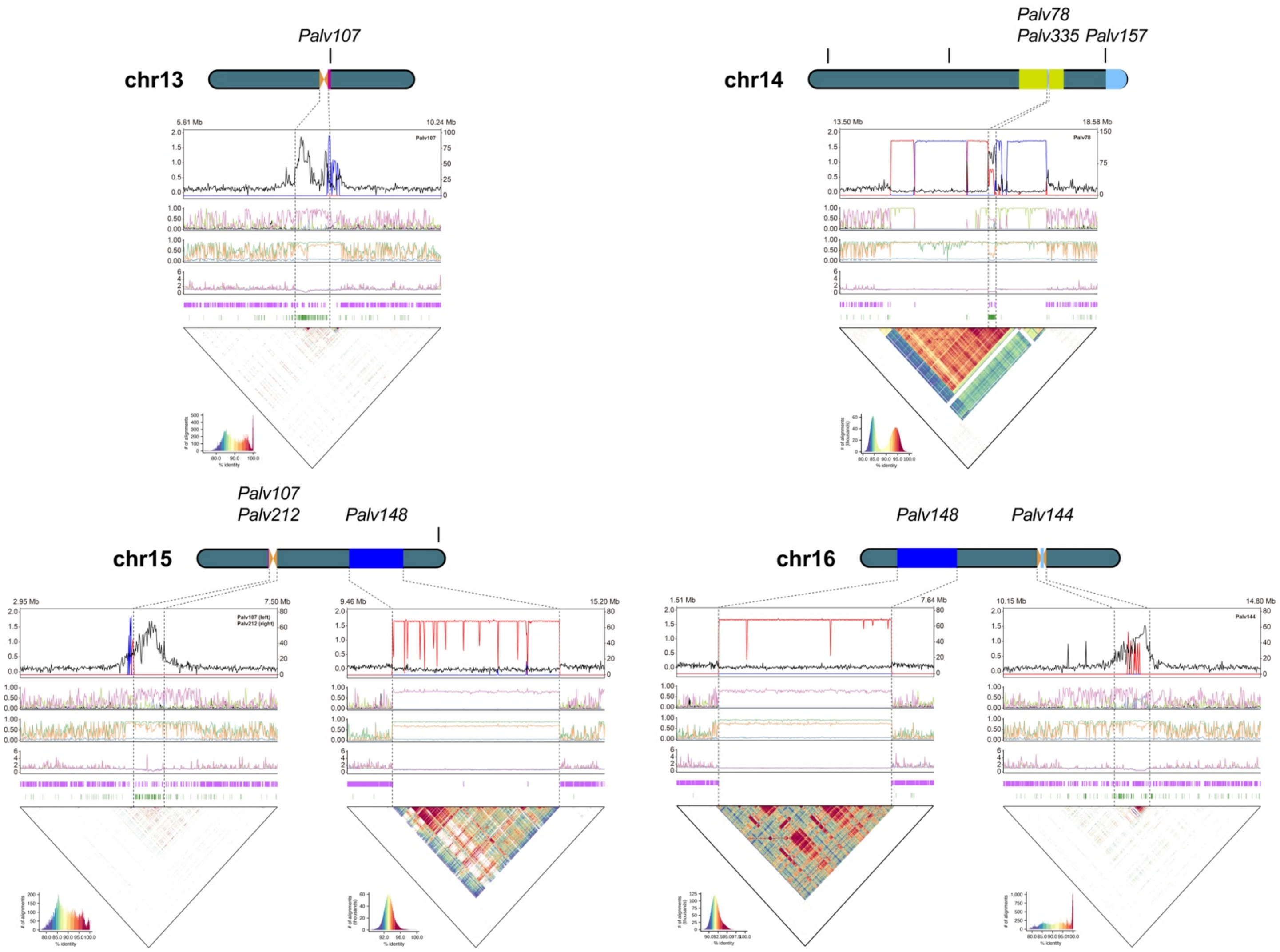
Palv107
chr13
5.61 Mb
10.24 Mb
Palv78
Palv335 Palv157
chr14
13.50 Mb
18.58 Mb
Palv107
Palv212
Palv148
chr15
2.95 Mb
7.50 Mb
9.46 Mb
15.20 Mb
Palv148
Palv144
chr16
1.51 Mb
7.64 Mb
10.15 Mb
14.80 Mb

(continued)

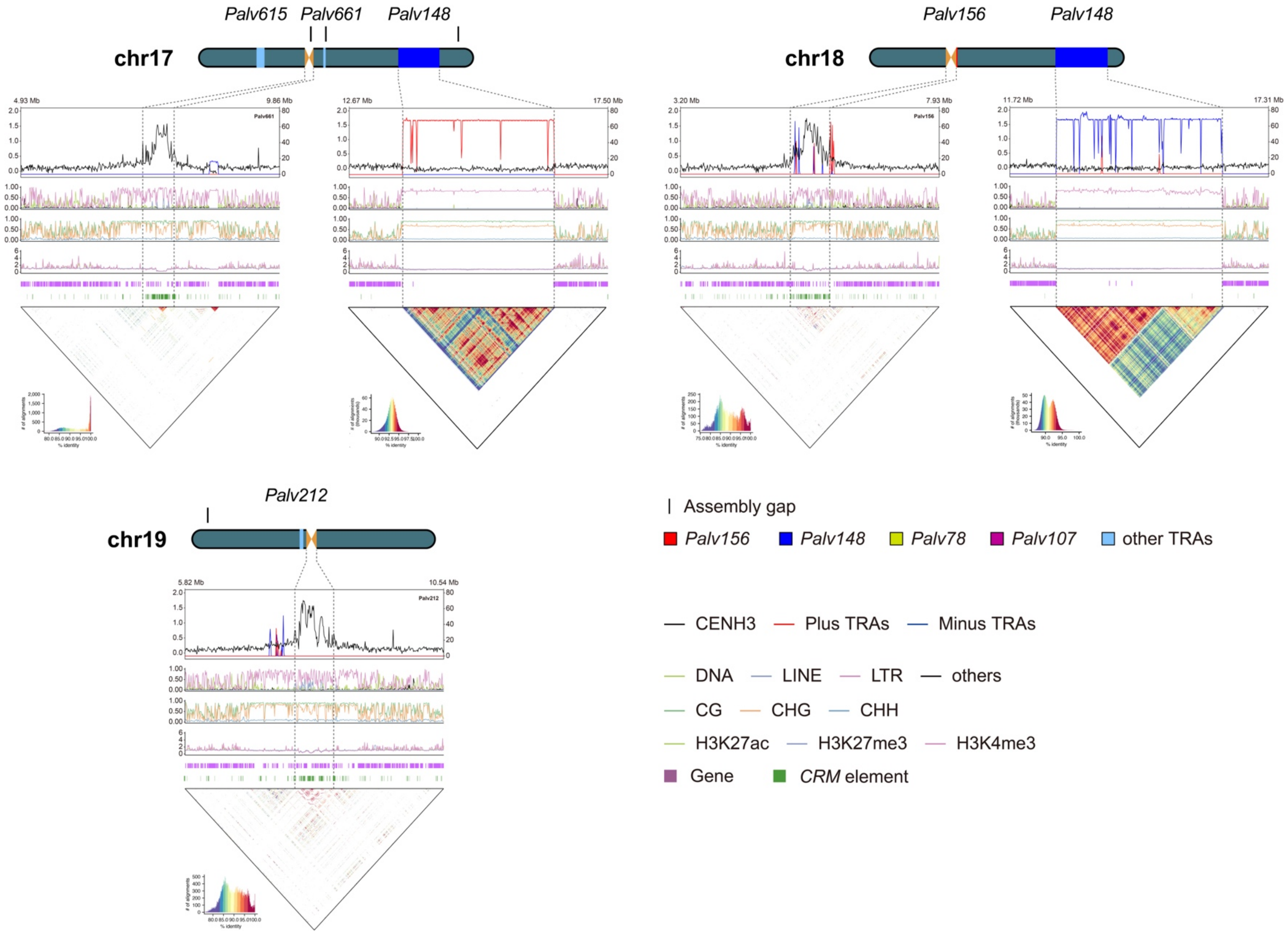

Palv615
Palv661
Palv148
chr17
Palv156
Palv148
chr18
Palv212
chr19
4.93 Mb
9.86 Mb
12.67 Mb
17.50 Mb
3.20 Mb
7.93 Mb
11.72 Mb
17.31 Mb
5.82 Mb
10.54 Mb
Assembly gap
Palv156
Palv148
Palv78
Palv107
other TRAs
CENH3
Plus TRAs
Minus TRAs
DNA
LINE
LTR
others
CG
CHG
CHH
H3K27ac
H3K27me3
H3K4me3
Gene
CRM element

Fig. S8. Characteristics and epigenetics for centromeres and *Palv148* TRAs of *P. alba* var. *pyramidalis* haplotype II. Plots from top to bottom separately represent assembly gaps, tandem repeats on forward (red) and reverse (blue) strands and CENH3 CUT&Tag distribution per 10-kb, transposable element distribution, DNA methylation level, histone modification level, gene distribution, *CRM* element distribution and sequence similarity on centromeres and adjacent regions. Regions marked in orange triangles represent centromeres.

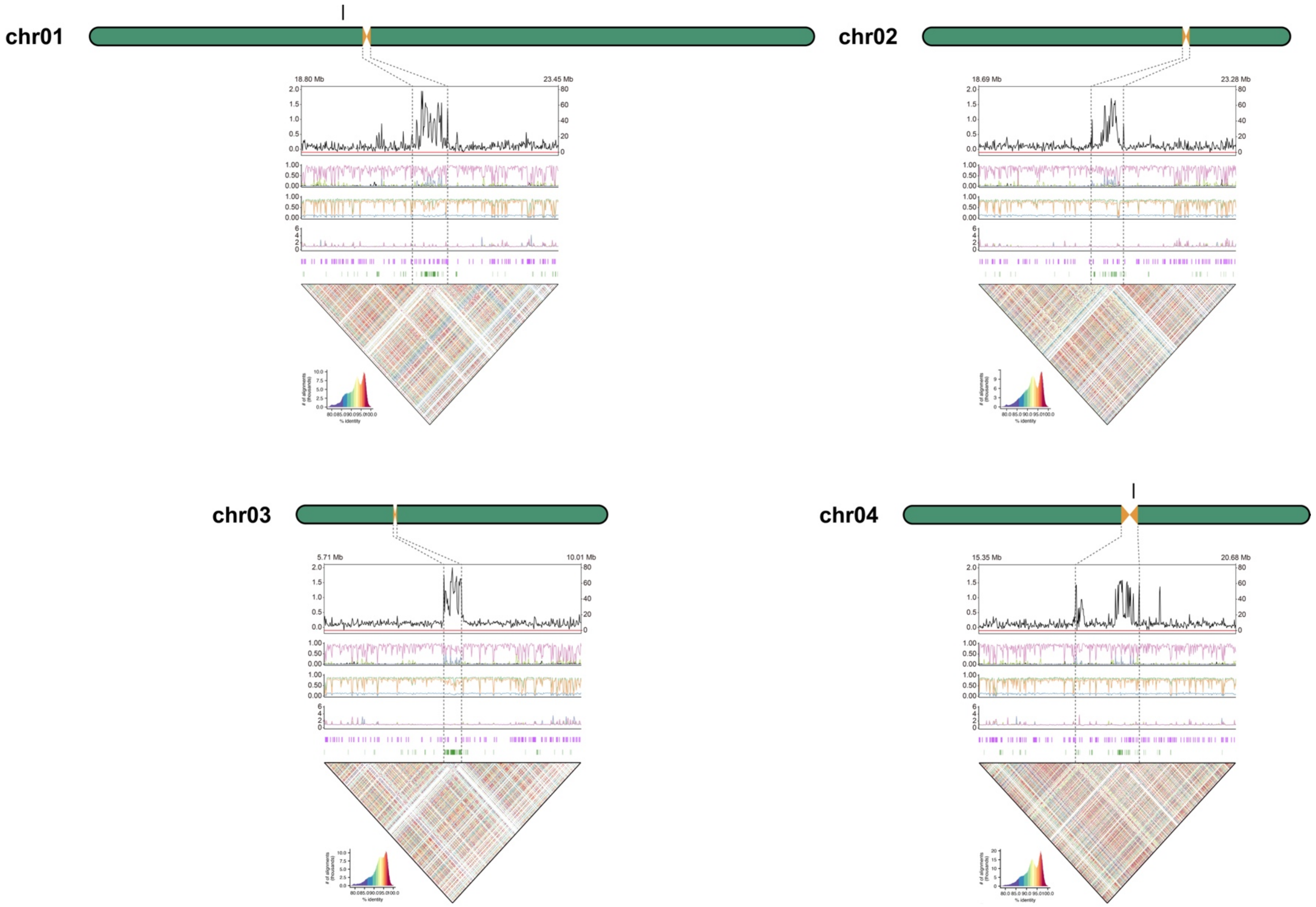
chr01
18.80 Mb
23.45 Mb
chr02
18.69 Mb
23.28 Mb
chr03
5.71 Mb
10.01 Mb
chr04
15.35 Mb
20.68 Mb

(continued)

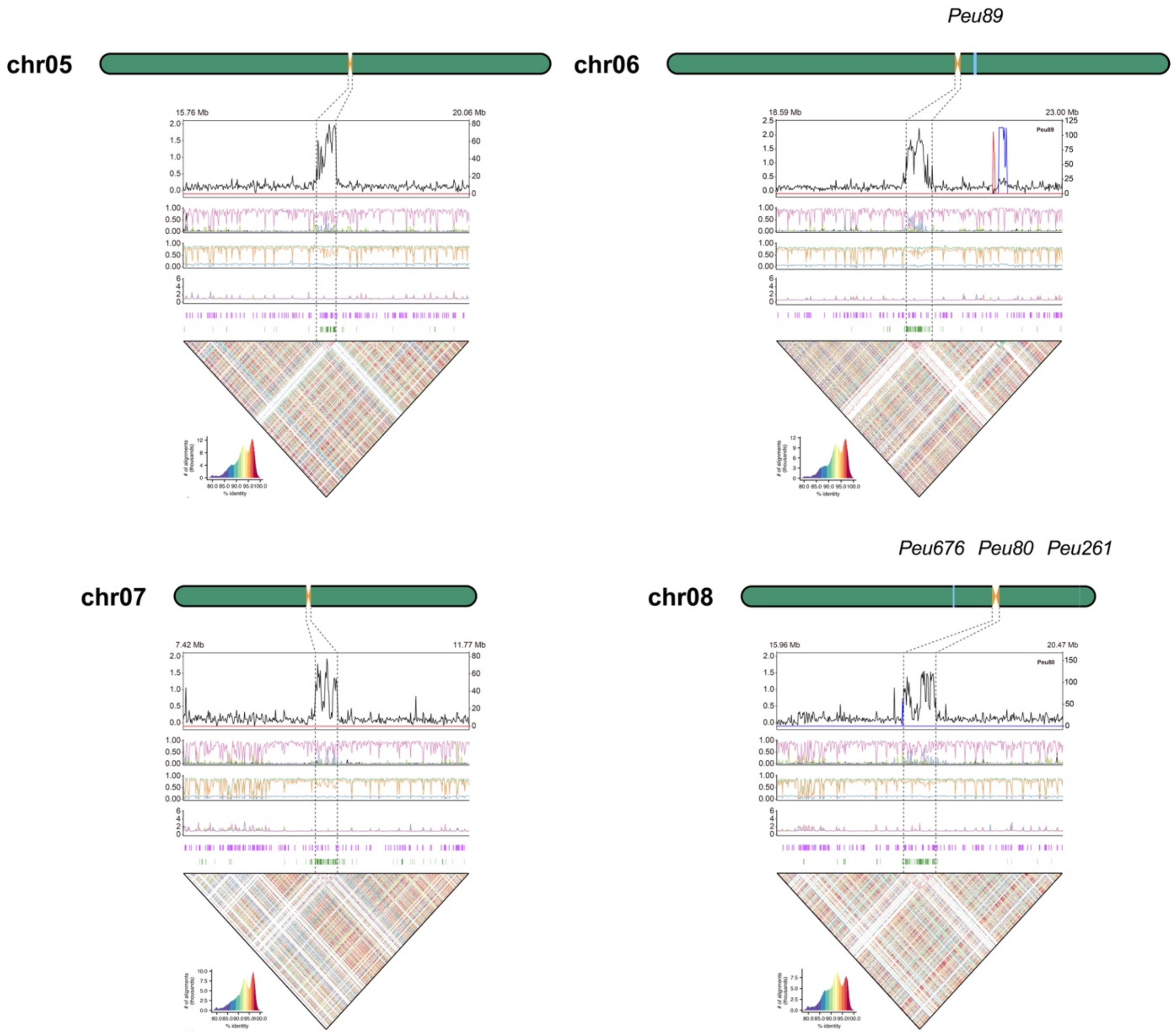
Peu89
chr05
chr06
15.76 Mb
20.06 Mb
18.59 Mb
23.00 Mb
Peu676
Peu80
Peu261
chr07
chr08
7.42 Mb
11.77 Mb
15.96 Mb
20.47 Mb

(continued)

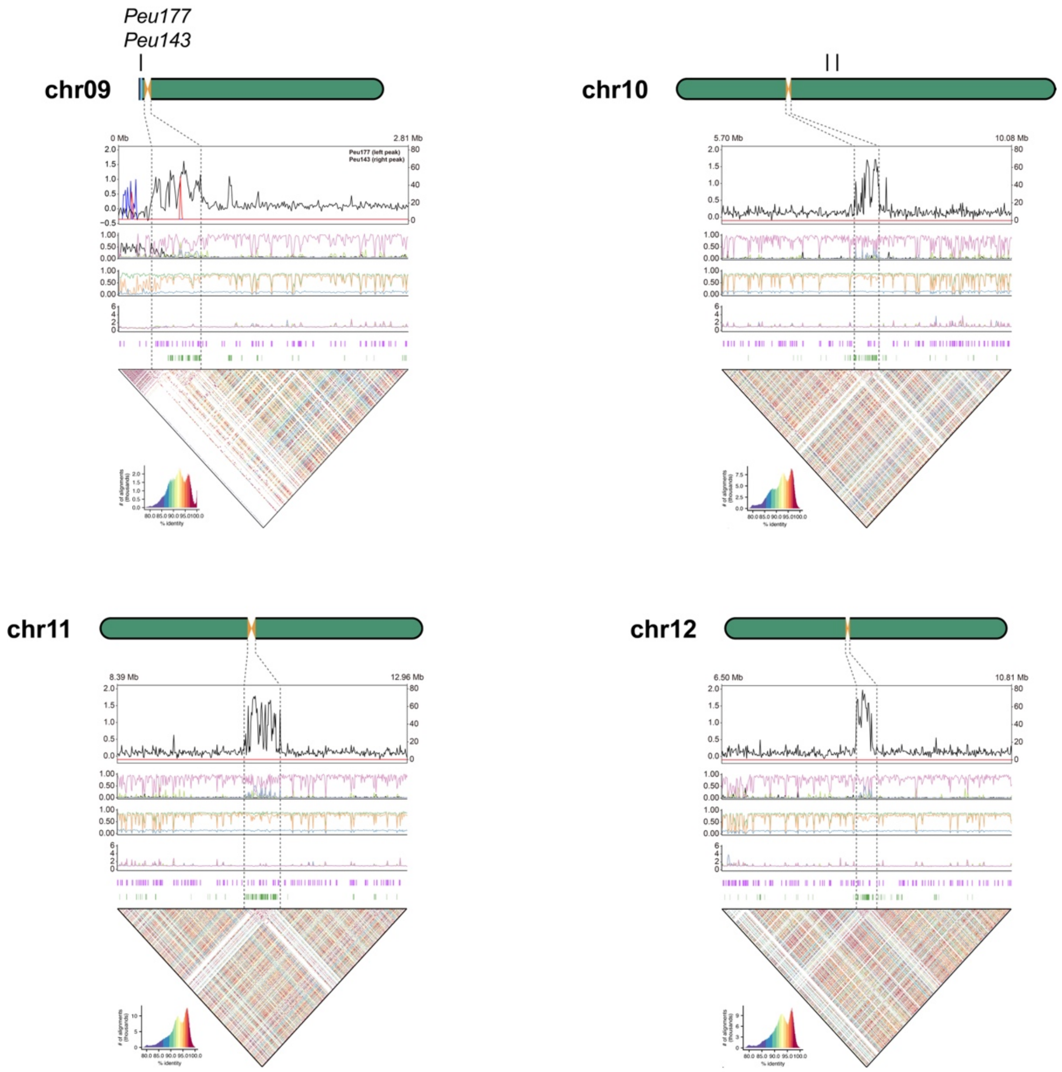
Peu177
Peu143
chr09
0 Mb
2.81 Mb
Peu177 (left peak)
Peu143 (right peak)
chr10
5.70 Mb
10.08 Mb
chr11
8.39 Mb
12.96 Mb
chr12
6.50 Mb
10.81 Mb

(continued)

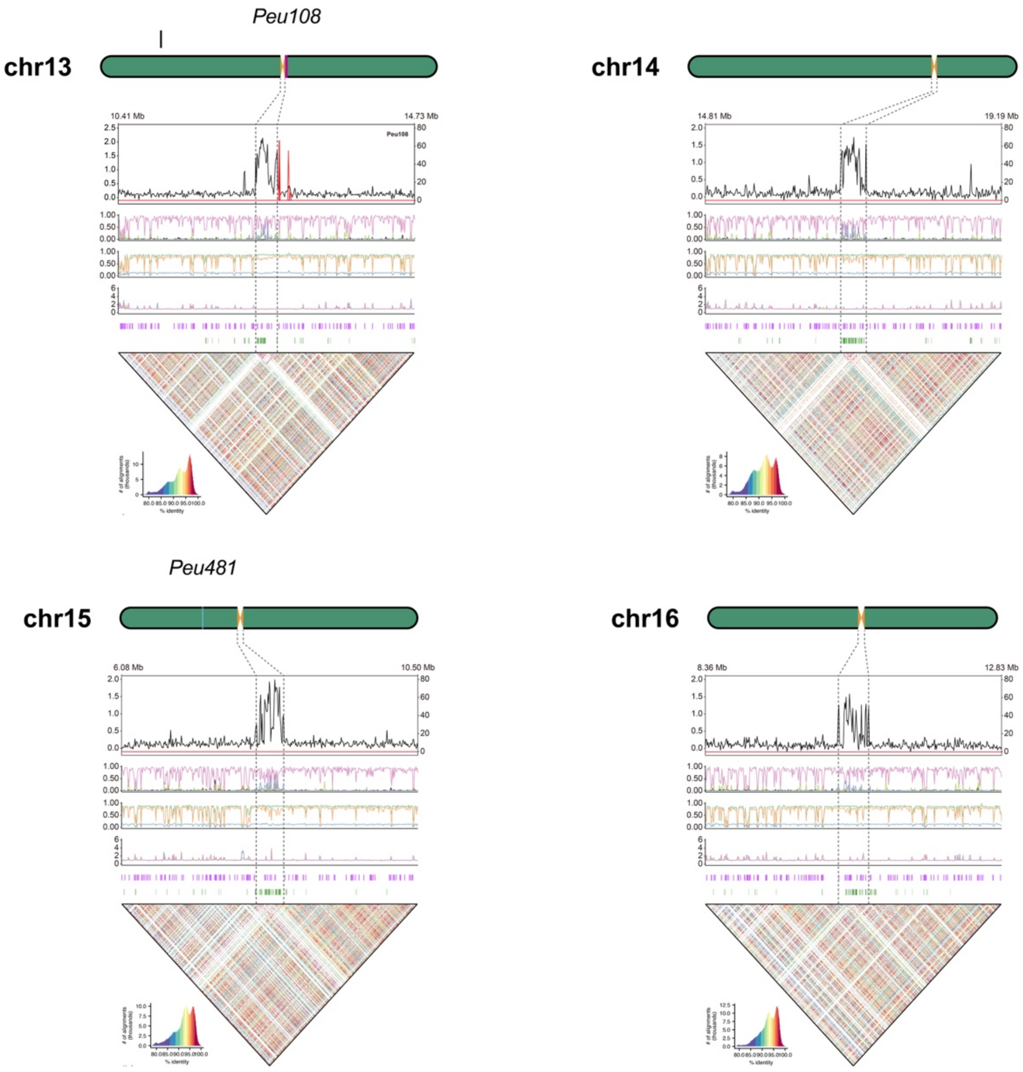
Peu108
chr13
10.41 Mb
14.73 Mb
chr14
14.81 Mb
19.19 Mb
Peu481
chr15
6.08 Mb
10.50 Mb
chr16
8.36 Mb
12.83 Mb

(continued)

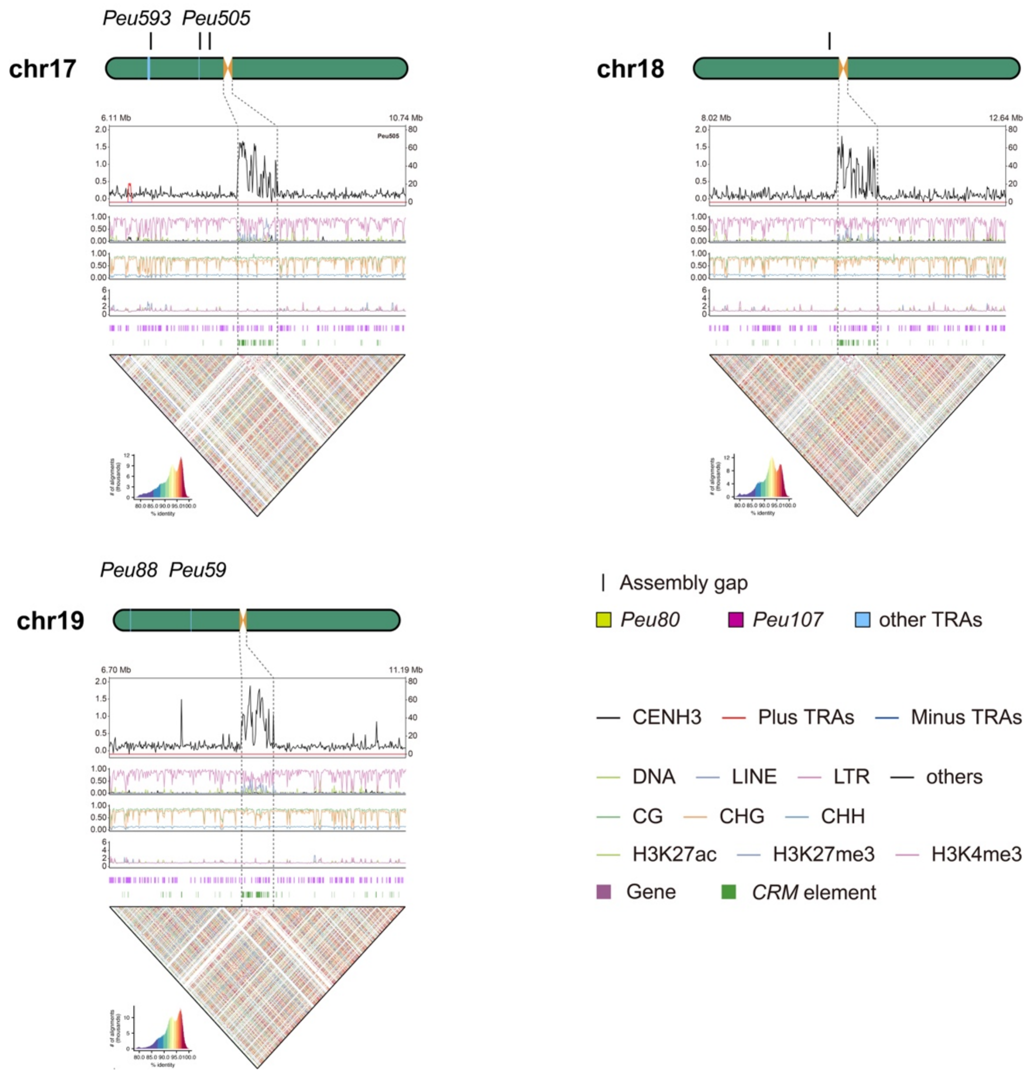

Peu593
Peu505
chr17
6.11 Mb
10.74 Mb
chr18
8.02 Mb
12.64 Mb
Peu88
Peu59
chr19
6.70 Mb
11.19 Mb
Assembly gap
Peu80
Peu107
other TRAs
CENH3
Plus TRAs
Minus TRAs
DNA
LINE
LTR
others
CG
CHG
CHH
H3K27ac
H3K27me3
H3K4me3
Gene
CRM element

Fig. S9. Characteristics and epigenetics for centromeres of *P. euphratica* haplotype I. Plots from top to bottom separately represent assembly gaps, tandem repeats on forward (red) and reverse (blue) strands and CENH3 CUT&Tag distribution per 10-kb, transposable element distribution, DNA methylation level, histone modification level, gene distribution, *CRM* element distribution and sequence similarity on centromeres and adjacent regions. Regions marked in orange triangles represent centromeres.

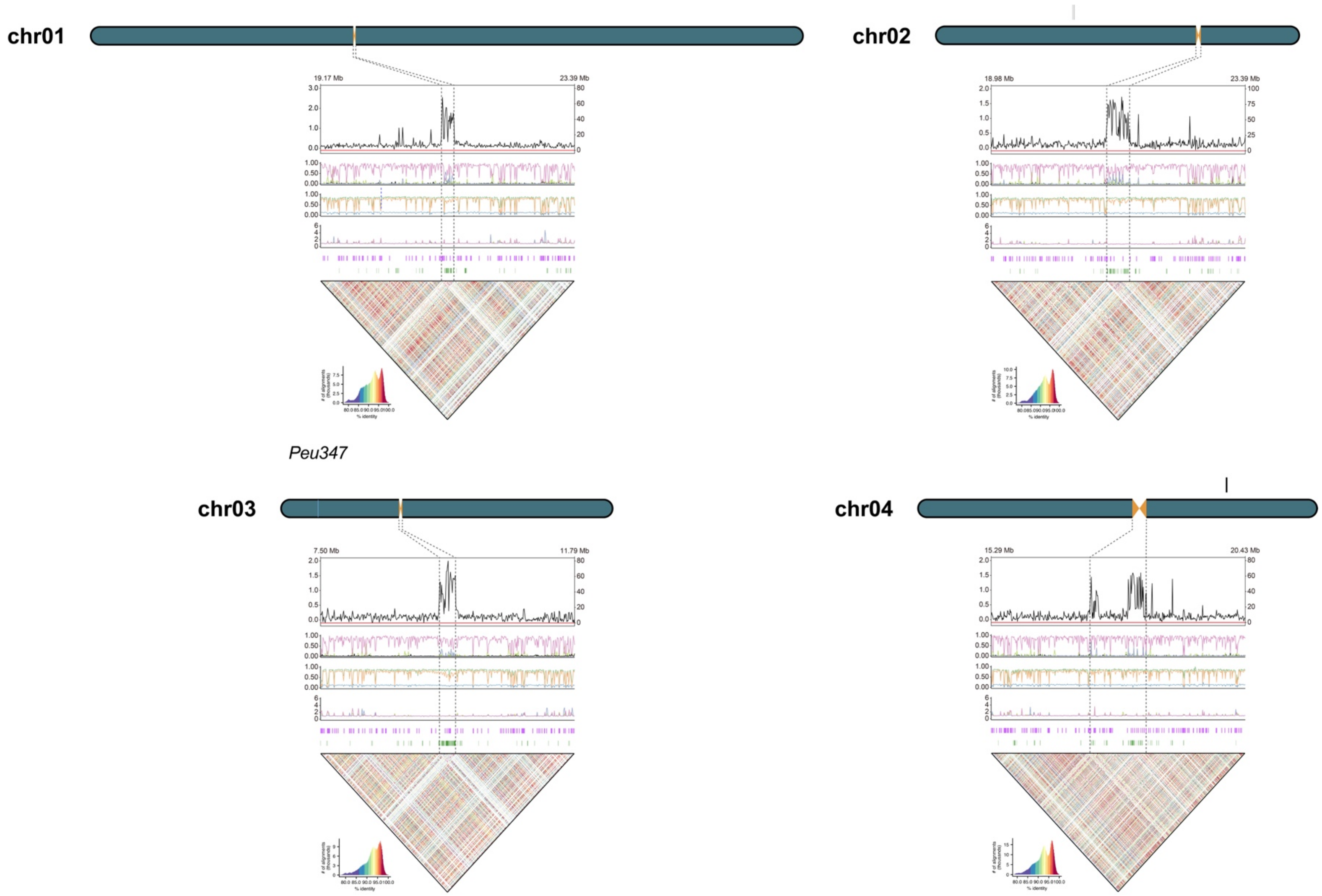
chr01
19.17 Mb
23.39 Mb
chr02
18.98 Mb
23.39 Mb
Peu347
chr03
7.50 Mb
11.79 Mb
chr04
15.29 Mb
20.43 Mb

(continued)

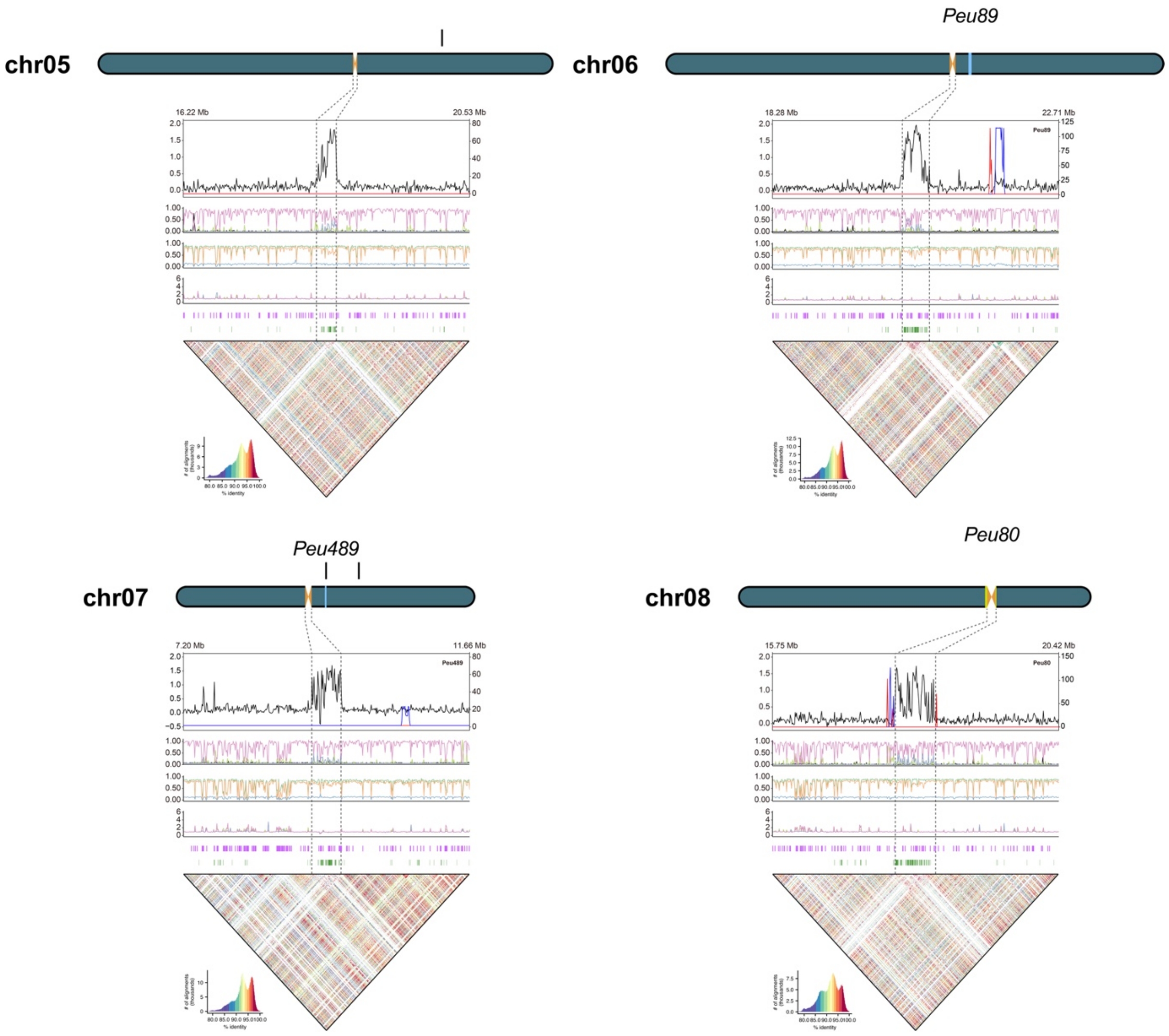

chr05
16.22 Mb
20.53 Mb
Peu89
chr06
18.28 Mb
22.71 Mb
Peu489
chr07
7.20 Mb
11.66 Mb
Peu80
chr08
15.75 Mb
20.42 Mb

(continued)

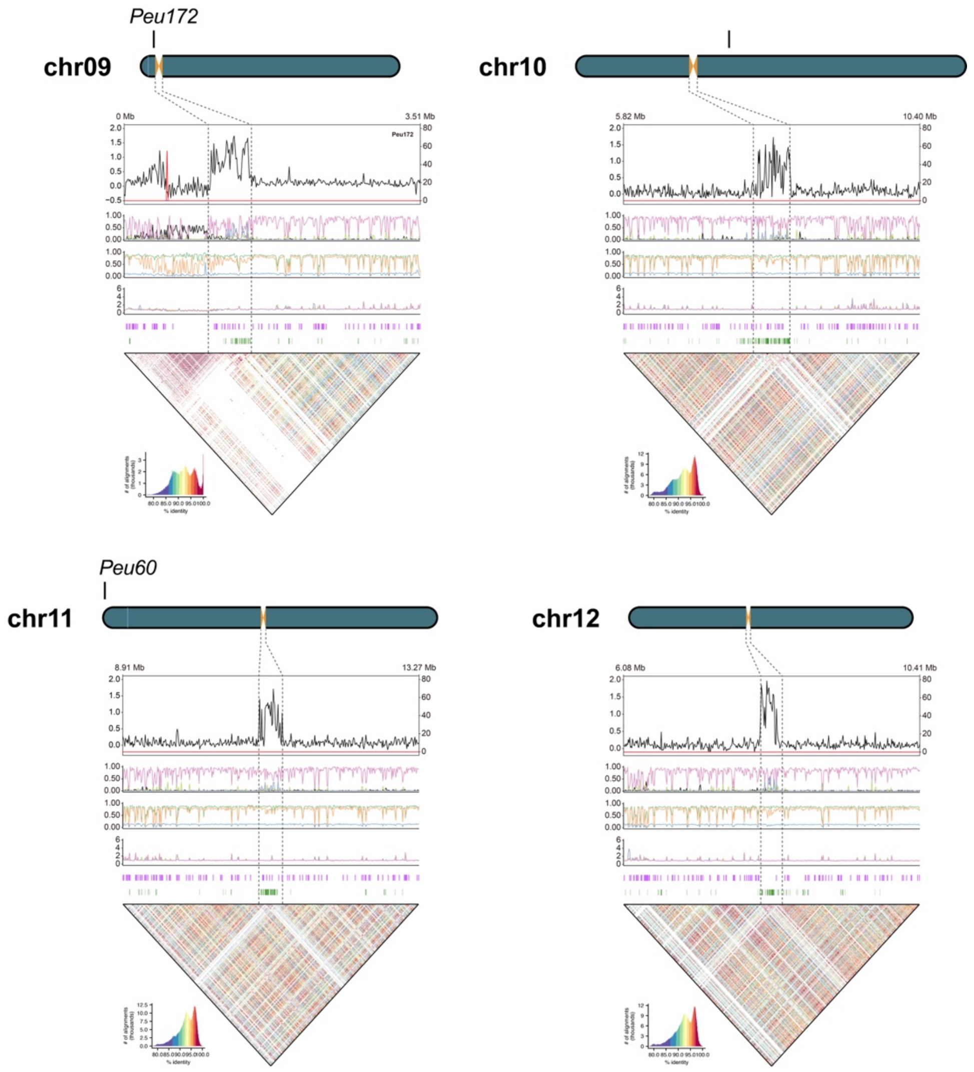
Peu172
chr09
chr10
0 Mb
3.51 Mb
5.82 Mb
10.40 Mb
Peu60
chr11
chr12
8.91 Mb
13.27 Mb
6.08 Mb
10.41 Mb
% identity

(continued)

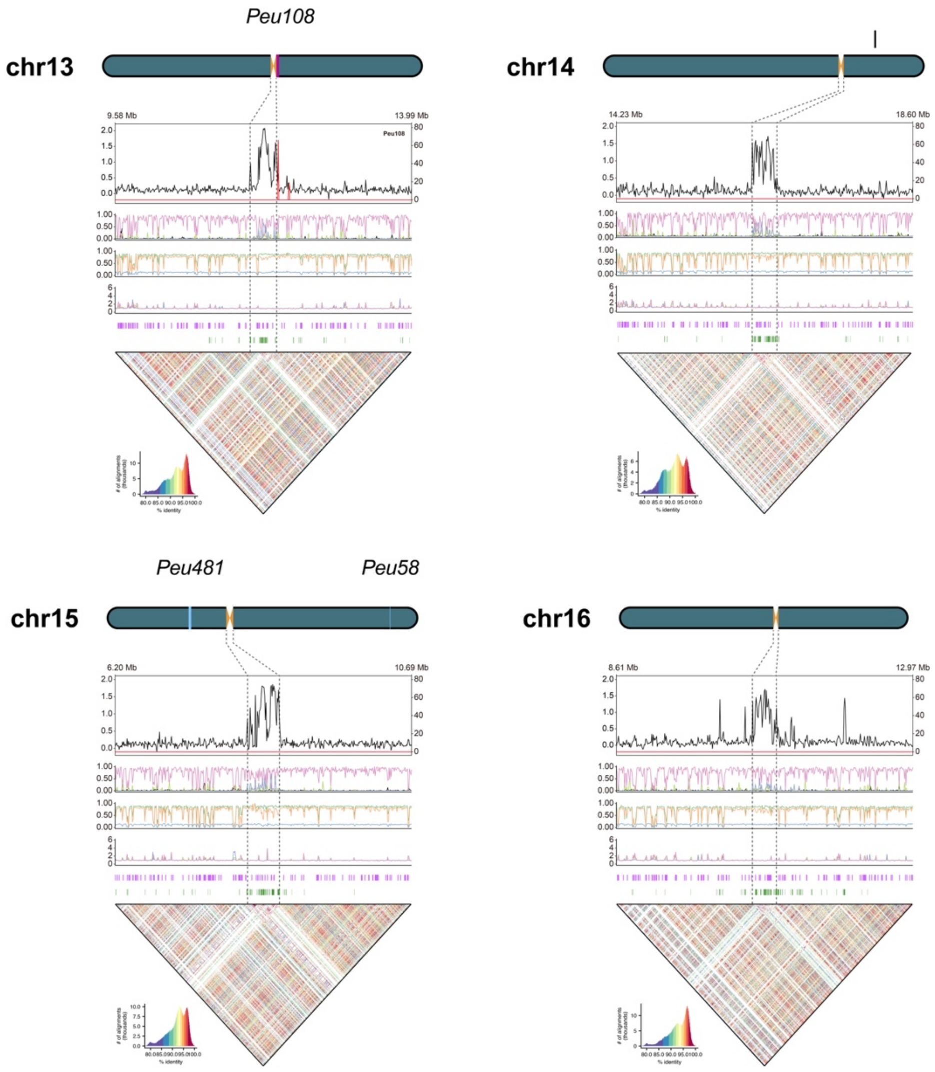
Peu108
chr13
chr14
9.58 Mb
13.99 Mb
Peu108
14.23 Mb
18.60 Mb
Peu481
Peu58
chr15
chr16
6.20 Mb
10.69 Mb
8.61 Mb
12.97 Mb

(continued)

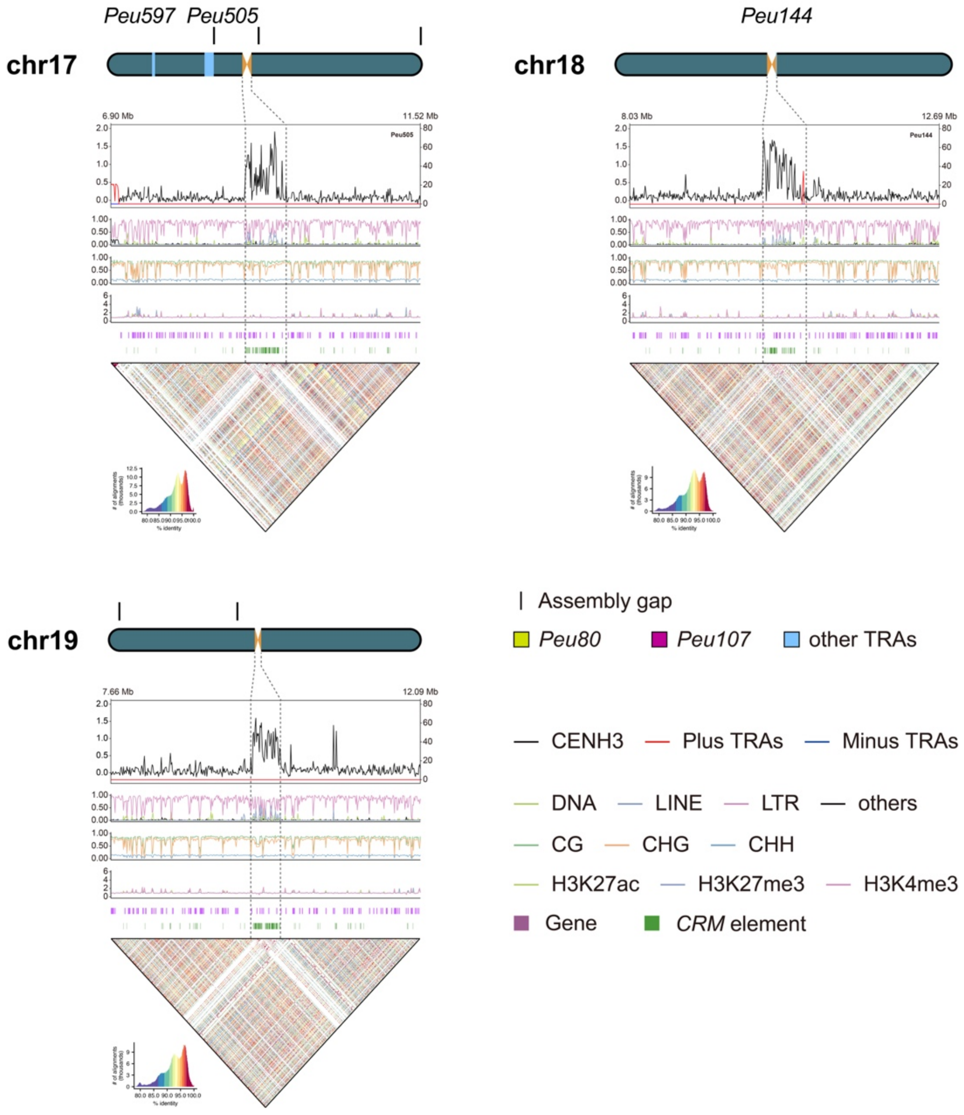

Peu597
Peu505
chr17
6.90 Mb
11.52 Mb
Peu505
Peu144
chr18
8.03 Mb
12.69 Mb
Peu144
chr19
7.66 Mb
12.09 Mb
% identity
Assembly gap
Peu80
Peu107
other TRAs
CENH3
Plus TRAs
Minus TRAs
DNA
LINE
LTR
others
CG
CHG
CHH
H3K27ac
H3K27me3
H3K4me3
Gene
CRM element

Fig. S10. Characteristics and epigenetics of centromeres for *P. euphratica* haplotype II. Plots from top to bottom separately represent assembly gaps, tandem repeats on forward (red) and reverse (blue) strands and CENH3 CUT&Tag distribution per 10-kb, transposable element distribution, DNA methylation level, histone modification level, gene distribution, *CRM* element distribution and sequence similarity on centromeres and adjacent regions. Regions marked in orange triangles represent centromeres.

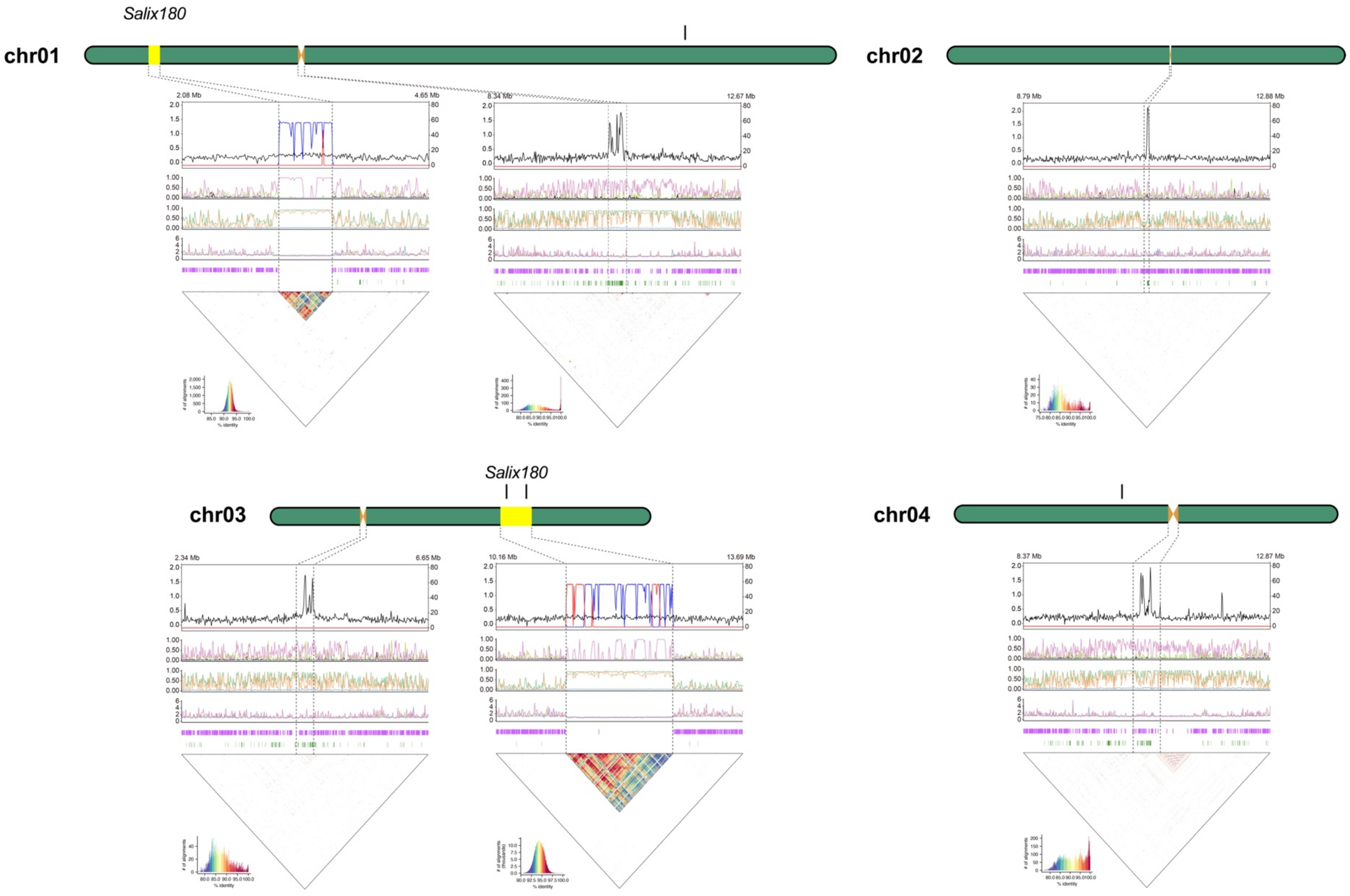
Salix180
chr01
chr02
2.08 Mb
4.65 Mb
8.34 Mb
12.67 Mb
8.79 Mb
12.88 Mb
Salix180
chr03
chr04
2.34 Mb
6.65 Mb
10.16 Mb
13.69 Mb
8.37 Mb
12.87 Mb

(continued)

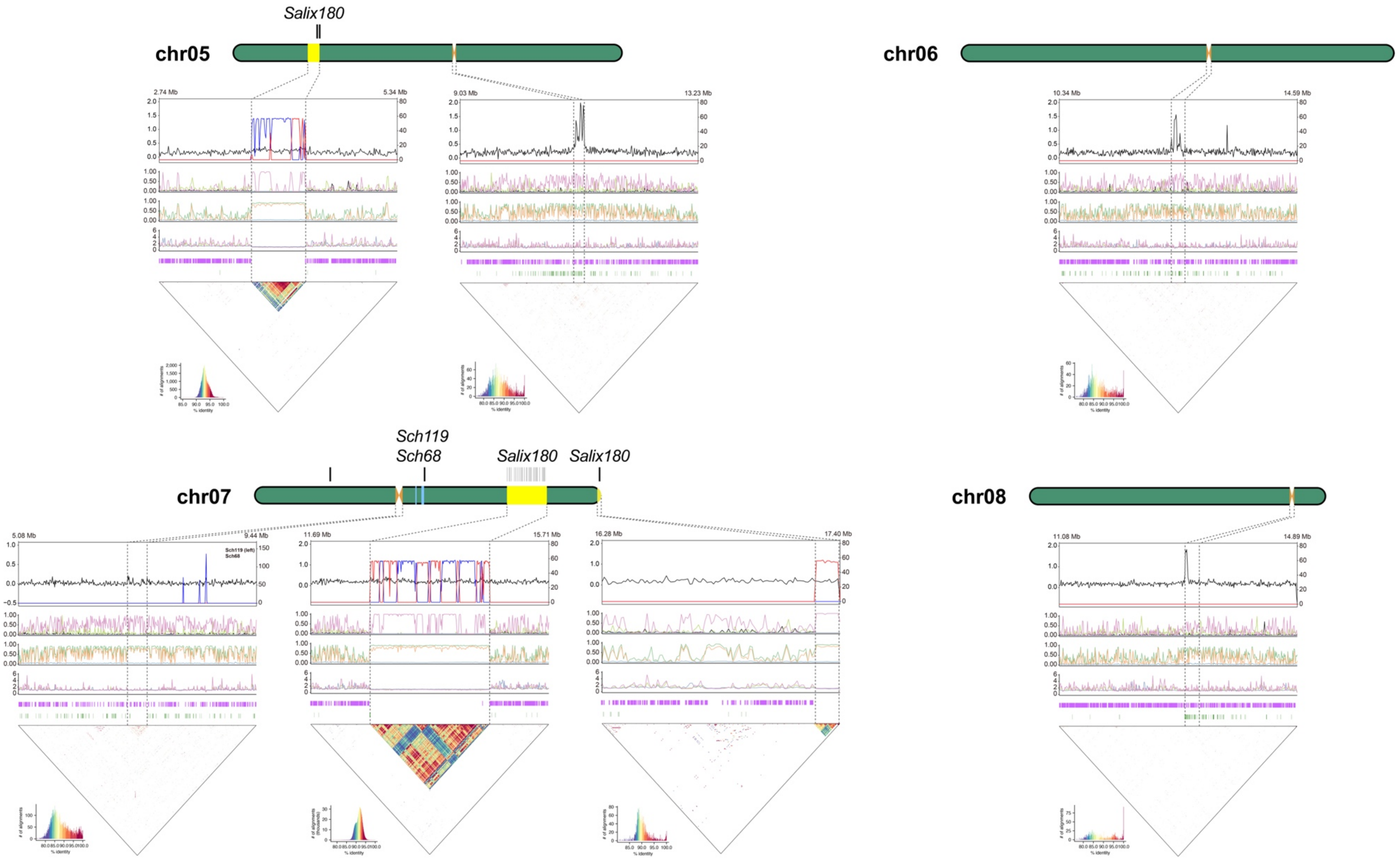

Salix180
chr05
chr06
2.74 Mb
5.34 Mb
9.03 Mb
13.23 Mb
10.34 Mb
14.59 Mb
Sch119
Sch68
Salix180
Salix180
chr07
chr08
5.08 Mb
9.44 Mb
Sch119 (left)
Sch68
11.69 Mb
15.71 Mb
16.28 Mb
17.40 Mb
11.08 Mb
14.89 Mb

(continued)

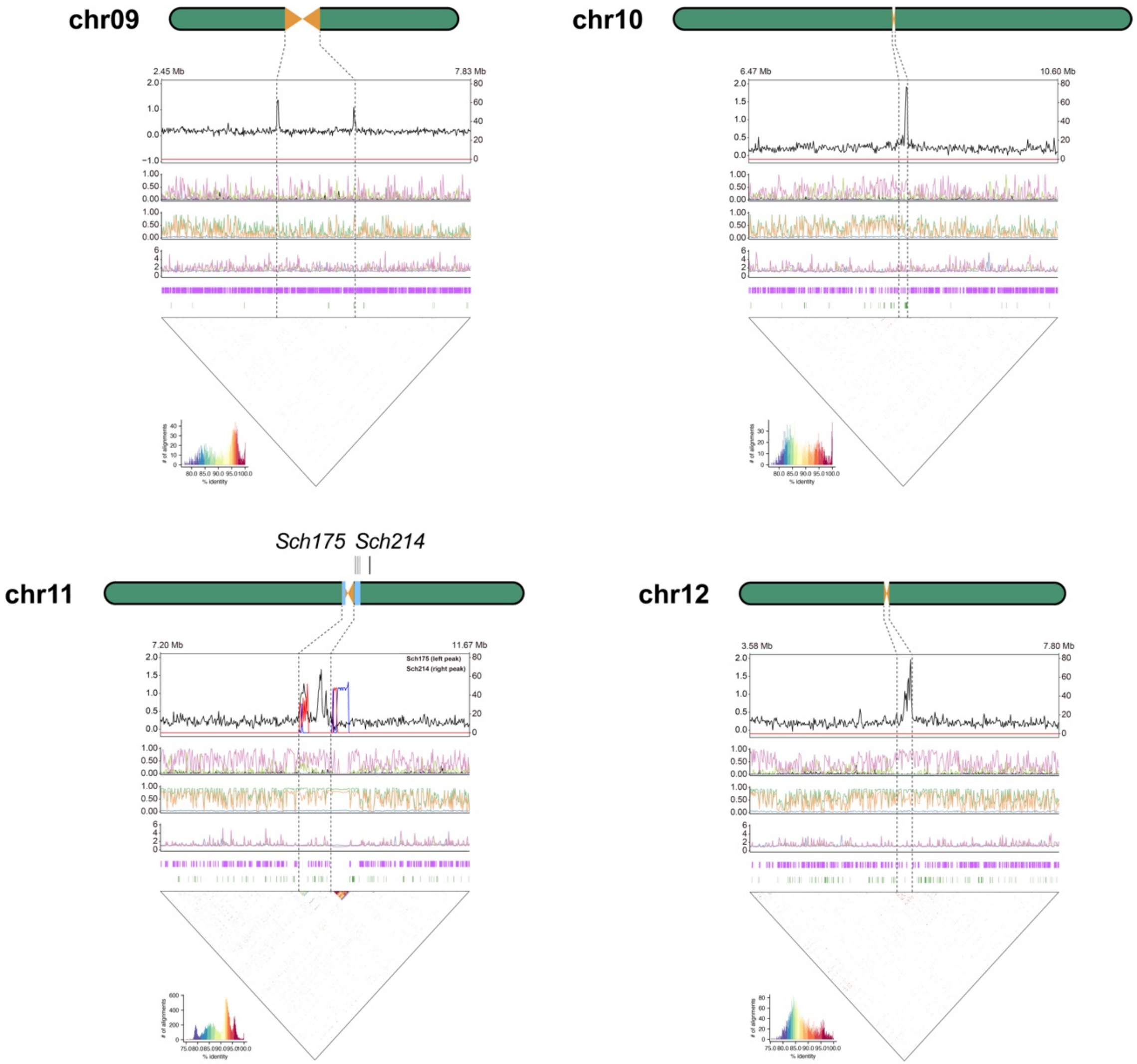
chr09
chr10
2.45 Mb
7.83 Mb
6.47 Mb
10.60 Mb
Sch175 Sch214
chr11
chr12
7.20 Mb
11.67 Mb
Sch175 (left peak)
Sch214 (right peak)
3.58 Mb
7.80 Mb
# of alignments
% identity

(continued)

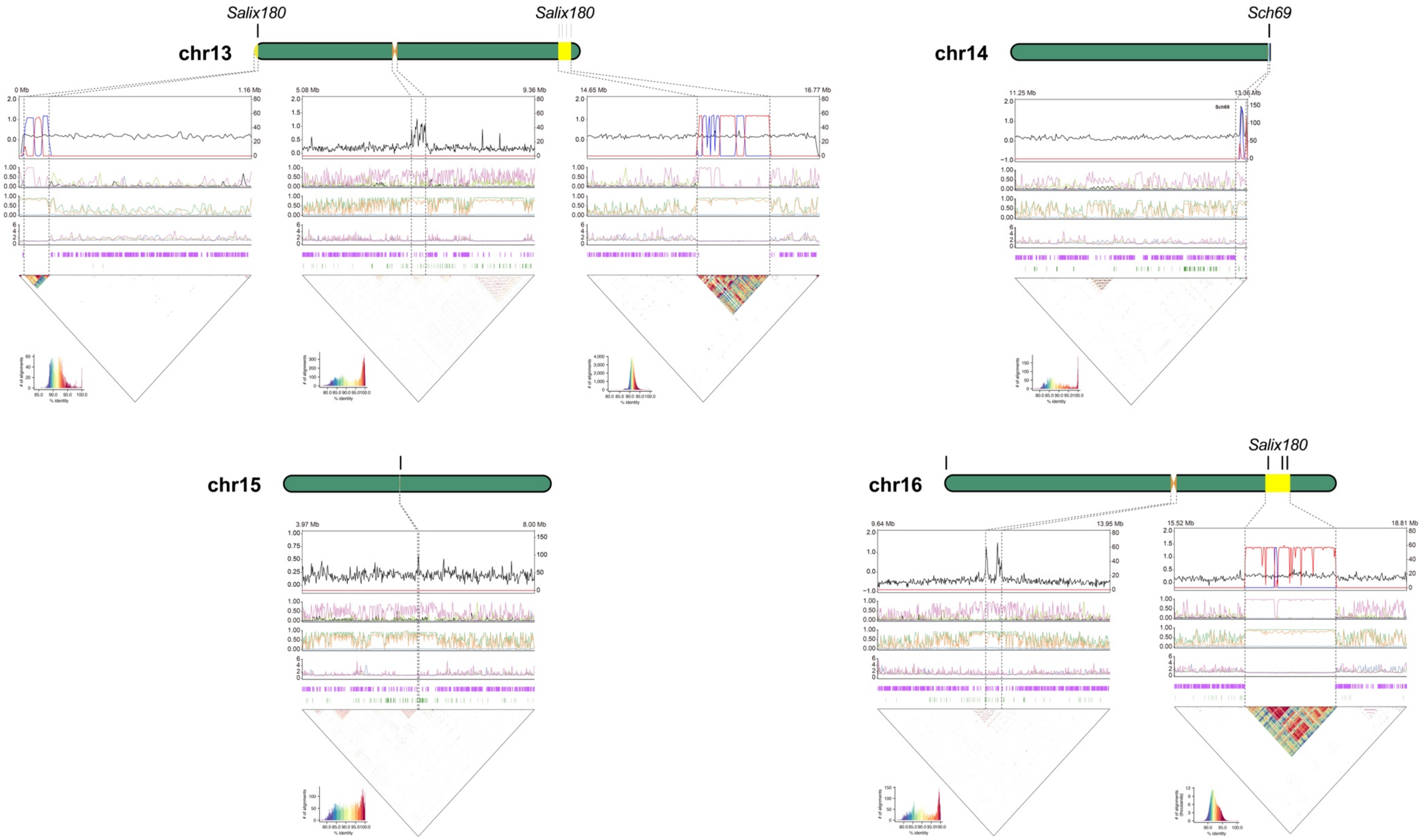

(continued)

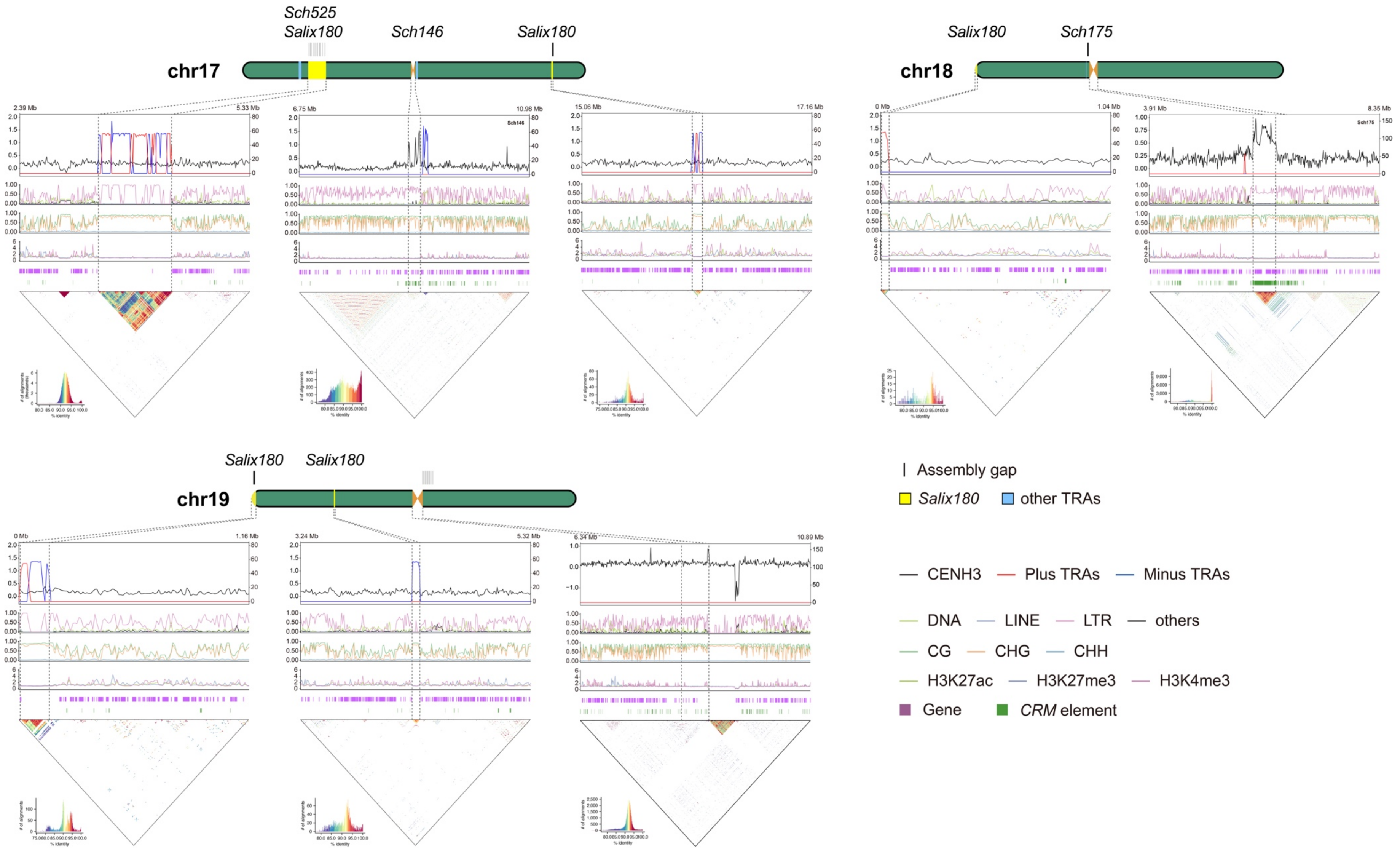
chr17
Sch525
Salix180
Sch146
Salix180
chr18
Salix180
Sch175
chr19
Salix180
Salix180
Assembly gap
Salix180
other TRAs
CENH3
Plus TRAs
Minus TRAs
DNA
LINE
LTR
others
CG
CHG
CHH
H3K27ac
H3K27me3
H3K4me3
Gene
CRM element

Fig. S11. Characteristics and epigenetics of centromeres and *Salix180* TRAs for *S. chaenomeloides* haplotype I. Plots from top to bottom separately represent assembly gaps, tandem repeats on forward (red) and reverse (blue) strands and CENH3 CUT&Tag distribution per 10-kb, transposable element distribution, DNA methylation level, histone modification level, gene distribution, *CRM* element distribution and sequence similarity on centromeres and adjacent regions. Regions marked in orange triangles represent centromeres.

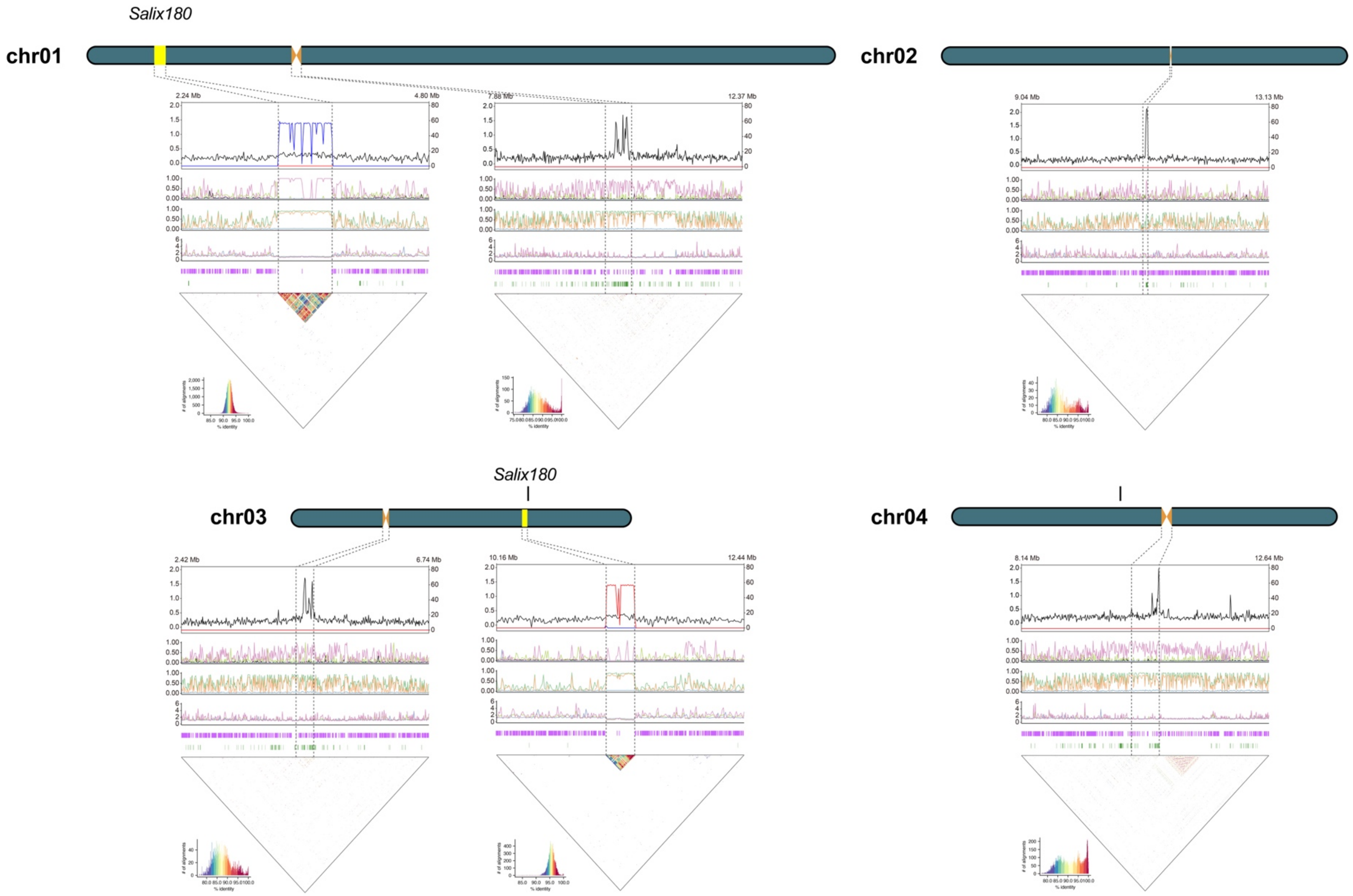

Salix180
chr01
chr02
2.24 Mb
4.80 Mb
7.88 Mb
12.37 Mb
9.04 Mb
13.13 Mb
Salix180
chr03
chr04
2.42 Mb
6.74 Mb
10.16 Mb
12.44 Mb
8.14 Mb
12.64 Mb

(continued)

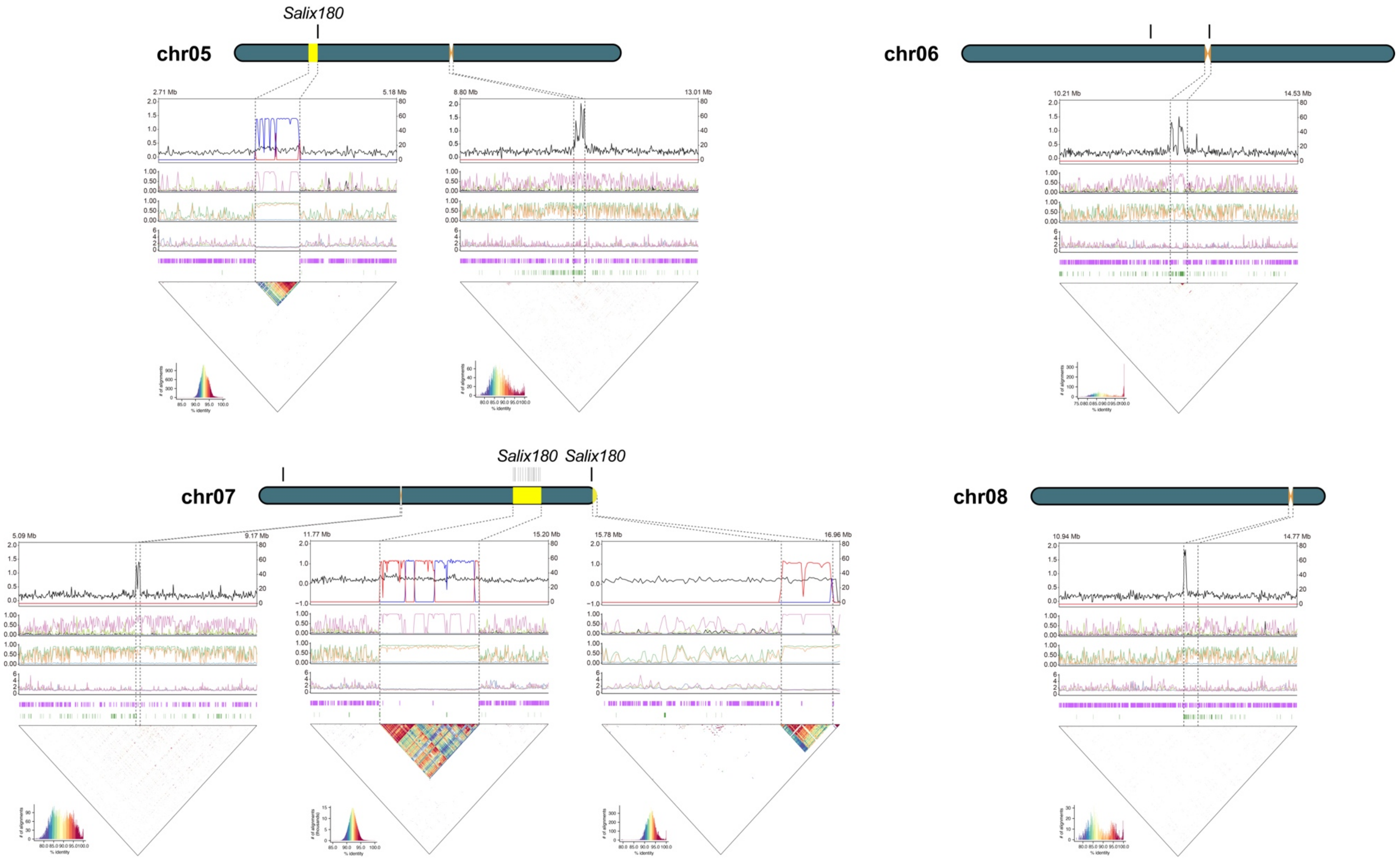
Salix180
chr05
chr06
2.71 Mb
5.18 Mb
8.80 Mb
13.01 Mb
10.21 Mb
14.53 Mb
Salix180
Salix180
chr07
chr08
5.09 Mb
9.17 Mb
11.77 Mb
15.20 Mb
15.78 Mb
16.96 Mb
10.94 Mb
14.77 Mb

(continued)

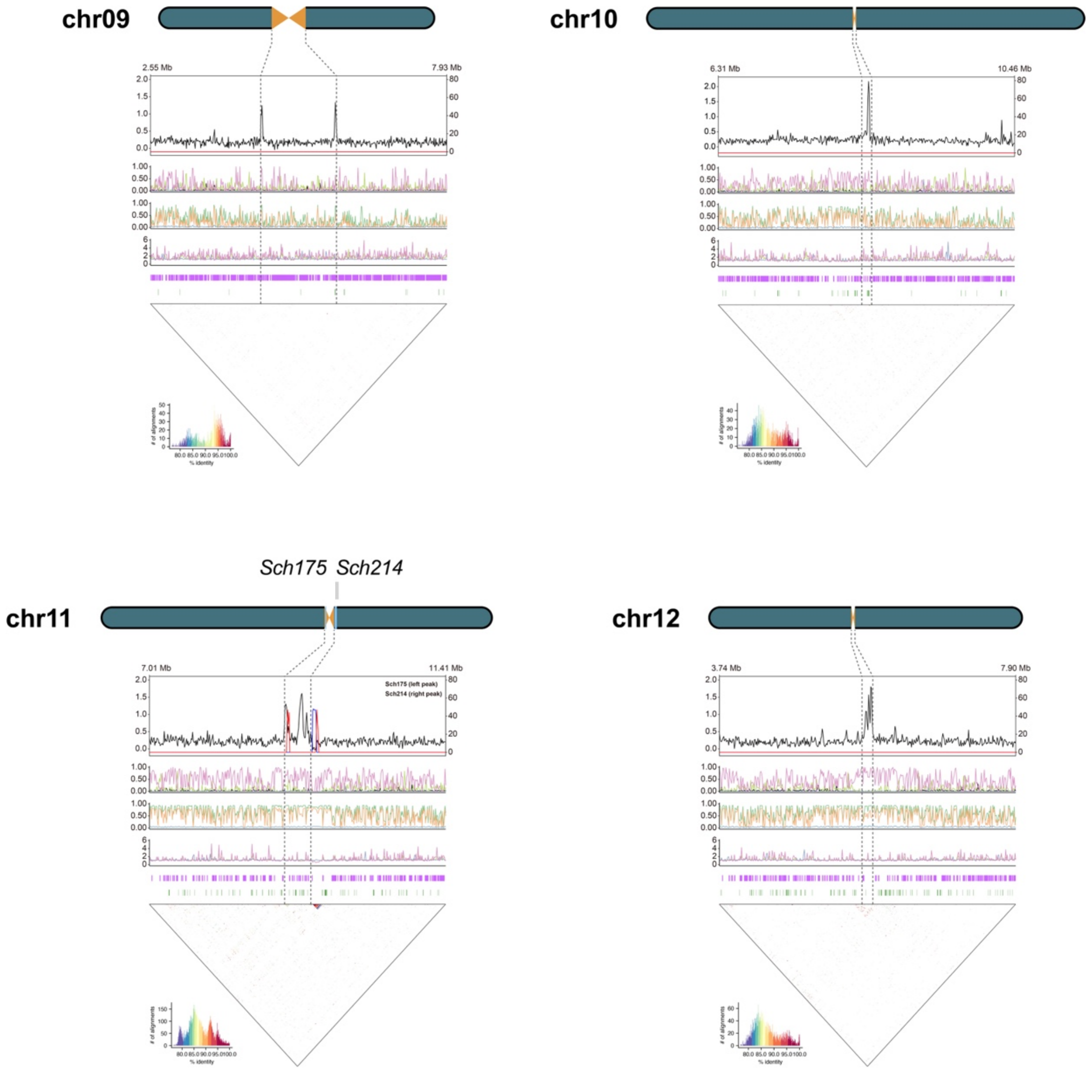
chr09
2.55 Mb
7.93 Mb
chr10
6.31 Mb
10.46 Mb
Sch175 Sch214
chr11
7.01 Mb
11.41 Mb
Sch175 (left peak)
Sch214 (right peak)
chr12
3.74 Mb
7.90 Mb
# of alignments
% identity

(continued)

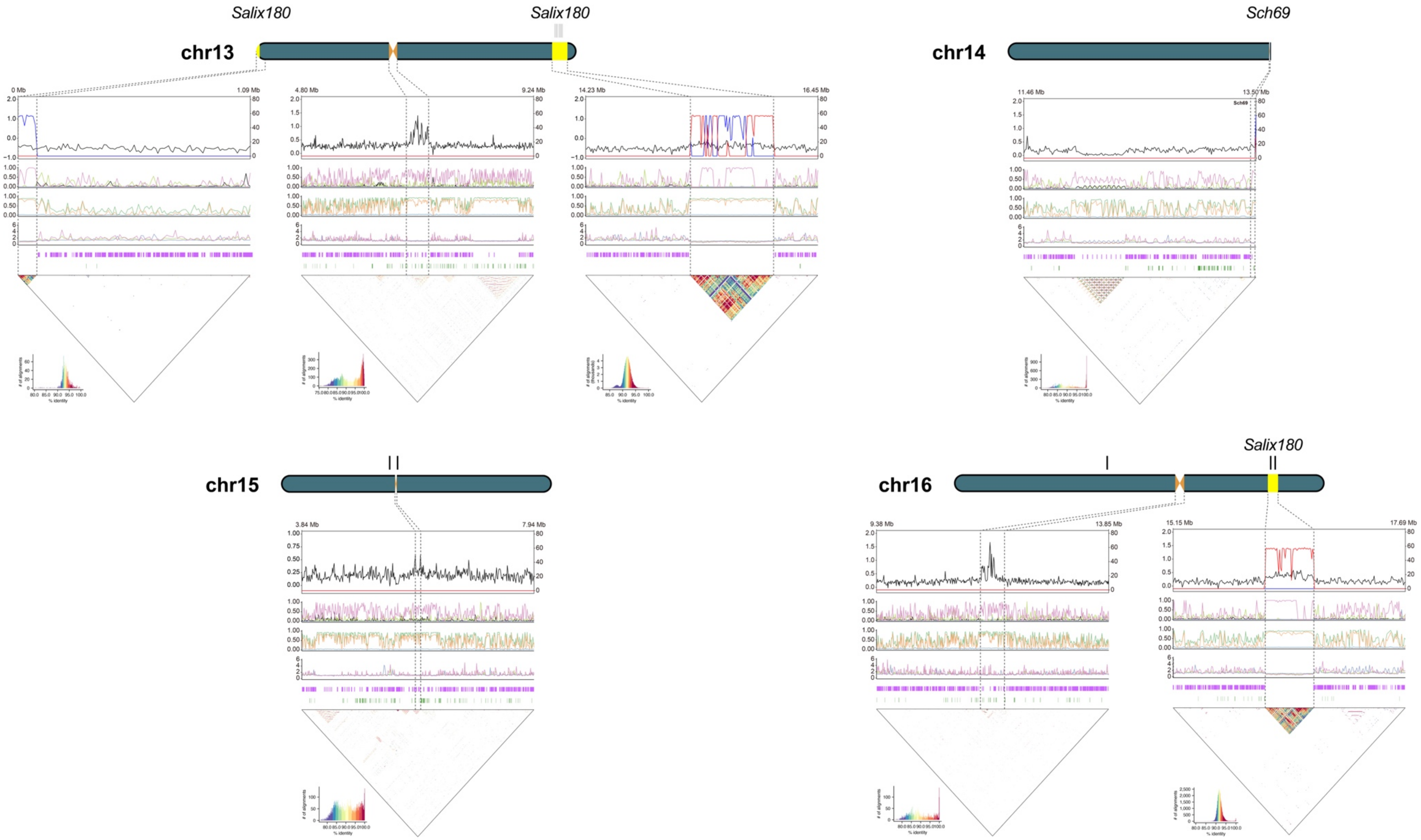
Salix180
Salix180
chr13
Sch69
chr14
0 Mb
1.09 Mb
4.80 Mb
9.24 Mb
14.23 Mb
16.45 Mb
11.46 Mb
13.50 Mb
chr15
Salix180
chr16
3.84 Mb
7.94 Mb
9.38 Mb
13.85 Mb
15.15 Mb
17.69 Mb

(continued)

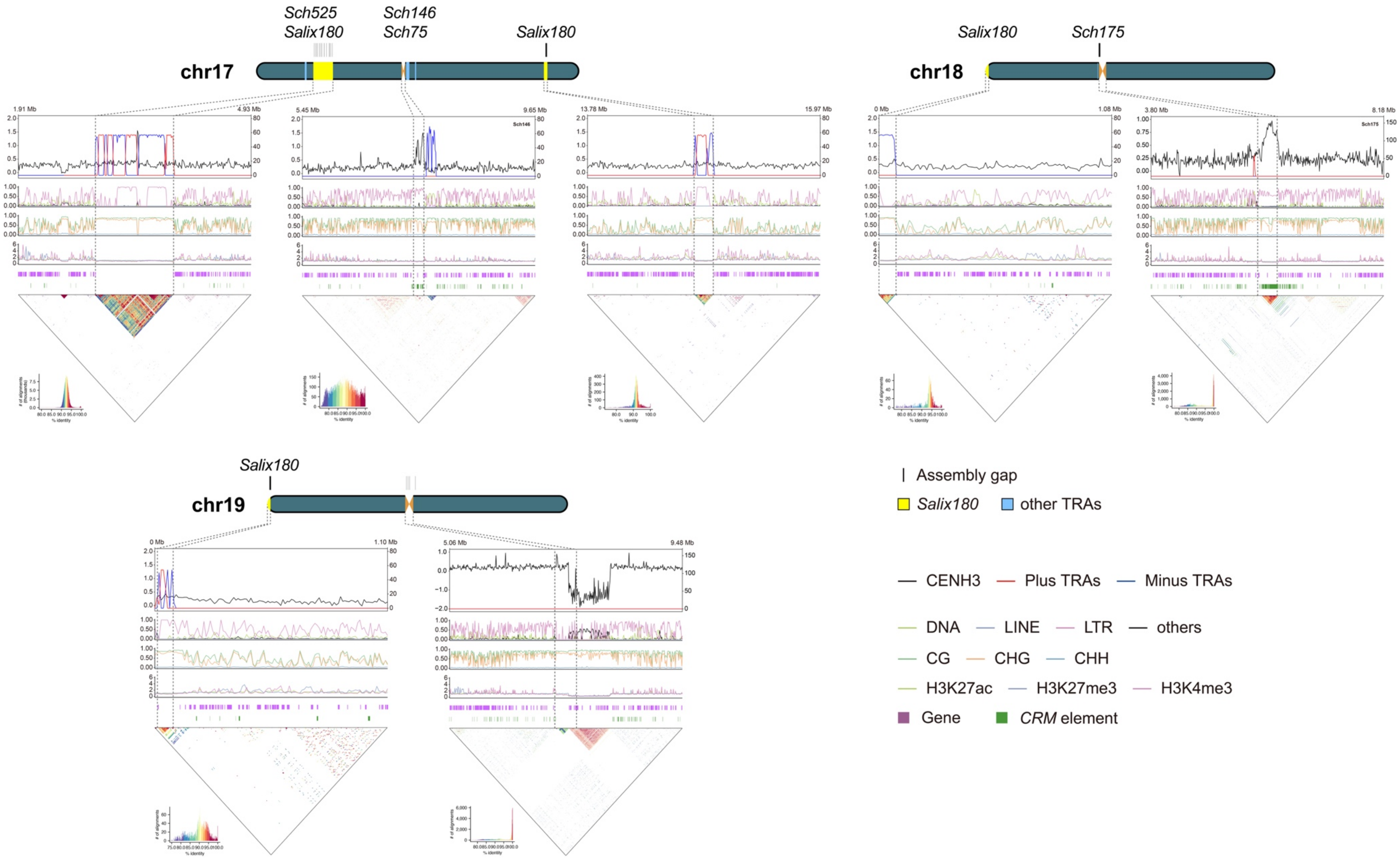

Sch525
Salix180
Sch146
Sch75
Salix180
chr17
Salix180
Sch175
chr18
Salix180
chr19
Assembly gap
Salix180
other TRAs
CENH3
Plus TRAs
Minus TRAs
DNA
LINE
LTR
others
CG
CHG
CHH
H3K27ac
H3K27me3
H3K4me3
Gene
CRM element

Fig. S12. Characteristics and epigenetics of centromeres and *Salix180* TRAs for *S. chaenomeloides* haplotype II. Plots from top to bottom separately represent assembly gaps, tandem repeats on forward (red) and reverse (blue) strands and CENH3 CUT&Tag distribution per 10-kb, transposable element distribution, DNA methylation level, histone modification level, gene distribution, *CRM* element distribution and sequence similarity on centromeres and adjacent regions. Regions marked in orange triangles represent centromeres.

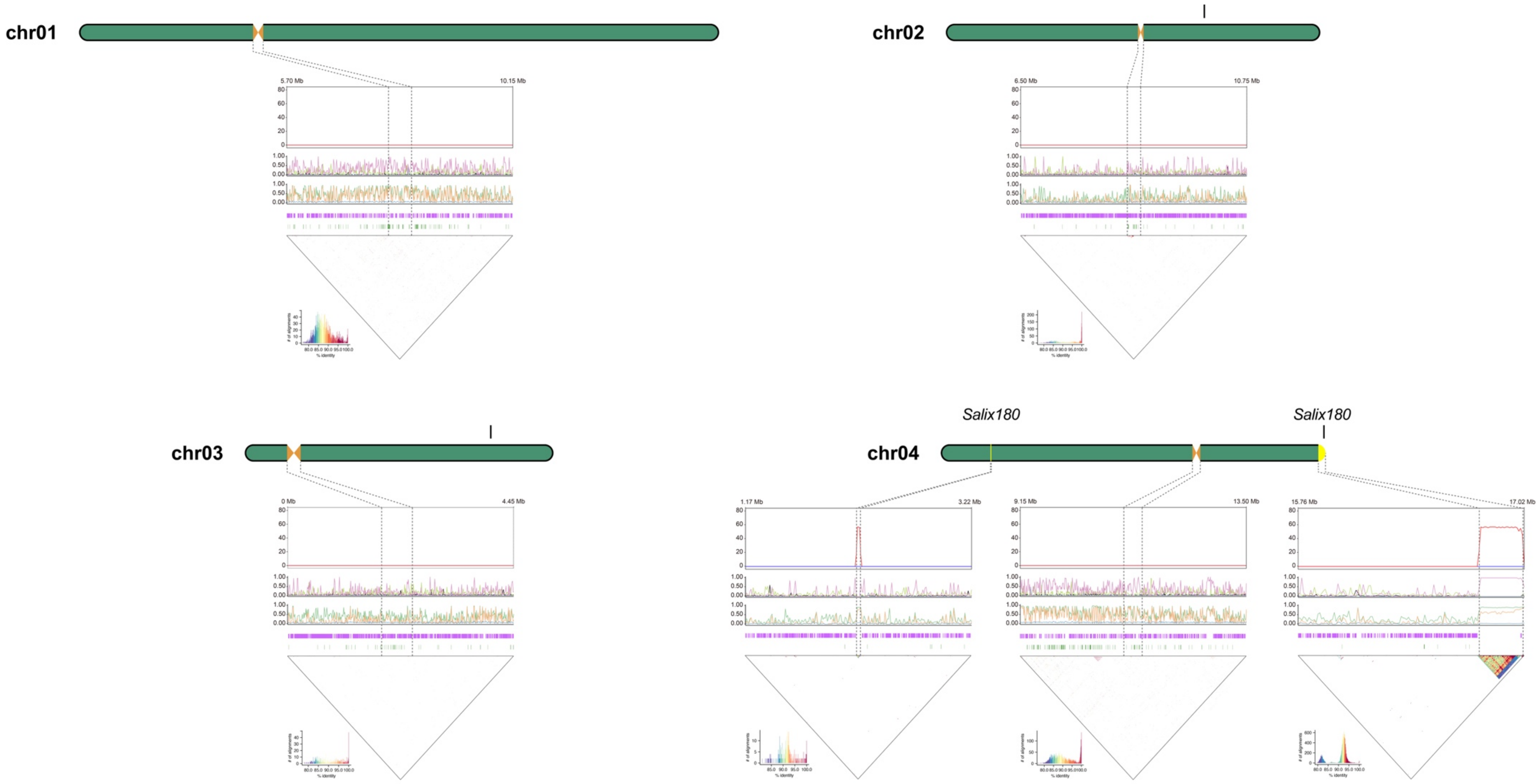
chr01
chr02
chr03
chr04
Salix180
Salix180
5.70 Mb
10.15 Mb
6.50 Mb
10.75 Mb
0 Mb
4.45 Mb
1.17 Mb
3.22 Mb
9.15 Mb
13.50 Mb
15.76 Mb
17.02 Mb

(continued)

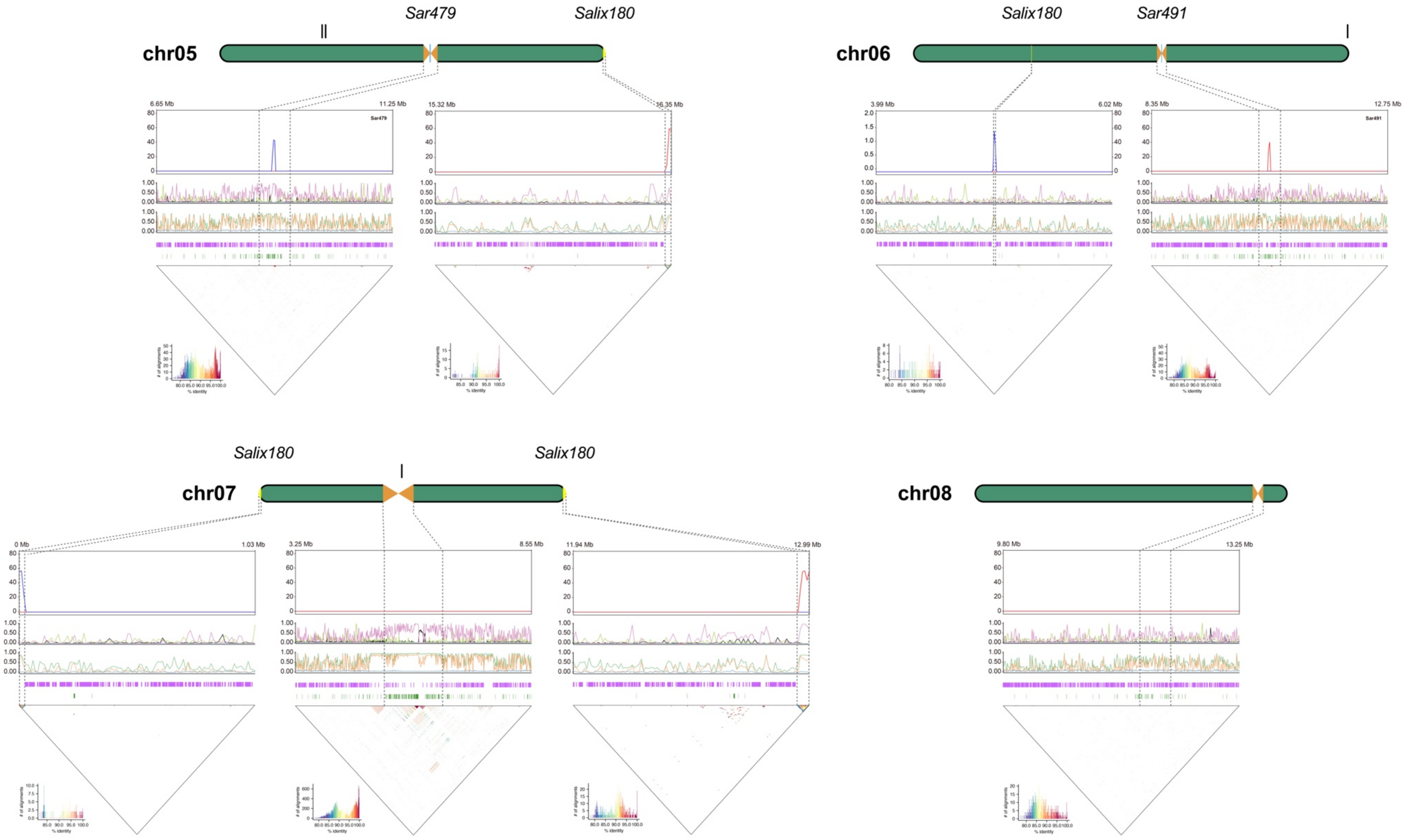
Sar479
Salix180
chr05
6.65 Mb
11.25 Mb
15.32 Mb
16.35 Mb
Salix180
Sar491
chr06
3.99 Mb
6.02 Mb
8.35 Mb
12.75 Mb
Salix180
Salix180
chr07
0 Mb
1.03 Mb
3.25 Mb
8.55 Mb
11.94 Mb
12.99 Mb
chr08
9.80 Mb
13.25 Mb

(continued)

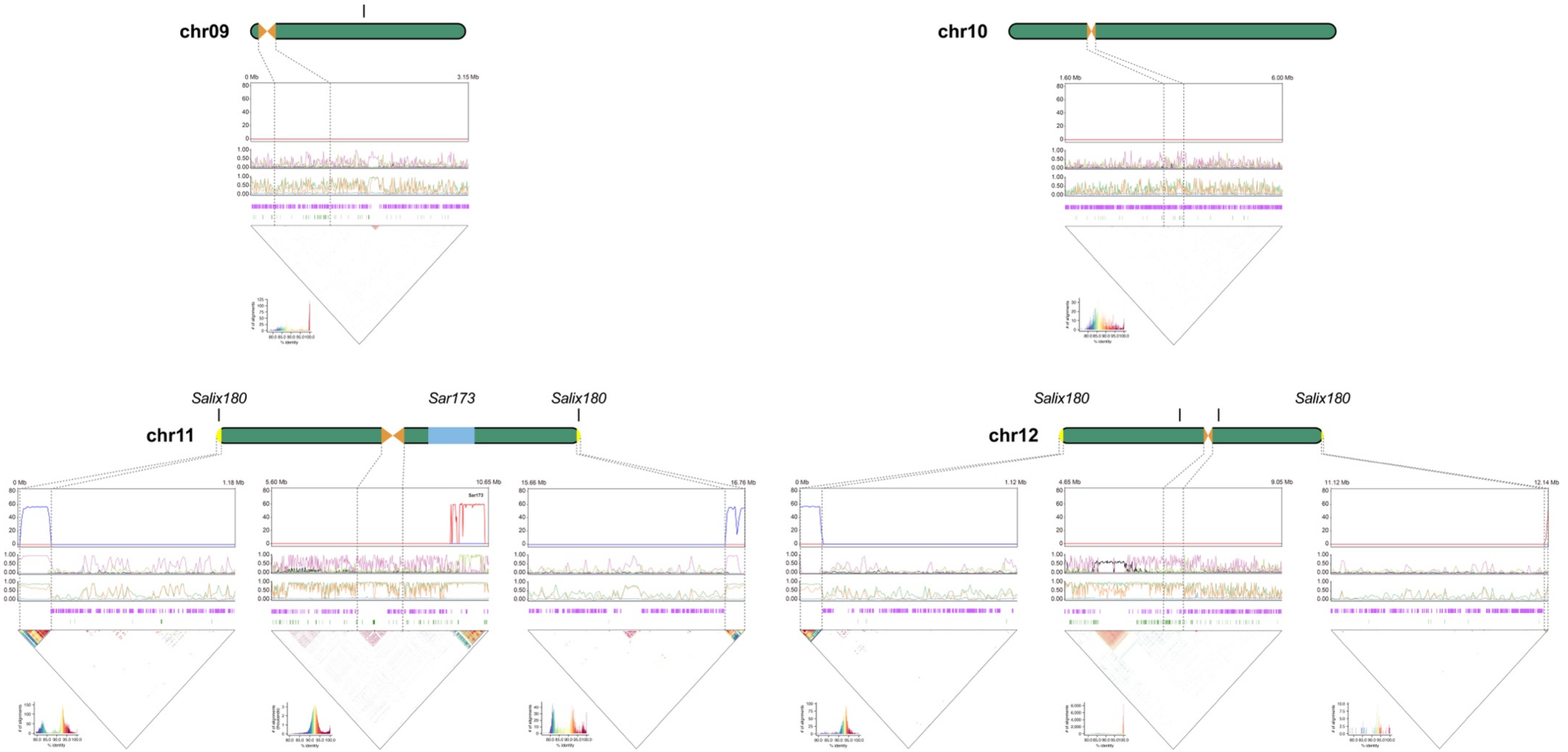
chr09
chr10
Salix180
Sar173
Salix180
chr11
Salix180
Salix180
chr12

(continued)

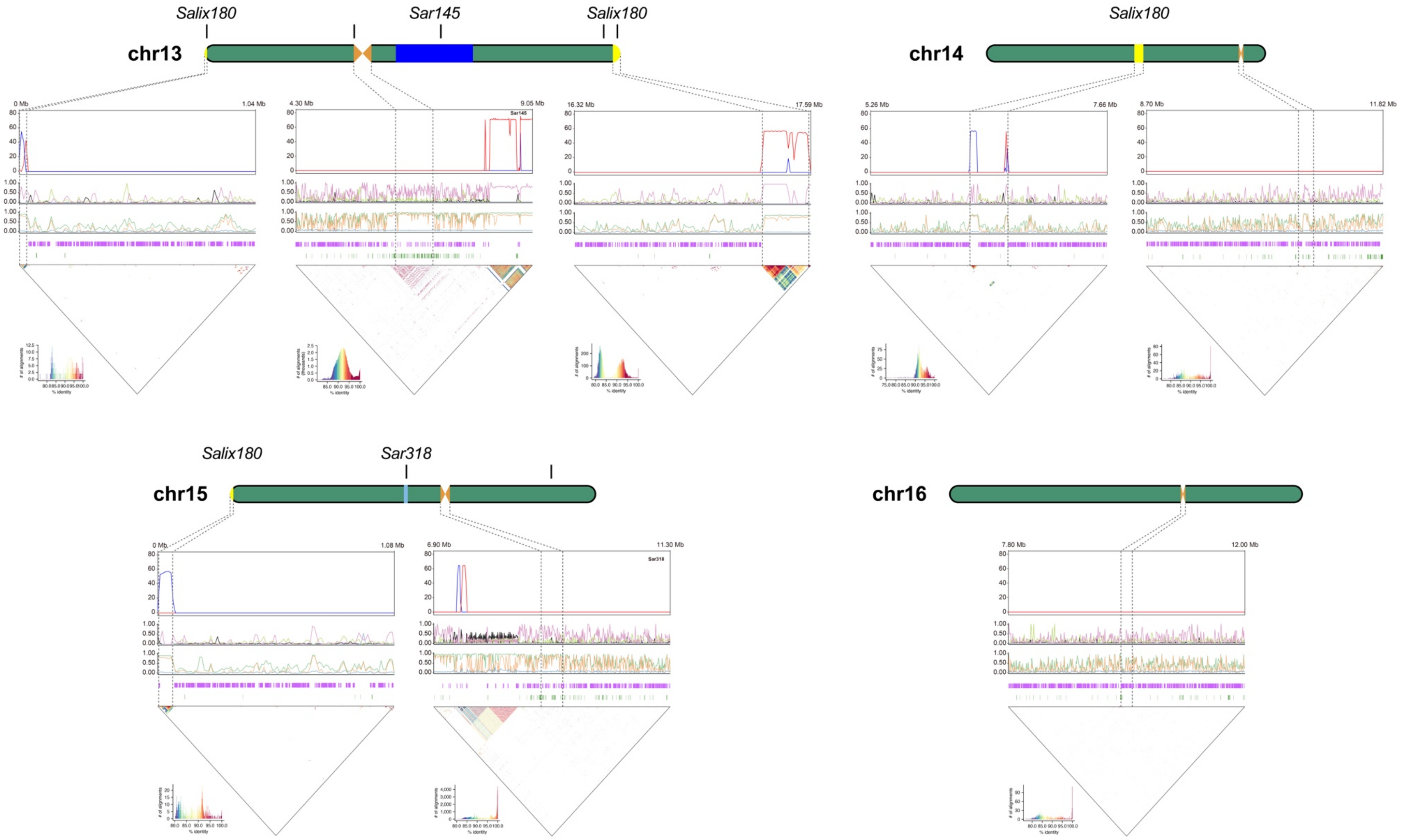
Salix180
Sar145
Salix180
chr13
Salix180
chr14
0 Mb
1.04 Mb
4.30 Mb
9.05 Mb
Sar145
16.32 Mb
17.59 Mb
5.26 Mb
7.66 Mb
8.70 Mb
11.82 Mb
Salix180
Sar318
chr15
chr16
0 Mb
1.08 Mb
6.90 Mb
11.30 Mb
Sar318
7.80 Mb
12.00 Mb

(continued)

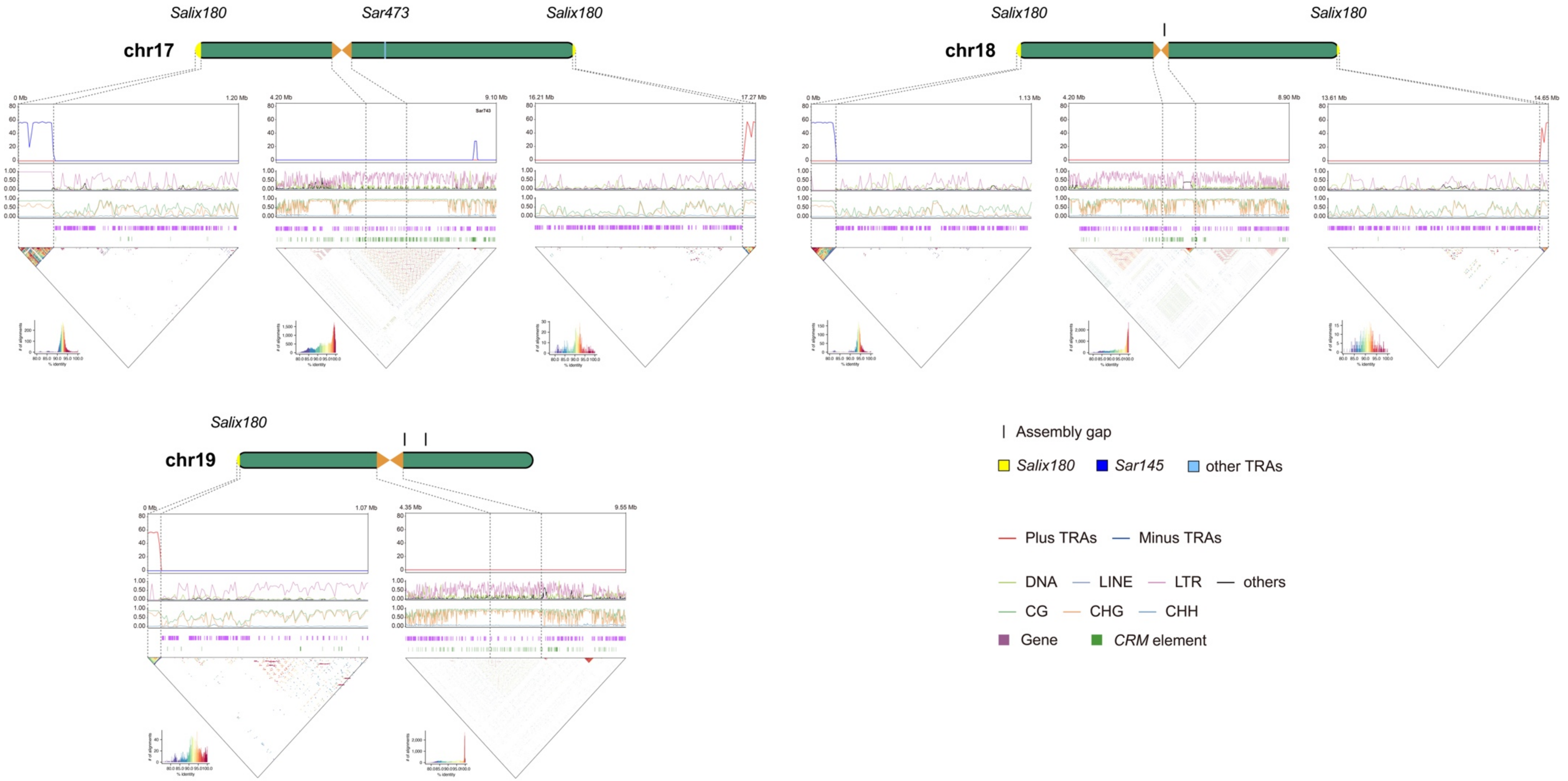

chr17
Salix180
Sar473
Salix180
chr18
Salix180
Salix180
chr19
Salix180
0 Mb
1.20 Mb
4.20 Mb
9.10 Mb
Sar743
16.21 Mb
17.27 Mb
0 Mb
1.13 Mb
4.20 Mb
8.90 Mb
13.61 Mb
14.65 Mb
0 Mb
1.07 Mb
4.35 Mb
9.55 Mb
% identity
# of alignments
Assembly gap
Salix180
Sar145
other TRAs
Plus TRAs
Minus TRAs
DNA
LINE
LTR
others
CG
CHG
CHH
Gene
CRM element

Fig. S13. Characteristics and epigenetics of centromeres and *Salix180* TRAs of *S. arbutifolia* haplotype I. Plots from top to bottom separately represent assembly gaps, tandem repeats on forward (red) and reverse (blue) strands per 10-kb, transposable element distribution, DNA methylation level, gene distribution, *CRM* element distribution and sequence similarity on centromeres and adjacent regions. Regions marked in orange triangles represent putative centromeres.

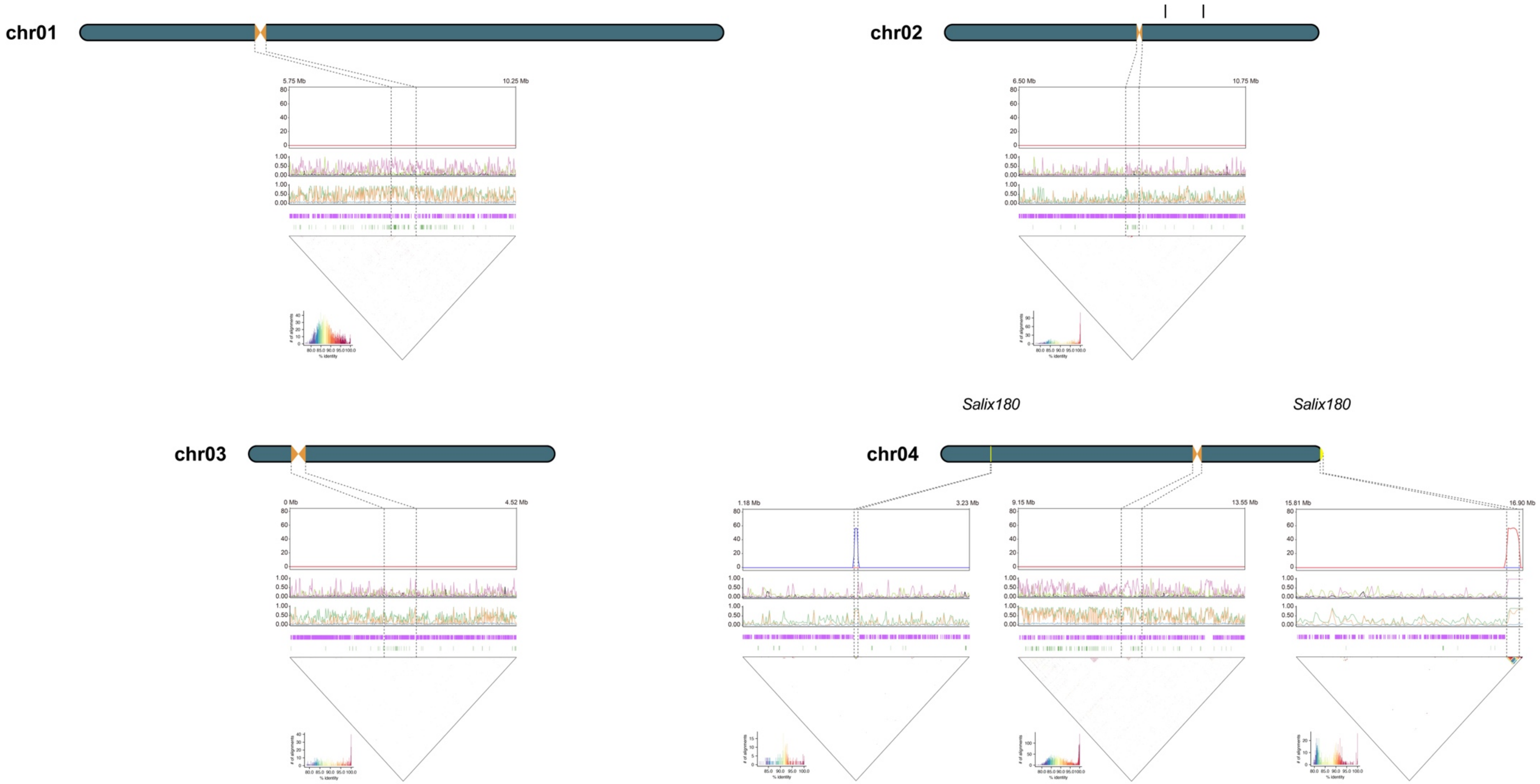
chr01
5.75 Mb
10.25 Mb
chr02
6.50 Mb
10.75 Mb
Salix180
Salix180
chr03
0 Mb
4.52 Mb
chr04
1.18 Mb
3.23 Mb
9.15 Mb
13.55 Mb
15.81 Mb
16.90 Mb

(continued)

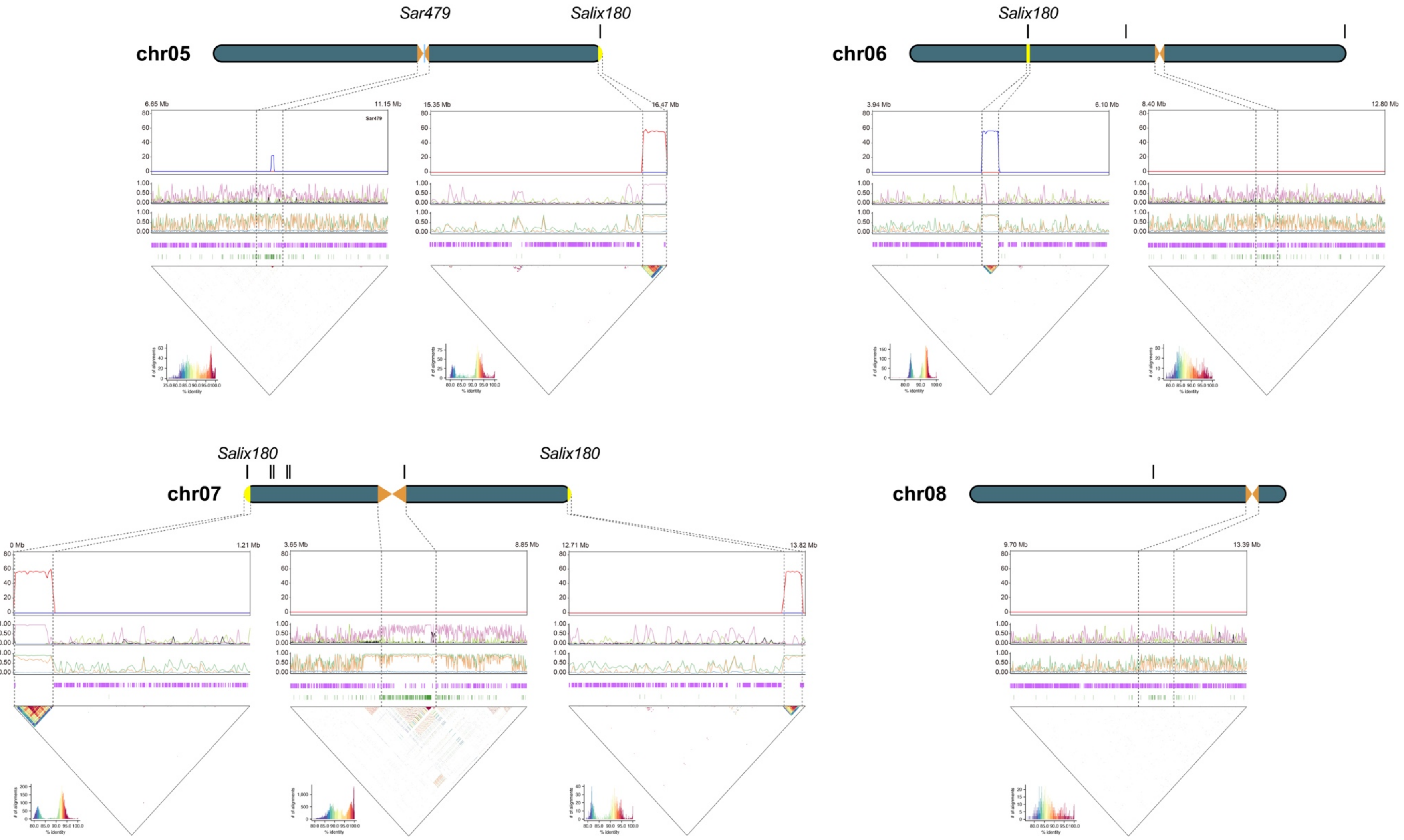

Sar479
Salix180
chr05
Salix180
chr06
Salix180
Salix180
chr07
chr08

(continued)

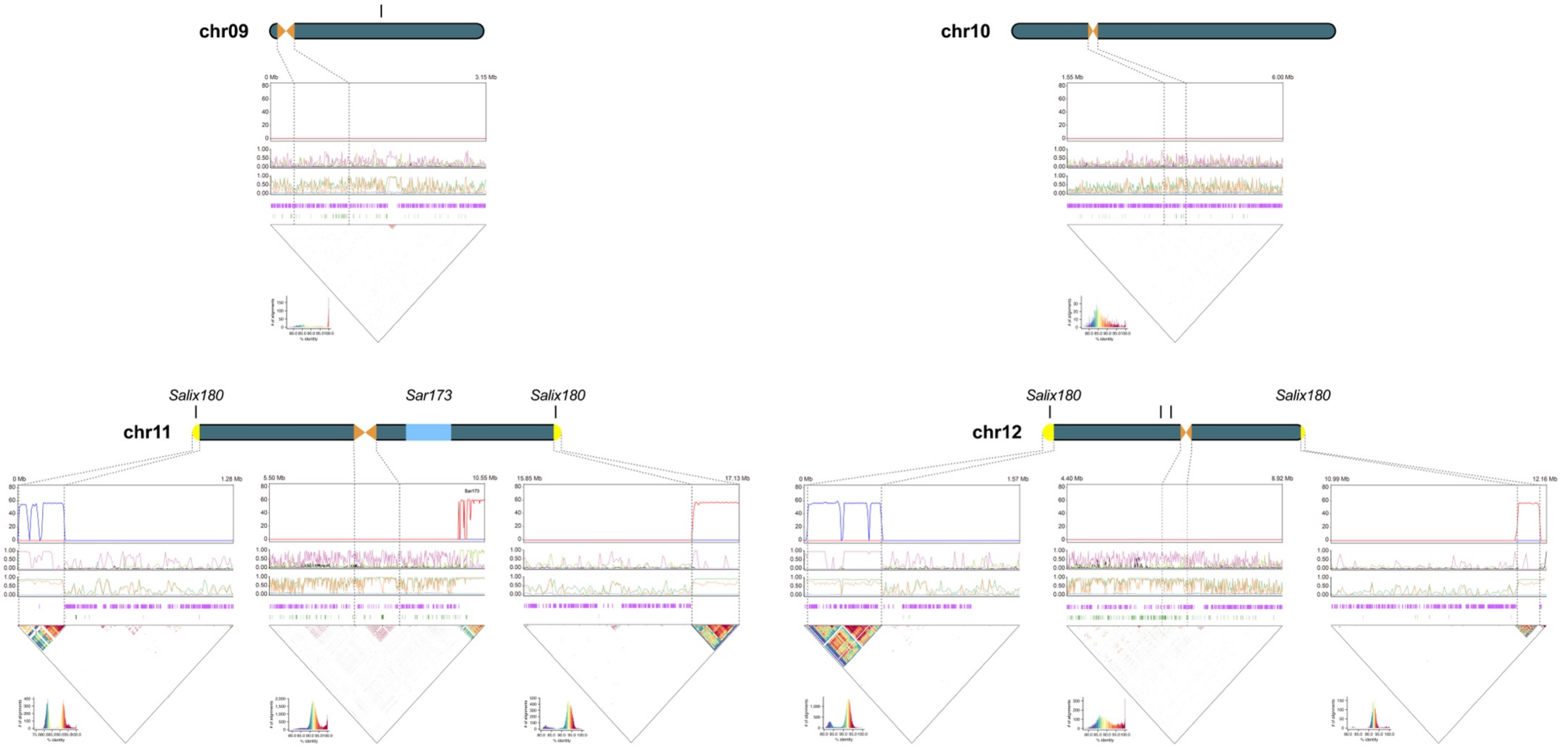
chr09
0 Mb
3.15 Mb
chr10
1.55 Mb
6.00 Mb
Salix180
Sar173
Salix180
chr11
0 Mb
1.28 Mb
5.50 Mb
10.55 Mb
Sar173
15.85 Mb
17.13 Mb
Salix180
Salix180
chr12
0 Mb
1.57 Mb
4.40 Mb
8.92 Mb
10.99 Mb
12.16 Mb

(continued)

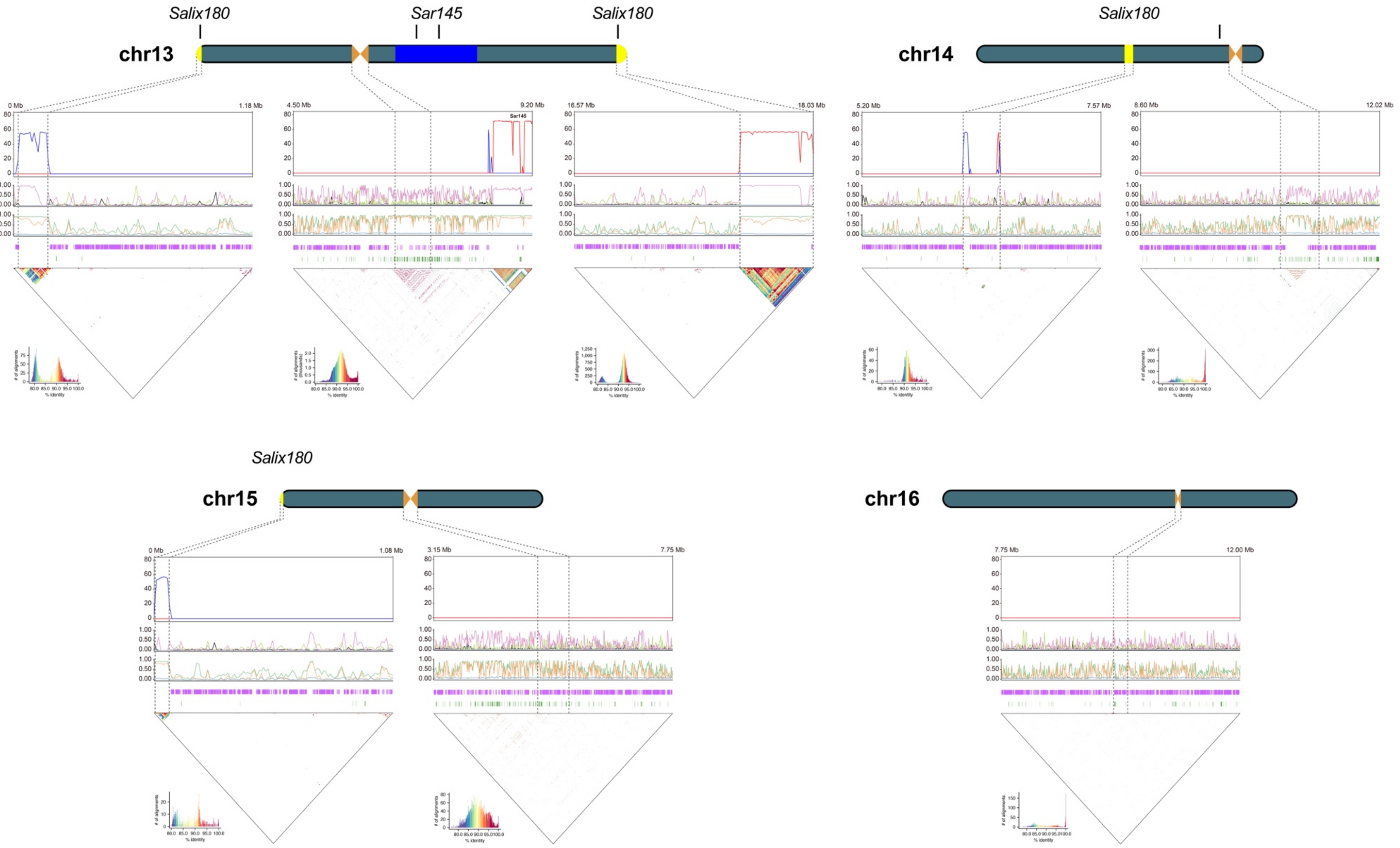
Salix180
Sar145
Salix180
chr13
Salix180
chr14
0 Mb
1.18 Mb
4.50 Mb
9.20 Mb
Sar145
16.57 Mb
18.03 Mb
5.20 Mb
7.57 Mb
8.60 Mb
12.02 Mb
Salix180
chr15
chr16
0 Mb
1.08 Mb
3.15 Mb
7.75 Mb
7.75 Mb
12.00 Mb
% identity

(continued)

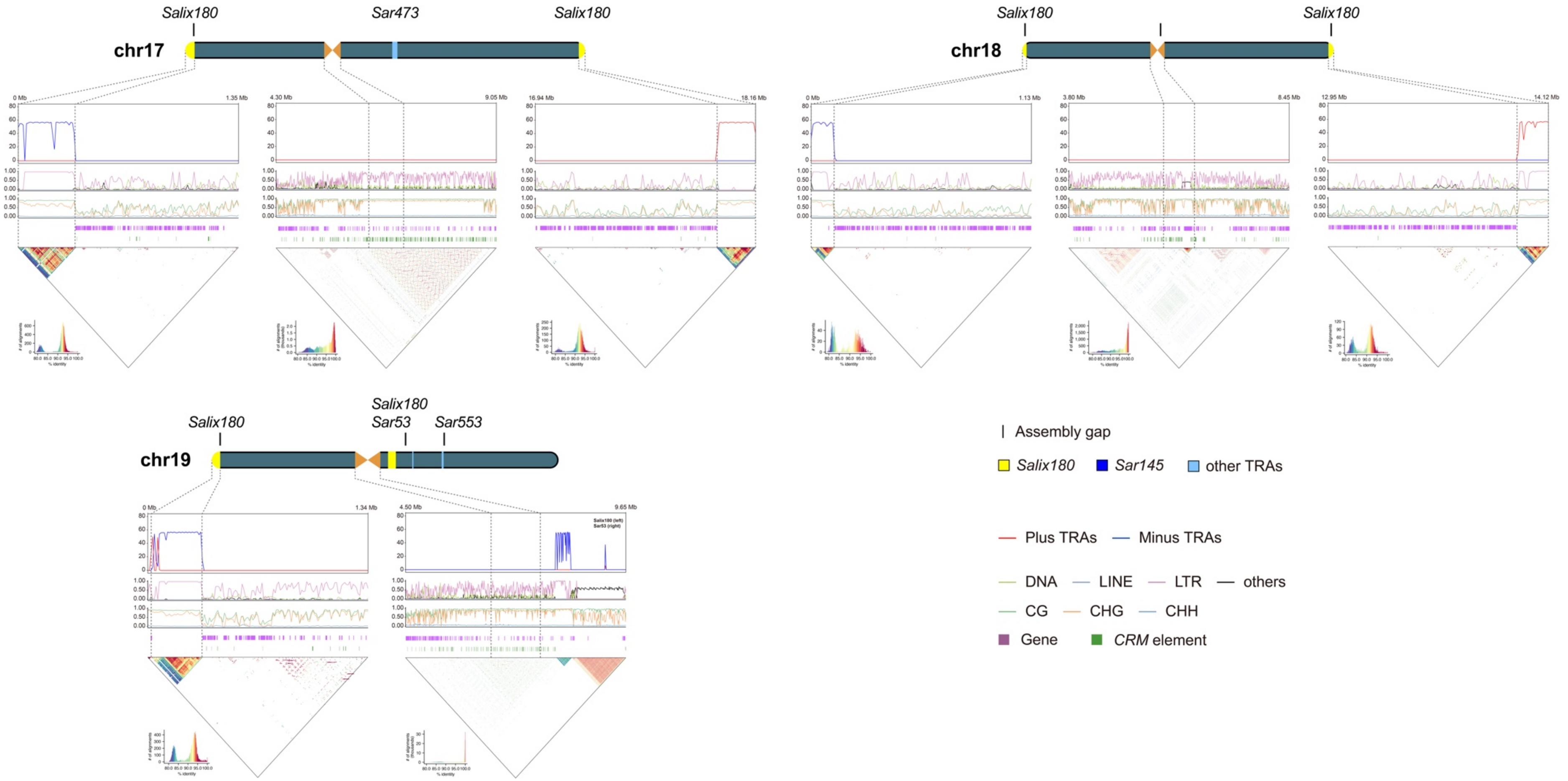

Fig. S14. Characteristics and epigenetics of centromeres and *Salix180* TRAs of *S. arbutifolia* haplotype II. Plots from top to bottom separately represent assembly gaps, tandem repeats on forward (red) and reverse (blue) strands per 10-kb, transposable element distribution, DNA methylation

level, gene distribution, *CRM* element distribution and sequence similarity on centromeres and adjacent regions. Regions marked in orange triangles represent putative centromeres.

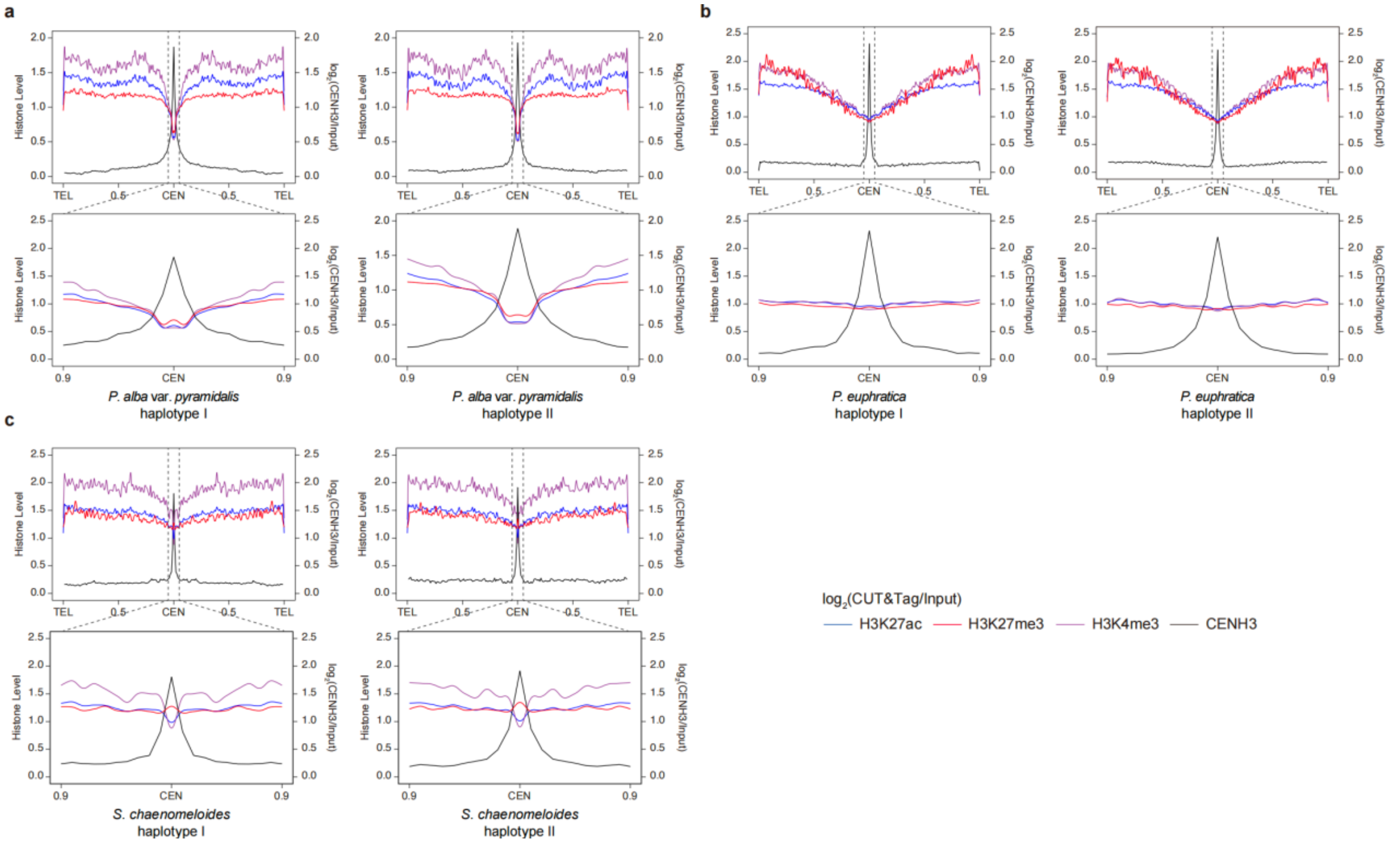
a
b
c
Histone Level
log2(CENH3/Input)
TEL
0.5
CEN
0.9
P. alba var. pyramidalis
haplotype I
haplotype II
P. euphratica
S. chaenomeloides
log2(CUT&Tag/Input)
H3K27ac
H3K27me3
H3K4me3
CENH3

Fig. S15. Histone modification features of *P. alba* var. *pyramidalis* (a), *P. euphratica* (b) and *S. chaenomeloides* (c). The upper figure represents epigenetic features plotted along chromosome arms that are proportionally scaled between telomeres (TEL) and centromere midpoints (CEN) [defined by maximum CENH3 log_2(CUT&Tag/Input) enrichment]. The x axis on the right represents the CENH3 enrichment level. The lower figure represents closed up plots of centromeres.

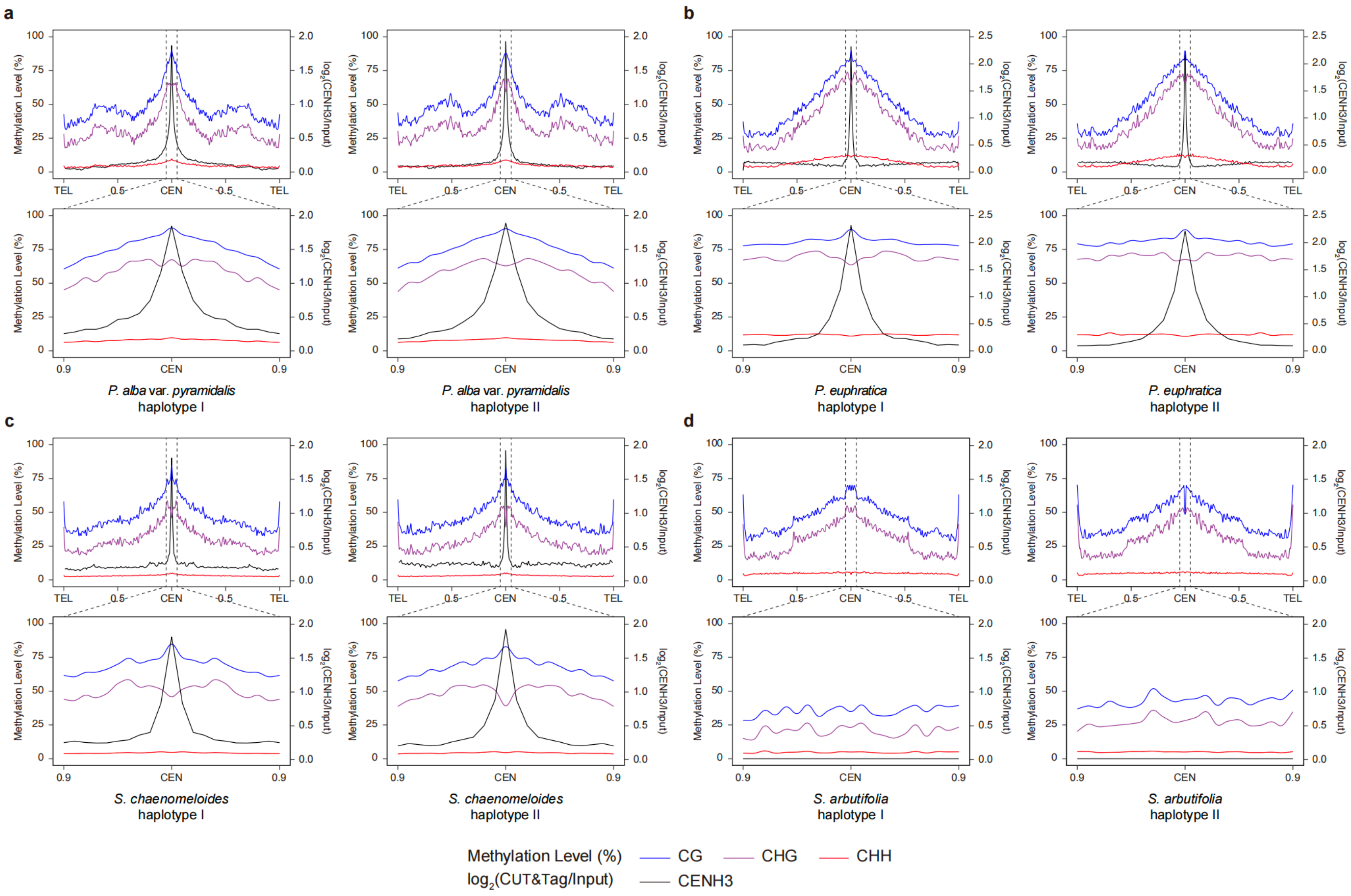
a
b
c
d
Methylation Level (%)
log2(CENH3/Input)
TEL
0.5
CEN
0.9
P. alba var. pyramidalis
haplotype I
haplotype II
P. euphratica
S. chaenomeloides
S. arbutifolia
Methylation Level (%)
CG
CHG
CHH
log2(CUT&Tag/Input)
CENH3

Fig. S16. WGBS methylation features of *P. alba* var. *pyramidalis* (a), *P. euphratica* (b), *S. chaenomeloides* (c) and *S. arbutifolia* (d). The upper figure represents methylation features plotted along chromosome arms that are proportionally scaled between telomeres (TEL) and centromere midpoints (CEN) [defined by maximum CENH3 log_2(CUT&Tag/Input) enrichment]. The x axis on the right represents the CENH3 enrichment level. The lower figure represents closed up plots of centromeres.

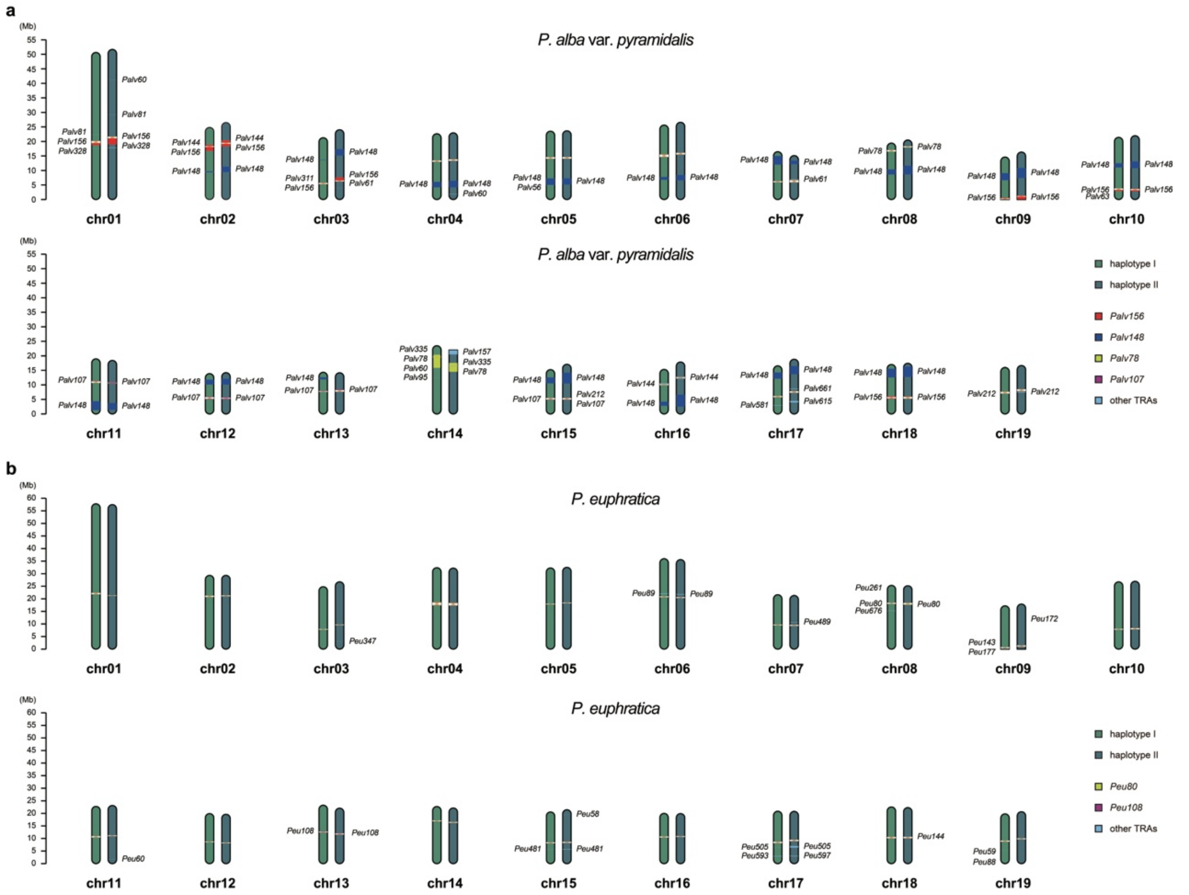

Fig. S17. TRA distribution of *P. alba* var. *pyramidalis* (a) and *P. euphratica* (b). Chromosomes are scaled by their actual length. Orange triangles represent centromeres.

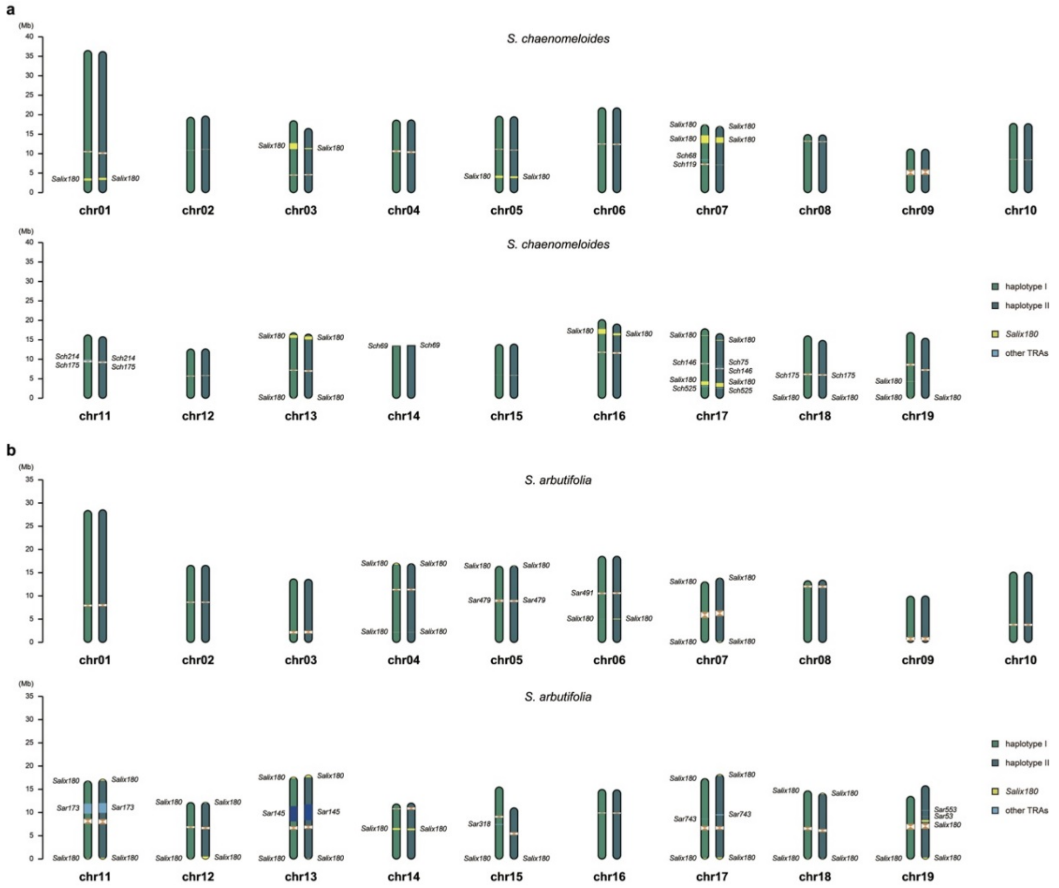

Fig. S18. TRA distribution of *S. chaenomeloides* (a) and *S. arbutifolia* (b). Chromosomes are scaled by their actual length. Orange triangles represent centromeres.

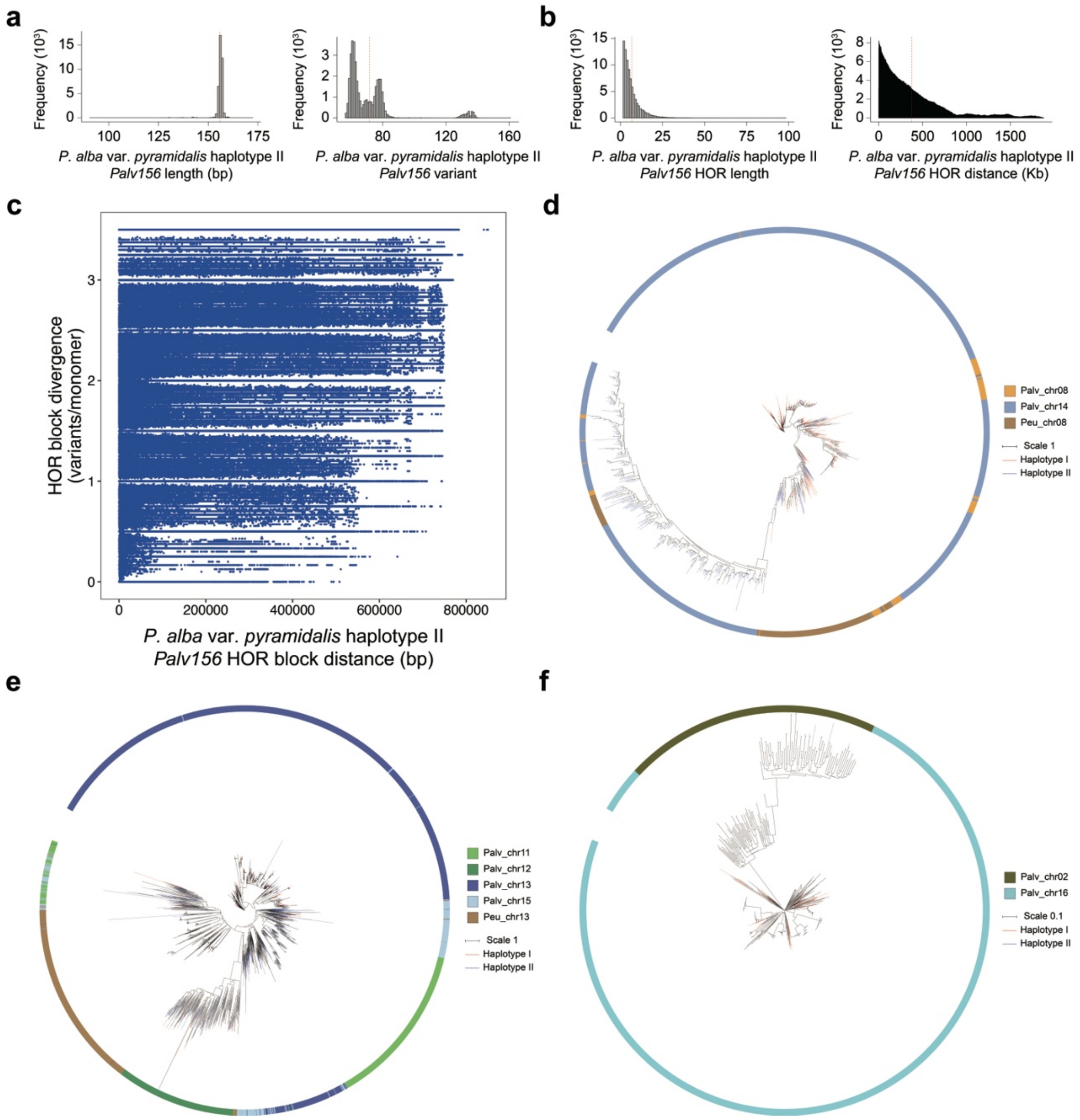

Fig. S19. Tandem repeat array analysis of *P. alba* var. *pyramidalis*. a Histograms of *Palv156* monomer lengths (left) and variant distances relative to the genome wide consensus (right) in *P. alba* var. *pyramidalis* haplotype II. **b** Histograms of monomer number (length) of *Palv156* in HOR blocks (left) and the distances between HORs (right) in *P. alba* var. *pyramidalis* haplotype II. **c** Dot plot distribution of *Palv156* HORs distance and HORs variation levels in *P. alba* var. *pyramidalis* haplotype II. **d** Phylogenetic tree of sampled *Palv78* and *Peu80* monomers. The color of the outer circle and the tree branch represented the corresponding chromosome and genome haplotype, respectively. **e** Same as for **d** but showing sampled *Palv107* and *Peu108* monomers. **f** Same as for **d** but showing *Palv144* monomers.

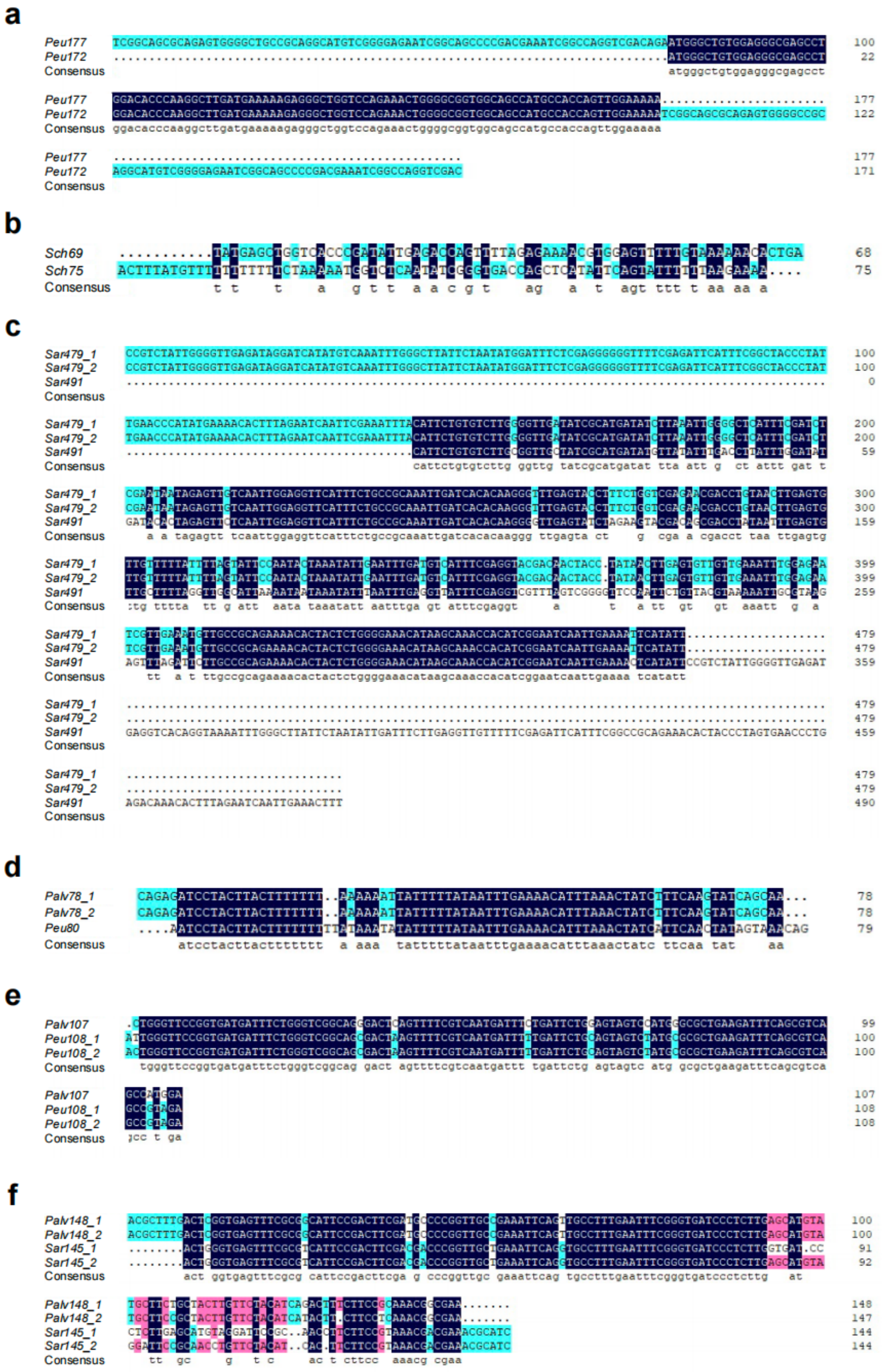

Fig. S20. Nucleic acid alignment between TRA monomers. **a** Alignments between *Peu177* monomer from *P. euphratica* haplotype I chr09 and Peu172 monomer from *P.*

euphratica haplotype II chr09. **b** Alignments between *Sch69* from *S. chaenomeloides* haplotype I chr14 and *Sch75* from *S. chaenomeloides* haplotype II chr17. **c** Alignments between *Sar479_1* monomer from *S. arbutifolia* haplotype I chr05, *Sar479_2* monomer from *S. arbutifolia* haplotype II chr05 and *Sar491* from *S. arbutifolia* haplotype I chr06. **d** Alignments between *Palv78_1* monomer from *P. alba* var. *pyramidalis* haplotype I chr14, *Palv78_2* monomer from *P. alba* var. *pyramidalis* haplotype II chr14 and *Peu80* monomer from *P. euphratica* haplotype II chr08. **e** Alignments between *Palv107* monomer from *P. alba* var. *pyramidalis* haplotype II chr15, *Peu108_1* monomer from *P. euphratica* haplotype I chr13 and *Peu108_2* monomer from *P. euphratica* haplotype II chr13. **f** Alignments between *Palv148_1* monomer from *P. alba* var. *pyramidalis* haplotype I chr11, *Palv148_2* monomer from *P. alba* var. *pyramidalis* haplotype II chr02, *Sar145_1* monomer from *S. arbutifolia* haplotype I chr13 and *Sar145_2* monomer from *S. arbutifolia* haplotype II chr13.

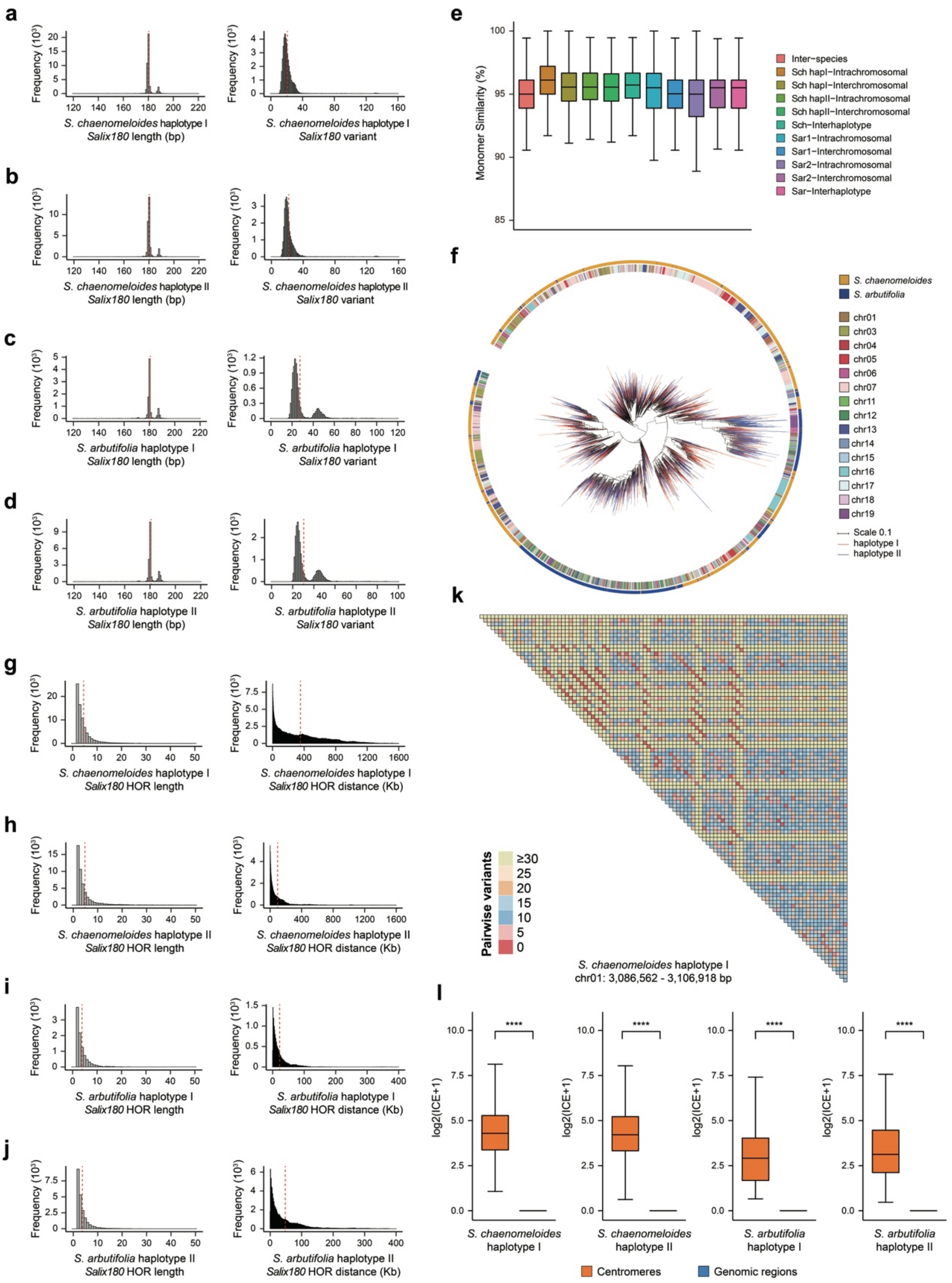

Fig. S21. Characteristics of non-centromeric *Salix180* TRAs. a to **d** Histograms of *Salix180* monomer lengths (left) and variant distances relative to the genome wide consensus (right) in *S. chaenomeloides* haplotype I, *S. chaenomeloides* haplotype II, *S. arbutifolia* haplotype I and *S. arbutifolia* haplotype II, respectively. **e** Boxplot of *Salix180* TRAs monomer similarity comparisons in both within and between

chromosomes, haplotypes and species. **f** Phylogenetic tree of sampled *Salix180* monomers. Colors of the outer circle, the inner circle and the tree branch represented the corresponding species, chromosome and genome haplotype, respectively. **g** to **j** Histograms of *Salix180* monomer number (length) in HOR blocks (left) and the distances between HORs (right) in *S. chaenomeloides* haplotype I, *S. chaenomeloides* haplotype II, *S. arbutifolia* haplotype I and *S. arbutifolia* haplotype II, respectively. **k** Representative *Salix180* TRA region heatmap colored according to pairwise variants between *Salix180* monomers. **l** Hi-C interaction strengths comparisons of *Salix180* TRAs with centromeres and non-centromeric regions, respectively (two-tailed Wilcoxon rank-sum test, ****P <= 0.0001, ***P <= 0.001, **P <= 0.01, *P <= 0.05, ns: not significant).

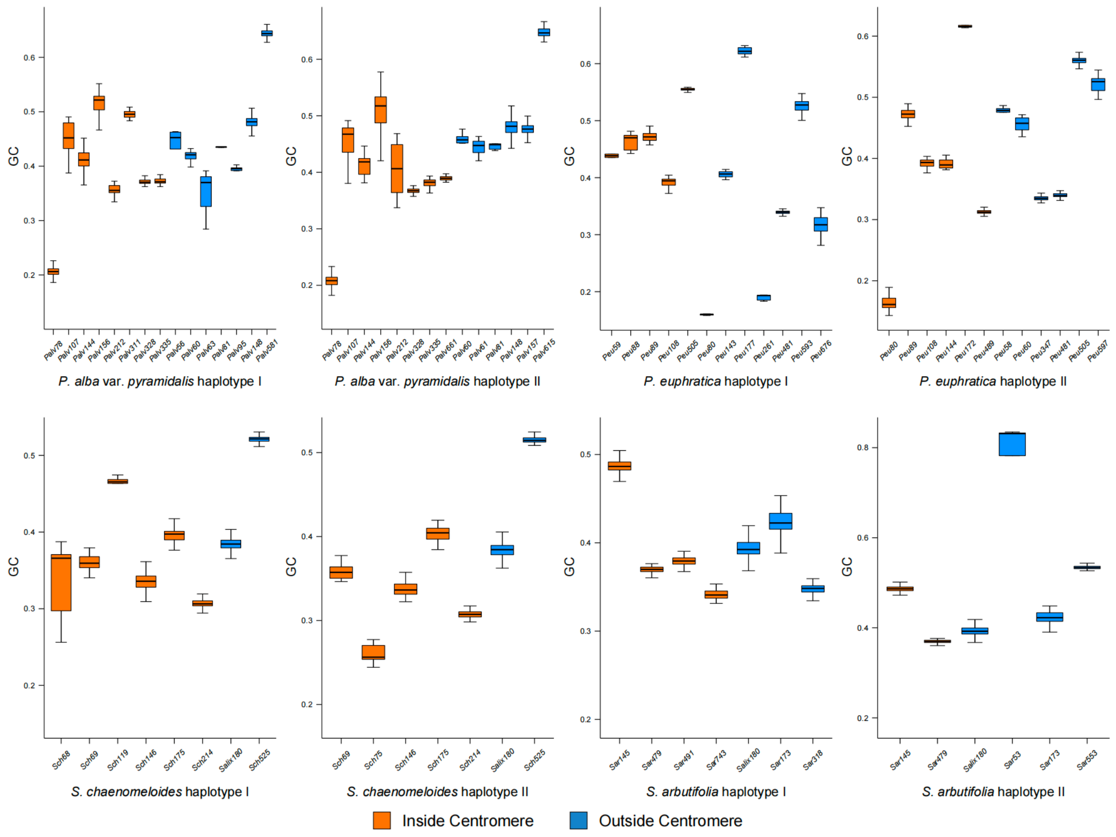

Fig. S22. Boxplots of GC content comparisons across all types of TRAs.

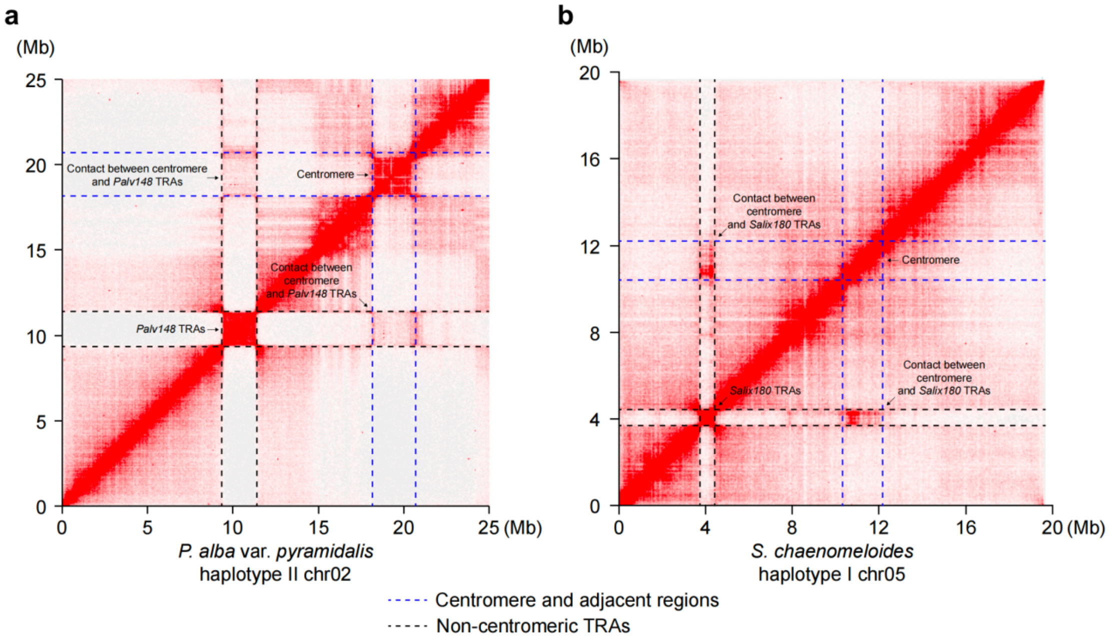

Fig. S23. Examples of Hi-C interaction visualization between centromeric regions and non-centromeric TRAs in *P. alba* var. *pyramidalis* haplotype II chr02 (a) and *S. chaenomeloides* haplotype I chr05 (b).

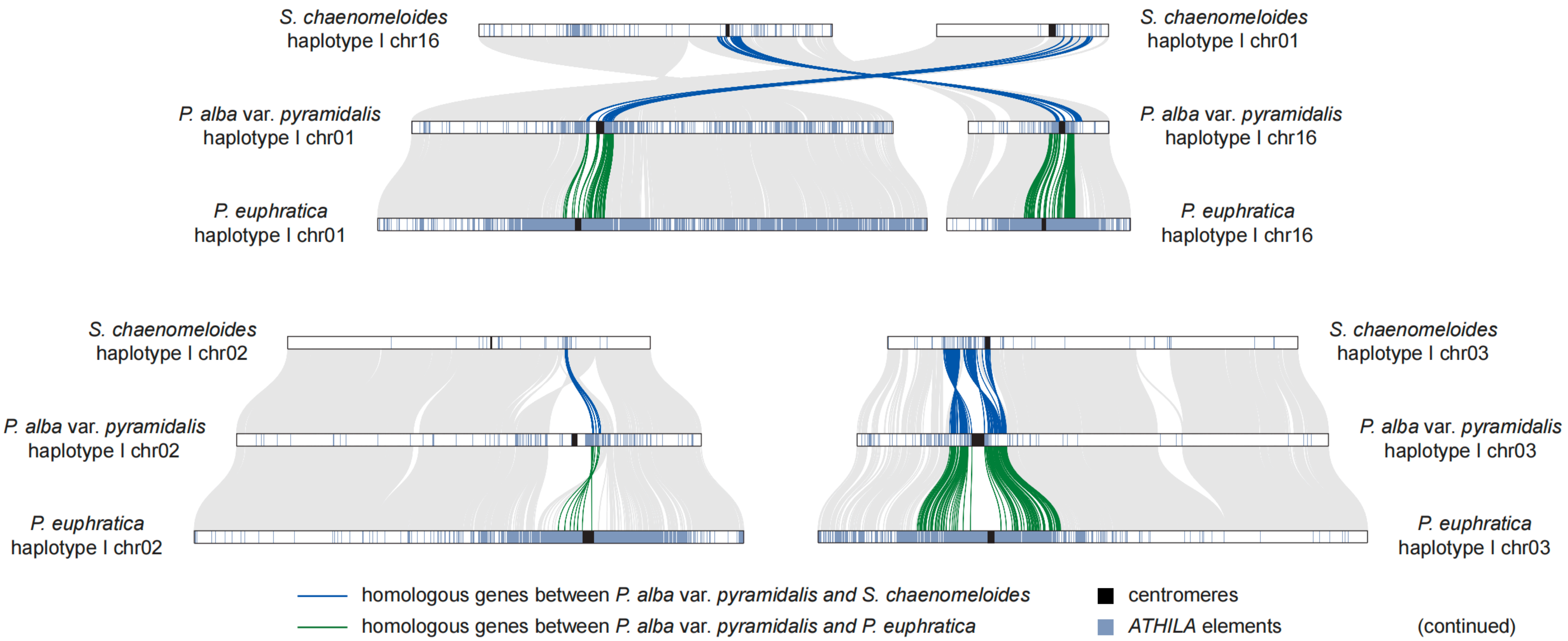
S. chaenomeloides haplotype I chr16
S. chaenomeloides haplotype I chr01
P. alba var. pyramidalis haplotype I chr01
P. alba var. pyramidalis haplotype I chr16
P. euphratica haplotype I chr01
P. euphratica haplotype I chr16
S. chaenomeloides haplotype I chr02
S. chaenomeloides haplotype I chr03
P. alba var. pyramidalis haplotype I chr02
P. alba var. pyramidalis haplotype I chr03
P. euphratica haplotype I chr02
P. euphratica haplotype I chr03
homologous genes between P. alba var. pyramidalis and S. chaenomeloides
homologous genes between P. alba var. pyramidalis and P. euphratica
centromeres
ATHILA elements

(continued)

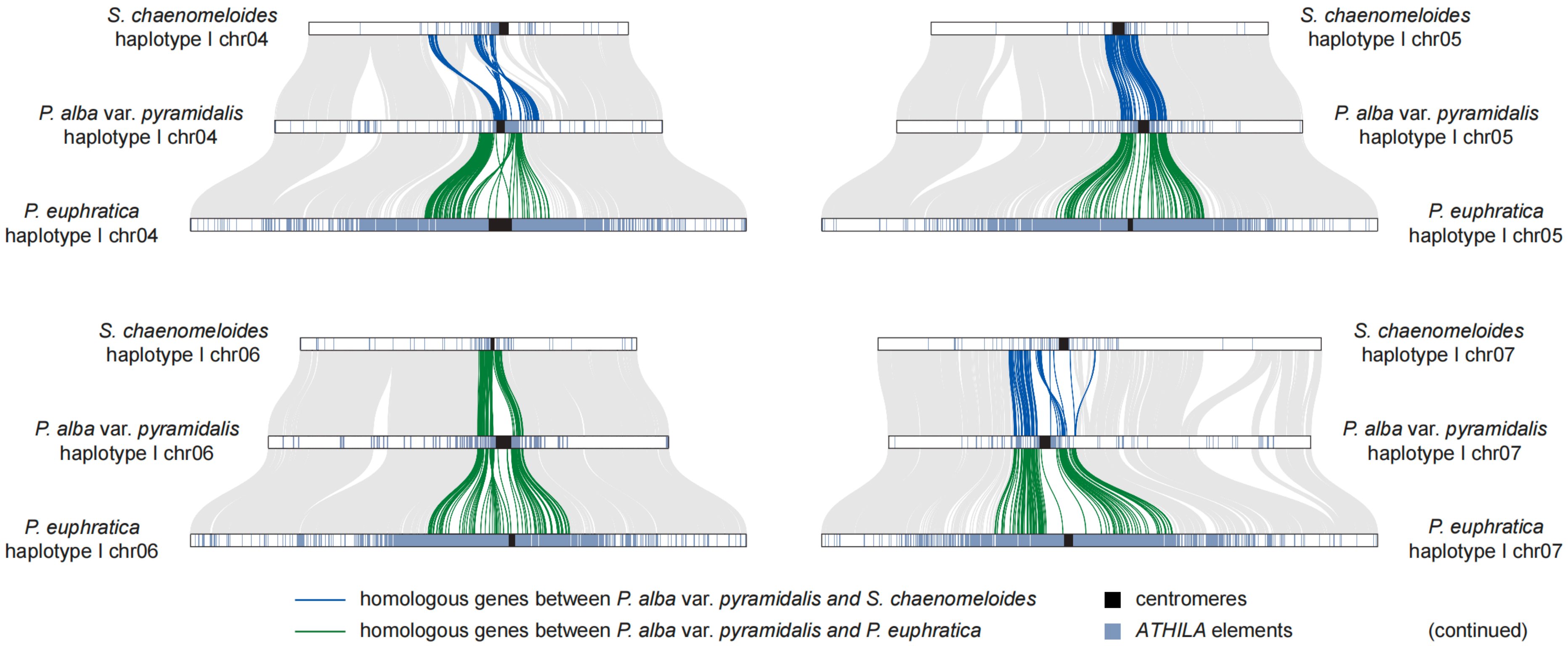
S. chaenomeloides haplotype I chr04
P. alba var. pyramidalis haplotype I chr04
P. euphratica haplotype I chr04
S. chaenomeloides haplotype I chr05
P. alba var. pyramidalis haplotype I chr05
P. euphratica haplotype I chr05
S. chaenomeloides haplotype I chr06
P. alba var. pyramidalis haplotype I chr06
P. euphratica haplotype I chr06
S. chaenomeloides haplotype I chr07
P. alba var. pyramidalis haplotype I chr07
P. euphratica haplotype I chr07
homologous genes between P. alba var. pyramidalis and S. chaenomeloides
homologous genes between P. alba var. pyramidalis and P. euphratica
centromeres
ATHILA elements

(continued)

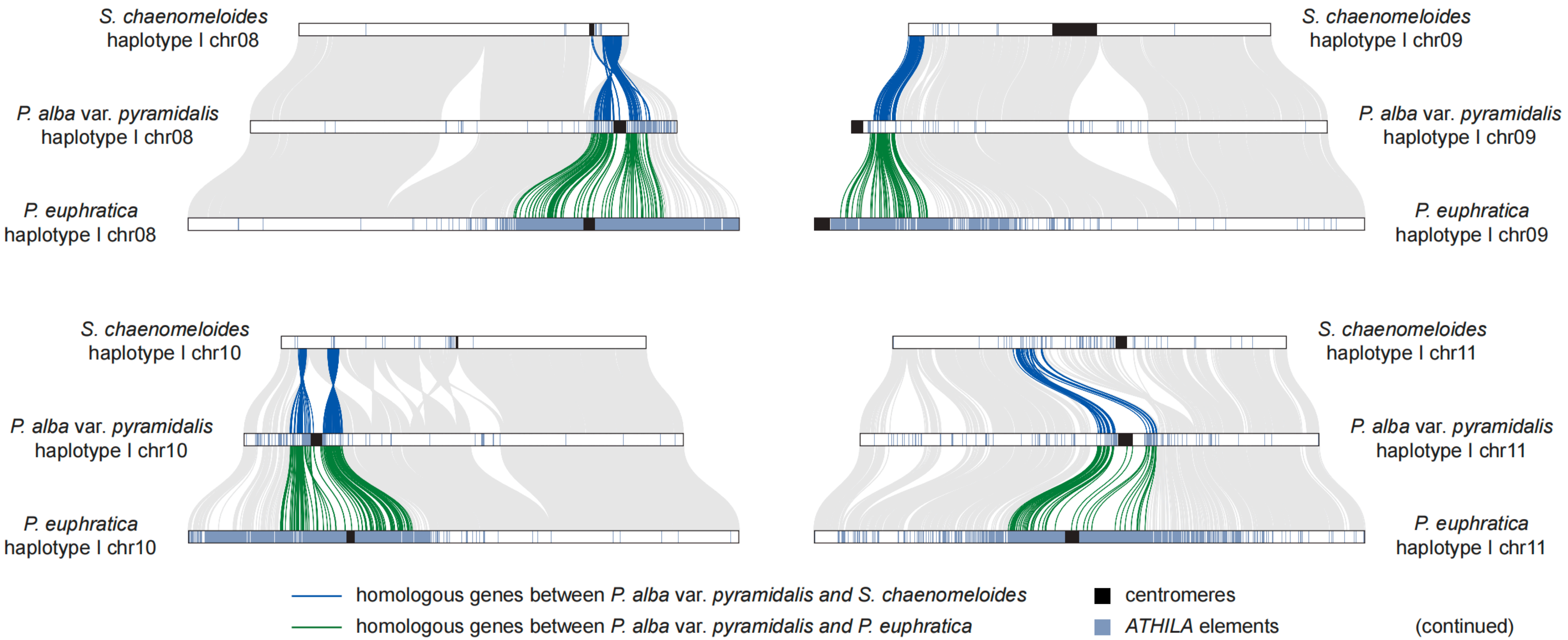

S. chaenomeloides haplotype I chr08
P. alba var. pyramidalis haplotype I chr08
P. euphratica haplotype I chr08
S. chaenomeloides haplotype I chr09
P. alba var. pyramidalis haplotype I chr09
P. euphratica haplotype I chr09
S. chaenomeloides haplotype I chr10
P. alba var. pyramidalis haplotype I chr10
P. euphratica haplotype I chr10
S. chaenomeloides haplotype I chr11
P. alba var. pyramidalis haplotype I chr11
P. euphratica haplotype I chr11
homologous genes between P. alba var. pyramidalis and S. chaenomeloides
homologous genes between P. alba var. pyramidalis and P. euphratica
centromeres
ATHILA elements

(continued)

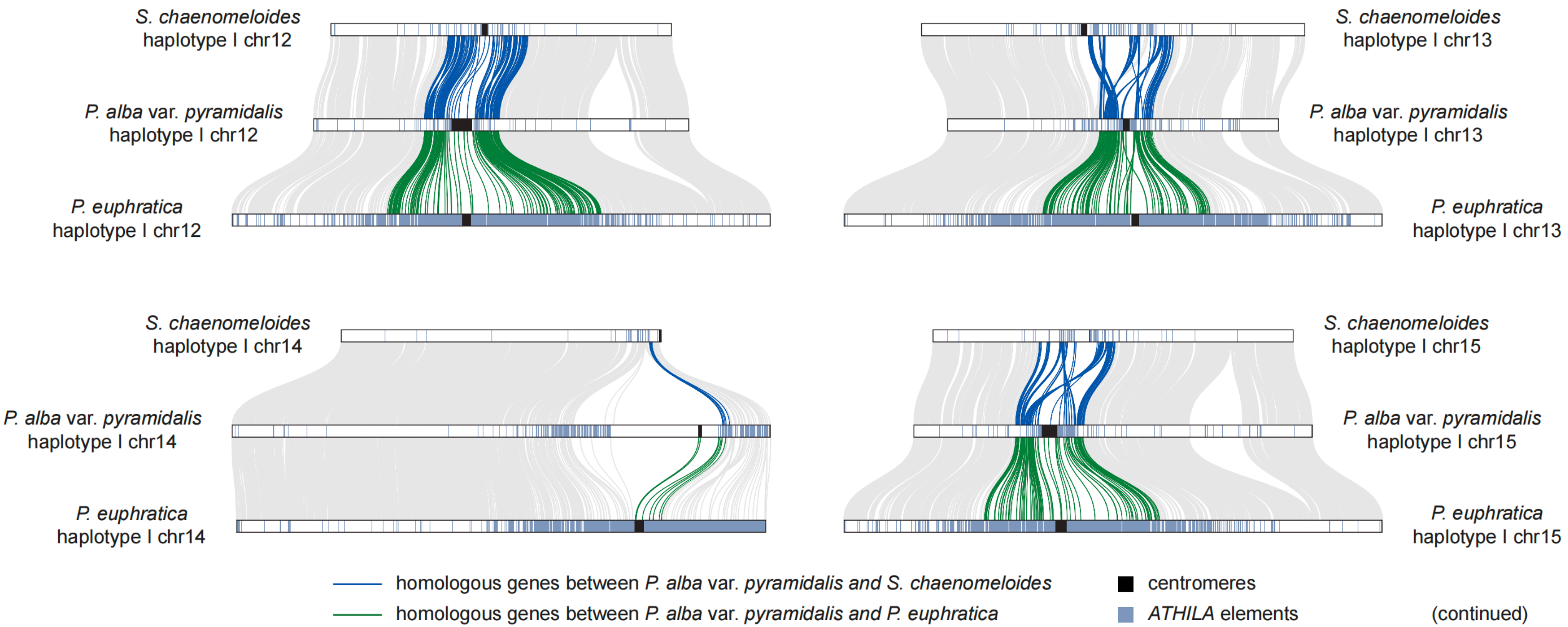

S. chaenomeloides haplotype I chr12
P. alba var. pyramidalis haplotype I chr12
P. euphratica haplotype I chr12
S. chaenomeloides haplotype I chr13
P. alba var. pyramidalis haplotype I chr13
P. euphratica haplotype I chr13
S. chaenomeloides haplotype I chr14
P. alba var. pyramidalis haplotype I chr14
P. euphratica haplotype I chr14
S. chaenomeloides haplotype I chr15
P. alba var. pyramidalis haplotype I chr15
P. euphratica haplotype I chr15
homologous genes between P. alba var. pyramidalis and S. chaenomeloides
homologous genes between P. alba var. pyramidalis and P. euphratica
centromeres
ATHILA elements

(continued)

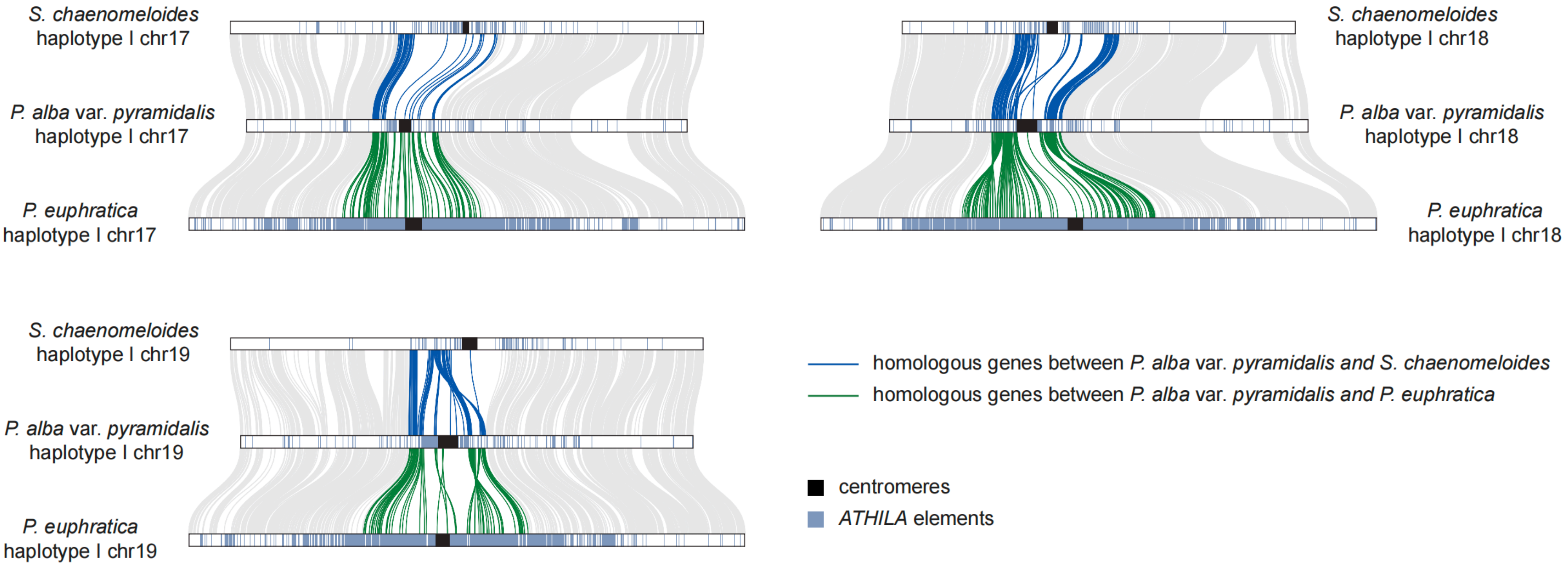

Fig. S24. *ATHILA* elements significantly expanded in *P. euphratica*. Straight bars represent homologous chromosomes of *S. chaenomeloides* haplotype I, *P. alba* var. *pyramidalis* haplotype I and *P. euphratica* I, which are scaled by chromosome length. Black boxes on lines represent corresponding centromeres. Grey lines connecting chromosomes represent homologous genes pairs across genomes, and colored lines represent homologous genes located within centromeres and peri-centromeric regions of *P. alba* var. *pyramidalis*. Light blue histograms in bars represent the distribution of *ATHILA* elements in chromosomes.

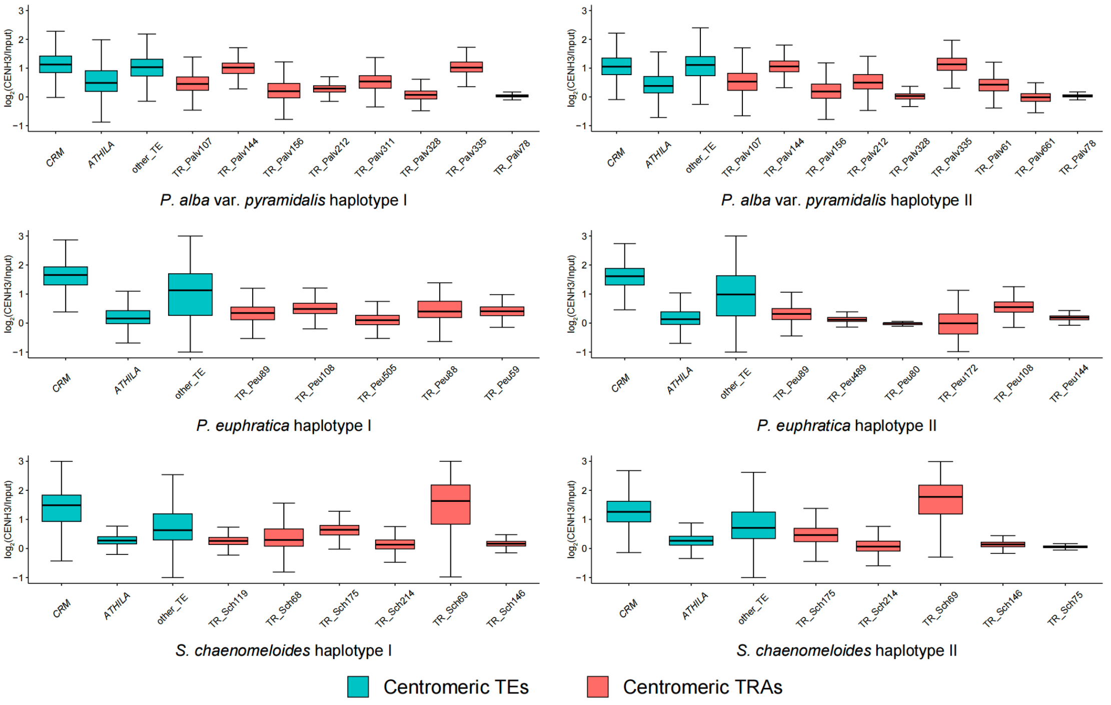

Fig. S25. Comparisons of CENH3 enrichment between TEs and TRAs within centromeres.

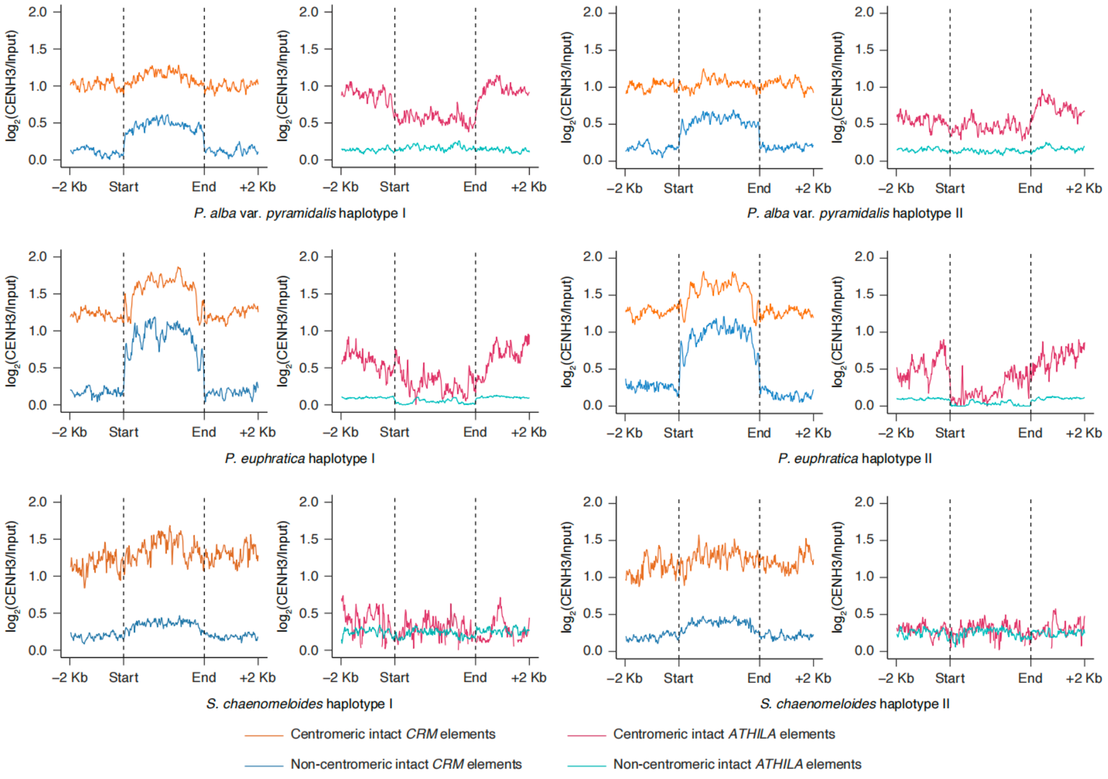

Fig. S26. Metaprofles of CENH3 enrichment for intact *CRM* and *ATHILA* elements and adjacent regions.

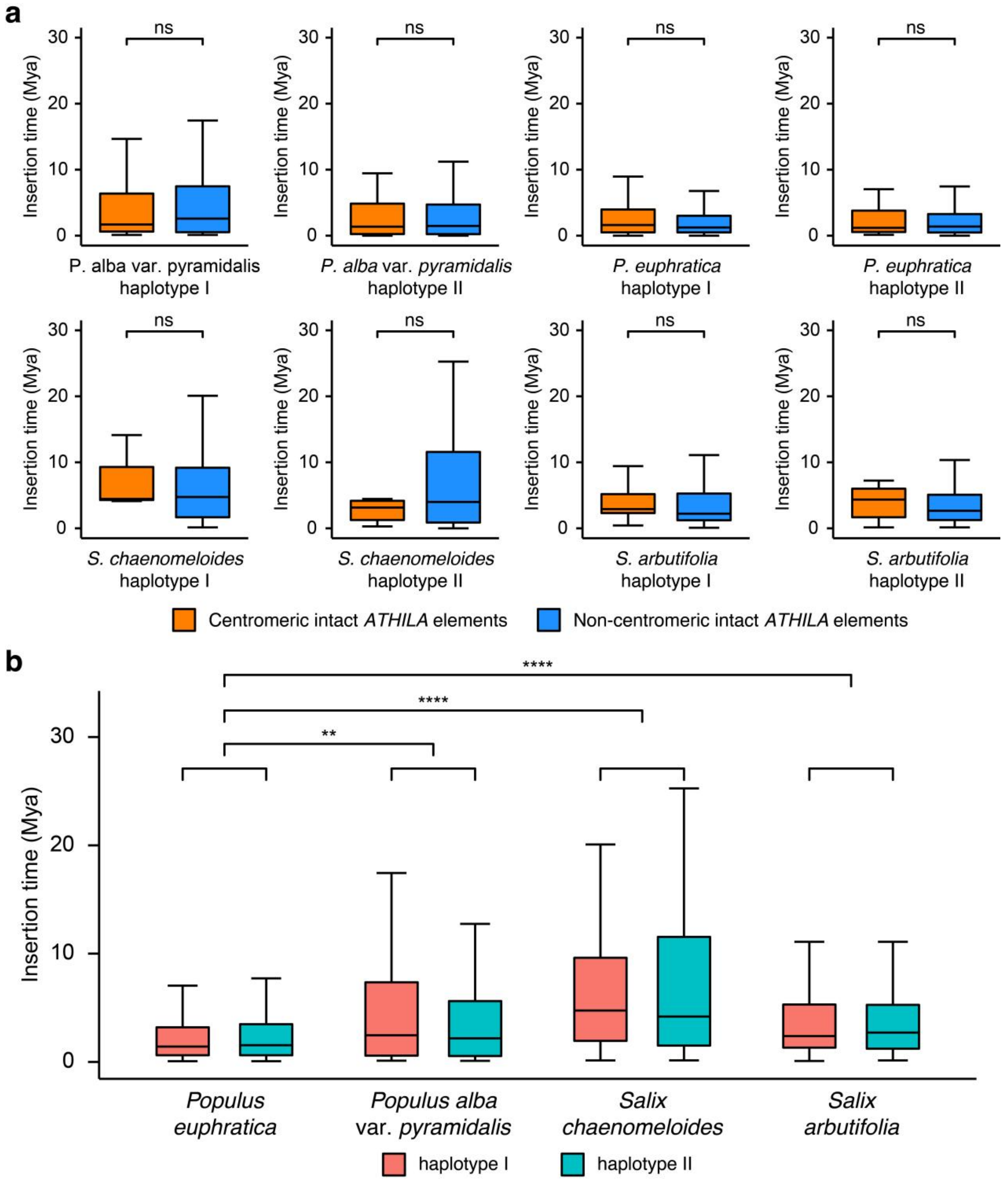

Fig. S27. Insertion time comparisons of *ATHILA* elements. **a** Comparisons of centromeric and non-centromeric *ATHILA* elements within each genome. **b** Same as **a** but showing comparisons between *P. euphratica* and other species. Asterisks represent significant differences (two-tailed Wilcoxon rank-sum test, *****P* <= 0.0001, ****P* <= 0.001, ***P* <= 0.01, **P* <= 0.05, ns: not significant).

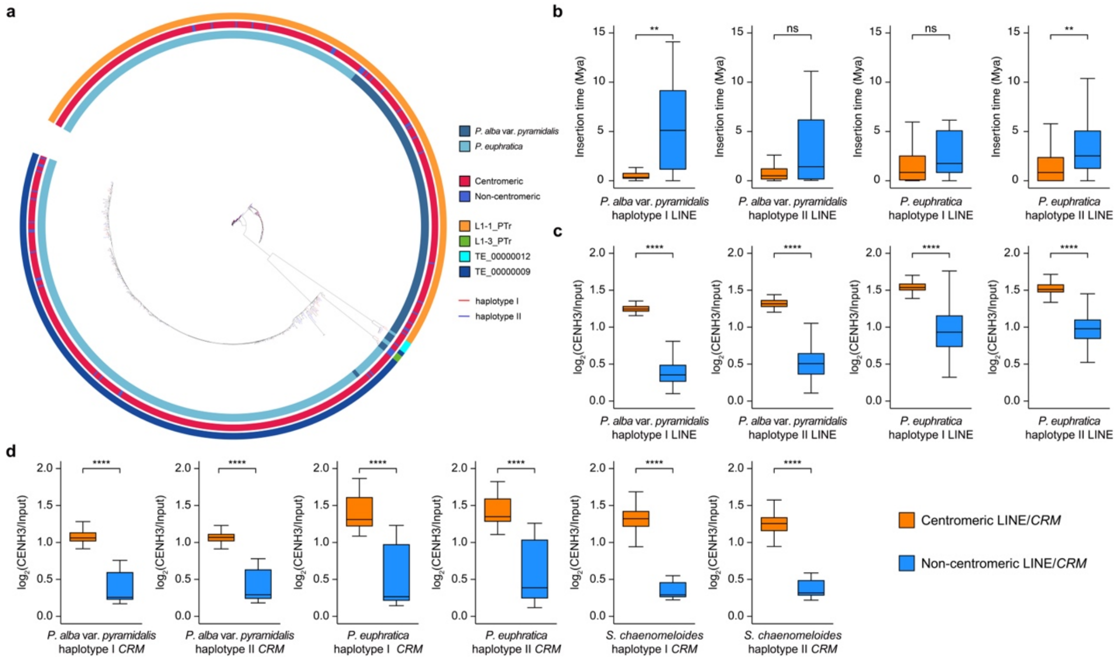

Fig. S28. Intact LINE and *CRM* elements analysis. a Phylogenetic tree of intact LINE elements. Colors of the outer, middle and inner circle and the tree branch represented the corresponding species, LINE position, LINE type and genome haplotype, respectively. The distance scale is 0.1. **b** Insertion time comparisons between centromeric and non-centromeric intact LINE elements in *P. alba* var. *pyramidalis* and *P. euphratica.* (two-tailed Wilcoxon rank-sum test, ****P <= 0.0001, ***P <= 0.001, **P <= 0.01, *P <= 0.05, ns: not significant). **c** Same as **b** but showing CENH3 modification comparisons between centromeric and non-centromeric intact LINE elements in *P. alba* var. *pyramidalis* and *P. euphratica.* **d** Same as **c** but showing intact *CRM* elements in *P. alba* var. *pyramidalis*, *P. euphratica* and *S. chaenomeloides*.

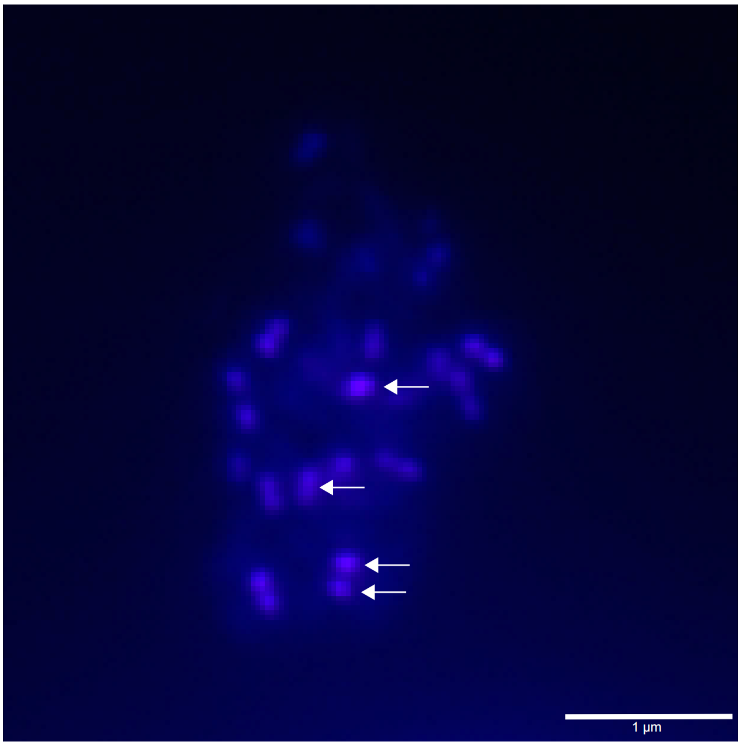

Fig. S29. FISH of the LINE element 'TE_000000009' on the somatic metaphase chromosomes prepared from *P. euphratica*. White arrows represent FISH signals.

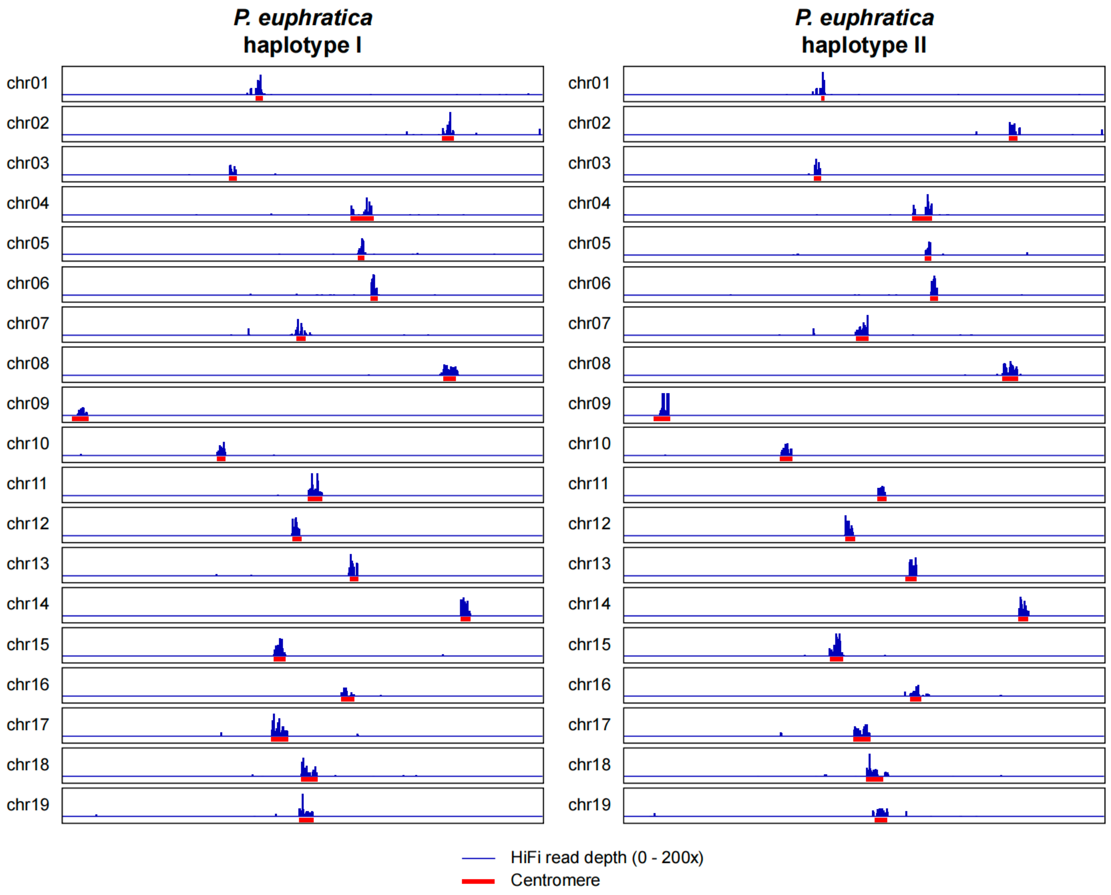

Fig. S30. Depth of HiFi reads containing 'TE_000000009' element on genomes of *P. euphratica*. The window size of calculating read depth is 10 kb.

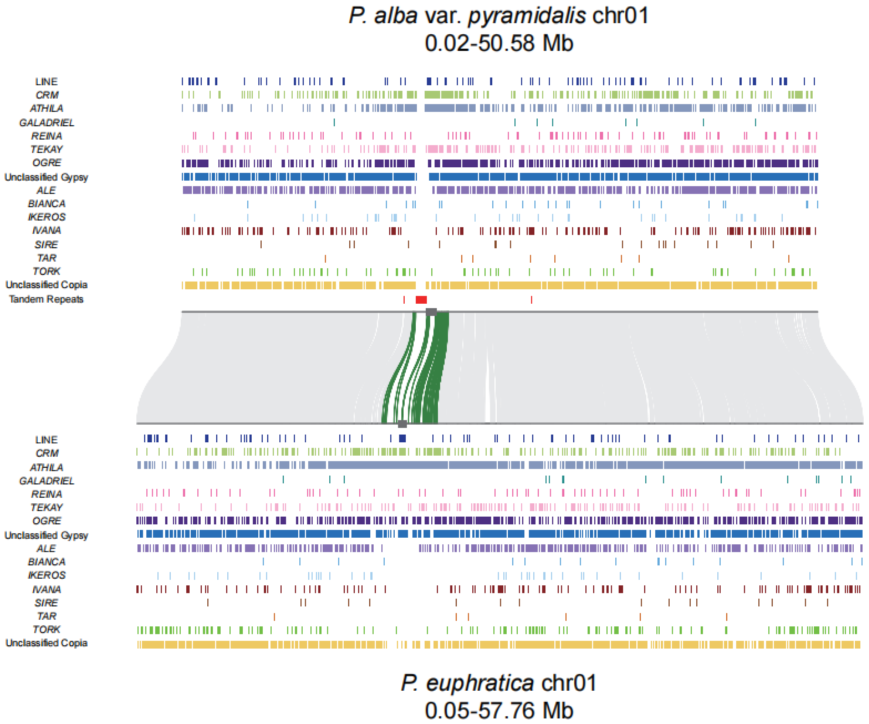
P. alba var. pyramidalis chr01
0.02-50.58 Mb
LINE
CRM
ATHILA
GALADRIEL
REINA
TEKAY
OGRE
Unclassified Gypsy
ALE
BIANCA
IKEROS
IVANA
SIRE
TAR
TORK
Unclassified Copia
Tandem Repeats
P. euphratica chr01
0.05-57.76 Mb

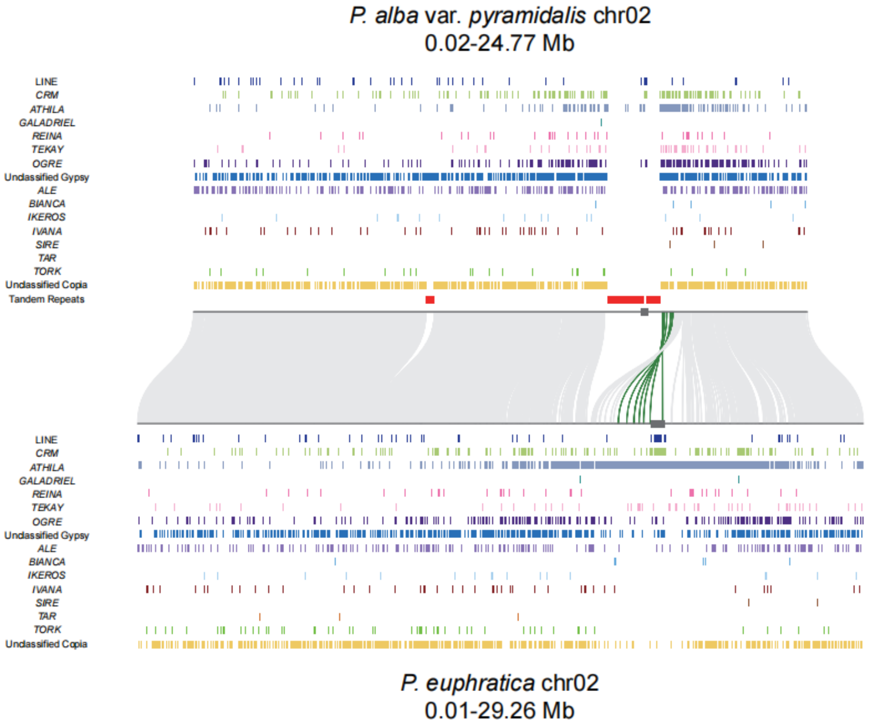
P. alba var. pyramidalis chr02
0.02-24.77 Mb
LINE
CRM
ATHILA
GALADRIEL
REINA
TEKAY
OGRE
Unclassified Gypsy
ALE
BIANCA
IKEROS
IVANA
SIRE
TAR
TORK
Unclassified Copia
Tandem Repeats
P. euphratica chr02
0.01-29.26 Mb

(continued)

P. alba var. *pyramidalis* chr03
0.07-21.24 Mb

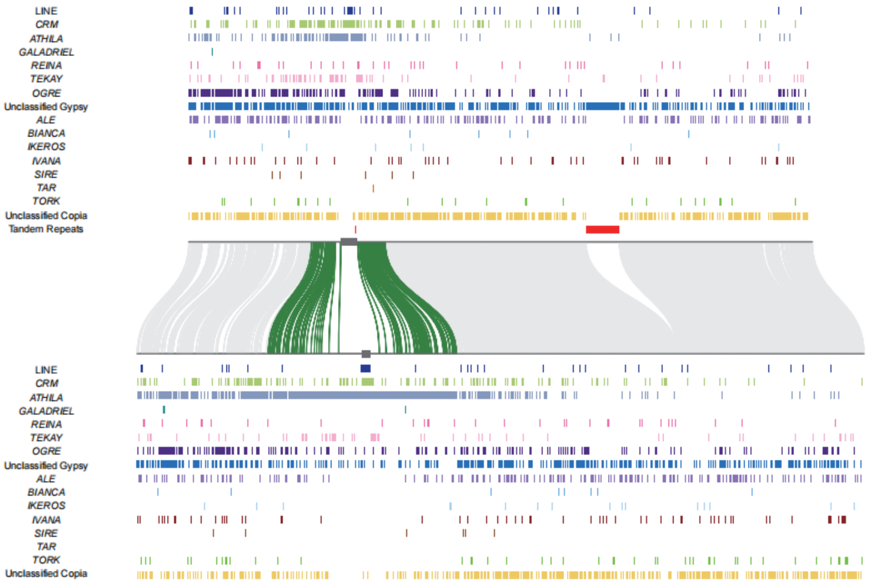

P. euphratica chr03
0.09-24.77 Mb

P. alba var. *pyramidalis* chr04
0.06-22.61 Mb

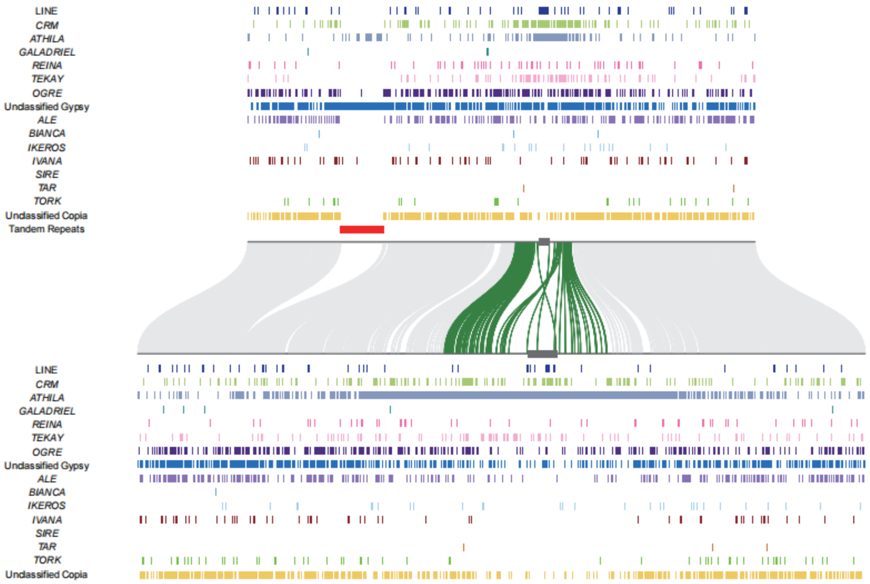

P. euphratica chr04
0.01-32.36 Mb

(continued)

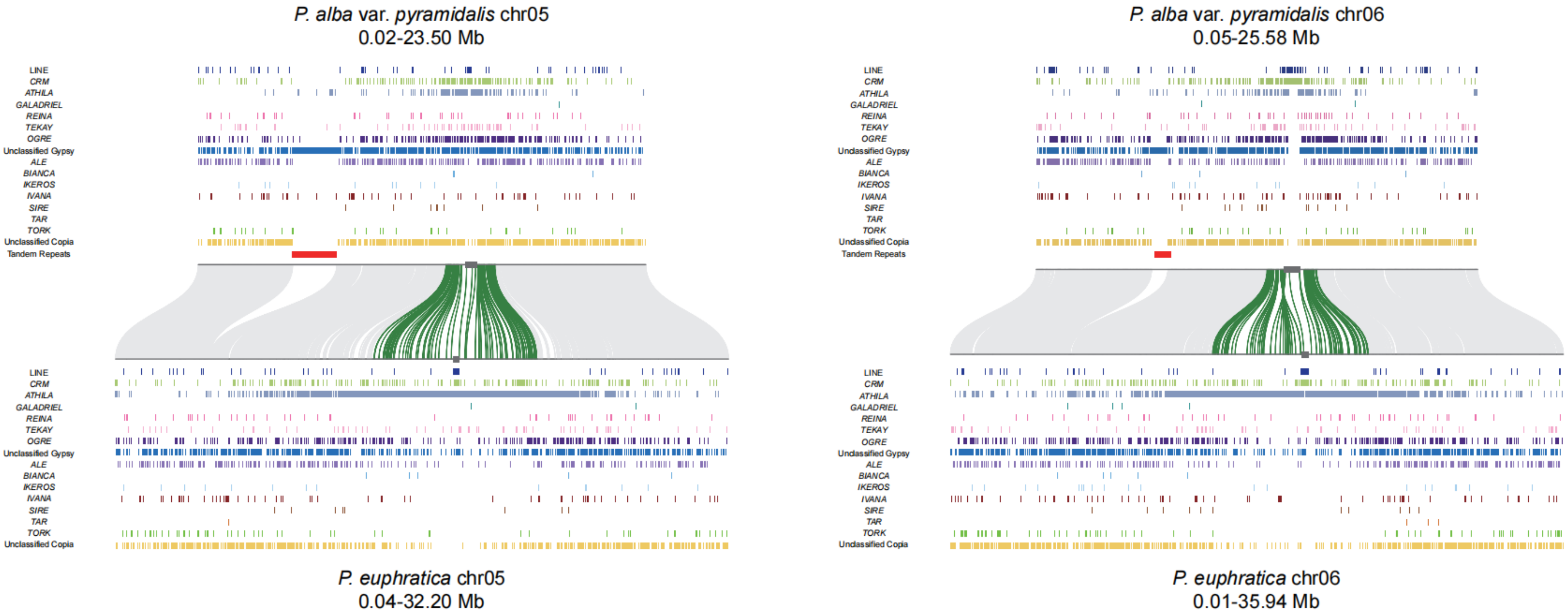
P. alba var. pyramidalis chr05
0.02-23.50 Mb
P. euphratica chr05
0.04-32.20 Mb
P. alba var. pyramidalis chr06
0.05-25.58 Mb
P. euphratica chr06
0.01-35.94 Mb
LINE
CRM
ATHILA
GALADRIEL
REINA
TEKAY
OGRE
Unclassified Gypsy
ALE
BIANCA
IKEROS
IVANA
SIRE
TAR
TORK
Unclassified Copia
Tandem Repeats

(continued)

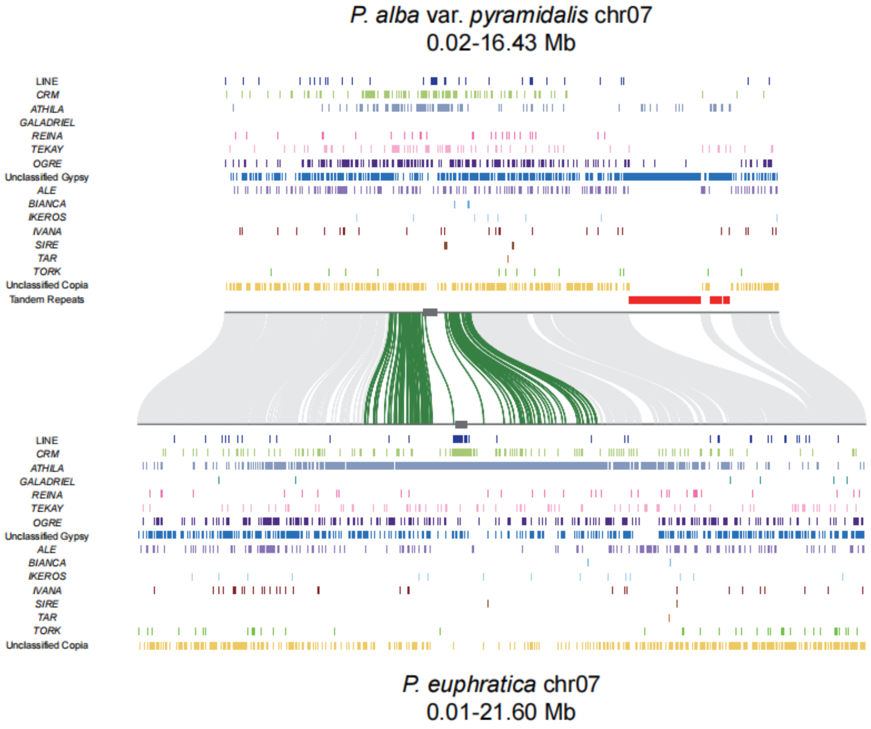

P. alba var. pyramidalis chr07
0.02-16.43 Mb
LINE
CRM
ATHILA
GALADRIEL
REINA
TEKAY
OGRE
Unclassified Gypsy
ALE
BIANCA
IKEROS
IVANA
SIRE
TAR
TORK
Unclassified Copia
Tandem Repeats
P. euphratica chr07
0.01-21.60 Mb

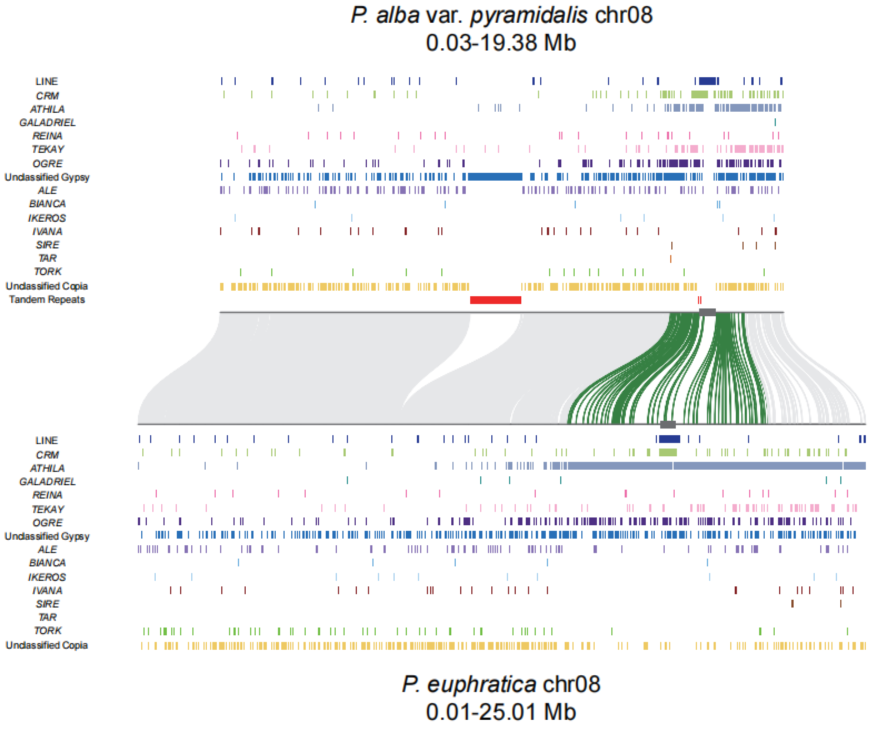

P. alba var. pyramidalis chr08
0.03-19.38 Mb
LINE
CRM
ATHILA
GALADRIEL
REINA
TEKAY
OGRE
Unclassified Gypsy
ALE
BIANCA
IKEROS
IVANA
SIRE
TAR
TORK
Unclassified Copia
Tandem Repeats
P. euphratica chr08
0.01-25.01 Mb

(continued)

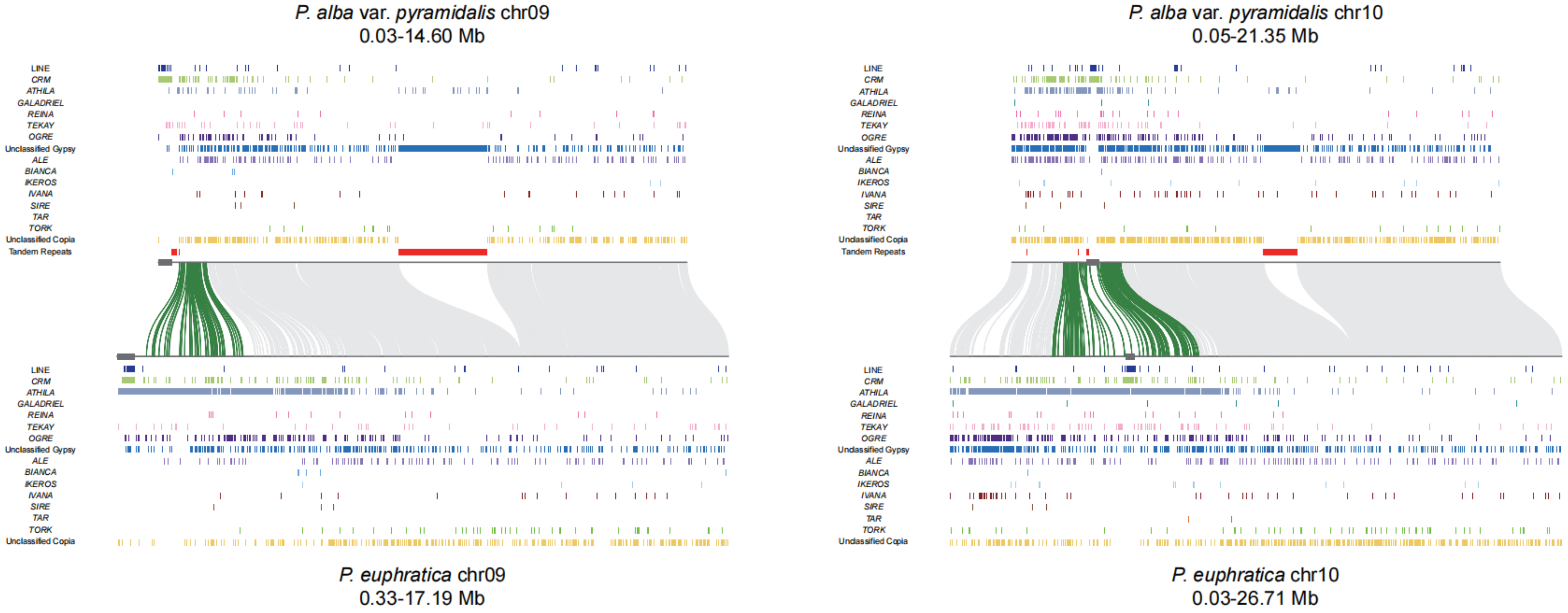

P. alba var. pyramidalis chr09
0.03-14.60 Mb
P. alba var. pyramidalis chr10
0.05-21.35 Mb
LINE
CRM
ATHILA
GALADRIEL
REINA
TEKAY
OGRE
Unclassified Gypsy
ALE
BIANCA
IKEROS
IVANA
SIRE
TAR
TORK
Unclassified Copia
Tandem Repeats
P. euphratica chr09
0.33-17.19 Mb
P. euphratica chr10
0.03-26.71 Mb

(continued)

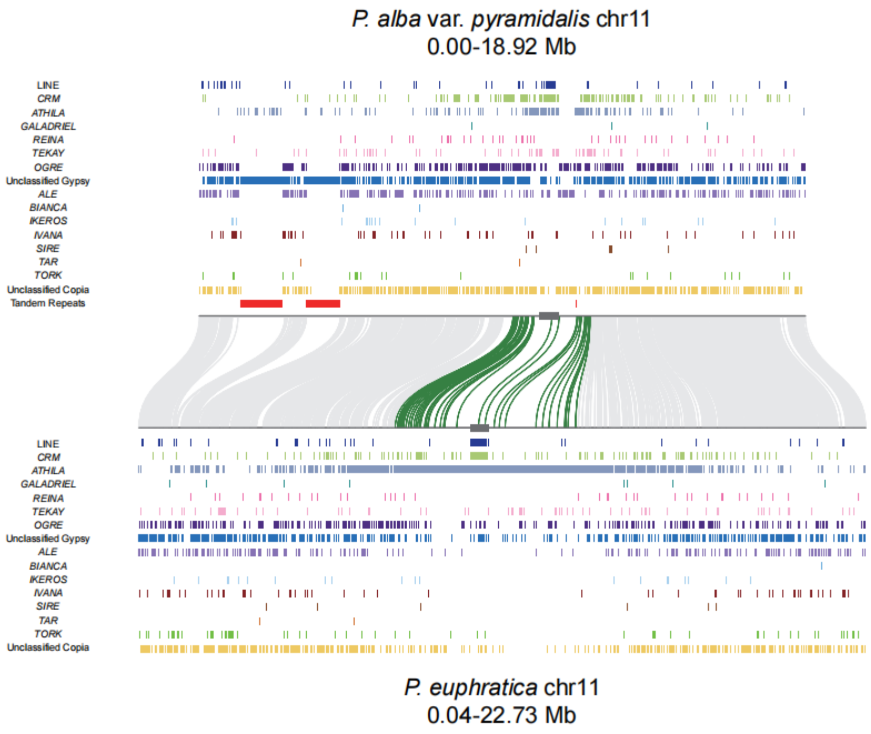
P. alba var. pyramidalis chr11
0.00-18.92 Mb
LINE
CRM
ATHILA
GALADRIEL
REINA
TEKAY
OGRE
Unclassified Gypsy
ALE
BIANCA
IKEROS
IVANA
SIRE
TAR
TORK
Unclassified Copia
Tandem Repeats
P. euphratica chr11
0.04-22.73 Mb

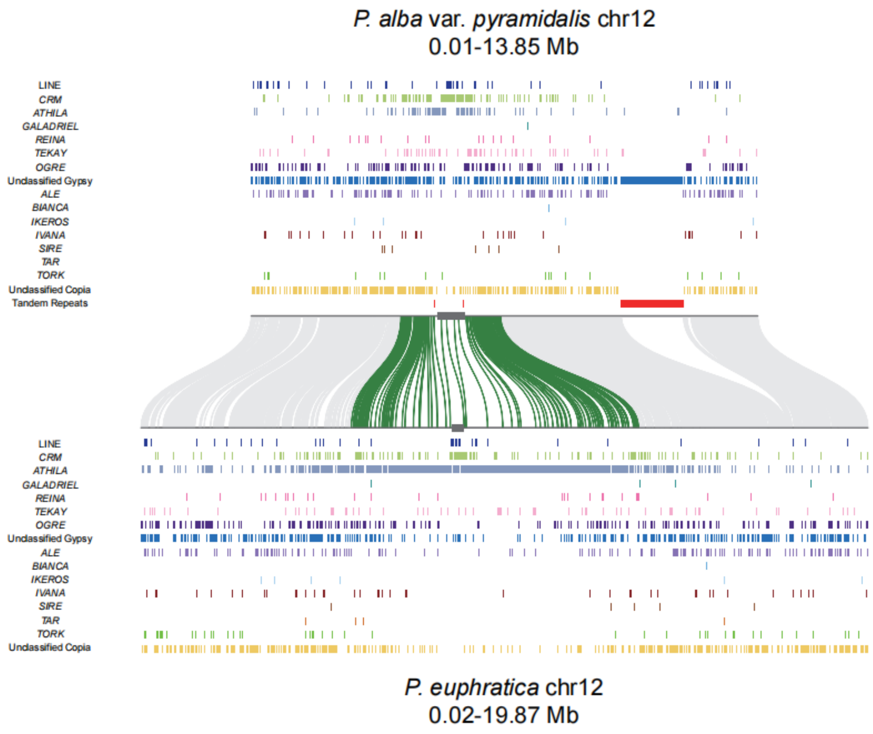
P. alba var. pyramidalis chr12
0.01-13.85 Mb
LINE
CRM
ATHILA
GALADRIEL
REINA
TEKAY
OGRE
Unclassified Gypsy
ALE
BIANCA
IKEROS
IVANA
SIRE
TAR
TORK
Unclassified Copia
Tandem Repeats
P. euphratica chr12
0.02-19.87 Mb

(continued)

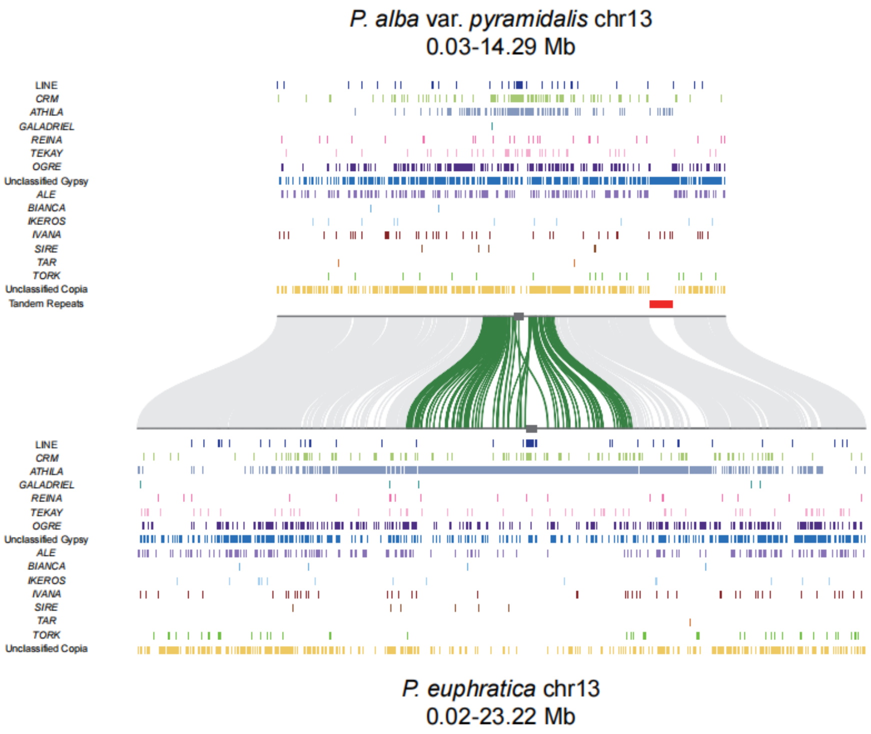

P. alba var. pyramidalis chr13
0.03-14.29 Mb
LINE
CRM
ATHILA
GALADRIEL
REINA
TEKAY
OGRE
Unclassified Gypsy
ALE
BIANCA
IKEROS
IVANA
SIRE
TAR
TORK
Unclassified Copia
Tandem Repeats
P. euphratica chr13
0.02-23.22 Mb

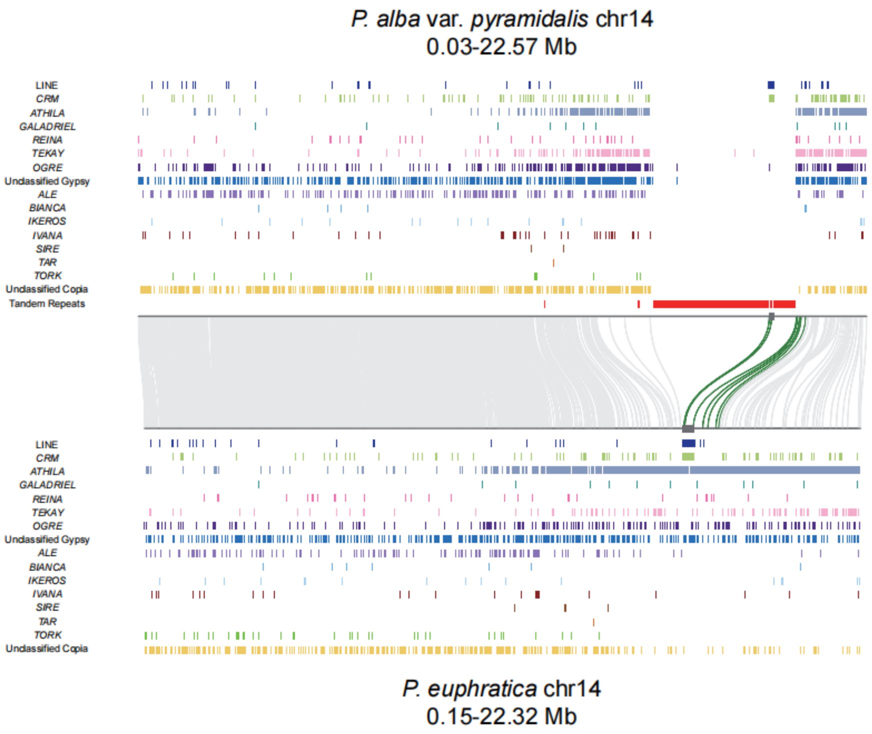

P. alba var. pyramidalis chr14
0.03-22.57 Mb
LINE
CRM
ATHILA
GALADRIEL
REINA
TEKAY
OGRE
Unclassified Gypsy
ALE
BIANCA
IKEROS
IVANA
SIRE
TAR
TORK
Unclassified Copia
Tandem Repeats
P. euphratica chr14
0.15-22.32 Mb

(continued)

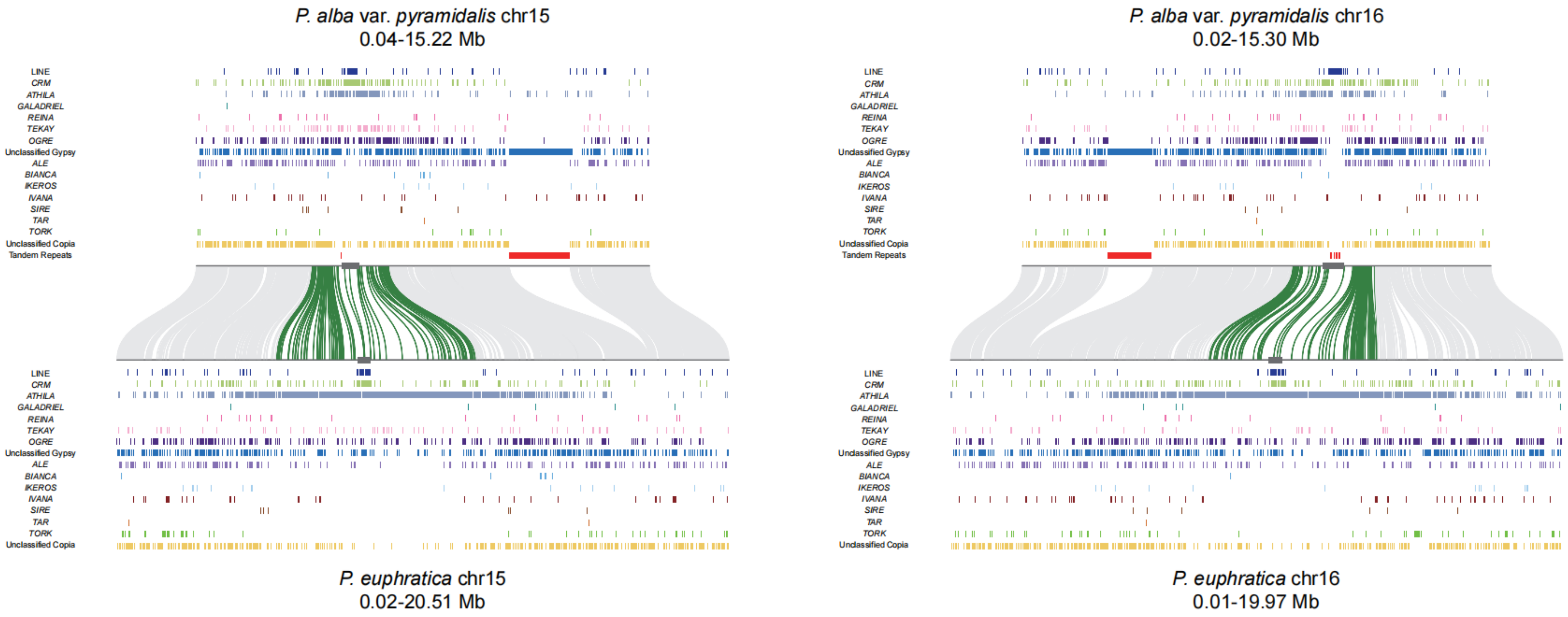
P. alba var. pyramidalis chr15
0.04-15.22 Mb
P. alba var. pyramidalis chr16
0.02-15.30 Mb
LINE
CRM
ATHILA
GALADRIEL
REINA
TEKAY
OGRE
Unclassified Gypsy
ALE
BIANCA
IKEROS
IVANA
SIRE
TAR
TORK
Unclassified Copia
Tandem Repeats
P. euphratica chr15
0.02-20.51 Mb
P. euphratica chr16
0.01-19.97 Mb

(continued)

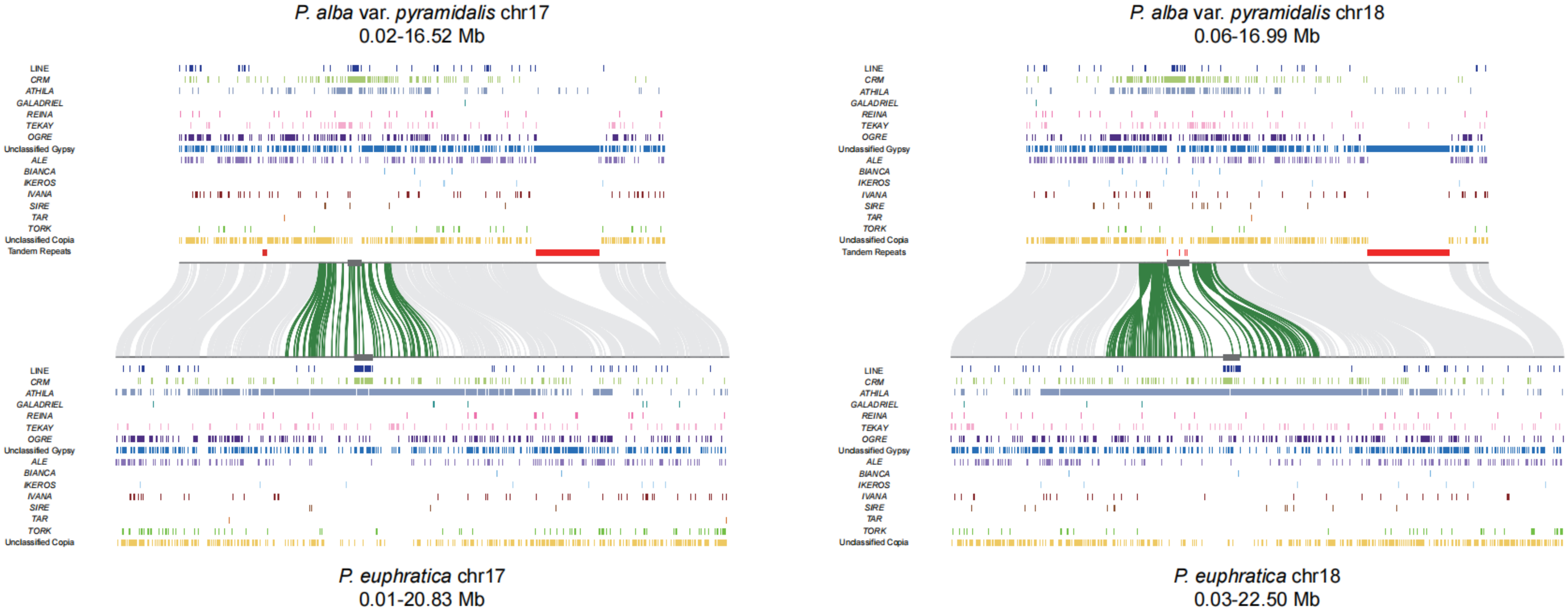
P. alba var. pyramidalis chr17
0.02-16.52 Mb
P. alba var. pyramidalis chr18
0.06-16.99 Mb
LINE
CRM
ATHILA
GALADRIEL
REINA
TEKAY
OGRE
Unclassified Gypsy
ALE
BIANCA
IKEROS
IVANA
SIRE
TAR
TORK
Unclassified Copia
Tandem Repeats
P. euphratica chr17
0.01-20.83 Mb
P. euphratica chr18
0.03-22.50 Mb

(continued)

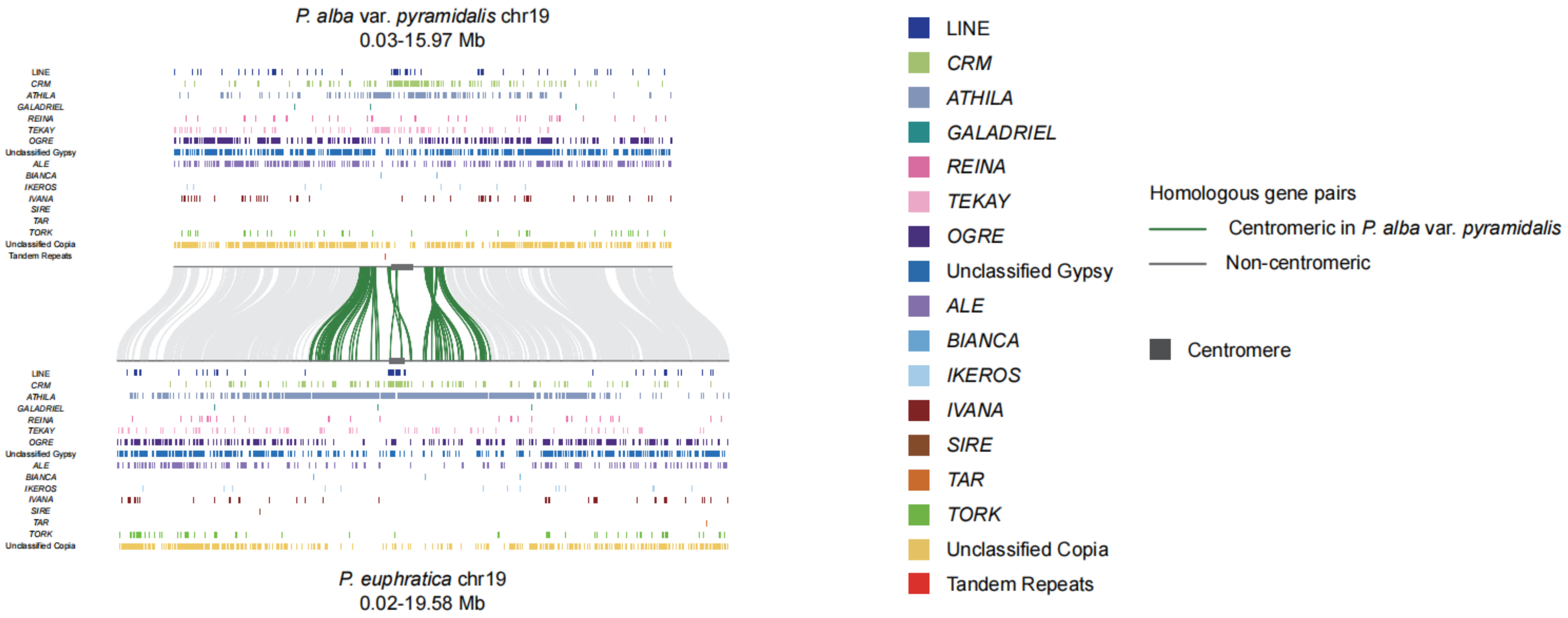

Fig. S31. Synteny analysis of homologous chromosomes between *P. alba* var. *pyramidalis* and *P. euphratica*. The two straight bars in the middle represent homologous chromosomes scaled by chromosome length and dark grey boxes on lines represent corresponding centromeres. Grey lines connecting chromosomes represent homologous genes pairs between *P. alba* var. *pyramidalis* and *P. euphratica* haplotype I genome, and green lines represent homologous genes located within centromeres and peri-centromeric regions of *P. alba* var. *pyramidalis*. Histograms represent the distribution of each type of LTRs and LINE elements from *P. alba* var. *pyramidalis* and *P. euphratica*.

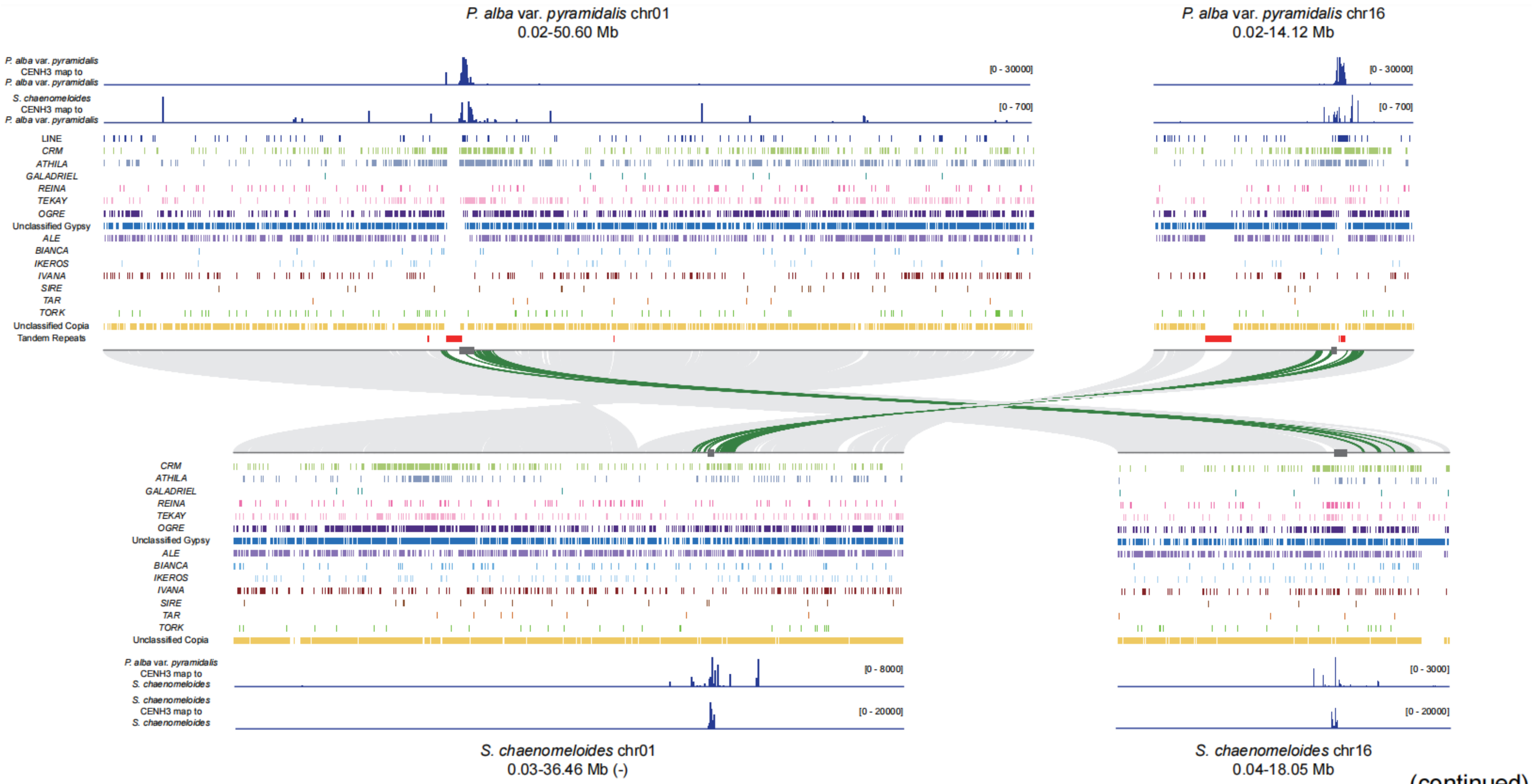

P. alba var. pyramidalis chr01
0.02-50.60 Mb
P. alba var. pyramidalis chr16
0.02-14.12 Mb
P. alba var. pyramidalis CENH3 map to P. alba var. pyramidalis
[0 - 30000]
S. chaenomeloides CENH3 map to P. alba var. pyramidalis
[0 - 700]
LINE
CRM
ATHILA
GALADRIEL
REINA
TEKAY
OGRE
Unclassified Gypsy
ALE
BIANCA
IKEROS
IVANA
SIRE
TAR
TORK
Unclassified Copia
Tandem Repeats
P. alba var. pyramidalis CENH3 map to S. chaenomeloides
[0 - 8000]
[0 - 3000]
S. chaenomeloides CENH3 map to S. chaenomeloides
[0 - 20000]
S. chaenomeloides chr01
0.03-36.46 Mb (-)
S. chaenomeloides chr16
0.04-18.05 Mb

(continued)

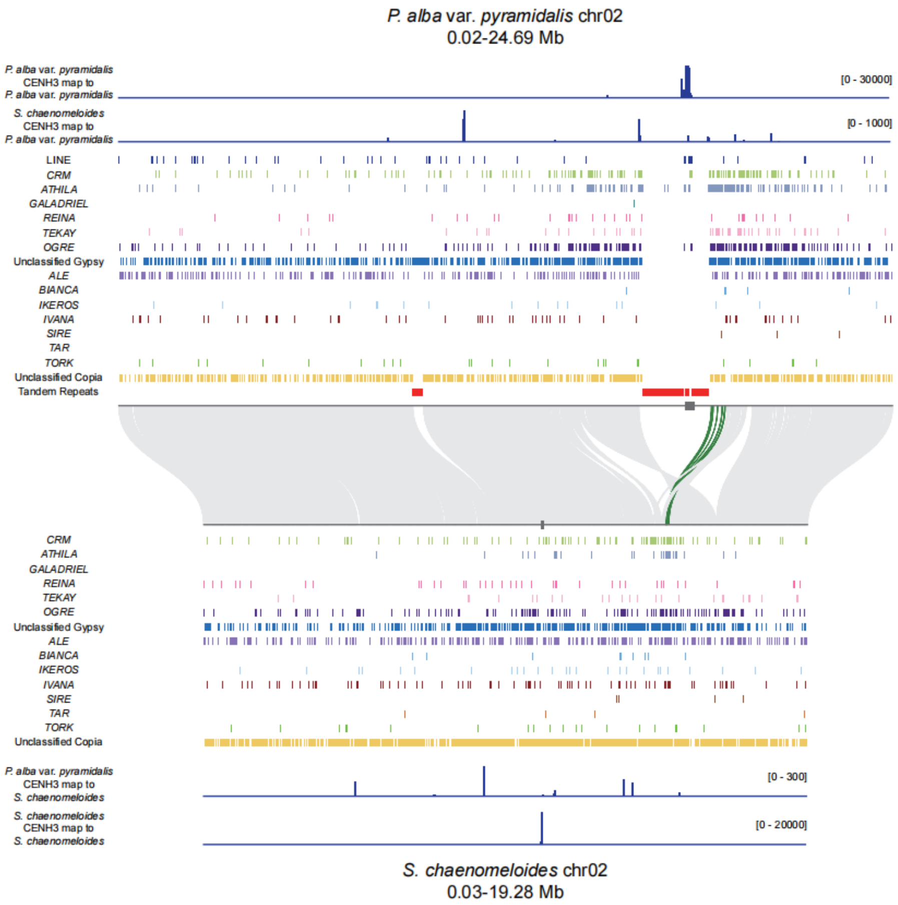
P. alba var. pyramidalis chr02
0.02-24.69 Mb
P. alba var. pyramidalis CENH3 map to P. alba var. pyramidalis
[0 - 30000]
S. chaenomeloides CENH3 map to P. alba var. pyramidalis
[0 - 1000]
LINE
CRM
ATHILA
GALADRIEL
REINA
TEKAY
OGRE
Unclassified Gypsy
ALE
BIANCA
IKEROS
IVANA
SIRE
TAR
TORK
Unclassified Copia
Tandem Repeats
P. alba var. pyramidalis CENH3 map to S. chaenomeloides
[0 - 300]
S. chaenomeloides CENH3 map to S. chaenomeloides
[0 - 20000]
S. chaenomeloides chr02
0.03-19.28 Mb

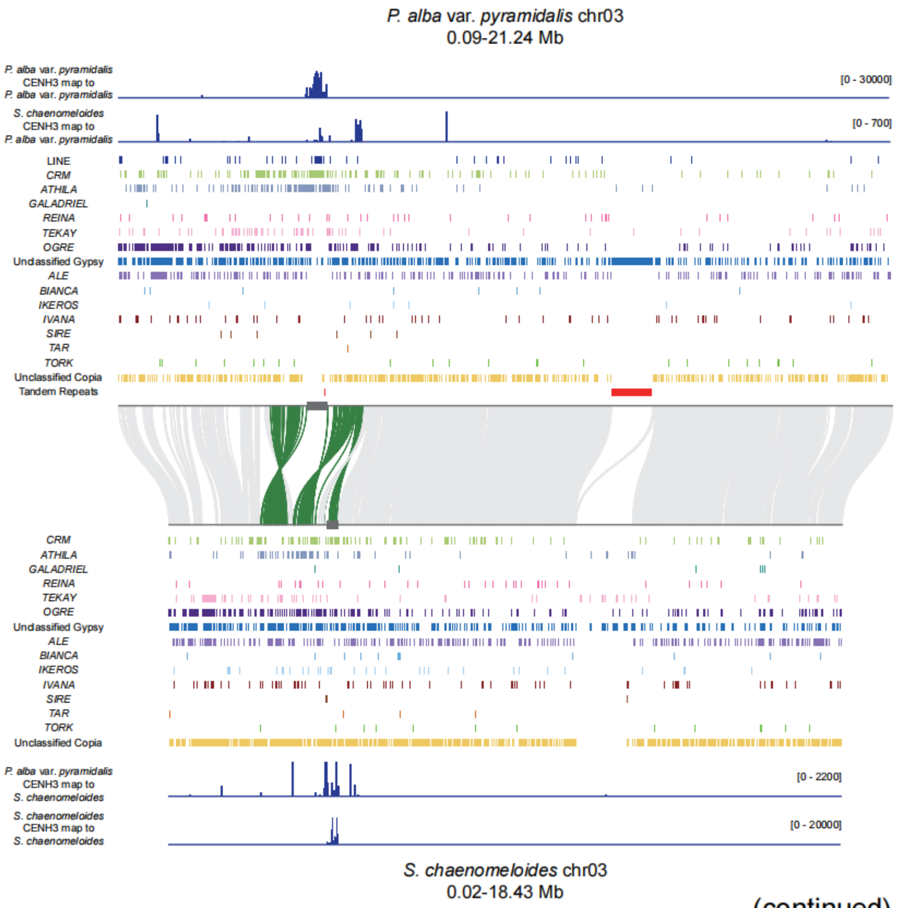
P. alba var. pyramidalis chr03
0.09-21.24 Mb
[0 - 30000]
[0 - 700]
[0 - 2200]
[0 - 20000]
S. chaenomeloides chr03
0.02-18.43 Mb

(continued)

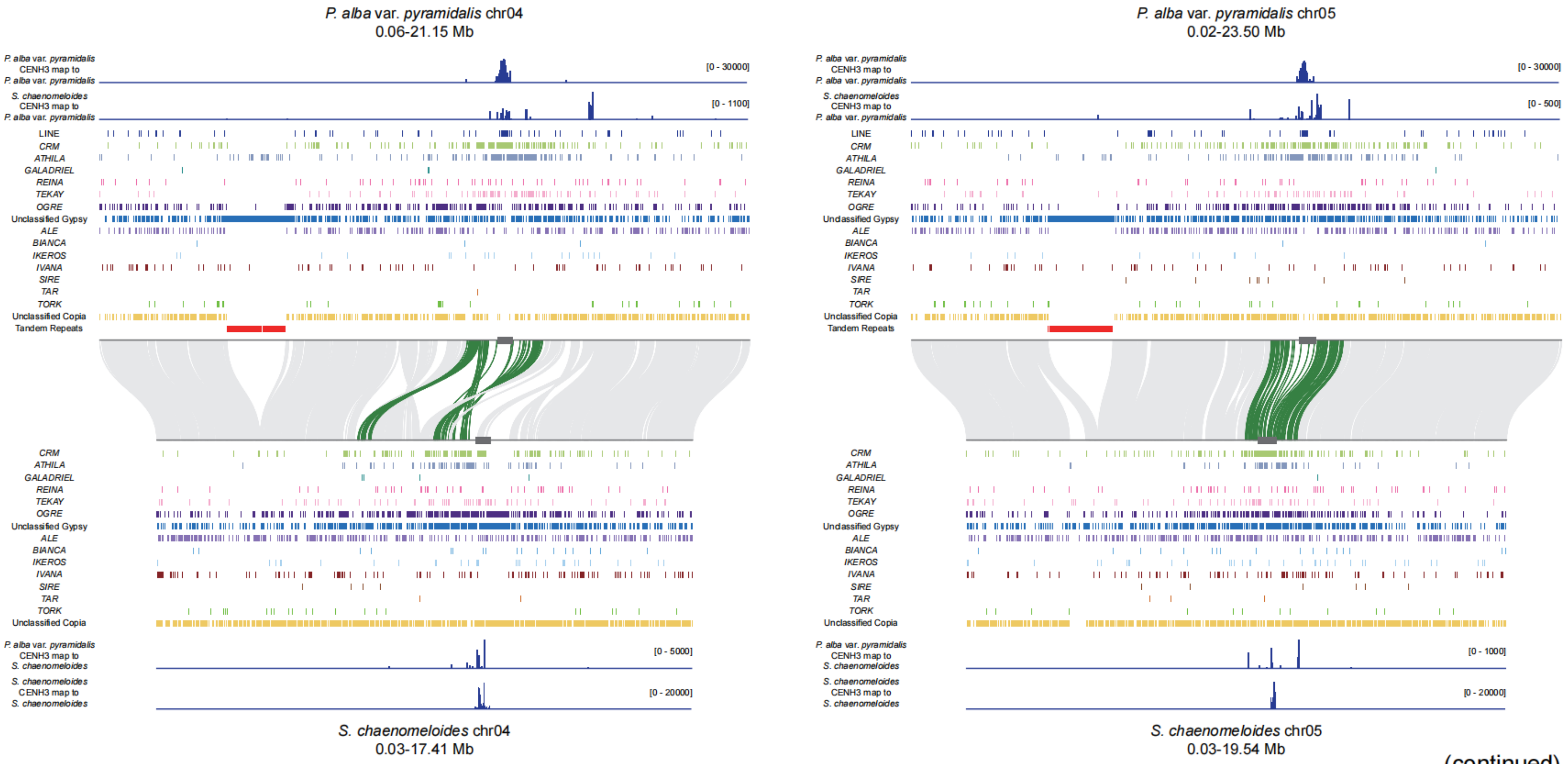
P. alba var. pyramidalis chr04
0.06-21.15 Mb
P. alba var. pyramidalis CENH3 map to P. alba var. pyramidalis
[0 - 30000]
S. chaenomeloides CENH3 map to P. alba var. pyramidalis
[0 - 1100]
LINE
CRM
ATHILA
GALADRIEL
REINA
TEKAY
OGRE
Unclassified Gypsy
ALE
BIANCA
IKEROS
IVANA
SIRE
TAR
TORK
Unclassified Copia
Tandem Repeats
CRM
ATHILA
GALADRIEL
REINA
TEKAY
OGRE
Unclassified Gypsy
ALE
BIANCA
IKEROS
IVANA
SIRE
TAR
TORK
Unclassified Copia
P. alba var. pyramidalis CENH3 map to S. chaenomeloides
[0 - 5000]
S. chaenomeloides CENH3 map to S. chaenomeloides
[0 - 20000]
S. chaenomeloides chr04
0.03-17.41 Mb
P. alba var. pyramidalis chr05
0.02-23.50 Mb
P. alba var. pyramidalis CENH3 map to P. alba var. pyramidalis
[0 - 30000]
S. chaenomeloides CENH3 map to P. alba var. pyramidalis
[0 - 500]
P. alba var. pyramidalis CENH3 map to S. chaenomeloides
[0 - 1000]
S. chaenomeloides CENH3 map to S. chaenomeloides
[0 - 20000]
S. chaenomeloides chr05
0.03-19.54 Mb

(continued)

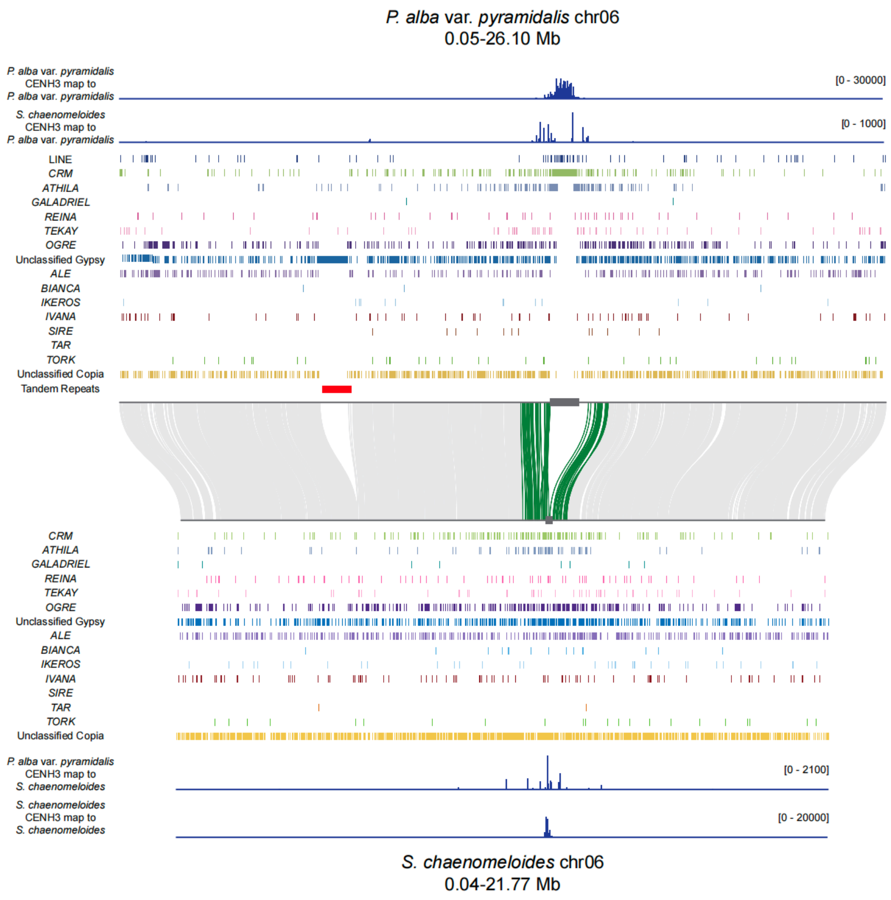
P. alba var. pyramidalis chr06
0.05-26.10 Mb
P. alba var. pyramidalis CENH3 map to P. alba var. pyramidalis
[0 - 30000]
S. chaenomeloides CENH3 map to P. alba var. pyramidalis
[0 - 1000]
LINE
CRM
ATHILA
GALADRIEL
REINA
TEKAY
OGRE
Unclassified Gypsy
ALE
BIANCA
IKEROS
IVANA
SIRE
TAR
TORK
Unclassified Copia
Tandem Repeats
P. alba var. pyramidalis CENH3 map to S. chaenomeloides
[0 - 2100]
S. chaenomeloides CENH3 map to S. chaenomeloides
[0 - 20000]
S. chaenomeloides chr06
0.04-21.77 Mb

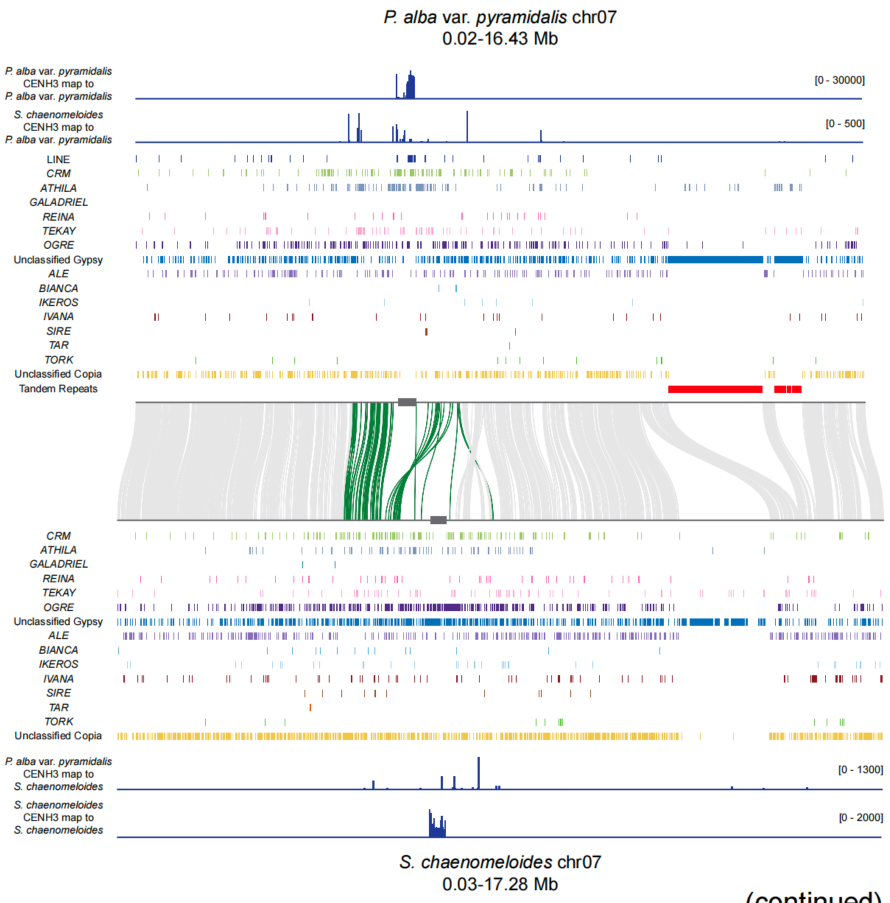
P. alba var. pyramidalis chr07
0.02-16.43 Mb
P. alba var. pyramidalis CENH3 map to P. alba var. pyramidalis
[0 - 30000]
S. chaenomeloides CENH3 map to P. alba var. pyramidalis
[0 - 500]
LINE
CRM
ATHILA
GALADRIEL
REINA
TEKAY
OGRE
Unclassified Gypsy
ALE
BIANCA
IKEROS
IVANA
SIRE
TAR
TORK
Unclassified Copia
Tandem Repeats
P. alba var. pyramidalis CENH3 map to S. chaenomeloides
[0 - 1300]
S. chaenomeloides CENH3 map to S. chaenomeloides
[0 - 2000]
S. chaenomeloides chr07
0.03-17.28 Mb

(continued)

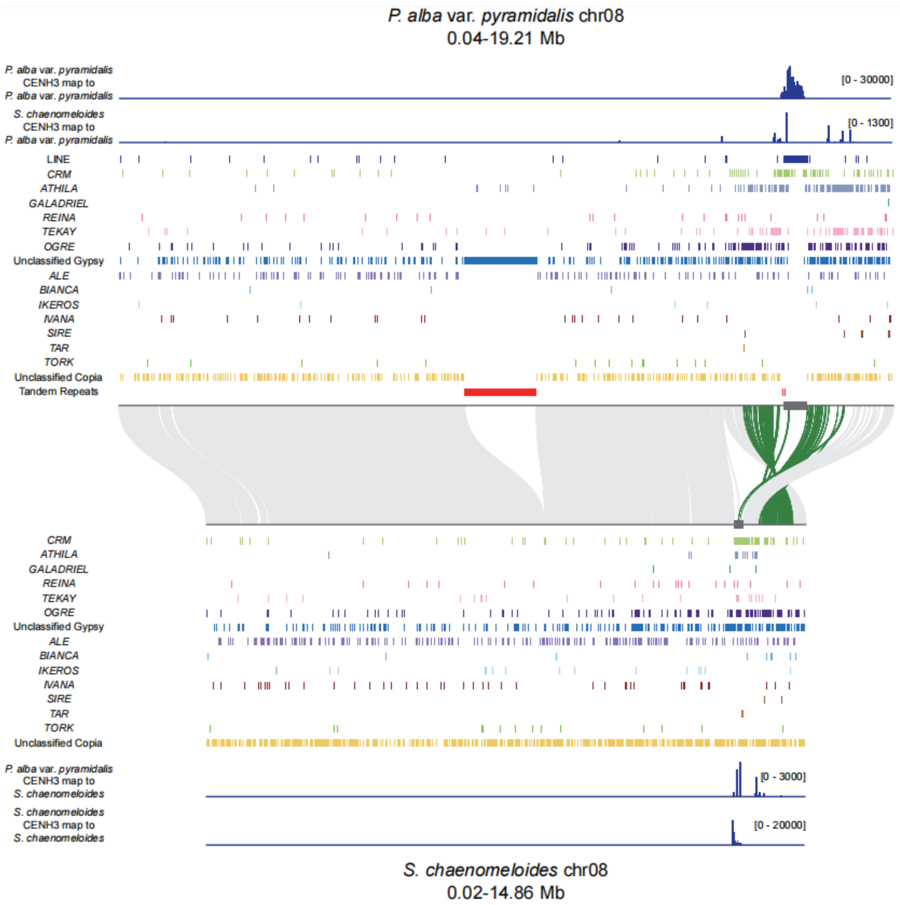

P. alba var. pyramidalis chr08
0.04-19.21 Mb
P. alba var. pyramidalis CENH3 map to P. alba var. pyramidalis
[0 - 30000]
S. chaenomeloides CENH3 map to P. alba var. pyramidalis
[0 - 1300]
LINE
CRM
ATHILA
GALADRIEL
REINA
TEKAY
OGRE
Unclassified Gypsy
ALE
BIANCA
IKEROS
IVANA
SIRE
TAR
TORK
Unclassified Copia
Tandem Repeats
P. alba var. pyramidalis CENH3 map to S. chaenomeloides
[0 - 3000]
S. chaenomeloides CENH3 map to S. chaenomeloides
[0 - 20000]
S. chaenomeloides chr08
0.02-14.86 Mb

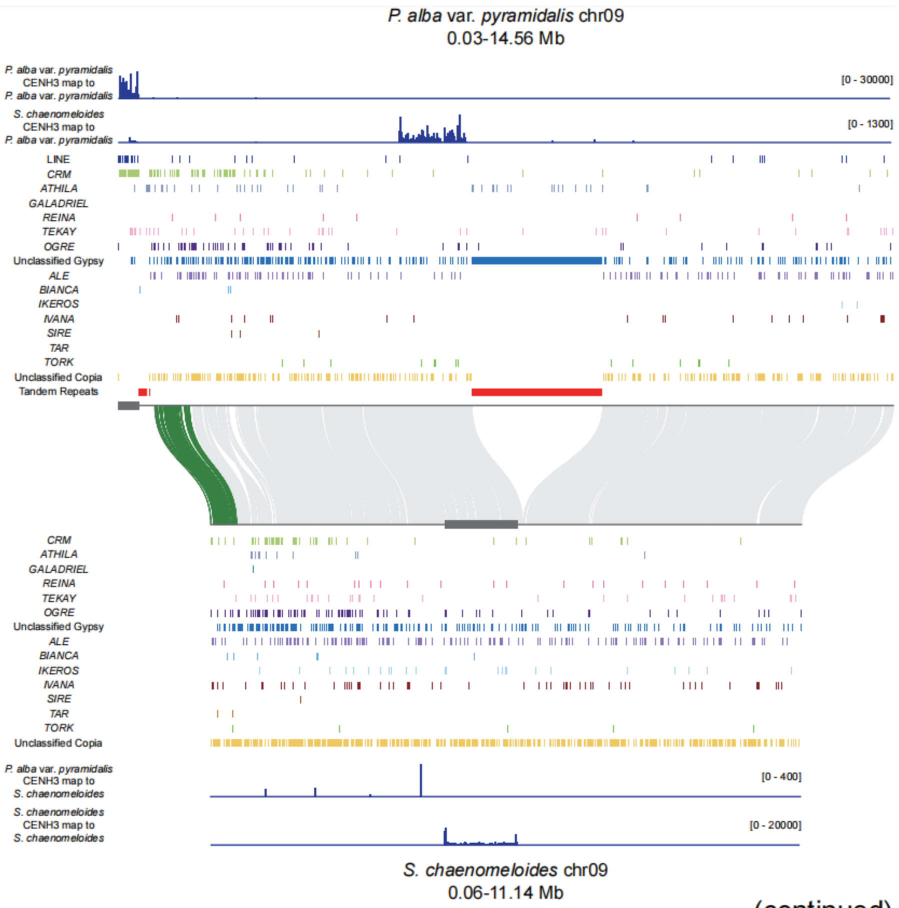

P. alba var. pyramidalis chr09
0.03-14.56 Mb
P. alba var. pyramidalis CENH3 map to P. alba var. pyramidalis
[0 - 30000]
S. chaenomeloides CENH3 map to P. alba var. pyramidalis
[0 - 1300]
P. alba var. pyramidalis CENH3 map to S. chaenomeloides
[0 - 400]
S. chaenomeloides CENH3 map to S. chaenomeloides
[0 - 20000]
S. chaenomeloides chr09
0.06-11.14 Mb

(continued)

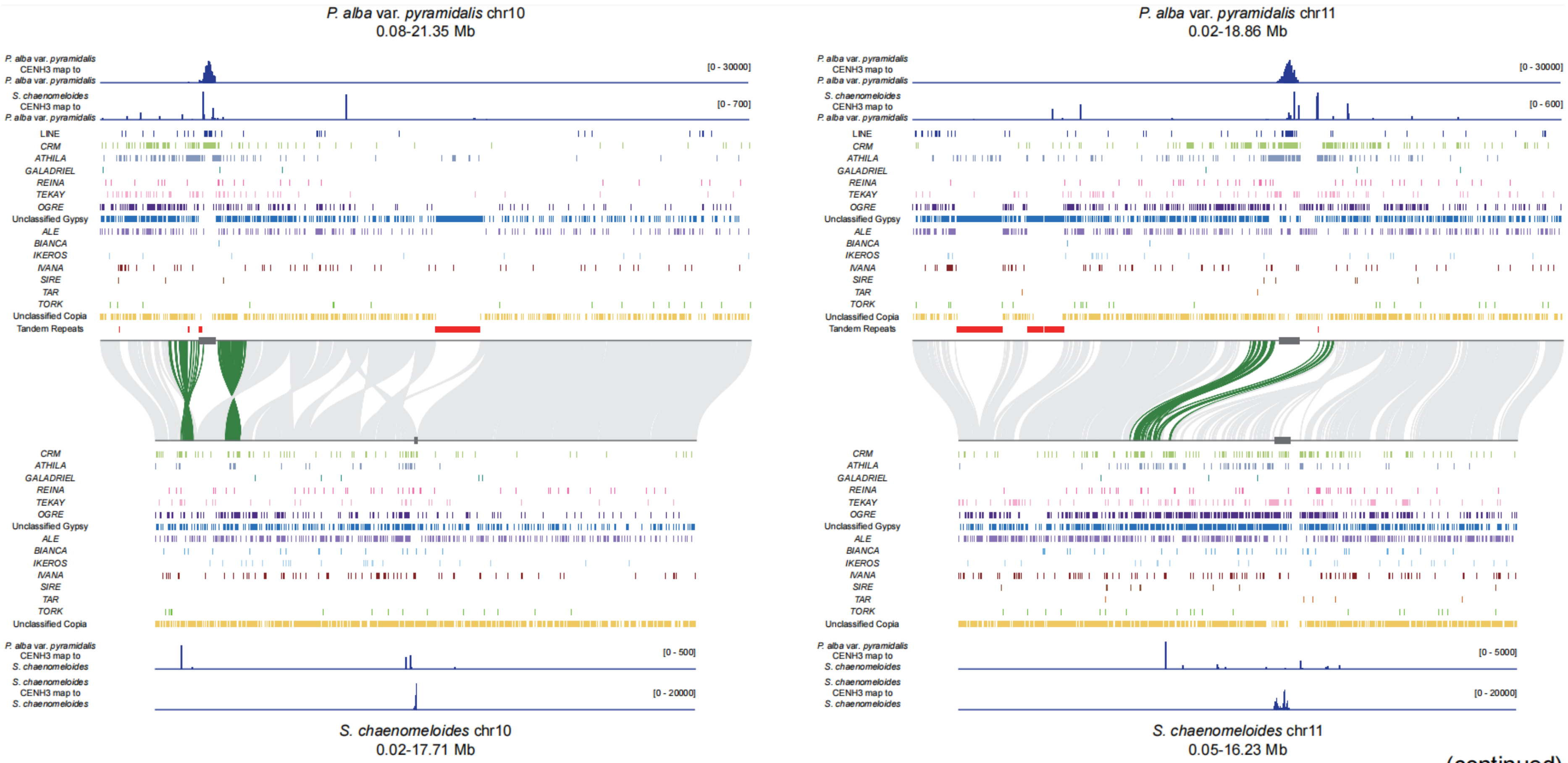
P. alba var. pyramidalis chr10
0.08-21.35 Mb
P. alba var. pyramidalis CENH3 map to P. alba var. pyramidalis
[0 - 30000]
S. chaenomeloides CENH3 map to P. alba var. pyramidalis
[0 - 700]
LINE
CRM
ATHILA
GALADRIEL
REINA
TEKAY
OGRE
Unclassified Gypsy
ALE
BIANCA
IKEROS
IVANA
SIRE
TAR
TORK
Unclassified Copia
Tandem Repeats
CRM
ATHILA
GALADRIEL
REINA
TEKAY
OGRE
Unclassified Gypsy
ALE
BIANCA
IKEROS
IVANA
SIRE
TAR
TORK
Unclassified Copia
P. alba var. pyramidalis CENH3 map to S. chaenomeloides
[0 - 500]
S. chaenomeloides CENH3 map to S. chaenomeloides
[0 - 20000]
S. chaenomeloides chr10
0.02-17.71 Mb
P. alba var. pyramidalis chr11
0.02-18.86 Mb
P. alba var. pyramidalis CENH3 map to P. alba var. pyramidalis
[0 - 30000]
S. chaenomeloides CENH3 map to P. alba var. pyramidalis
[0 - 600]
LINE
CRM
ATHILA
GALADRIEL
REINA
TEKAY
OGRE
Unclassified Gypsy
ALE
BIANCA
IKEROS
IVANA
SIRE
TAR
TORK
Unclassified Copia
Tandem Repeats
CRM
ATHILA
GALADRIEL
REINA
TEKAY
OGRE
Unclassified Gypsy
ALE
BIANCA
IKEROS
IVANA
SIRE
TAR
TORK
Unclassified Copia
P. alba var. pyramidalis CENH3 map to S. chaenomeloides
[0 - 5000]
S. chaenomeloides CENH3 map to S. chaenomeloides
[0 - 20000]
S. chaenomeloides chr11
0.05-16.23 Mb

(continued)

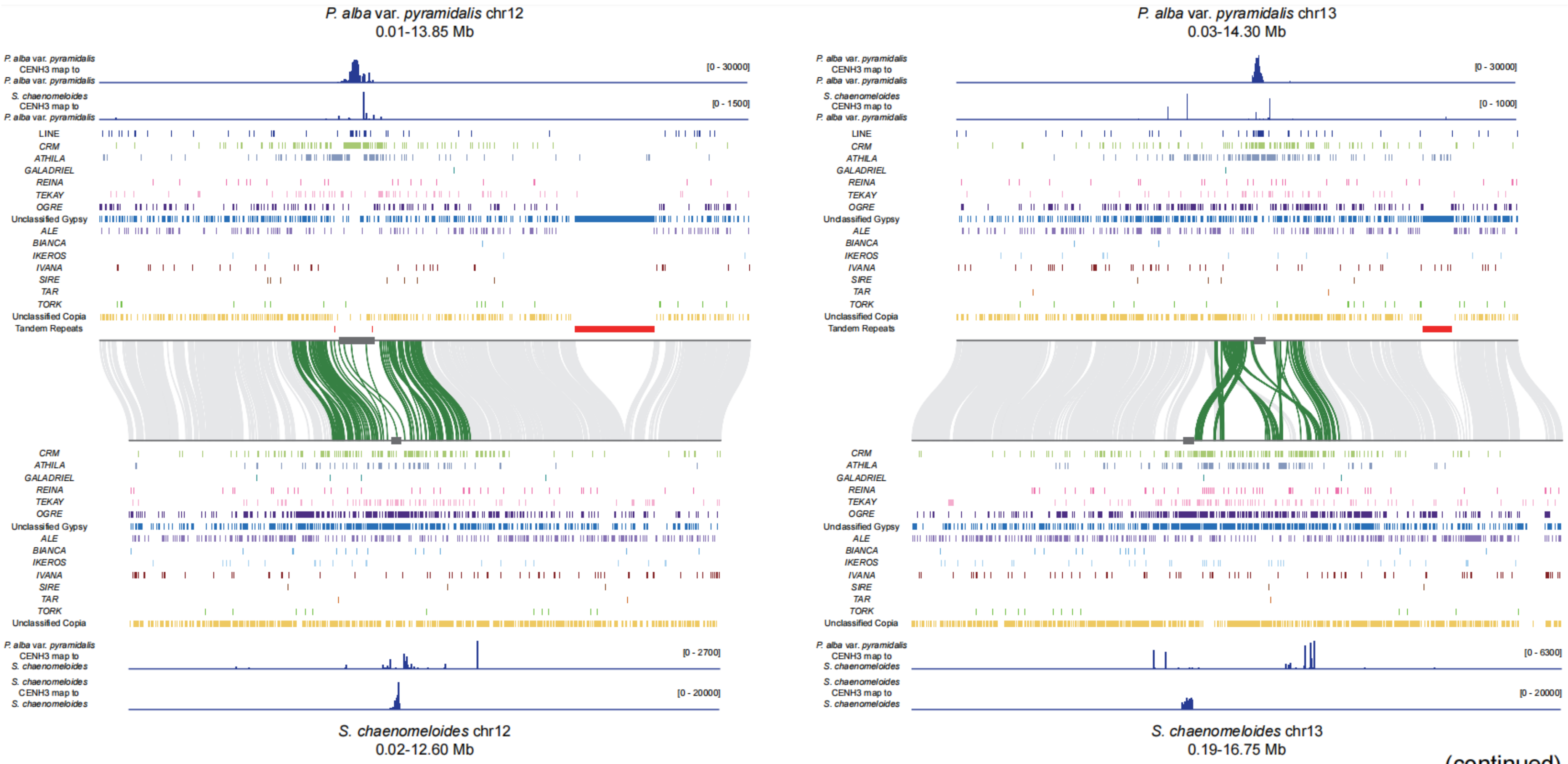

P. alba var. pyramidalis chr12
0.01-13.85 Mb
P. alba var. pyramidalis CENH3 map to P. alba var. pyramidalis
[0 - 30000]
S. chaenomeloides CENH3 map to P. alba var. pyramidalis
[0 - 1500]
LINE
CRM
ATHILA
GALADRIEL
REINA
TEKAY
OGRE
Unclassified Gypsy
ALE
BIANCA
IKEROS
IVANA
SIRE
TAR
TORK
Unclassified Copia
Tandem Repeats
P. alba var. pyramidalis CENH3 map to S. chaenomeloides
[0 - 2700]
S. chaenomeloides CENH3 map to S. chaenomeloides
[0 - 20000]
S. chaenomeloides chr12
0.02-12.60 Mb
P. alba var. pyramidalis chr13
0.03-14.30 Mb
[0 - 30000]
[0 - 1000]
[0 - 6300]
[0 - 20000]
S. chaenomeloides chr13
0.19-16.75 Mb

(continued)

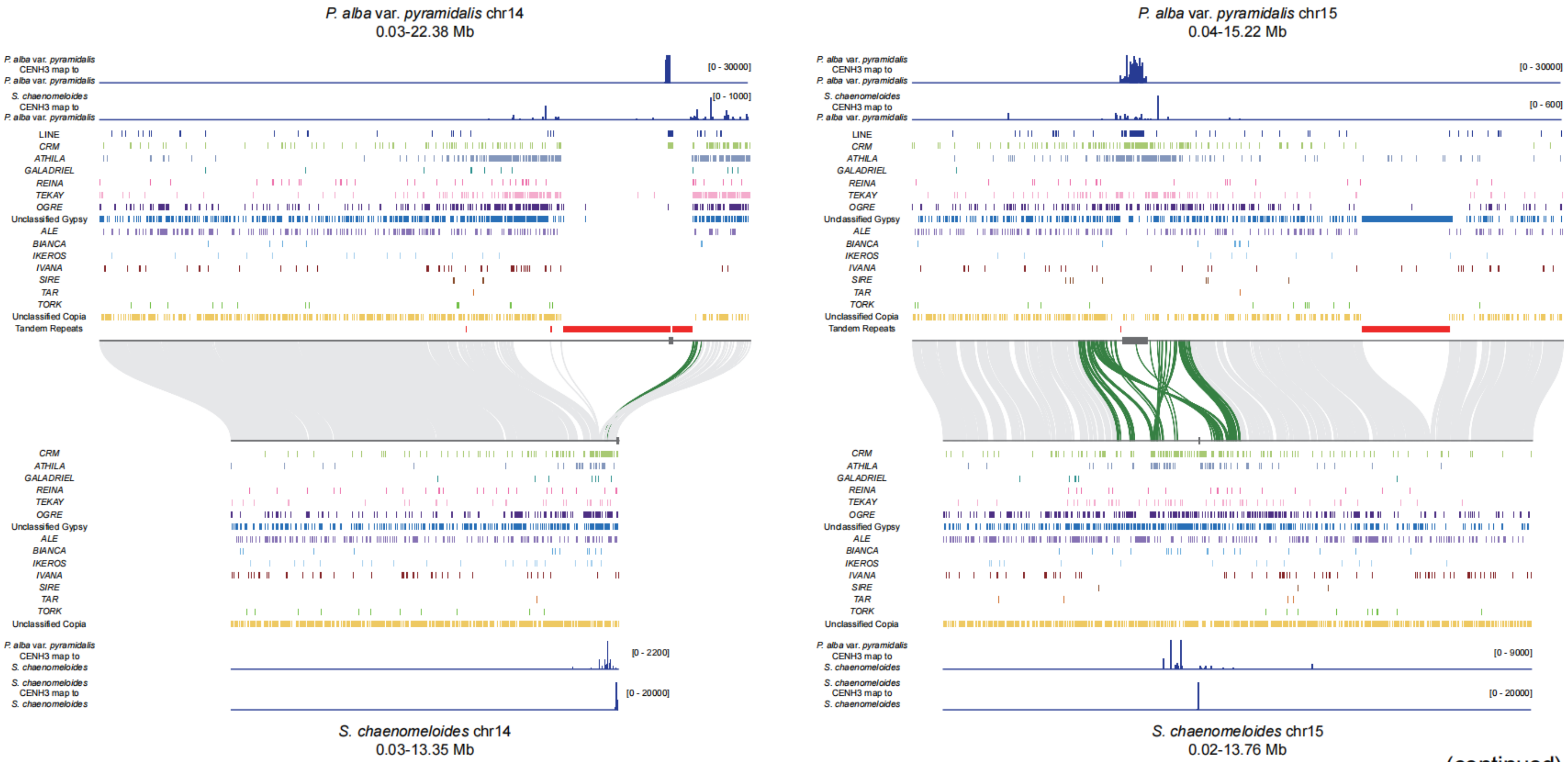

P. alba var. pyramidalis chr14
0.03-22.38 Mb
P. alba var. pyramidalis CENH3 map to P. alba var. pyramidalis
[0 - 30000]
S. chaenomeloides CENH3 map to P. alba var. pyramidalis
[0 - 1000]
LINE
CRM
ATHILA
GALADRIEL
REINA
TEKAY
OGRE
Unclassified Gypsy
ALE
BIANCA
IKEROS
IVANA
SIRE
TAR
TORK
Unclassified Copia
Tandem Repeats
P. alba var. pyramidalis CENH3 map to S. chaenomeloides
[0 - 2200]
S. chaenomeloides CENH3 map to S. chaenomeloides
[0 - 20000]
S. chaenomeloides chr14
0.03-13.35 Mb
P. alba var. pyramidalis chr15
0.04-15.22 Mb
[0 - 30000]
[0 - 600]
[0 - 9000]
[0 - 20000]
S. chaenomeloides chr15
0.02-13.76 Mb

(continued)

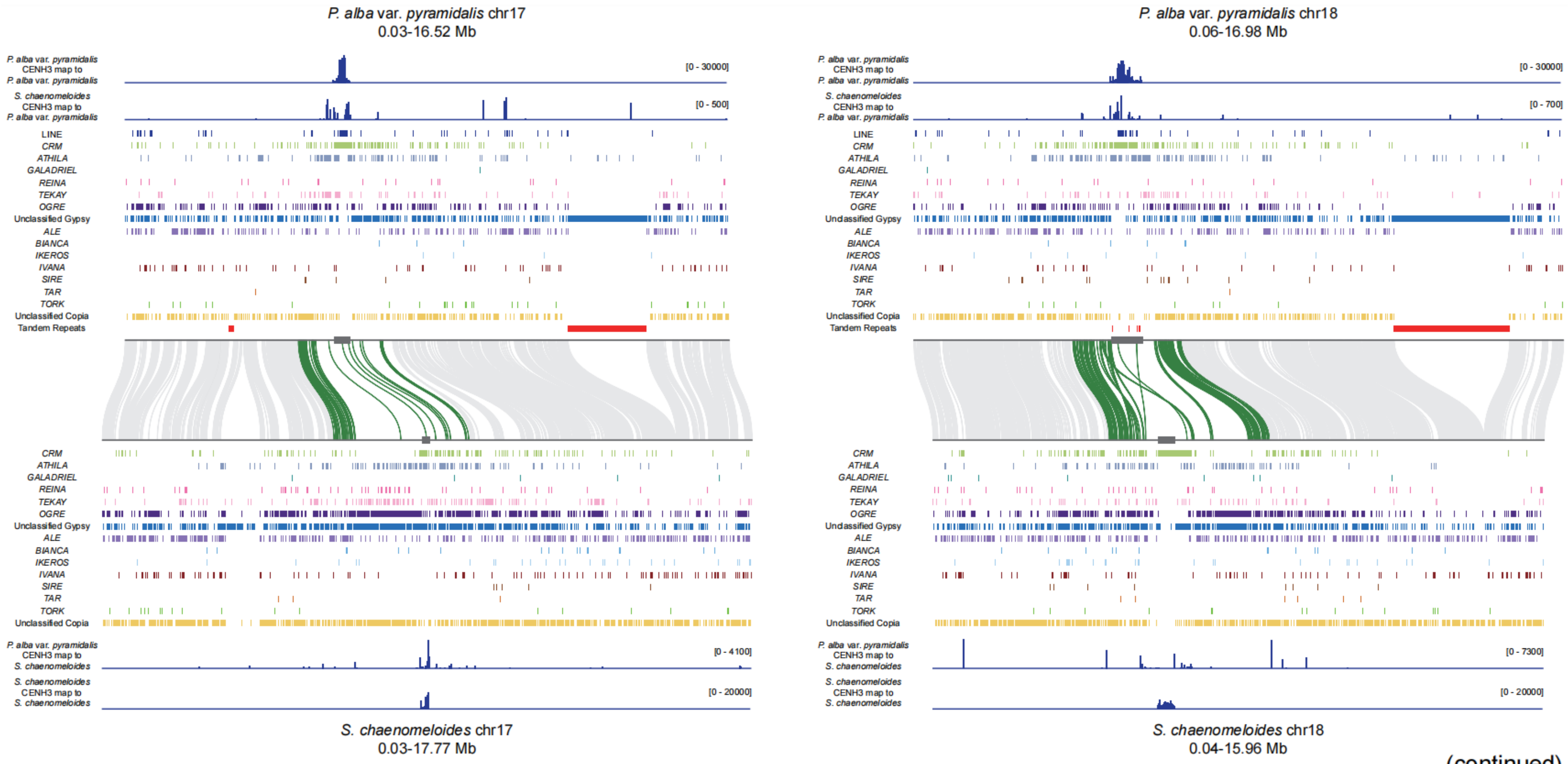
P. alba var. pyramidalis chr17
0.03-16.52 Mb
P. alba var. pyramidalis CENH3 map to P. alba var. pyramidalis
[0 - 30000]
S. chaenomeloides CENH3 map to P. alba var. pyramidalis
[0 - 500]
LINE
CRM
ATHILA
GALADRIEL
REINA
TEKAY
OGRE
Unclassified Gypsy
ALE
BIANCA
IKEROS
IVANA
SIRE
TAR
TORK
Unclassified Copia
Tandem Repeats
CRM
ATHILA
GALADRIEL
REINA
TEKAY
OGRE
Unclassified Gypsy
ALE
BIANCA
IKEROS
IVANA
SIRE
TAR
TORK
Unclassified Copia
P. alba var. pyramidalis CENH3 map to S. chaenomeloides
[0 - 4100]
S. chaenomeloides CENH3 map to S. chaenomeloides
[0 - 20000]
S. chaenomeloides chr17
0.03-17.77 Mb
P. alba var. pyramidalis chr18
0.06-16.98 Mb
P. alba var. pyramidalis CENH3 map to P. alba var. pyramidalis
[0 - 30000]
S. chaenomeloides CENH3 map to P. alba var. pyramidalis
[0 - 700]
LINE
CRM
ATHILA
GALADRIEL
REINA
TEKAY
OGRE
Unclassified Gypsy
ALE
BIANCA
IKEROS
IVANA
SIRE
TAR
TORK
Unclassified Copia
Tandem Repeats
CRM
ATHILA
GALADRIEL
REINA
TEKAY
OGRE
Unclassified Gypsy
ALE
BIANCA
IKEROS
IVANA
SIRE
TAR
TORK
Unclassified Copia
P. alba var. pyramidalis CENH3 map to S. chaenomeloides
[0 - 7300]
S. chaenomeloides CENH3 map to S. chaenomeloides
[0 - 20000]
S. chaenomeloides chr18
0.04-15.96 Mb

(continued)

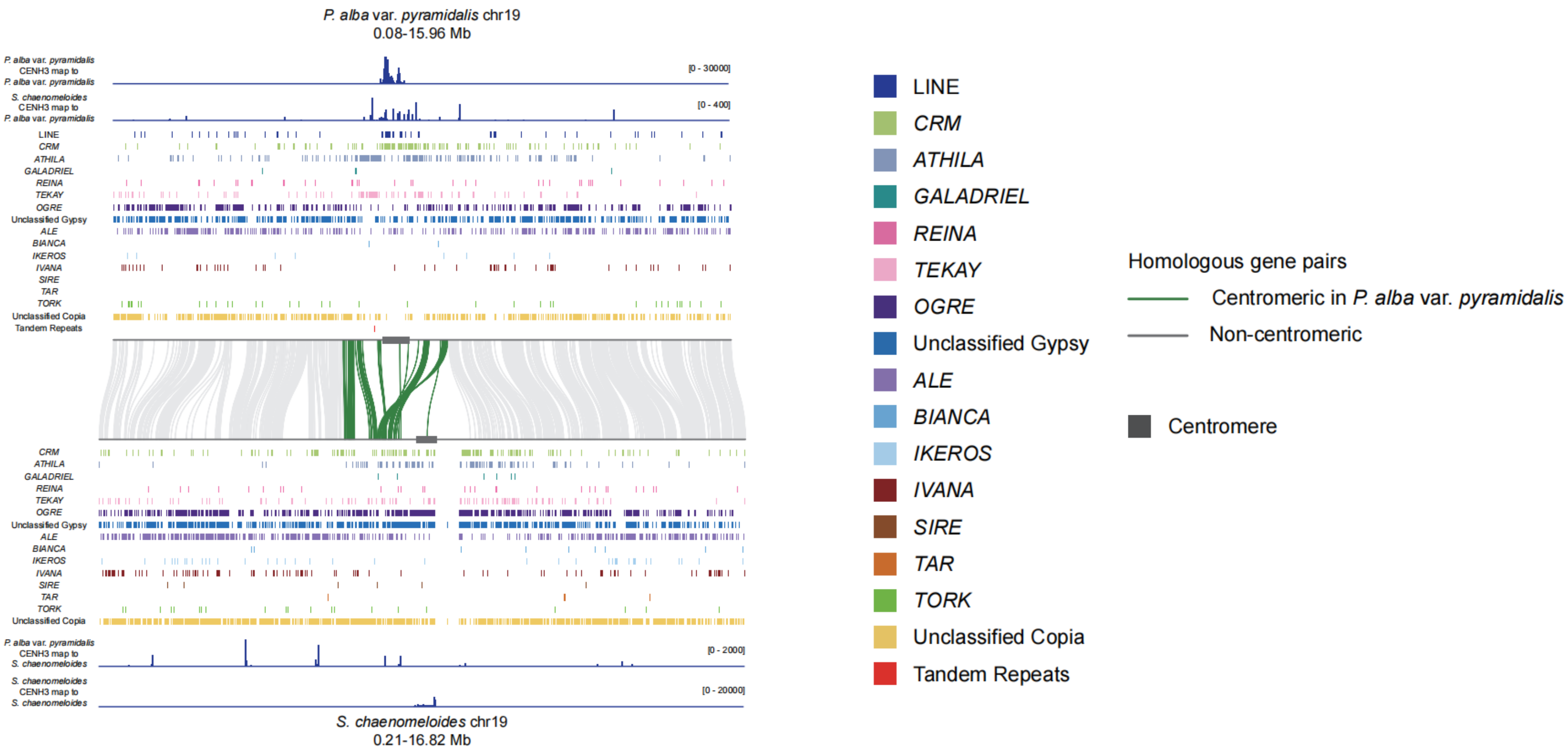

Fig. S32. Synteny analysis of homologous chromosomes between *P. alba* var. *pyramidalis* and *S. chaenomeloides*. The two straight lines in the middle represent homologous chromosomes scaled by chromosome length and dark grey boxes on lines represent corresponding centromeres. Grey lines connecting chromosomes represent homologous genes pairs between *P. alba* var. *pyramidalis* and *S. chaenomeloides* haplotype I

genome, and green lines represent homologous genes located within centromeres and peri-centromeric regions of *P. alba* var. *pyramidalis*. Histograms represent the distribution of each type of LTRs and LINE elements from *P. alba* var. *pyramidalis* and *S. chaenomeloides*. CUT&Tag data coverage from both *S. chaenomeloides* and *P. alba* var. *pyramidalis* to the *P. alba* var. p*yramidalis* genome are shown at the top. CUT&Tag data coverage from both *S. chaenomeloides* and *P. alba* var. *pyramidalis* to the *S. chaenomeloides* genome are shown at the bottom.

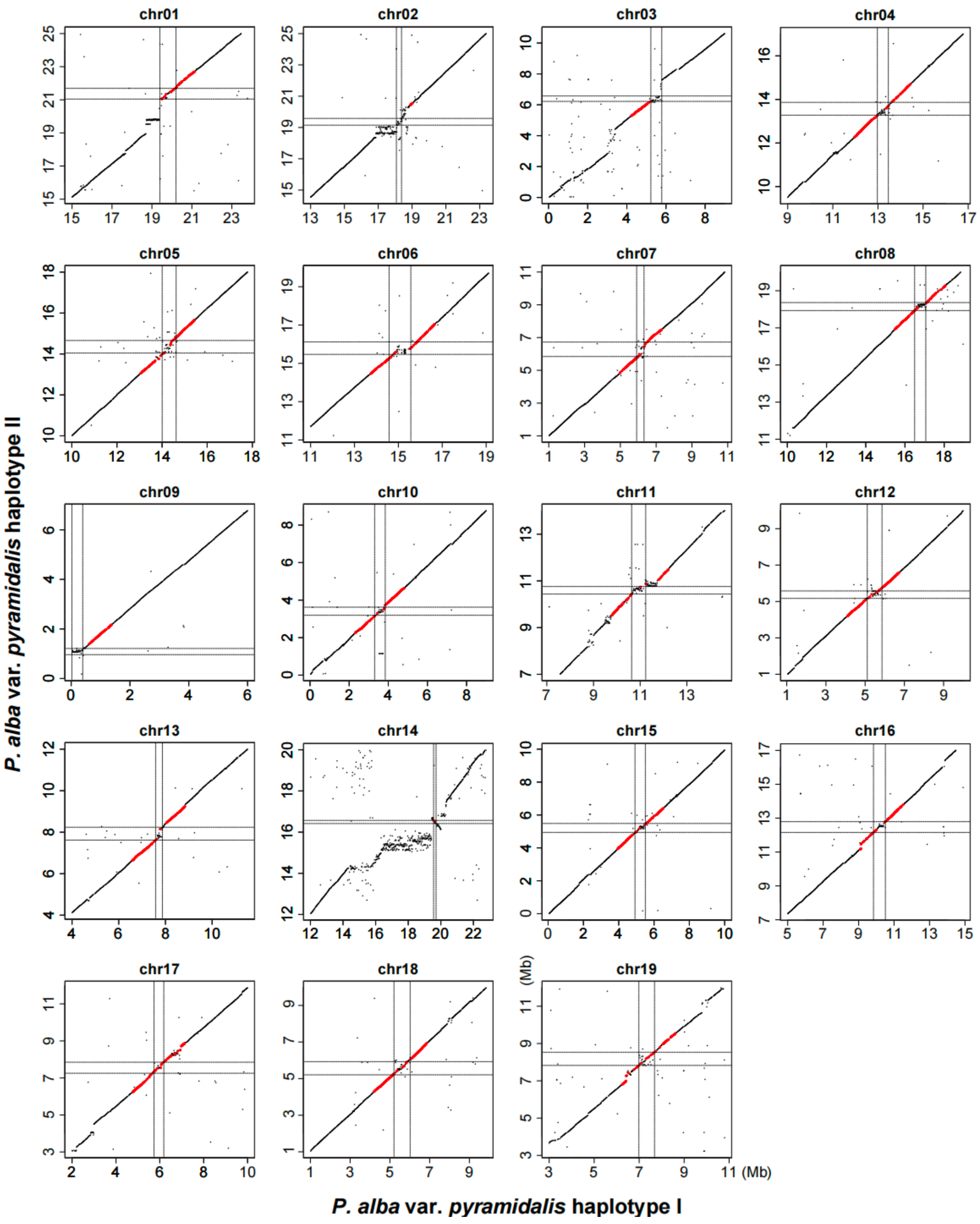

Fig. S33. Dot plot alignments between haplotype chromosomes of *P. alba* var. *pyramidalis*. Sequence synteny between haplotypes are shown as black dots, while red dots represent homologous genes located within centromeres and pericentromeres of haplotype I.

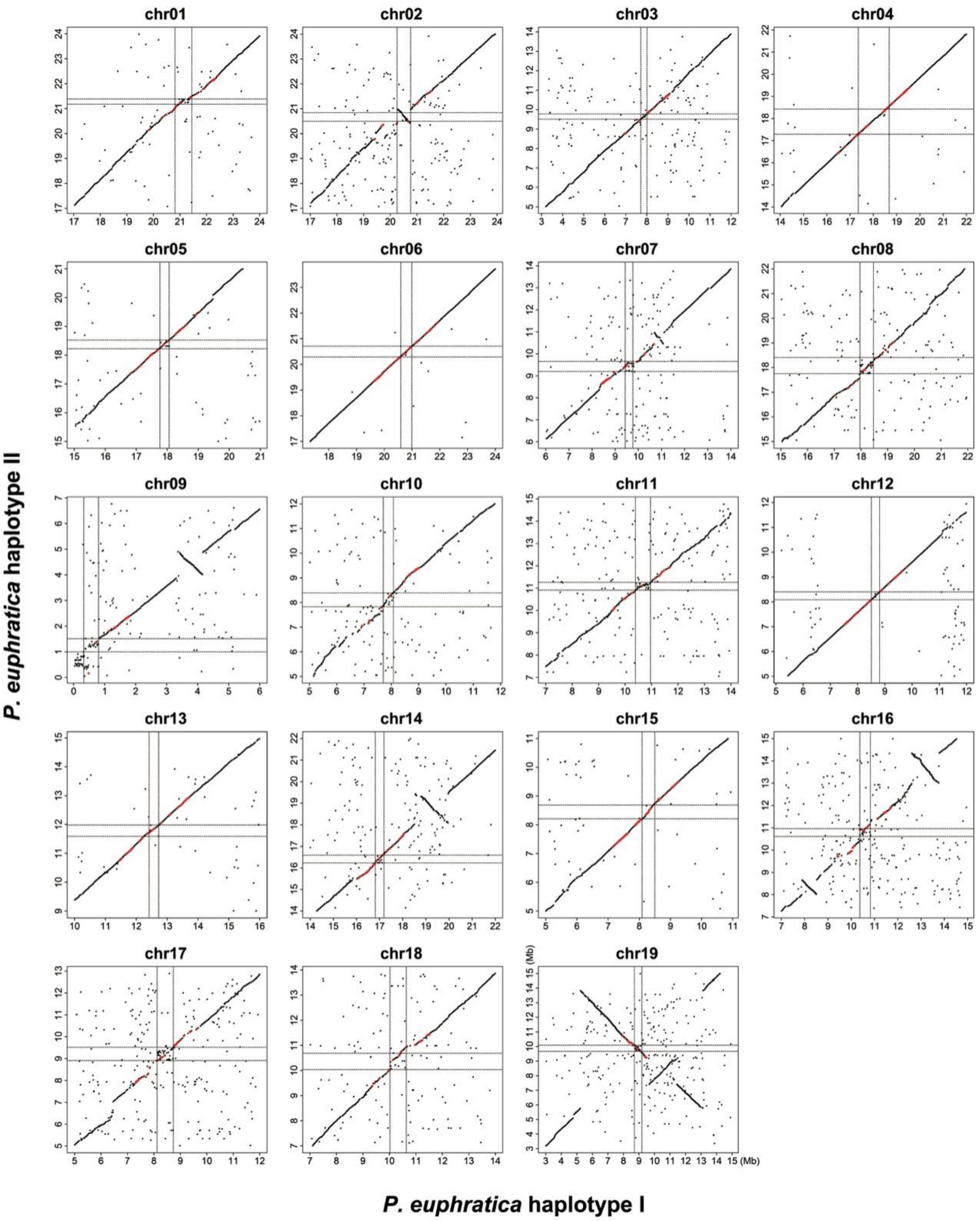

Fig. S34. Dot plot alignments between haplotype chromosomes of *P. euphratica*. Sequence synteny between haplotypes are shown as black dots, while red dots represent homologous genes located within centromeres and pericentromeres of haplotype I.

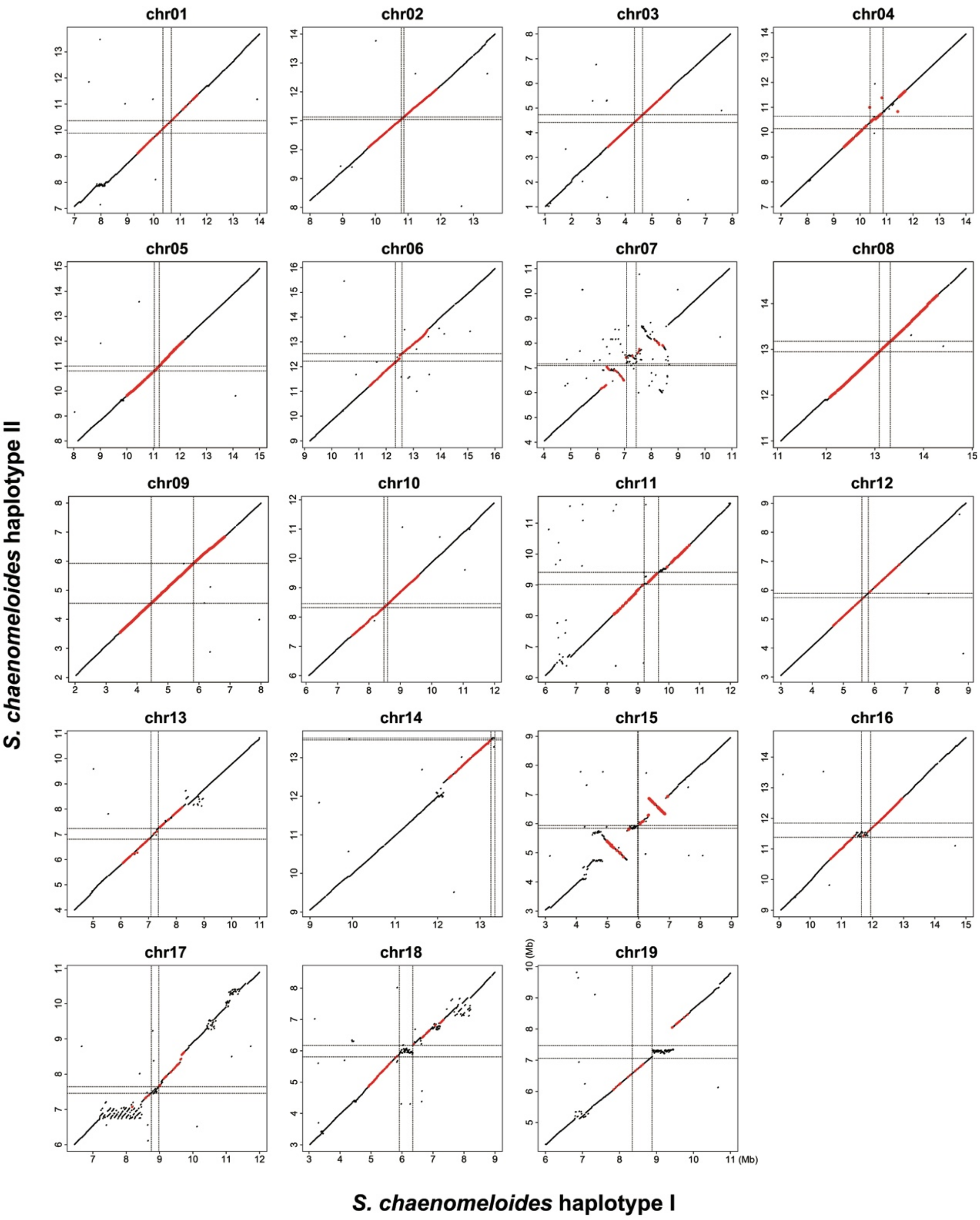

Fig. S35. Dot plot alignments between haplotype chromosomes of *S. chaenomeloides*. Sequence synteny between haplotypes are shown as black dots, while red dots represent homologous genes located within centromeres and pericentromeres of haplotype I.

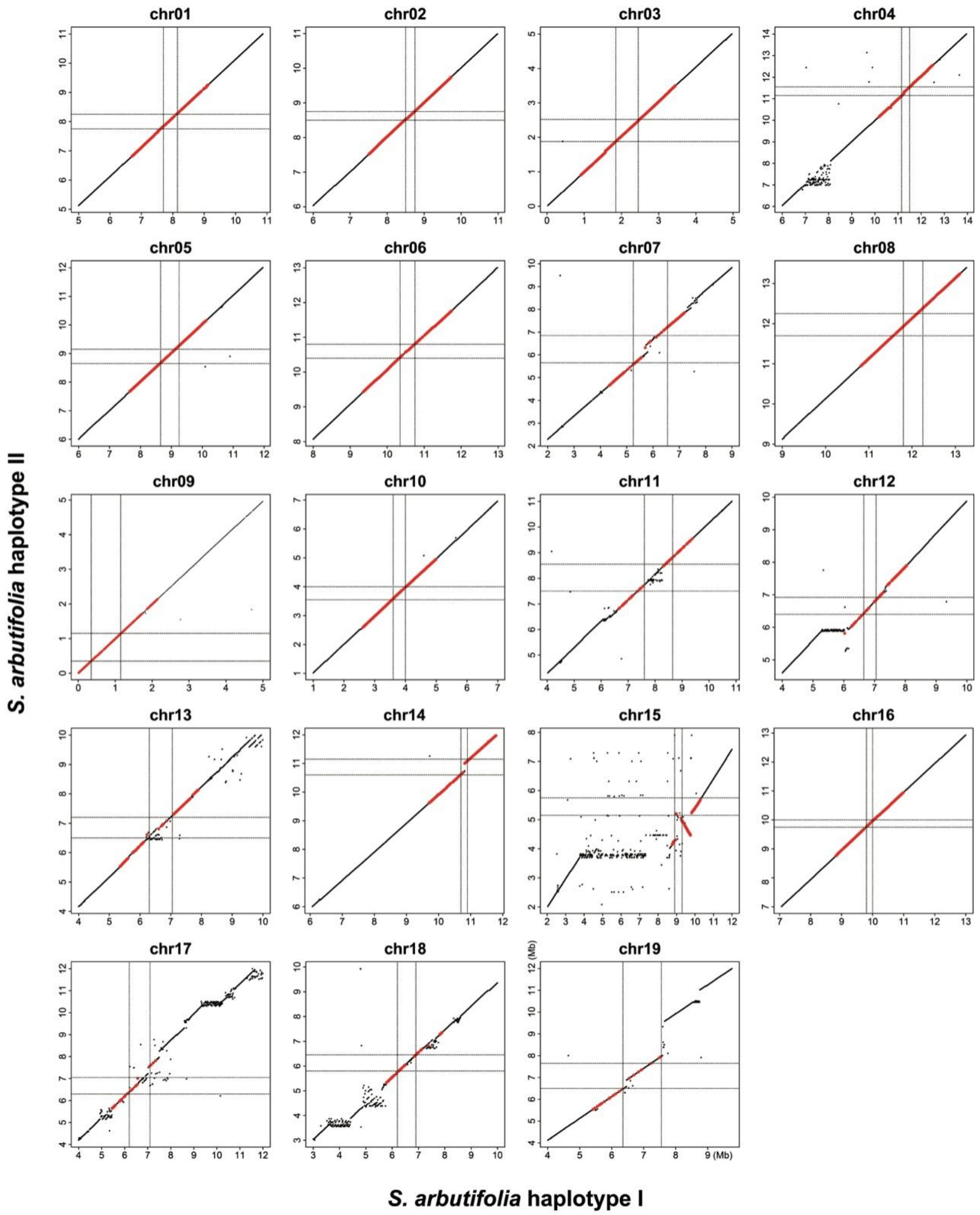

Fig. S36. Dot plot alignments between haplotype chromosomes of *S. arbutifolia*. Sequence synteny between haplotypes are shown as black dots, while red dots represent homologous genes located within centromeres and pericentromeres of haplotype I.

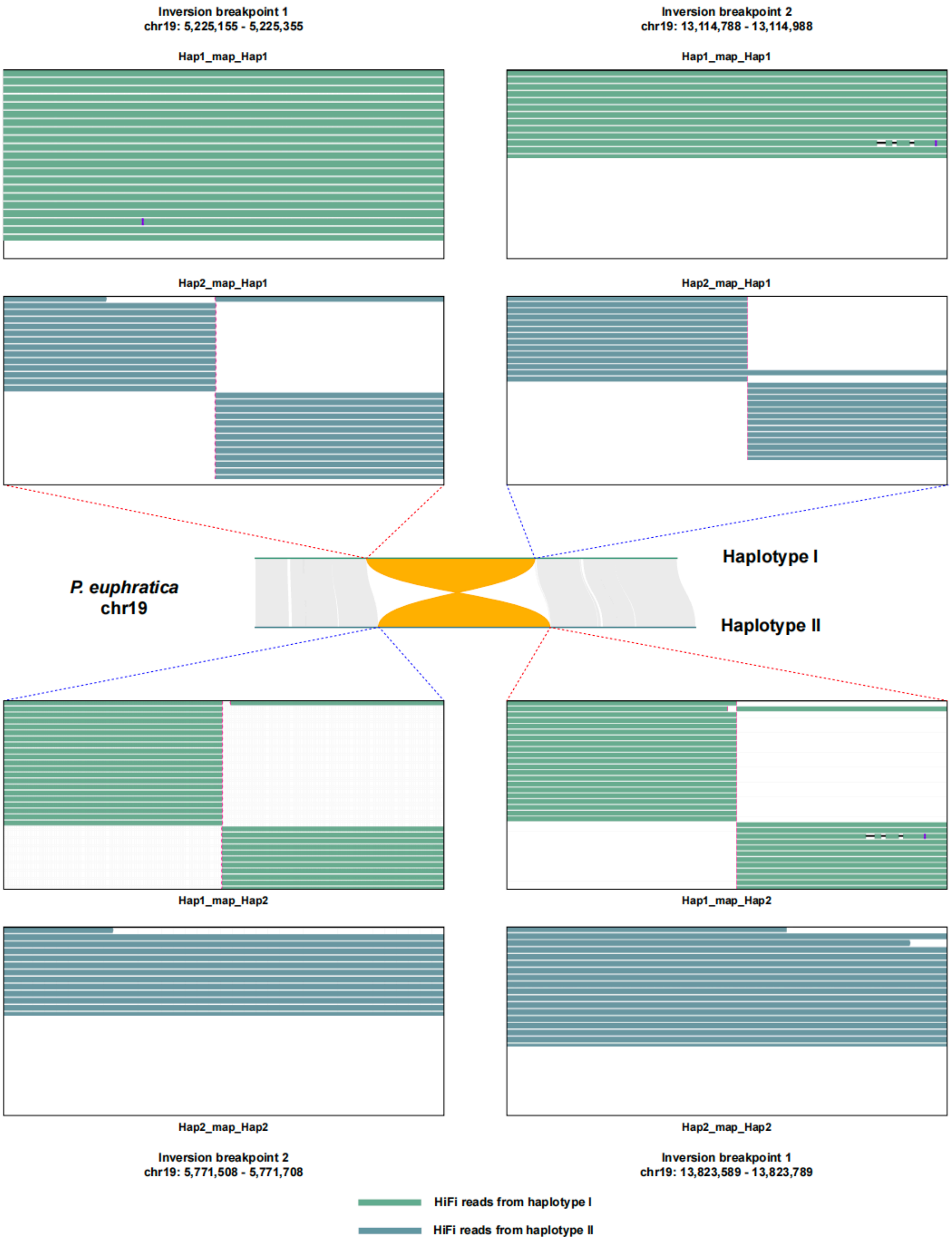

Fig. S37. Example of HiFi validation of the insertion event between haplotype genomes in *P. euphratica* chr19. The upper four rectangles show alignments of HiFi reads on the two inversion breakpoints obtained from IGV (closed up by red and blue dashed lines, respectively), including mapping HiFi reads in haplotype I against the haplotype I genome (Hap1_map_Hap1) and mapping HiFi reads in haplotype II against the haplotype I genome (Hap2_map_Hap1). HiFi reads in haplotype I can cover

inversion breakpoints, while HiFi reads in haplotype II are truncated at the inversion breakpoint. The middle panel shows genome alignment between chr19 of haplotype I and haplotype II. Grey blocks connecting chromosomes represent homologous regions, and the orange block represent the inversion event. The lower four rectangles show alignments of HiFi reads on the two inversion breakpoints in the haplotype II genome. The data mapping strategy is similar to those in the upper track.

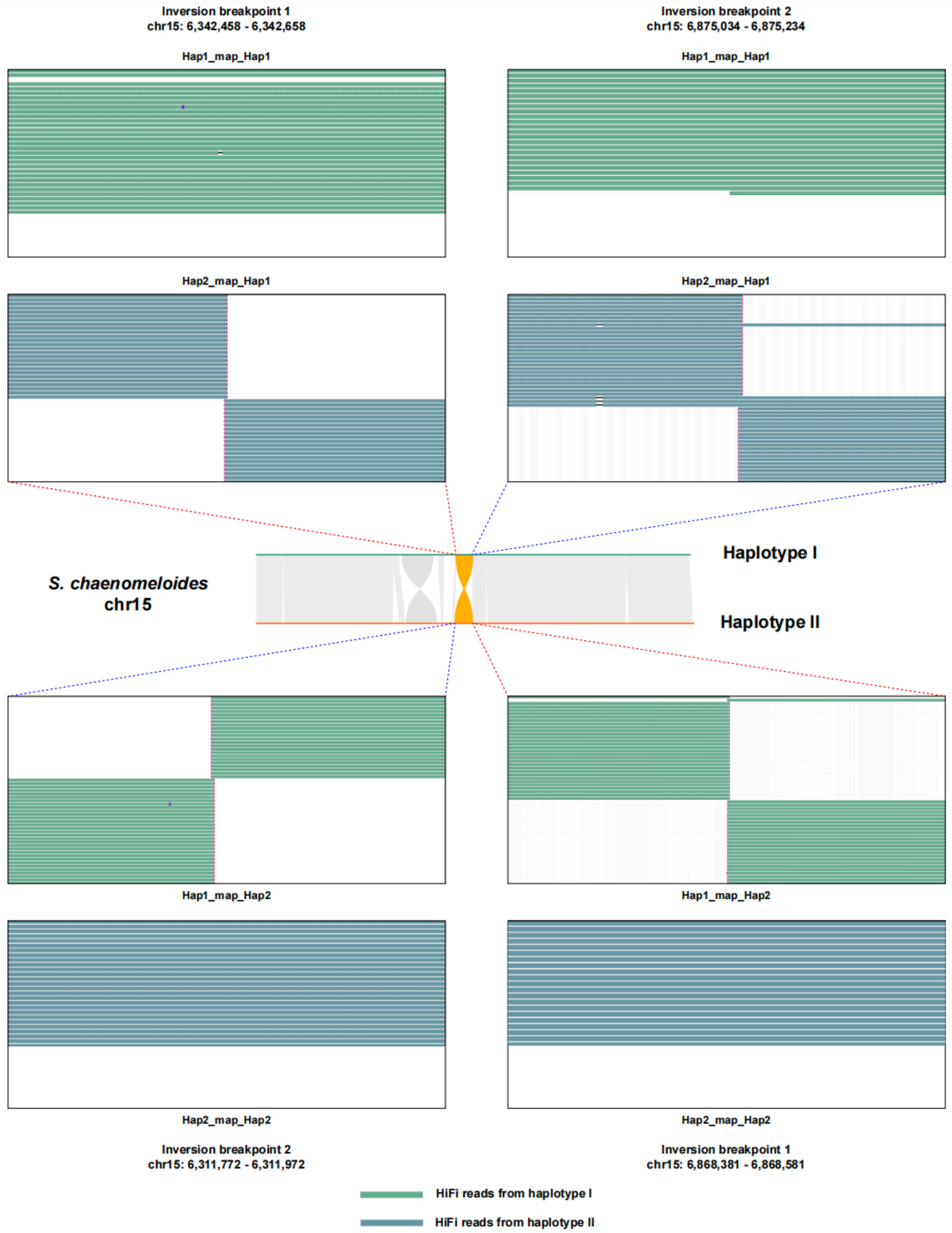

Fig. S38. Example of HiFi validation of the insertion event between haplotype genomes in *S. chaenomeloides* chr15. The upper four rectangles show alignments of HiFi reads on the two inversion breakpoints obtained from IGV (closed up by red and blue dashed lines, respectively), including mapping HiFi reads in haplotype I against the haplotype I genome (Hap1_map_Hap1) and mapping HiFi reads in haplotype II against the haplotype I genome (Hap2_map_Hap1). HiFi reads in haplotype I can cover

inversion breakpoints, while HiFi reads in haplotype II are truncated at the inversion breakpoint. The middle panel shows genome alignment between chr15 of haplotype I and haplotype II. Grey blocks connecting chromosomes represent homologous regions, and the orange block represent the inversion event. The lower four rectangles show alignments of HiFi reads on the two inversion breakpoints in the haplotype II genome. The data mapping strategy is similar to those in the upper track.

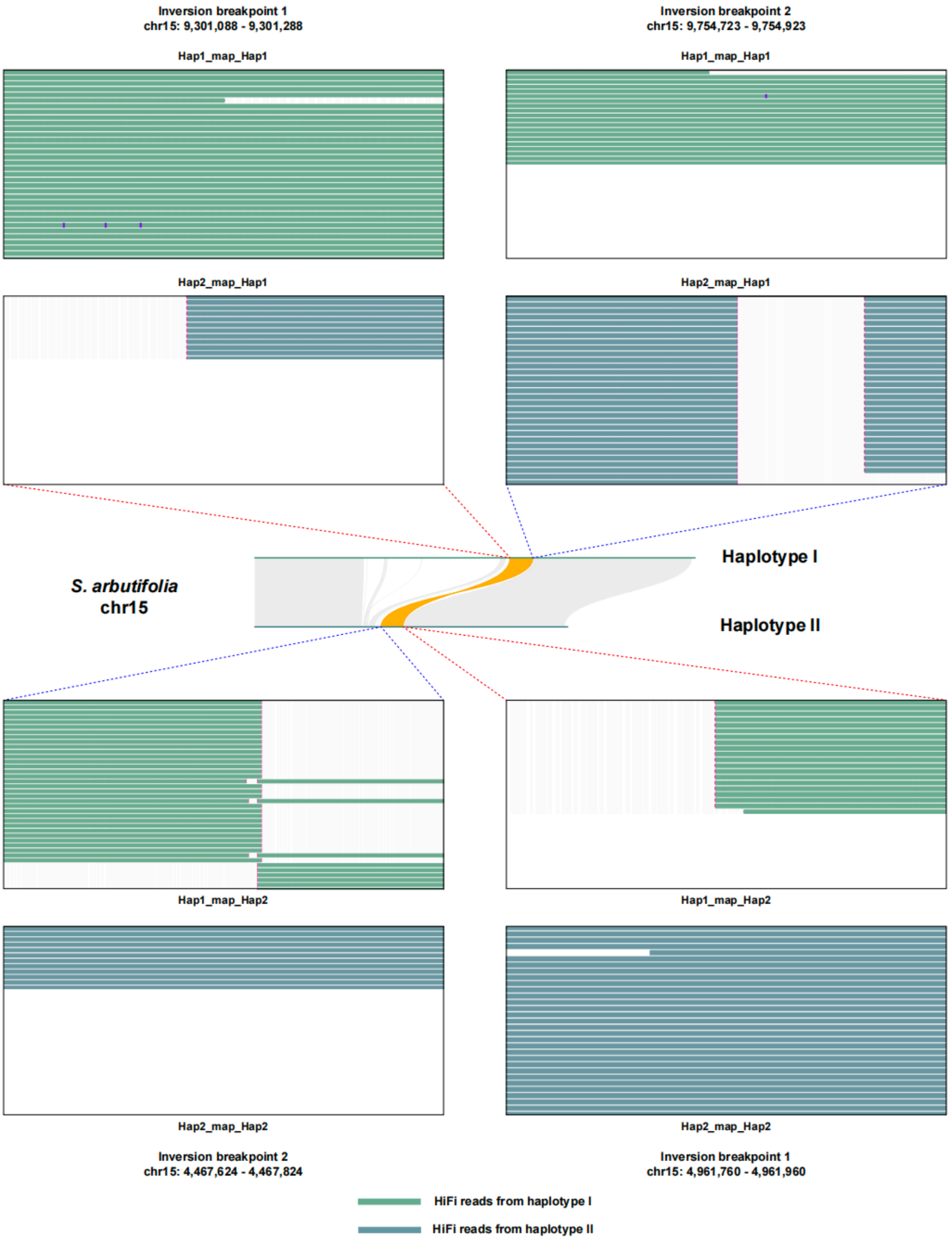

Fig. S39. Example of HiFi validation of the insertion event between haplotype genomes in *S. arbutifolia* chr15. The upper four rectangles show alignments of HiFi reads on the two inversion breakpoints obtained from IGV (closed up by red and blue dashed lines, respectively), including mapping HiFi reads in haplotype I against the haplotype I genome (Hap1_map_Hap1) and mapping HiFi reads in haplotype II against the haplotype I genome (Hap2_map_Hap1). HiFi reads in haplotype I can cover

inversion breakpoints, while HiFi reads in haplotype II are truncated at the inversion breakpoint. The middle panel shows genome alignment between chr15 of haplotype I and haplotype II. Grey blocks connecting chromosomes represent homologous regions, and the orange block represent the inversion event. The lower four rectangles show alignments of HiFi reads on the two inversion breakpoints in the haplotype II genome. The data mapping strategy is similar to those in the upper track.

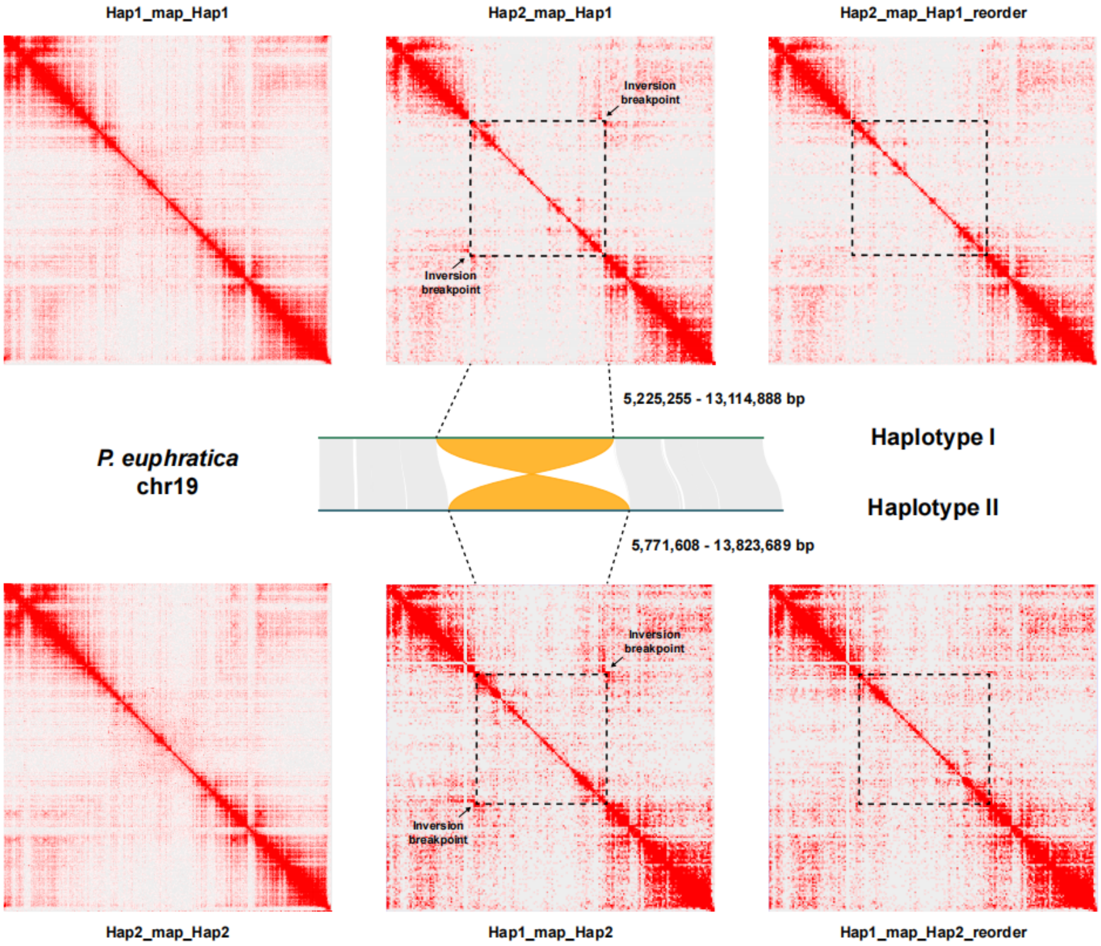

Fig. S40. Example of Hi-C validation of the insertion event between haplotype genomes in *P. euphratica* chr19. The upper three heatmaps show a chromatin interaction matrix, including mapping Hi-C data in haplotype I against the haplotype I genome (Hap1_map_Hap1), mapping Hi-C data in haplotype II against the haplotype I genome (Hap2_map_Hap1) and mapping Hi-C data in haplotype II against reordered genomic sequences of haplotype I genome based on these inversions (Hap2_map_Hap1_reorder). The position of the inversion event is shown as the rectangle, and inversion breakpoints are shown by arrows. The middle panel shows genome alignment between chr19 of haplotype I and haplotype II. Grey blocks connecting chromosomes represent homologous regions, and the orange block represent the inversion event. The lower three heatmaps show a chromatin interaction matrix with mapping Hi-C data against the haplotype II genome. The data mapping strategy is similar to those in the upper track. The Hi-C heatmaps are shown at 25 kb resolution.

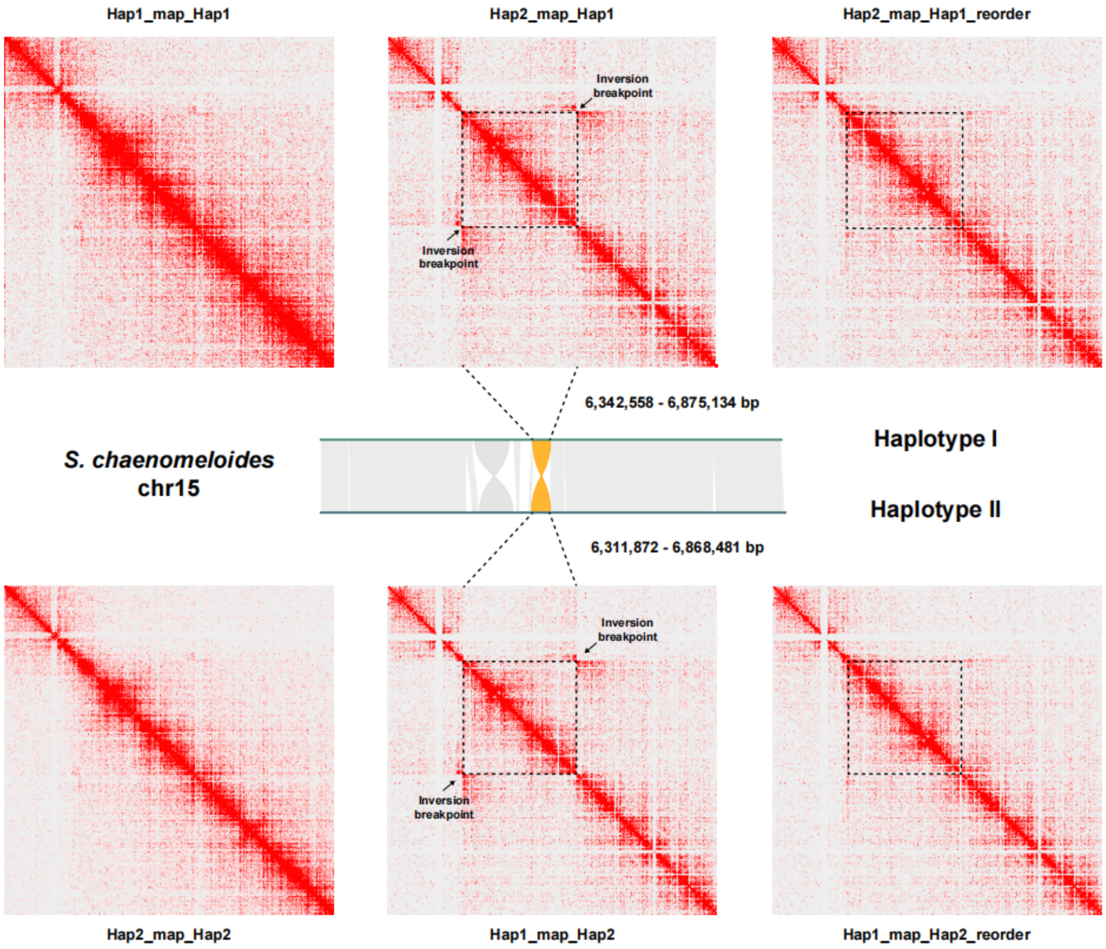

Fig. S41. Example of Hi-C validation of the insertion event between haplotype genomes in *S. chaenomeloides* chr15. The upper three heatmaps show a chromatin interaction matrix, including mapping Hi-C data in haplotype I against the haplotype I genome (Hap1_map_Hap1), mapping Hi-C data in haplotype II against the haplotype I genome (Hap2_map_Hap1) and mapping Hi-C data in haplotype II against reordered genomic sequences of haplotype I genome based on these inversions (Hap2_map_Hap1_reorder). The position of the inversion event is shown as the rectangle, and inversion breakpoints are shown by arrows. The middle panel shows genome alignment between chr15 of haplotype I and haplotype II. Grey blocks connecting chromosomes represent homologous regions, and the orange block represent the inversion event. The lower three heatmaps show a chromatin interaction matrix with mapping Hi-C data against the haplotype II genome. The data mapping strategy is similar to those in the upper track. The Hi-C heatmaps are shown at 25 kb resolution.

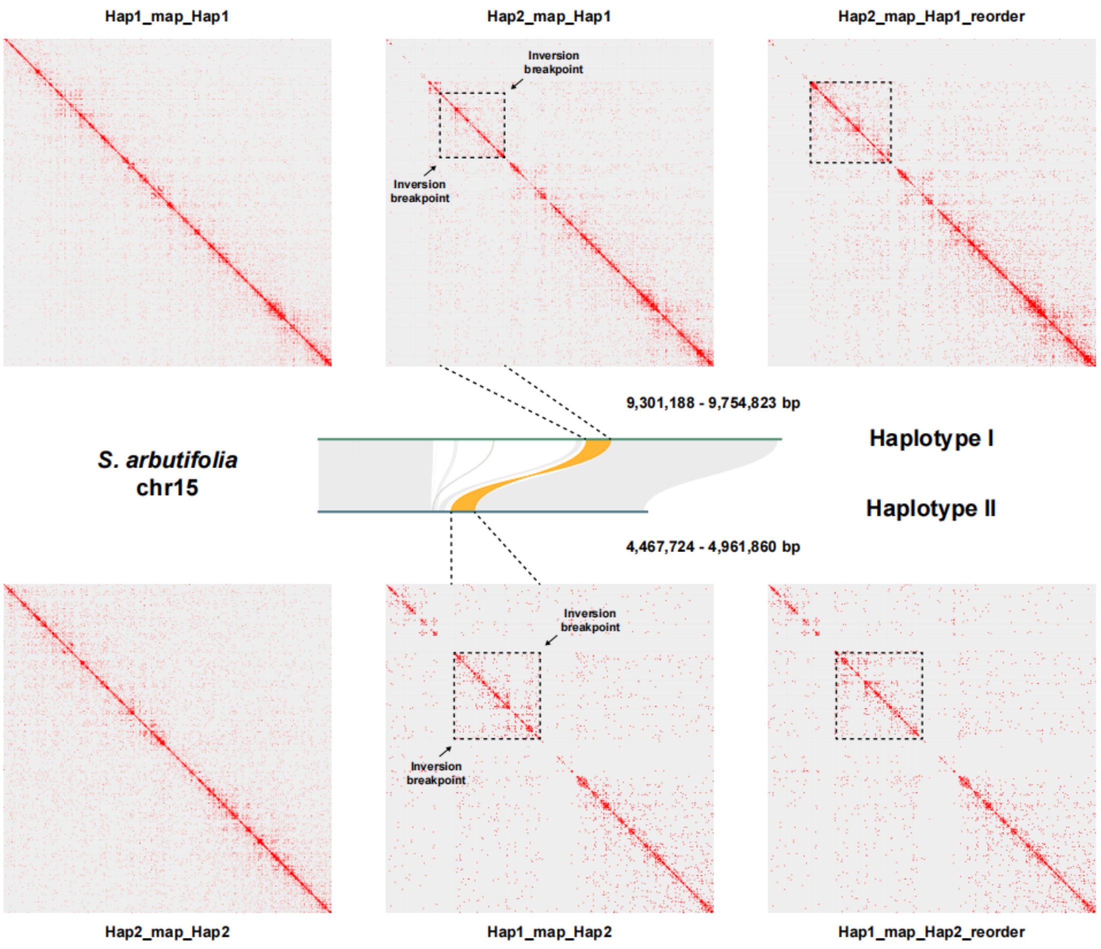

Fig. S42. Example of Hi-C validation of the insertion event between haplotype genomes in *S. arbutifolia* chr15. The upper three heatmaps show a chromatin interaction matrix, including mapping Hi-C data in haplotype I against the haplotype I genome (Hap1_map_Hap1), mapping Hi-C data in haplotype II against the haplotype I genome (Hap2_map_Hap1) and mapping Hi-C data in haplotype II against reordered genomic sequences of haplotype I genome based on these inversions (Hap2_map_Hap1_reorder). The position of the inversion event is shown as the rectangle, and inversion breakpoints are shown by arrows. The middle panel shows genome alignment between chr15 of haplotype I and haplotype II. Grey blocks connecting chromosomes represent homologous regions, and the orange block represent the inversion event. The lower three heatmaps show a chromatin interaction matrix with mapping Hi-C data against the haplotype II genome. The data mapping strategy is similar to those in the upper track. The Hi-C heatmaps are shown at 5 kb resolution.

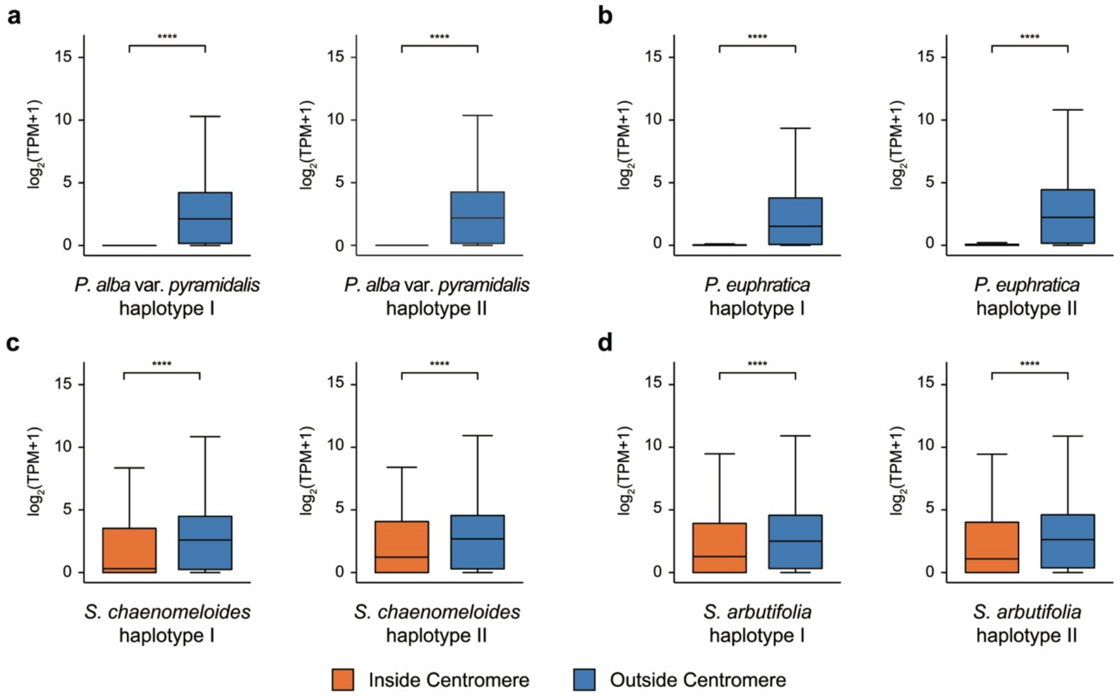

Fig. S43. Boxplots of expression comparison between centromeric genes and non-centromeric genes of *P. alba* var. *pyramidalis* (a), *P. euphratica* (b), *S. chaenomeloides* (c) and *S. arbutifolia* (d). Asterisks represent significant differences (two-tailed Wilcoxon rank-sum test, ****P <= 0.0001, ***P <= 0.001, **P <= 0.01, *P <= 0.05, ns: not significant).

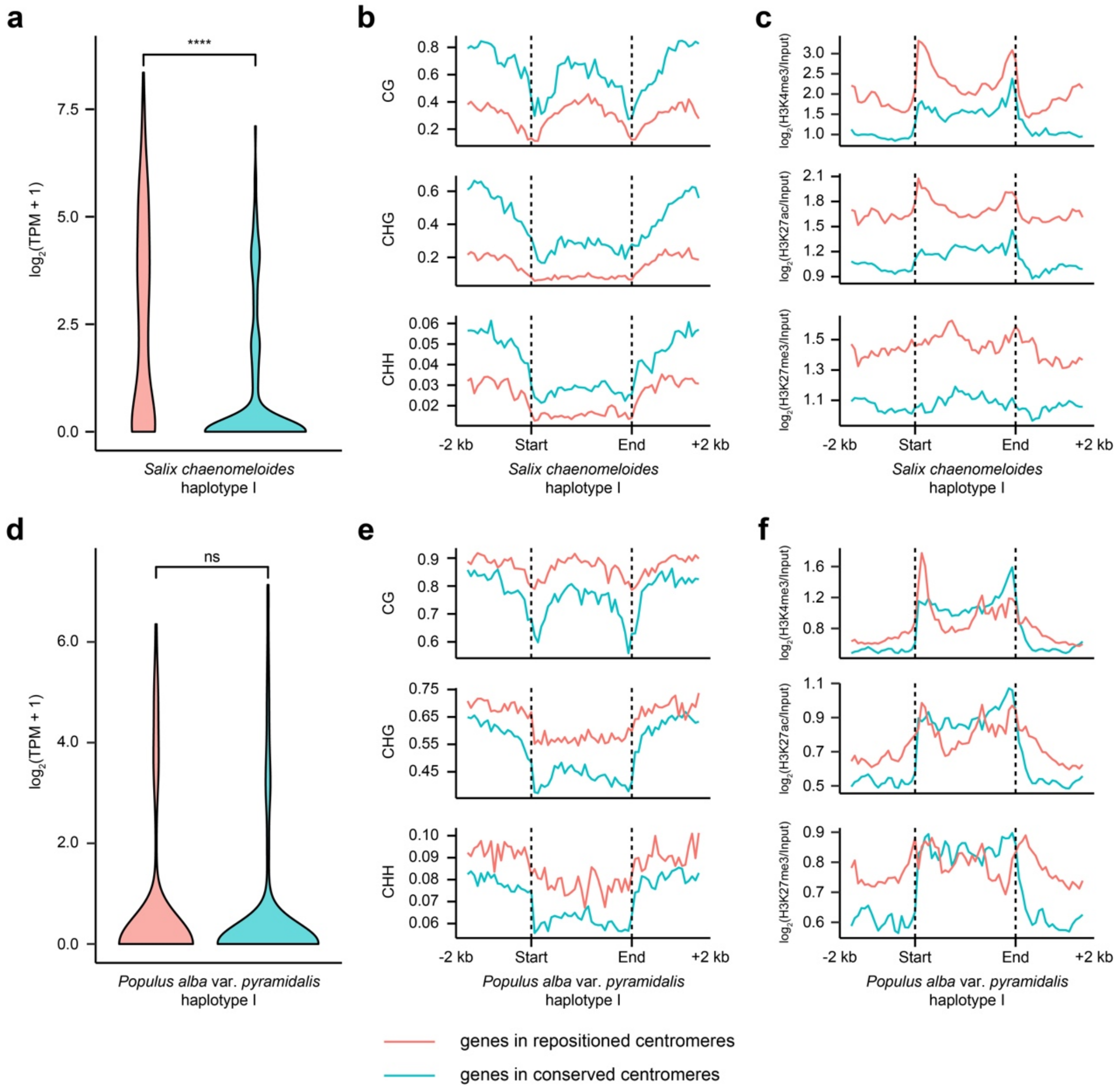

Fig. S44. Repositioned centromeres have higher-expressed genes. a Gene expression level comparison between genes in repositioned centromeres and conserved centromeres in *S. chaenomeloides*. **b** Metaprofiles of methylation levels for genes in repositioned centromeres and conserved centromeres in *S. chaenomeloides*. **c** Metaprofiles of histone modification levels for genes in repositioned centromeres and conserved centromeres in *S. chaenomeloides*. **d** Same as **a** but showing the comparison in *P. alba* var. *pyramidalis*. **e** and **f** Same as **b** and **c** but showing metaprofles in *P. alba* var. *pyramidalis*. Asterisks represent significant differences (two-tailed Wilcoxon rank-sum test, ****P <= 0.0001, ***P <= 0.001, **P <= 0.01, *P <= 0.05, ns: not significant).
